# Supplementary material for: Implementation of safety management systems (SMS) in healthcare: a systematic review and international comparison
Source: BMJ Open. 2026 May 12;16(5):e107772. doi: 10.1136/bmjopen-2025-107772 (PMC13223942; doi:10.1136/bmjopen-2025-107772)
Supplement: Supplementary data [file bmjopen-16-5-s001.pdf]

Supplementary material 1: Protocol

The implementation of Safety Management Systems (SMS) in healthcare

Systematic review protocol

Version 5

24 November 2023

de Bell S, *et al. BMJ Open* 2026; 16:e107772. doi: 10.1136/bmjopen-2025-107772

**Produced by**	Exeter HS&DR Evidence Synthesis Centre<br>Department of Health & Community Sciences<br>South Cloisters<br>St Luke's Campus<br>Heavitree Road<br>Exeter<br>EX1 2LU<br>UK
**Authors**	**Zhivko Zhelev** Systematic Reviewer [1]<br>z.zhelev@exeter.ac.uk<br>**Sian de Bell** Systematic Reviewer [1]<br>s.c.de-bell@exeter.ac.uk<br>**Alison Bethel** Information Specialist[1]<br>a.bethel@exeter.ac.uk<br>**Maria Clarke** Information Specialist[1]<br>m.e.clarke2@exeter.ac.uk<br>**Rob Anderson** Senior/Academic Lead[1]<br>r.anderson@exeter.ac.uk<br>**Jo Thompson Coon** Senior/Academic Lead[1]<br>j.thompson-coon@exeter.ac.uk<br><br>[1] University of Exeter
**Correspondence to**	z.zhelev@exeter.ac.uk

**Date completed**	24 November 2023
**Source of funding**	This work was commissioned by the NIHR HSDR programme as a project (project code NIHR136105) within grant number NIHR130538.
**Role of funder/institution in protocol creation**	The aims and scope of this protocol and the planned work were informed by information provided by and discussions with staff in the NHS Patient Safety Team and Department of Health & Social Care

**Declared competing interests of the authors**	None
**Rider on responsibility for document**	The views and opinions expressed by authors in this publication are those of the authors and do not necessarily reflect those of the NHS, the NIHR, NETSCC, the HSDR programme or the Department of Health & Social Care. Any errors are the responsibility of the authors.
**PROSPERO registration number**	CRD42023487512

de Bell S, *et al. BMJ Open* 2026; 16:e107772. doi: 10.1136/bmjopen-2025-107772

Table of Contents

1 Background 5
1.1 Development of the review 6
1.2 Research questions 7
2 Methods 8
2.1 Approach to evidence synthesis 8
2.2 Identification of studies 9
2.2.1 Searches 9
2.2.2 Inclusion and exclusion criteria 9
2.2.3 Process for applying inclusion criteria 11
2.3 Data extraction 11
2.4 Study quality assessment strategy 12
2.5 Data analysis and presentation 12
3 Stakeholder and patient/public involvement 14
4 Dissemination plans 15
5 References 16

de Bell S, *et al. BMJ Open* 2026; 16:e107772. doi: 10.1136/bmjopen-2025-107772

1 Background

There are a number of industries, from aviation to nuclear power, in which safety is a priority. Within these industries, failures or errors could have serious consequences for people, causing loss of life or injury, and for the environment, in terms of environmental damage or harm to equipment or property.(1) Healthcare is now considered to be one of these 'safety-critical industries', with safety in this setting defined as:

> "The avoidance, prevention and amelioration of adverse outcomes or injuries stemming from the process of healthcare."(2)

Examples of safety incidents in healthcare include medication errors, wrong site surgery, and lack of a timely response to deterioration in a patient's condition.

High-risk industries have improved safety by moving from measuring and responding to specific incidents, to assessing the presence of, and managing, the conditions that lead to hazards.(3) This requires a systems thinking approach, considering interactions and intersections between social and technological elements, which are where safety events tend to occur.(3, 4) Change in some areas may be slow, such as to structural factors e.g. physical infrastructure or the organisation of departments, whereas in other areas of the system, particularly those dependent on more transient 'mediating' factors such as morale, teamwork, and individual performance, change may be affected more rapidly.(3)

Within many safety-critical industries, a systems approach to safety has been operationalised in the form of safety management systems (SMS): "an organised approach to managing safety, including the necessary organisational structures, accountabilities, policies and procedures".(5) These are generally considered to have four key components:

- Policy and objectives,
- Risk management,
- Safety assurance,
- Safety promotion (safety culture).(5)

These are sometimes expanded into six areas, offering further detail on considerations and requirements within the four key components identified above: (i) a safety policy; (ii) organisational arrangements to support safety e.g. supervision and training of staff in implementing the safety policy and processes; (iii) a safety plan, detailing standards and processes for safety, including risk assessment; (iv) measurement of safety performance; (v)

reviews of safety performance e.g. incident investigation, safety audits; and (vi) feedback loops to improve safety performance.(3)

A systematic review of SMS in three safety-critical industries - aviation, marine and rail – published in 2012 indicated that they led to improvements in safety e.g. reduced accident rates.(6) There is some debate over whether indicators such as accident rates are an appropriate way of measuring the effectiveness of SMS,(7) but this does suggest that they have the potential to improve patient safety in organisations providing healthcare. However, whilst the basic principles of SMS are widely accepted as transferrable,(4, 7) evidence from other industries suggests that the details (e.g. of accountability frameworks) need to be context-specific to be effective.(8) In healthcare, this might involve identifying and improving the management of known patient safety risks.(9) Components of SMS, such as national strategies for improving patient safety,(4) exist in most healthcare systems, and some countries, such as Australia (10) and the Netherlands,(11) are explicitly developing and promoting safety management systems. Their experiences offer opportunities for learning that could support the implementation of the principles of SMS in other healthcare systems, which will have different starting points in terms of the organisation and management of healthcare, and different pre-existing system components.

1.1 Development of the review

This review of research and other evidence has been commissioned by the NIHR HSDR programme to provide evidence and information to NHS England and the NHS Patient Safety Team. It is primarily intended to inform the further development of the NHS England's patient safety policy and practice as laid out in the NHS Patient Safety Strategy 2019(12) and related documents.

The policy questions of interest detailed in the project brief were:

Q1: What are the key attributes of a successful SMS for the NHS in England?

Q2: What are the links between a SMS and a Quality Management System?

Q3: What are the next steps for safety management in the NHS in England?

Evidence is emerging that could begin to answer these questions following the implementation of NHS Patient Safety Strategy 2019.(12) For example, the work of the National Patient Safety team is saving an estimated 160 lives per year (13), and evaluation of the Patient Safety Incident Response Framework (PSIRF) early adopter programme indicates it has led to improved safety cultures and identification of more effective risk reduction strategies in participating organisations.(13) A recent report published by the

Healthcare Safety Investigation Branch (HSIB)(14) has used evidence on the implementation of SMS in a range of high-risk industries, from aviation to the oil and gas industry, to describe key attributes of SMS at a broad level and make recommendations for the further development of a SMS in the NHS. However, more detailed evidence is needed to assess how a SMS approach might be applicable to the NHS in England.

The review described in this protocol aims to provide information on SMS in healthcare specifically, by investigating how they have been implemented in other countries. This will offer context to inform primary research into the questions raised above, and consideration of how NHS England's current patient safety policy, processes and practice could be extended into a full SMS.

1.2 Research questions

In high-income countries applying the SMS approach (as specified below), what principles have been used to inform healthcare policy, management and practice related to patient and staff safety and what is the evidence supporting their adoption and implementation?

Within this broader question, our specific questions are:

How are the components of SMS reflected in the healthcare policy documents of the above countries, or their regional healthcare systems?

What research (e.g. formal evaluations, or based on routine data) or other relevant evidence (e.g. staff and patient narratives) is available regarding the effectiveness, implementation or experience of SMS within healthcare?

What does existing research and other relevant evidence from beyond the UK tell us about the effectiveness, implementation or experience of SMS within healthcare?

de Bell S, *et al. BMJ Open* 2026; 16:e107772. doi: 10.1136/bmjopen-2025-107772

2 Methods

2.1 Approach to evidence synthesis

As noted above, there is a paucity of empirical evidence on the use of SMS in healthcare. Also, the specific application of such systems in healthcare is likely to depend on the local context, reflecting pre-existing arrangements as well as current priorities and initiatives. To better understand the context of each application and given the expectation of limited empirical evidence, we will conduct a systematic review with a broader scope, including, in addition to research evidence, an analysis of policy documents, training materials and other relevant information (e.g. patients and healthcare professionals' narratives). This will allow better understanding of the logic and process of each implementation; more adequate interpretation of the available empirical evidence; better understanding of the similarities and differences across systems; and the identification of specific barriers, facilitators and challenges that need to be addressed in future research. The review will follow the process detailed below.

Firstly, we will take the four pillars of a SMS as defined by HSIB (14), and compare them to other key SMS literature e.g. documents from safety-critical industries such as aviation (5). In consultation with stakeholders, we will produce an expanded and more detailed framework of the components of a SMS. This will be used to inform data extraction and analysis, to allow us to identify the aspects of SMS that have and have not been adopted by different healthcare systems.

Secondly, we will identify national or regional healthcare systems in which SMS principles have been used to inform policy and practice related to patient and staff safety. For pragmatic reasons, we will focus first on high-income English-speaking countries and, if necessary and feasible, include other countries after discussion with stakeholders. If key policy documents do not state explicitly that a SMS approach was used, then we will contact relevant organisations to enquire about this. Only healthcare systems where such a link and commitment are explicitly stated will be included in the review.

Thirdly, we will identify relevant policy documents, research and other relevant information (e.g. patient stories) from the organisations' websites and by focused searches of electronic databases and other sources. Each organisation will be contacted to ascertain that all relevant information has been captured and request additional data that may have been missed or is not available online.

Fourthly, we will develop a data extraction form organised around the core principles of SMS (as defined in the background) and the different types of evidence we are including: policy

documents, research and other evidence. If possible, data will be extracted verbatim or, if too long, summarised with reference to its location in the source document. Rather than just depicting a snapshot of the *status quo*, as much as possible, the information gathered will attempt to distinguish the sequence and timings of the adoption and application of different core principles of SMS, or of the main strategies and processes to implement and embed them in a particular country or region/province. This therefore aims to shed light on which principles and systems/processes may have pre-existed the formal policy commitment to adopting a SMS approach, which were seen as most critical for expanding the scope and depth of the SMS, and interactions between the different components of SMS.

Finally, information on the implementation of SMS in the eligible healthcare systems will be synthesised narratively and summarised in tables and graphs. Common themes across different healthcare systems will be identified, evidence gaps will be highlighted, and recommendations on how to address those will be made.

2.2 Identification of studies

2.2.1 Searches

The main method for identifying relevant policy documents, research and other evidence will be through searching the websites of, and direct contact with, relevant organisations (e.g. Australian Commission on Safety and Quality in Healthcare). Website searching will be carried out either by browsing publication lists or using site search functionality. Search parameters (e.g. keywords, publication date) will be pre-specified and the search process will be documented (e.g. number of hits, number of potentially relevant titles) to allow transparency and reproducibility.

The above will be complemented by focused database searches using search strategies developed by an information specialist (AB) in consultation with the review team and the commissioning group. The database search strategies will use both controlled vocabulary when available and relevant (e.g. MeSH in MEDLINE) and free-text searching. Search terms will be partly derived from the literature and from stakeholders and experts' input. Searches will be limited to English-language texts published after a pre-specified date (to be agreed with stakeholders and experts). The reference lists of all relevant documents will be hand-searched for additional titles.

2.2.2 Inclusion and exclusion criteria

The following overarching inclusion and exclusion criteria will be applied:

de Bell S, *et al. BMJ Open* 2026; 16:e107772. doi: 10.1136/bmjopen-2025-107772

- **Safety management system:** Safety management systems are defined by the following key elements:
    - policy and objectives
    - risk management
    - safety assurance
    - safety promotion (safety culture).

  Texts will be excluded if they refer to different concepts or models without direct link to the above definition of a safety management system.

- **Application of SMS** in the healthcare system of a high-income country (as defined by the World Bank). For pragmatic reasons, we will focus on English-speaking countries; if necessary and feasible, and after discussion with our policy costumer, we may extend the scope to include other, selected high-income countries.

- **Type of evidence:** we will include three distinct types of evidence:
    - Relevant policy documents, including documents related to the implementation of SMS, such as staff training and patient-facing materials
    - Published or unpublished research / evaluations (quantitative, qualitative or mixed methods),
    - Other evidence, e.g. patient and staff experience or opinions published on the organisations' websites.

- **Provenance of evidence:** As mentioned earlier, for pragmatic reasons we will focus first on high-income English-speaking countries as evidence from such healthcare systems is more likely to be relevant to the UK and all relevant policy documents and research will be available in English. Therefore, evidence related to the implementation of SMS in the healthcare of the following countries will be considered first: Ireland, Australia, New Zealand, Canada and the Netherlands. The list will be revised / amended once the initial searches have been completed and the volume, type and quality of evidence is assessed; or, it has been decided in discussion with experts and stakeholders that including a specific country or expanding the list is desirable and feasible.

de Bell S, *et al. BMJ Open* 2026; 16:e107772. doi: 10.1136/bmjopen-2025-107772

- **Language:** we will include texts available in English; if the scope of the review is extended (see above) we may decide to include texts in other languages, if necessary and feasible.

2.2.3 Process for applying the inclusion criteria

A small sample (n ~ 10) of potentially relevant documents from the three types of evidence will be used to calibrate the selection process. Each document will be reviewed by multiple reviewers. Decisions will be compared and discussed in a group meeting to ensure consistent application of criteria. If necessary, definitions will be updated and explanatory notes added to aid the process.

The full text of all potentially relevant documents identified through the website searches and direct contact with the healthcare organisations will be retrieved and checked for relevance against the inclusion criteria independently by two reviewers.

The results from the database searches will be screened following the standard process. Two reviewers will independently apply the inclusion and exclusion criteria to the title and abstract of each identified citation. We will obtain the full text of papers where either reviewer judges it to be a potential inclusion. Two reviewers will assess the full text of each record independently for inclusion, with disagreements settled through discussion or by a third reviewer. The full texts of all potentially relevant publications identified from the website searches will be obtained and screened as per the above process.

The study selection process will be detailed using a PRISMA-style flowchart, with a reason reported for exclusion of each record assessed at full text.

2.3 Data extraction

A data extraction form will be developed and piloted independently by two reviewers on a small sample of included documents. Data will be extracted by one reviewer and checked by a second reviewer. Disagreements will be resolved through discussion.

The following data will be extracted:

- Country
- Healthcare organisation (purpose and remit, website)
- Type of document (policy, research, anecdotal evidence)
- Aims

- Relation to SMS (e.g. explicitly stated or implied, description)
- Information regarding specific SMS components:
    - Policy and objectives
    - Risk management
    - Safety assurance
    - Safety promotion (safety culture).
- Information regarding implementation and/or impact
- **Research:** research design, aim, methods, results, conclusions
- **Other evidence:** type of document, informant (patient, healthcare professional, policy maker), perspective (relationship to the programme), main points.

The NIHR-INCLUDE guidelines (15) were used to reflect on Equality, Diversity and Inclusion (EDI) whilst designing the protocol. EDI is important to consider in relation to the topic of safety management systems, as there may be differences in safety incidents between social and demographic groups and resulting inequities in health outcomes. However, it is likely this information will be difficult to ascertain from the types of evidence e.g. policy documents, we will be including in the review. We will consider PROGRESS-Plus characteristics during the process of data extraction and analysis (16, 17) and, where possible, use this information to inform the findings and recommendations of the review.

2.4 Study quality assessment strategy

The methodological quality of all research studies will be assessed using validated quality assessment tools according to the specific research design. Quality will be assessed by two reviewers independently and disagreements resolved through discussion or arbitration. No quality appraisal will be carried out on policy documents or non-research evidence.

2.5 Data analysis and presentation

The type of information identified for each healthcare system will be summarised and the following information will be reported:

- The way in which the SMS approach has informed patient and staff safety policy and practice.
- Information related to each of the SMS components (see above).

de Bell S, et al. BMJ Open 2026; 16:e107772. doi: 10.1136/bmjopen-2025-107772

- Information related to the process and timing of implementing SMS (e.g. steps, processes etc.).
- Evidence related to the effectiveness / impact and implementation of SMS.
- Stakeholders' perceptions and experience.

Comparisons across healthcare systems will be carried out to identify similarities and differences, including barriers, facilitators and challenges; the volume, type and quality of the available evidence and any evidence gaps that need to be addressed in future research. The results will be summarised in tables and diagrams and presented narratively, considering their relevance to the application of SMS in the NHS of England.

3 Stakeholder and patient/public involvement and engagement

The following stakeholders will be involved:

- NHS England's Patient Safety Team and the Department of Health and Social Care (as commissioners of the work)
- Topic experts (including from other countries and other industries, if possible)
- Any other relevant Patient Safety organisations (NHS or external) e.g. local patient safety teams

Due to the focus of the review, we will consult Public and Patient Involvement and Engagement representatives who have some knowledge of the topic e.g. members of local patient safety teams, or PPIE representatives who have worked with the National Patient Safety Team.

We will consult stakeholders throughout the review process, to discuss topics such as the focus of the review, analysis, and findings. Meetings will be arranged to suit the project progress and stakeholder availability. We will also ask for their input on dissemination plans and materials as described below.

4 Dissemination plans

We will produce a report on the implementation of safety management systems in healthcare in high-income countries, available as an open access in the NIHR Journals Library.

Further materials for dissemination will be finalised after discussion with the project's advisory group. These are likely to include:

- an evidence briefing, giving a plain language summary of the report and its findings (primarily aimed at policy makers and health service managers);
- an article in an academic journal identified as being relevant to stakeholders for this review; and
- presentations at key national and regional meetings.

Outputs will be disseminated via the Exeter HSDR Evidence Synthesis Centre webpage and social media. Additional material may be produced to promote them, such as a blog post based on the evidence briefing and report.

de Bell S, *et al. BMJ Open* 2026; 16:e107772. doi: 10.1136/bmjopen-2025-107772

5 References

1. Saunders F. Safety-critical industries: definitions, tensions and tradeoffs 2015 [Available from: http://fionasaunders.co.uk/safety-critical-industries-definitions-tensions-and-tradeoffs/#:~:text=Combining%20these%20two%20definitions%2C%20a%20safety-critical%20industry%20can,environmental%20damage%2C%20or%20harm%20to%20plant%20or%20property.

2. Vincent C. Patient safety. 2nd edition ed. Chichester: John Wiley and Sons; 2010.

3. Vincent C, Burnett S, Carthey J. The measurement and monitoring of safety. London: The Health Foundation; 2013.

4. Macchi L, Pietikäinen E, Reiman T, Heikkilä J, Ruuhilehto K. Patient safety management: Available models and systems. VTT; 2011.

5. International Civil Aviation Organisation Safety Management Manual (ICAOSMM). 2nd ed. Doc 9859, Safety Management Manual (SMM) 2009 [Available from: www2.icao.int/en/ism/Guidance%20Materials/DOC_9859_FULL_EN.pdf.

6. Thomas M. A systematic review of the effectiveness of safety management systems. ATSB Transport Safety Report, Cross-modal Research Investigation, XR-2011-0. Canberra: Australian Transport Safety Bureau; 2012.

7. Li Y, Guldenmund FW. Safety management systems: A broad overview of the literature. Safety Science. 2018;103:94-123.

8. Kapur N, Parand A, Soukup T, Reader T, Sevdalis N. Aviation and healthcare: a comparative review with implications for patient safety. JRSM Open. 2016;7.

9. HSIB. A thematic analysis of HSIB's first 22 national investigations. Healthcare Safety Investigation Branch I2020/016; 2021.

10. Australian Commission on Safety and Quality in Health Care. The state of patient safety and quality in Australian hospitals 2019. Sydney: Australian Commission on Safety and Quality in Health Care; 2019.

11. Bolk M. Final report: Embedding patient safety in education & training. VMS Safety Program; 2013.

12. NHS England. The NHS Patient Safety Strategy no date [Available from: https://www.england.nhs.uk/patient-safety/the-nhs-patient-safety-strategy/#key-docs.

13. NHS England. NHS patient safety strategy – progress so far no date [Available from: https://www.england.nhs.uk/patient-safety/the-nhs-patient-safety-strategy/nhs-patient-safety-strategy-progress-so-far/.

14. HSIB. Safety management systems - an introduction for healthcare. 2023.

15. National Institute for Health Research. Improving inclusion of under-served groups in clinical research: Guidance from the NIHR-INCLUDE project. UK: National Institute for Health Research; 2020.

16. O'Neill J, Tabish H, Welch V, Petticrew M, Pottie K, Clarke M, et al. Applying an equity lens to interventions: using PROGRESS ensures consideration of socially stratifying factors to illuminate inequities in health. J Clin Epidemiol. 2014;67:56-64.

17. Cochrane Methods Equity. PROGRESS-Plus no date [Available from: https://methods.cochrane.org/equity/projects/evidence-equity/progress-plus.

de Bell S, *et al. BMJ Open* 2026; 16:e107772. doi: 10.1136/bmjopen-2025-107772

Supplementary material 2: Inclusion criteria

	Include	Exclude
**Population**	Populations with any health need.	-
**Initiative**	Any use of an SMS approach in a healthcare setting, with an SMS being defined by four key components:<br>• policy and objectives<br>• risk management<br>• safety assurance<br>• safety promotion (safety culture).<br><br>Where it is not clear whether a country is using an SMS approach, we will include studies if they relate to one or more components of an SMS.	Studies of safety where this is not linked to an SMS component.<br>Studies of occupational health and safety.
**Setting**	Any healthcare setting:<br>- Secondary care,<br>- Primary care.	Non-healthcare settings:<br>- Care homes,<br>- Home care.
**Country**	Australia, Canada, Ireland, the Netherlands, New Zealand.<br><br>Studies which included other countries were included if the results for these countries were reported separately.	Any other country.
**Language**	English or Dutch.	Any other language.

	Include	Exclude
**Type of document**	• Relevant policy documents, including documents related to the implementation of SMS, such as staff training and patient-facing materials<br>• Published or unpublished research / evaluations (quantitative, qualitative or mixed methods),<br>• Other evidence (e.g. patient and staff experience or opinions published on the organisations' websites).	Theoretical

de Bell S, *et al. BMJ Open* 2026; 16:e107772. doi: 10.1136/bmjopen-2025-107772

Supplementary material 3: Excluded and thin studies

Excluded papers from database searching (n=68)

Excluded on country (n=7)

Ma E, Wei W, Ho C. An environmental scan of patient safety reporting and learning systems in community healthcare for multi- disciplinary teams. *Can J Hosp Pharm*. 2022;**75**(2):135.

Coffey M, Marino M, Lyren A, Purcell D, Hoffman JM, Brilli R, et al. Association between hospital-acquired harm outcomes and membership in a national patient safety collaborative. *JAMA Pediatrics*. 2022;**176**(9):924-32.

Kristensen S, Hammer A, Bartels P, Sunol R, Groene O, Thompson CA, et al. Quality management and perceptions of teamwork and safety climate in European hospitals. *Int J Qual Health Care*. 2015;**27**(6):499-506.

Holmstrom AR, Laaksonen R, Airaksinen M. How to make medication error reporting systems work - Factors associated with their successful development and implementation. *Health Policy*. 2015;**119**(8):1046-54.

Windsor JA, Maxim S. Patient safety in medicine: Are surgeons ready for checklists? *ANZ Journal of Surgery*. 2010;**80**(1-2):3-5.

Sunol R, Vallejo P, Groene O, Escaramis G, Thompson A, Kutryba B, et al. Implementation of patient safety strategies in European hospitals. *Qual Saf Health Care. 2009*;**18**(Suppl 1):i57-61.

Shaw C, Kutryba B, Crisp H, Vallejo P, Sunol R. Do European hospitals have quality and safety governance systems and structures in place? *Qual Saf Health Care*. 2009;**18**(Suppl 1):i51-6.

Excluded on focus (n=54)

Ong N, Lucien A, Long JC, Weise J, Walton M, Burgess A. What do healthcare staff think about the quality and safety of care provided to children and young people with an intellectual disability? A qualitative study using the framework method of analysis. *BMJ Open*. 2023;**13**(7):e071494.

Kaud Y, McKeon D, Lydon S, O'Connor P. Measuring and monitoring patient safety in hospitals in the Republic of Ireland. *Irish J Med Sci*. 2023;1**92:**2581–259.

Cords CI, van Baar ME, Nieuwenhuis MK, Pijpe A, van der Vlies CH. Reliability and validity of a frailty assessment tool in specialized burn care, a retrospective multicentre cohort study. *Burns*. 2023;**49**(7):1621-31.

Terry D, Kim J-a, Gilbert J, Jang S, Nguyen H. "Thank You for Listening": An Exploratory Study Regarding the Lived Experience and Perception of Medical Errors Among Those Who Receive Care. *International Journal of Health Services*. 2022;**52**(2):292-302.

Arnold A, Ward I, Gandhidasan S. Incident review in radiation oncology. *J Med Imaging Radiat Oncol*. 2022;**66**(2):291-8.

Malik RF, Buljac-Samardzic M, Amajjar I, Hilders C, Scheele F. Open organisational culture: what does it entail? Healthcare stakeholders reaching consensus by means of a Delphi technique. *BMJ Open*. 2021;**11**(9):e045515.

Connell CJ, Cooper S, Endacott R. Measuring the safety climate in an Australian emergency department. *Int Emerg Nurs*. 2021;**58**:101048.

Adie K, Fois RA, McLachlan AJ, Walpola RL, Chen TF. The nature, severity and causes of medication incidents from an Australian community pharmacy incident reporting system: The QUMwatch study. *Br J Clin Pharmacol*. 2021;**87**(12):4809-22.

Wright KM, Bonser M. The Essential Steps of Medication Administration Practices Project medication administration improvement practices among acute inpatients in a tertiary hospital: a best practice implementation project. *International Journal of Evidence-Based Healthcare*. 2020;**18**(4):408-19.

Floyd E, Hodgkins R, Naylon L, Noon M, Sirotti L, Monaro S. The costs and benefits of pressure injury point prevalence auditing. *Wound Practice & Research*. 2020;**28**(2):66-74.

Emond Y, Wolff AP, Peters YAS, Bloo GJA, Westert GP, Damen J, et al. Reducing work pressure and IT problems and facilitating IT integration and audit & feedback help adherence to perioperative safety guidelines: a survey among 95 perioperative professionals. *Implement*. 2020;**1**:49.

Clay-Williams R, Taylor N, Winata T, Ting HP, Arnolda G, Braithwaite J. Organization quality systems and department-level strategies: refinement of the Deepening our Understanding in Quality in Australia (DUQuA) organization and department-level scales. *Int J Qual Health Care*. 2020;**32**(Supplement_1):22-34.

Braithwaite J, Taylor N, Clay-Williams R, Ting HP, Arnolda G. Conclusion: the road ahead: where should we go now to improve healthcare quality in acute settings? *Int J Qual Health Care*. 2020;**32**(Supplement_1):99-103.

Ward M, Ni She E, De Brun A, Korpos C, Hamza M, Burke E, et al. The co-design, implementation and evaluation of a serious board game 'PlayDecide patient safety' to educate junior doctors about patient safety and the importance of reporting safety concerns. *BMC Med Educ*. 2019;**19**(1):232.

McHugh S, Droog E, Foley C, Boyce M, Healy O, Browne JP. Understanding the impetus for major systems change: A multiple case study of decisions and non-decisions to reconfigure emergency and urgent care services. *Health Policy*. 2019;**123**(8):728-36.

Liao E, Eisenberg N, Kaushal A, Montbriand J, Tan KT, Roche-Nagle G. Utility of the Vascular Quality Initiative in improving quality of care in Canadian patients undergoing vascular surgery. *Can J Surg*. 2019;**62**(1):66-9.

Eljiz K, Greenfield D, Derrett A, Radmore S. Health system redesign: Changing thoughts, values, and behaviours for the co-production of a safety culture. *Int J Health Plann Manage*. 2019;**34**(4):1477-84.

Veitch D. One province, one healthcare system: A decade of healthcare transformation in Alberta. *Healthc Manage Forum*. 2018;**31**(5):167-71.

Sinclair JE, Austin MA, Bourque C, Kortko J, Maloney J, Dionne R, et al. Barriers to self-reporting patient safety incidents by paramedics: A mixed methods study. *Prehospital Emergency Care*. 2018;**22**(6):762-72.

Khalil H, Lee S. Medication safety challenges in primary care: Nurses' perspective. *Journal of Clinical Nursing*. 2018;**27**(9-10):2072-82.

Debono D, Taylor N, Lipworth W, Greenfield D, Travaglia J, Black D, et al. Applying the Theoretical Domains Framework to identify barriers and targeted interventions to enhance nurses' use of electronic medication management systems in two Australian hospitals. *Implement Sci*. 2017;**12**(1):42.

Davies JM, Delaney G. Can the Aviation Industry be Useful in Teaching Oncology about Safety? *Clin Oncol*. 2017;**29**(10):669-75.

Blijleven V, Koelemeijer K, Wetzels M, Jaspers M. Workarounds emerging from electronic health record system usage: Consequences for patient safety, effectiveness of care, and efficiency of care. *JMIR Hum Factors*. 2017;**4**(4):e27.

Wagner C, Merten H, Zwaan L, Lubberding S, Timmermans D, Smits M. Unit-based incident reporting and root cause analysis: variation at three hospital unit types. *BMJ Open*. 2016;**6**(6):e011277.

Ng J, Andrew P, Crawley M, Pevreal W, Peach J. Assessing a hospital medication system for patient safety: findings and lessons learnt from trialling an Australian modified tool at Waitemata District Health Board. *N Z Med J*. 2016;**129**(1430):63-77.

Gill FJ, Leslie GD, Marshall AP. Family initiated escalation of care for the deteriorating patient in hospital: Family centred care or just "box ticking". *Aust Crit Care*. 2016;**29**(4):195-200.

Yates M, Reddy M, Machumpurath B, Phelps G, Hampson SA. Modification of the National Inpatient Medication Chart improves venous thromboembolism prophylaxis rates in high-risk medical patients. *Intern Med J*. 2014;**44**(2):190-4.

Wong KS, Ryan DP, Liu BA. A system-wide analysis using a senior-friendly hospital framework identifies current practices and opportunities for improvement in the care of hospitalized older adults. *J Am Geriatr Soc*. 2014;**62**(11):2163-70.

Teare GF. Measurement of quality and safety in healthcare: the past decade and the next. *Healthc Q*. 2014;**17 Spec No**:45-50.

Russell L, Dawda P. Lessons for the Australian healthcare system from the Berwick report. *Aust Health Rev*. 2014;**38**(1):106-8.

Berg M, Black G. A Canadian perspective on clinical governance. *Clinical Governance: An International Journal*. 2014;**19**(4):314-21.

van Beuzekom M, Boer F, Akerboom S, Dahan A. Perception of patient safety differs by clinical area and discipline. *Br J Anaesth*. 2013;**110**(1):107-14.

Levi M. Towards a hospital-wide integrated system for quality and safety of health care. Neth J Med. 2013;**71**(1):2-3.

Marsden J, van Dijk M, Doris P, Krause C, Cochrane D. Improving care for British Columbians: the critical role of physician engagement. *Healthc Q*. 2012;**15 Spec No**:51-5.

Krause C, Cochrane D. BC Patient Safety & Quality Council: using network and social movement theory to improve healthcare. *Healthc Manage Forum*. 2012;**25**(4):181-4.

Ilan R, Squires M, Panopoulos C, Day A. Increasing patient safety event reporting in 2 intensive care units: a prospective interventional study. *J Crit Care*. 2011;**26**(4):431.e11-8.

Green E, White R, Janes K, Fields A, Easty A. Courage, collaboration, complexity and chemotherapy safety: the view from the sharp end. *Canadian Oncology Nursing Journal*. 2011;**21**(2):81-90.

Duckers ML, Wagner C, Vos L, Groenewegen PP. Understanding organisational development, sustainability, and diffusion of innovations within hospitals participating in a multilevel quality collaborative. *Implement Sci*. 2011;**6**:18.

van Gaal BG, Schoonhoven L, Hulscher ME, Mintjes JA, Borm GF, Koopmans RT, et al. The design of the SAFE or SORRY? study: a cluster randomised trial on the development and testing of an evidence based inpatient safety program for the prevention of adverse events. *BMC Health Serv Res*. 2009;**9**:58.

Relihan E, Silke B, O'Grady F. Internally-developed electronic reporting system for medication errors. *Ir Med J*. 2009;**102**(7):223-4.

Michel J, Nghiem HD, Jackson TJ. Using ICD-10-AM codes to characterise hospital-acquired complications. *Health Inf Manag*. 2009;**38**(3):18-25.

Neudorf K, Dyck N, Scott D, Davidson Dick D. Nursing education: a catalyst for the patient safety movement. *Healthc Q*. 2008;**11**(3 Spec No.):35-9.

Makeham MA, Saltman DC, Kidd MR. Lessons from the TAPS study--recall and reminder systems. *Aust Fam Physician*. 2008;**37**(11):923-4.

Makeham MA, Cooper C, Kidd MR. Lessons from the TAPS study - message handling and appointment systems. *Aust Fam Physician*. 2008;**37**(6):438-9.

Duckett S, Daniels S, Kamp M, Stockwell A, Walker G, Ward M. Pay for performance in Australia: Queensland's new Clinical Practice Improvement Payment. *Journal of Health Services Research and Policy*. 2008;**13**(3):174-7.

Booth BJ, Snowdon T, Harris MF, Tomlins R. Safety and quality in primary care: The view from general practice. *Australian Journal of Primary Health*. 2008;**14**(2):19-27.

David U. Medication safety... Your call to make a difference. *Alta RN*. 2007;**63**(8):10-1.

Runciman WB, Williamson JA, Deakin A, Benveniste KA, Bannon K, Hibbert PD. An integrated framework for safety, quality and risk management: an information and incident management system based on a universal patient safety classification. *Qual Saf Health Care*. 2006;**15**(Suppl 1):i82-90.

Greenberg A, Kramer S, Welch V, O'Sullivan E, Hall S. Cancer Care Ontario's computerized physician order entry system: a province-wide patient safety innovation. *Healthc Q*. 2006;**9 Spec No**:108-13.

Yassi A, Hancock T. Patient safety--worker safety: building a culture of safety to improve healthcare worker and patient well-being. *Healthc Q*. 2005;**8 Spec No**:32-8.

Greenall J, U D, Lam R. An effective tool to enhance a culture of patient safety and assess the risks of medication use systems. *Healthc Q*. 2005;**8 Spec No**:53-8.

Court D. Quest for patient safety in a challenging environment. *Aust N Z J Obstet Gynaecol*. 2003;**43**(2):97-100.

Nicklin W, McVeety JE. Canadian nurses' perceptions of patient safety in hospitals. *Can J Nurs Leadersh*. 2002;**15**(3):11-21.

de Bell S, *et al. BMJ Open* 2026; 16:e107772. doi: 10.1136/bmjopen-2025-107772

Baldwin I, Beckman U, Shaw L, Morrison A. Australian Incident Monitoring Study in intensive care: local unit review meetings and report management. *Anaesth Intensive Care*. 1998;**26**(3):294-7.

Excluded on language (n=1)

Broeke RT, Lammers R, Ackerman E. The safety management system for medication contributes to a 'safe culture' in the hospital: The pharmacist participates in the medical round at the nursing department. *Pharmaceutisch Weekblad*. 2008;**143**(17):30-2.

Excluded on publication type (n=2)

Hanskamp-Sebregts M, Zegers M, Boeijen W, Westert GP, van Gurp PJ, Wollersheim H. Effects of auditing patient safety in hospital care: design of a mixed-method evaluation. BMC Health Serv Res. 2013;13:226.

Barker A, Mengersen K, Morton A. What is the value of hospital mortality indicators, and are there ways to do better? Aust Health Rev. 2012;36(4):374-7.

Excluded on setting (n=2)

Masotti P, Green M, McColl MA. Adverse events in community care: implications for practice, policy and research. *Healthc Q*. 2009;**12**(1):69-76.

Lang A, Edwards N, Fleiszer A. Safety in home care: a broadened perspective of patient safety. *Int J Qual Health Care*. 2008;**20**(2):130-5.

Update search

Excluded on focus (n=2)

Lam JYJ, Barras M, Scott IA, Abdel-Hafez A, Snoswell C, Gordon E, Morris C, Long D, Wang A, Falconer N. Impact evaluation of the modified adverse inpatient medication event (AIME-Frail) model in hospitalised adults. *Res Soc Ad Pharm*. 2025;**21**:687-696.

McLennan C, Sherrington C, Naganathan V, Tilden W, Richards B, McVeigh T, Hallahan A, Nayak V, Jennings M, Hassett L, Haynes A. Supported implementation of tailored multicomponent fall prevention interventions in hospital: a feasibility study. *BMJ Open Qual.* 2025:**14:**e003313.

List of 'thin' studies

Australia

Abou Elnour A, Hernan AL, Ford D, Clark S, Fuller J, Johnson JK, et al. Surveyors' perceptions of the impact of accreditation on patient safety in general practice. *Med J Aust*. 2014;**201**(3):S56-S9. https://dx.doi.org/10.5694/mja14.00198

Adamson L, Beldham-Collins R, Sykes J, Thwaites D. Evaluating incident learning systems and safety culture in two radiation oncology departments. *J Med Radiat Sci*. 2022;**69**(2):208-17. https://dx.doi.org/10.1002/jmrs.563

Adie K, Fois RA, McLachlan AJ, Chen TF. Medication incident recovery and prevention utilising an Australian community pharmacy incident reporting system: the QUMwatch study. *Eur J Clin Pharmacol*. 2021;**77**(9):1381-95. https://dx.doi.org/10.1007/s00228-020-03075-9

Avery MJ, Cripps AW, Rogers GD. Health boards' governance of quality and risk: quality improvement agenda for the board. *Int J Health Gov*. 2021;**26**(3):292-306. https://dx.doi.org/10.1108/IJHG-01-2021-0006

Braithwaite J, Westbrook MT, Mallock NA, Travaglia JF, Iedema RA. Experiences of health professionals who conducted root cause analyses after undergoing a safety improvement programme. *Qual Saf Health Care*. 2006;**15**(6):393-9. https://dx.doi.org/10.1136/qshc.2005.017525

Braithwaite J, Westbrook MT, Travaglia JF, Iedema R, Mallock NA, Long D, et al. Are health systems changing in support of patient safety? A multi-methods evaluation of education, attitudes and practice. *Int J Health Care Qual Assur*. 2007;**20**(7):585-601. https://dx.doi.org/10.1108/09526860710822725

Braithwaite J, Westbrook M, Travaglia J. Attitudes toward the large-scale implementation of an incident reporting system. *Int J Qual Health Care*. 2008;**20**(3):184-91. https://dx.doi.org/10.1093/intqhc/mzn004

Brand C, Ibrahim J, Bain C, Jones C, King B. Engineering a safe landing: engaging medical practitioners in a systems approach to patient safety. *Intern Med J*. 2007;**37**(5):295-302. https://dx.doi.org/10.1111/j.1445-5994.2007.01310.x

Canaway R, Bismark M, Dunt D, Kelaher M. Medical directors' perspectives on strengthening hospital quality and safety. *J Health Organ Manag*. 2017;**31**(7-8):696-712. https://dx.doi.org/10.1108/JHOM-05-2017-0109

Canaway R, Bismark M, Dunt D, Kelaher M. Public reporting of hospital performance data: views of senior medical directors in Victoria, Australia. *Aust Health Rev*. 2018;**42**(5):591-9. https://dx.doi.org/10.1071/AH17120

Clay-Williams R, Taylor N, Ting HP, Arnolda G, Winata T, Braithwaite J. Do quality management systems influence clinical safety culture and leadership? A study in 32 Australian hospitals. *Int J Qual Health Care*. 2020;**32**(Supplement_1):60-6. https://dx.doi.org/10.1093/intqhc/mzz107

Clay-Williams R, Taylor N, Ting HP, Winata T, Arnolda G, Austin E, et al. The relationships between quality management systems, safety culture and leadership and patient outcomes in Australian Emergency Departments. *Int J Qual Health Care*. 2020;**32**(Supplement_1):43-51. https://dx.doi.org/10.1093/intqhc/mzz105

Graudins LV, Dooley MJ. Medication safety: Experiential learning for pharmacy students and staff in a hospital setting. *Pharmacy*. 2016;**4**(4):17. https://dx.doi.org/10.3390/pharmacy4040038

Greenfield D, Hinchcliff R, Banks M, Mumford V, Hogden A, Debono D, et al. Analysing 'big picture' policy reform mechanisms: the Australian health service safety and quality accreditation scheme. *Health Expect*. 2015;**18**(6):3110-22. https://dx.doi.org/10.1111/hex.12300

Hanley E, Quoyle C. Approaches to surveillance of staphylococcus aureus bacteraemia and clostridium difficile infection in Australian states and territories. *Healthc Infect*. 2014;**19**(4):141-6. https://dx.doi.org/10.1071/HI14019

Hughes CF, Mackay P. Sea change: Public reporting and the safety and quality of the Australian health care system. *Med J Aust*. 2006;**184**(10S):S44-S7. https://dx.doi.org/10.5694/j.1326-5377.2006.tb00361.x

Johnson A, Clay-Williams R, Lane P. Framework for better care: reconciling approaches to patient safety and quality. *Aust Health Rev*. 2019;**43**(6):653-5. https://dx.doi.org/10.1071/AH18050

Jones D. Improving clinical leadership in patient safety: from strategic principle to active participation -- the Western Australian approach. *Int J Clin Leadersh*. 2008;**16**(3):157-62.

Leggat SG, Balding C. Effective quality systems: implementation in Australian public hospitals. *Int J Health Care Qual Assur*. 2018;**31**(8):1044-57. https://dx.doi.org/10.1108/IJHCQA-02-2017-0037

Lucas J, Leggat SG, Taylor NF. Association between use of clinical governance systems at the frontline and patient safety: a pre-post study. *Int J Health Gov*. 2022;**27**(3):282-95. https://dx.doi.org/10.1108/IJHG-02-2022-0023

Ong N, Lucien A, Long J, Weise J, Burgess A, Walton M. What do parents think about the quality and safety of care provided by hospitals to children and young people with an intellectual disability? A qualitative study using thematic analysis. *Health Expect*. 2023;**27**(1):e13925. https://dx.doi.org/10.1111/hex.13925

Pain C, Green M, Duff C, Hyland D, Pantle A, Fitzpatrick K, et al. Between the flags: implementing a safety-net system at scale to recognise and manage deteriorating patients in the New South Wales Public Health System. *Int J Qual Health Care*. 2017;**29**(1):130-6. https://dx.doi.org/10.1093/intqhc/mzw132

Rubin GL, Leeder SR. Health care safety: what needs to be done? *Med J Aust*. 2005;**183**(10):529-31. https://dx.doi.org/10.5694/j.1326-5377.2005.tb07155.x

Runciman WB. Lessons from the Australian Patient Safety Foundation: setting up a national patient safety surveillance system--is this the right model? *Qual Saf Health Care*. 2002;**11**(3):246-51. https://dx.doi.org/10.1136/qhc.11.3.246

Sinni SV, Wallace EM, Cross WM. Perinatal staff perceptions of safety and quality in their service. *BMC Health Serv Res*. 2014;**14**:591. https://dx.doi.org/10.1186/s12913-014-0591-4

Spigelman AD, Swan J. Review of the Australian incident monitoring system. *ANZ J Surg*. 2005;**75**(8):657-61. https://dx.doi.org/10.1111/j.1445-2197.2005.03482.x

Spigelman AD, Rendalls S. Clinical governance in Australia. *Clin Gov*. 2015;**20**(2):56-73. https://doi.org/10.1108/CGIJ-03-2015-0008

Stark HE, Graudins LV, McGuire TM, Lee CYY, Duguid MJ. Implementing a sustainable medication reconciliation process in Australian hospitals: The World Health Organization High 5s project. *Res Social Adm Pharm*. 2020;**16**(3):290-8. https://doi.org/10.1016/j.sapharm.2019.05.011

Stride P, Seleem M, Nath N, Horne A, Kapitsalas C. Integration of patient safety systems in a suburban hospital. *Aust Health Rev*. 2012;**36**(4):359-62. https://doi.org/10.1071/AH11099

Taitz J, Genn K, Brooks V, Ross D, Ryan K, Shumack B, et al. System-wide learning from root cause analysis: a report from the New South Wales Root Cause Analysis Review Committee. *Qual Saf Health Care*. 2010;**19**(6):e63. https://doi.org/10.1136/qshc.2008.032144

Taylor KA, Durrheim DN, Merritt T, Massey P, Ferguson J, Ryan N, et al. Multidisciplinary analysis of invasive meningococcal disease as a framework for continuous quality and safety improvement in regional Australia. *BMJ Open Qual*. 2018;**7**(1):e000077. https://doi.org/10.1136/bmjoq-2017-000077

Travaglia JF, Westbrook MT, Braithwaite J. Implementation of a patient safety incident management system as viewed by doctors, nurses and allied health professionals. *Health*. 2009;**13**(3):277-96. https://doi.org/10.1177/1363459308101804

Westbrook MT, Braithwaite J, Travaglia JF, Long D, Jorm C, Iedema RA. Promoting safety: longer-term responses of three health professional groups to a safety improvement programme. Int *J Health Care Qual Assur*. 2007;**20**(7):555-71. https://doi.org/10.1108/09526860710822707

Australia & Canada

Arah OA, Klazinga NS. How safe is the safety paradigm? *Qual Saf Health Care*. 2004;**13**(3):226-32. https://doi.org/10.1136/qshc.2003.007070

Cheng L, Sun N, Li Y, Zhang Z, Wang L, Zhou J, et al. International comparative analyses of incidents reporting systems for healthcare risk management. *J Evid Based Med*. 2011;**4**(1):32-47. https://doi.org/10.1111/j.1756-5391.2011.01119.x

Australia & New Zealand

Adamson L, Beldham-Collins R, Sykes J, Thwaites D. Safety culture and incident learning systems in radiation oncology: Staff perceptions across Australia and New Zealand. *J Med Imaging Radiat Oncol*. 2022;**66**(2):299-309. https://doi.org/10.1111/1754-9485.13335

Harrison R, Lee H, Sharma A. A survey of the impact of patient adverse events and near misses on anaesthetists in Australia and New Zealand. *Anaesth Intensive Care*. 2018;**46**(5):510-5. https://doi.org/10.1177/0310057X1804600513

Canada

Baker RG. Governance, policy and system-level efforts to support safer healthcare. *Healthc Q*. 2014;**17 Spec no**:21-6. https://doi.org/10.12927/hcq.2014.23955

Beard P, Smyrski L. Reporting for learning and improvement: the Manitoba and Saskatchewan experience. *Healthc Q*. 2006;**9**:61-4. https://doi.org/10.12927/hcq..18461

Beesoon S, Robert J, White J. Surgery Strategic Clinical Network: Improving quality, safety and access to surgical care in Alberta. *CMAJ*. 2019;**191**:S27-S9. https://doi.org/10.1503/cmaj.190590

Chartier LB, Ovens H, Hayes E, Davis B, Calder L, Schull M, et al. Improving quality of care through a mandatory provincial audit program: Ontario's emergency department return visit quality program. *Ann Emerg Med*. 2021;**77**(2):193-202. https://doi.org/10.1016/j.annemergmed.2020.09.449

Cochrane D, Taylor A, Miller G, Hait V, Matsui I, Bharadwaj M, et al. Establishing a provincial patient safety and learning system: pilot project results and lessons learned. *Healthc Q*. 2009;**12**:147-53. https://doi.org/10.12927/hcq.2009.20717

Cowell J, Harvie M. HQCA: building a credible, transparent, and independent healthcare quality and safety organization in Alberta. *Healthc Manage Forum*. 2012;**25**(4):185-7. https://doi.org/10.1016/j.hcmf.2012.09.011

Espin S, D'Arpino M, Indar A, Gross M. Incident management in health care: A pan-Canadian perspective. *J Nurs Care Qual*. 2022;**37**(1):E15-E21. https://doi.org/10.1097/NCQ.0000000000000572

Hewitt T, Chreim S, Forster A. Sociocultural factors influencing incident reporting among physicians and nurses: understanding frames underlying self- and peer-reporting practices. *J Patient Saf*. 2017;**13**(3):129-37. https://doi.org/10.1097/PTS.0000000000000130

Jeffs L, Baker GR, Taggar R, Hubley P, Richards J, Merkley J, et al. Attributes and actions required to advance quality and safety in hospitals: Insights from nurse executives. *Nurs Leadersh*. 2018;**31**(2):20-31. https://doi.org/10.12927/cjnl.2018.25606

Jeffs L, Bruno F, Zeng RL, Schonewille N, Kinder K, De Souza G, et al. Integrating implementation science in a quality and patient safety improvement learning collaborative: Essential ingredients and impact. *Jt Comm J Qual Patient Saf*. 2023;**49**(5):255-64. https://doi.org/10.1016/j.jcjq.2023.02.001

Kassam A, Sharma N, Harvie M, O'Beirne M, Topps M. Patient safety principles in family medicine residency accreditation standards and curriculum objectives: Implications for primary care. *Can Fam Physician*. 2016;**62**(12):e731-e9.

Law MP, Zimmerman R, Baker GR, Smith T. Assessment of safety culture maturity in a hospital setting. *Healthc Q*. 2010;**13**:110-5. https://doi.org/10.12927/hcq.2010.21975

Milligan C, Allin S, Farr M, Farmanova E, Peckham A, Byrd J, et al. Mandatory reporting legislation in Canada: improving systems for patient safety? *Health Econ Policy Law*. 2021;**16**(3):355-70. https://doi.org/10.1017/S1744133121000050

Milne JK, Lalonde AB. Patient safety in women's health-care: professional colleges can make a difference. The Society of Obstetricians and Gynaecologists of Canada MORE(OB) program. *Best Pract Res Clin Obstet Gynaecol*. 2007;**21**(4):565-79. https://doi.org/10.1016/j.bpobgyn.2007.01.013

Mitchell JI, Izad Shenas SA, Kuziemsky C. Governance standards: A roadmap for increasing safety at care transitions. *Healthc Manage Forum*. 2015;**28**(1):28-33. https://doi.org/10.1177/0840470414551894

Nicklin W, Mass H, Affonso DD, O'Connor P, Ferguson-Paré M, Jeffs L, et al. Patient safety culture and leadership within Canada's Academic Health Science Centres: towards the development of a collaborative position paper. Nurs Leadersh. 2004;**17**(1):22-34. https://doi.org/10.12927/cjnl.2004.16243

O'Beirne M, Reid R, Zwicker K, Sterling P, Sokol E, Flemons W, et al. The costs of developing, implementing, and operating a safety learning system in community practice. *J Patient Saf*. 2013;**9**(4):211-8. https://doi.org/10.1097/PTS.0000000000000052

O'Beirne M, Sterling PD. Medical safety and community practice: necessary elements and barriers to implement a safety learning system. *Healthc Q*. 2009;**12**:141-6. https://doi.org/10.12927/hcq.2009.20982

O'Beirne M, Sterling PD, Zwicker K, Hebert P, Norton PG. Safety incidents in family medicine. *BMJ Qual Saf*. 2011;**20**(12):1005-10. 10.1136/bmjqs-2011-000105

Skutezky T, Small SS, Peddie D, Balka E, Hohl CM. Beliefs and perceptions of patient safety event reporting in a Canadian Emergency Department: a qualitative study. *CJEM*. 2022;**24**(8):867-75. https://doi.org/10.1007/s43678-022-00400-2

Weaver RR. Seeking high reliability in primary care: Leadership, tools, and organization. *Health Care Manage Rev*. 2015;**40**(3):183-92. https://doi.org/10.1097/HMR.0000000000000022

Williams A. Nursing informaticians address patient safety to improve usability of health information technologies. *Stud Health Technol Inform*. 2019;**257**:501-7.

Zecevic AA, Ho-Ting Li A, Ngo C, Halligan M, Kothari A, Li AH-T. Improving safety culture in hospitals: Facilitators and barriers to implementation of Systemic Falls Investigative Method (SFIM). *Int J Qual Health Care*. 2017;**29**(3):371-7. https://doi.org/10.1093/intqhc/mzx034

Update search:

Kuluski K, Asselbergs M, Baker R, Burns KKK, Bruno F, Saragosa M, MacLaurin A, Flintoft V, Jeffs L. ‘Safety is about partnership’: Safety through the lens of patients and caregivers. *Health Expect*. 2024;**27**:e13939.

Recsky C, Rush KL, MacPhee M, Stowe M, Blackburn L, Muniak A, Currie LM. Clinical informatics team members' perspectives on health information technology safety after experiential learning and safety process development: Qualitative descriptive study. *JMIR Formative Research*. 2024;**5:**e53302.

Ireland

Flynn MA, Burgess T, Crowley P. Supporting and activating clinical governance development in Ireland: sharing our learning. *J Health Organ Manag*. 2015;**29**(4):455-81. https://doi.org/10.1108/JHOM-03-2014-0046

O'Connor P, O'Malley R, Kaud Y, St Pierre E, Dunne R, Byrne D, et al. A scoping review of patient safety research carried out in the Republic of Ireland. *Irish J Med Sci*. 2023;**192**(1):1-9. https://doi.org/10.1007/s11845-022-02930-1

Netherlands

Bilijam CW. Patient safety in general practice. *Tijdschrift voor Kindergeneeskunde*. 2014;**82**(5):155-9.

Cords CI, van der Vlies CH, Stoop M, Nieuwenhuis MK, Boudestein K, Mattace-Raso FUS, et al. Frailty screening practice in specialized burn care-a retrospective multicentre cohort study. *Eur Burn J*. 2023;**4**(1):87-100. https://doi.org/10.3390/ebj4010009

de Korne DF, Van Wijngaarden JDH, Van Dyck C, Hiddema UF, Klazinga NS. Evaluation of aviation-based safety team training in a hospital in The Netherlands. *J Health Organ Manag*. 2014;**28**(6):731-53. https://doi.org/10.1108/jhom-01-2013-0008

Harmsen M, Gaal S, van Dulmen S, de Feijter E, Giesen P, Jacobs A, et al. Patient safety in Dutch primary care: a study protocol. *Implement Sci*. 2010;**5**:50. https://doi.org/10.1186/1748-5908-5-50 Heim N, van Fenema EM, Weverling-Rijnsburger AW, Tuijl JP, Jue P, Oleksik AM, et al. Optimal screening for increased risk for adverse outcomes in hospitalised older adults. *Age Ageing*. 2015;**44**(2):239-44. https://doi.org/10.1093/ageing/afu187

Hermans MPJ, Eindhoven DC, van Winden LAM, de Grooth GJ, Blauw GJ, Muller M, et al. Frailty score for elderly patients is associated with short-term clinical outcomes in patients with ST-segment elevated myocardial infarction treated with primary percutaneous coronary intervention. *Neth Heart J*. 2019;**27**(3):127-33. https://doi.org/10.1007/s12471-019-1240-7

Jepma P, Verweij L, Tijssen A, Heymans MW, Flierman I, Latour CHM, et al. The performance of the Dutch Safety Management System frailty tool to predict the risk of readmission or mortality in older hospitalised cardiac patients. *BMC Geriatr*. 2021;**21**(1):299. https://doi.org/10.1186/s12877-021-02243-5

Jessurun JG, Hunfeld NGM, Van Rosmalen J, Van Dijk M, Van Den Bemt P. Effect of automated unit dose dispensing with barcode scanning on medication administration errors: an uncontrolled before-and-after study. *Int J Qual Health Care*. 2021;**33**(4):13. https://doi.org/10.1093/intqhc/mzab142

Oud FMM, Schut MC, Spies PE, van der Zaag-Loonen HJ, de Rooij SE, Abu-Hanna A, et al. Interaction between geriatric syndromes in predicting three months mortality risk. *Arch Gerontol Geriatr*. 2022;**103:**104774. https://doi.org/10.1016/j.archger.2022.104774

Oud FMM, Wolzak NK, Spies PE, Zaag-Loonen HJV, van Munster BC. The predictive value of the 'VMS frail older patients' for adverse outcomes in geriatric inpatients. *Arch Gerontol Geriatr*. 2021;**97**:104514. https://doi.org/10.1016/j.archger.2021.104514

Schuijt HJ, Oud FMM, Bruns EJR, van Duijvendijk P, Van der Zaag-Loonen HJ, Spies PE, et al. Does the Dutch Safety Management Program predict adverse outcomes for older patients in the emergency department? *Neth J Med*. 2020;**78**(5):244-50.

Snijders BMG, Emmelot-Vonk MH, Souwer ETD, Kaasjager HAH, van den Bos F. Prognostic value of screening instrument based on the Dutch national VMS guidelines for older patients in the emergency department. *Eur Geriatr Med*. 2021;**12**(1):143-50. https://doi.org/10.1007/s41999-020-00385-0

Snijders C. Patient safety in the neonatal intensive care unit: A look back on the NEOSAFE study. *Tijdschrift voor Kindergeneeskunde*. 2014;**82**(5):160-5.

van Dam CS, Trappenburg MC, Ter Wee MM, Hoogendijk EO, de Vet HC, Smulders YM, et al. The accuracy of four frequently used frailty instruments for the prediction of adverse health outcomes among older adults at two Dutch emergency departments: Findings of the AmsterGEM study. *Ann Emerg Med*. 2021;**78**(4):538-48. https://doi.org/10.1016/j.annemergmed.2021.04.027

van der Starre, C., van Dijk, M. & Tibboel, D. Real-time registration of adverse events in Dutch hospitalized children in general pediatric units: first experiences. *Eur J Pediatr.* 2012;**171**:553–558. https://doi.org/10.1007/s00431-011-1608-1

van der Zanden V, Paarlberg KM, van der Zaag-Loonen HJ, Meijer WJ, Mourits MJE, van Munster BC. Risk assessment for postoperative outcomes in a mixed hospitalized gynecological population by the Dutch safety management system (Veiligheidsmanagementsysteem, VMS) screening tool 'frail elderly'. *Arch Gynecol Obstet*. 2021;**304**(2):465-73. https://doi.org/10.1007/s00404-021-06073-z

van Munster BC, Drost D, Kalf A, Vogtlander NP. Discriminative value of frailty screening instruments in end-stage renal disease. *Clin Kidney J*. 2016;**9**(4):606-10. https://doi.org/10.1093/ckj/sfw061

van Schoten SM, Baines RJ, Spreeuwenberg P, de Bruijne MC, Groenewegen PP, Groeneweg J, et al. The ecometric properties of a measurement instrument for prospective risk analysis in hospital departments. *BMC Health Serv Res*. 2014;**14**:103. https://doi.org/10.1186/1472-6963-14-103

Verstappen W, Gaal S, Bowie P, Parker D, Lainer M, Valderas JM, et al. A research agenda on patient safety in primary care. Recommendations by the LINNEAUS collaboration on patient safety in primary care. *Eur J Gen Pract*. 2015;**21**(sup1):72-7. https://doi.org/10.3109/13814788.2015.1043726

Warnier RMJ, van Rossum E, van Kuijk SMJ, Magdelijns F, Schols J, Kempen G. Frailty screening in hospitalised older adults: How does the brief Dutch National Safety Management Program perform compared to a more extensive approach? *J Clin Nurs*. 2020;**29**(7-8):1064-73. https://doi.org/10.1111/jocn.15148

Weske U, Boselie P, van Rensen E, Schneider M. Physician compliance with quality and patient safety regulations: The role of perceived enforcement approaches and commitment. *Health Serv Manage Res*. 2019;**32**(2):103-12. https://doi.org/10.1177/0951484818813324

Weske U, Boselie P, van Rensen ELJ, Schneider MME. Using regulatory enforcement theory to explain compliance with quality and patient safety regulations: the case of internal audits. *BMC Health Serv Res*. 2018;**18**(1):62. https://doi.org/10.1186/s12913-018-2865-8

Update search:

Oud FMM, Meulman MD, Merten H, Wagner C, van Munster BC. Value of the Safety Management System (VMS) frailty instrument as a frailty screener in care for older hospital patients: a systematic review. *Eur Geriat Med*. 2024;**15**:609-620.

New Zealand

Raymont A, Graham P, Hider PN, Finlayson MP, Fraser J, Cumming JM. Variation in the adoption of patient safety practices among New Zealand district health boards. *Aust Health Rev*. 2012;**36**(2):163-8. https://doi.org/10.1071/AH10972

Robin G, Horsburgh S. Healthcare professional perspectives on quality and safety in New Zealand public hospitals: findings from a national survey. *Aust Health Rev*. 2014;**38**(1):109-14. https://doi.org/10.1071/AH13116

Shuker C, Bohm G, Bramley D, Frost S, Galler D, Hamblin R, et al. The Health Quality and Safety Commission: making good health care better. *N Z Med J*. 2015;**128**(1408):97-109.

Mobius Research & Strategy. The Health Quality & Safety Commission Surgical Safety Culture Survey Research Report. 2017. URL: https://www.hqsc.govt.nz/assets/Our-work/Improved-service-delivery/Safe-surgery/Publications-resources/2017_Surgical_Safety_Culture_Survey_Report_-_FINAL.pdf (accessed 24 April 2024).

Supplementary material 4: Rich and descriptive studies

Documents relating to patient safety and/or health services in each country included in the review

**Document**	**Year of publication**	**Description of document**
**Netherlands**		
Policy		
NTA 8009 'Safety management system for hospitals and institutions that provide hospital care' (initial technical agreement).[29]*	2007	Describes the basic requirements for an SMS for hospitals and institutions that provide hospital care. It focuses on managing risks and reducing harm to patients. It is based on the Rein Willems report '*Here you work safely or you don't work here'* (2004) and the improvement cycle 'plan-do-check-act' on which other SMS are based. The structure of the document includes: 1. Subject and scope, 2. Terms and definitions, 3. Responsibilities of the Board of Directors, and 4. Basic requirements for the SMS. Additional information is provided in the appendices on the organisations involved in the drafting of the document; the reporting matrix (roles and responsibilities for reporting different types of incidents such as near misses and serious incidents); conversion tables that explain how the NTA relates to other standards (e.g. ISO 9001: 2000 Quality Management System standard); examples of prospective and retrospective risk assessment; and safety culture (which is not explicitly included in the basic requirements).
NEN 8009 'Safety management system for hospitals and institutions that provide hospital care' (replaces NTA 8009 2014).[30]*	2018	This is the third update of the original NTA 8009 (following updates in 2011 and 2014). It supplements, without replacing, the original SMS requirements with Safety-II concepts that promote a proactive safety culture and encourage learning from daily activities with desired outcomes as well as incidents. It acknowledges that improvement could vary from continuous improvements in small steps to significant changes at organisational level and highlights the importance of encouraging employees and patients to play an active role, identify opportunities and successful practices and contribute to the overall process of organisational learning.

de Bell S, *et al. BMJ Open* 2026; 16:e107772. doi: 10.1136/bmjopen-2025-107772

Other				
Bal R, Wagner C. The Netherlands: Patient safety in Dutch hospitals: How can we explain success, in Health systems improvement across the Globe: Success stories from 60 countries, eds. Braithwaite J et al. Boca Raton: CRC Press.[31]	2018	Provides an overview of the Dutch patient safety programme and tries to explain its success, accounting for the broader socio-cultural and historical context, and identifying the 'conditions under which such a programme was able to succeed' (p. 208). It argues that five such factors contributed to the success of the Dutch patient safety programme: experience in project work, market competition, public pressure, activities of regulatory agencies, and public reporting of adverse events and mortality.		
Bolk M. Final report: Embedding patient safety in education and training. Utrecht: VMS Safety Programme.[32]	2013	Reports on the '*Embedding patient safety in education and training*' project which aimed 'to support, facilitate and accelerate education for doctors, nurses and the supporting professions (medical and otherwise), with the help of a national incentive program and the corresponding support structure' (p. 4). As a result, a national 'patient safety competencies framework' was developed; all findings from the project were compiled in a digital portal and campaign website and a 'patient safety toolkit' was developed; examples for good patient safety education were collected; and the attention generated regarding patient safety resulted in the project linking to other educational initiatives already working on patient safety. The project made a number of recommendations for embedding patient safety in various educational and learning processes and programmes.		
Research		**Aims**	**Methods**	**Findings**
Baines R et al. How effective are patient safety initiatives? A retrospective patient record review study of changes to patient safety over time. *BMJ Qual Saf*. 2015;**24**(9):561-71.[33]	2015	To assess whether hospital care in 2011/12 was safer than in 2004 and 2008.	Retrospective patient record review. 15, 997 patient admissions were included, 7926 from 2004, 4023 from 2008 and 4048 from 2011/2012.	In comparison with 2008, uncorrected crude overall adverse event rates showed no change in 2011/2012 but preventable adverse event rates showed a reduction of 45%. After multilevel corrections, the

				decrease was still visible but not statistically significant.
de Blok C et al. Implementation of the Dutch National Patient Safety Programme (VMS Veiligheidsprogrammesma). Utrecht/Amsterdam: NIVEL/EMGO+.[34]	2013	English summary of de Blok[21] (see below).		
de Blok C, Koster E, Schilp J, Wagner C. Implementatie VMS Veiligheidsprogrammesma. Evaluatieonderzoek in Nederlandse ziekenhuizen. Utrecht / Amsterdam: NIVEL/EMGO+.[21*]	2013	To assess the extent to which the ten themes of the Dutch patient safety programme have been implemented and gain insight into the factors that influence the degree of implementation and compliance with the advice.	Data collection varied across themes and included observations and semi-structured interviews, review of patient records and registry data. A large proportion of Dutch hospitals were involved in the evaluation, which varied across themes.	Implementation objectives set in 2008 were almost achieved for some of the themes (e.g. ‘Preventing renal insufficiency during intravascular use of iodinated contrast agent’ and subtheme ‘Treatment of severe sepsis’). There was a positive trend for other themes, but considerable variation was observed across and within hospitals. The study identified a range of organisational, staff- and theme-related factors associated with the success of implementation.
de Bruijne MC, Zegers M, Hoonhout LHF, Wagner C. Onbedoelde schade in Nederlandse ziekenhuizen: Dossieronderzoek van ziekenhuisopnames in 2004. Amsterdam: EMGO+ Instituut, Utrech: NIVEL[35*]	2007	To establish a baseline measurement in the field of patient safety.	Retrospective patient record review; 200 patient records from patients who were discharged and 200 from patients who died in hospitals in 2004 were randomly selected from each of the participating 21	Of all admissions: (i) 5.7% suffered unintentional healthcare-related harm, 5% of which experienced permanent harm and 8% died partly as a result of this; (ii) 2.3% suffered preventable adverse events. Of the patients who died in hospital,

			Dutch hospitals. Patients admitted to psychiatry and obstetrics and those less then one year of age were excluded as the assessment system was not suitable for such patients.	10.7% suffered unintentional healthcare-related events; of those, 4.1% were related to preventable adverse events during admission. It was estimated that in 2004, there were 1735 (95%CI 1482 to 2032) potentially preventable deaths in Dutch hospitals.
de Groot JF et al. Implementing paediatric early warning scores systems in the Netherlands: future implications. *BMC Pediatr*. 2018;**18**(1):128.[36]	2018	(i) To evaluate the implementation of paediatric early warning score systems in five hospitals.<br>(ii) To gain insight into the attitudes of professionals towards the system.	(i) Retrospective patient record review (n = 554) at the start, mid and endpoint of implementation.<br>(ii) Semi-structured interviews with professionals (n = 8).	(i) None of the hospitals started with a system but after 1 year, they were recorded in 69.2% of patient records and resulted in appropriate action in 49.1%.<br>(ii) Sustainable implementation might be hindered by staff concerns about validity, effectiveness and communication with other hospitals during transfers to higher levels of care.
Klopotowska J, Schutijser B, de Bruijne M, Wagner C. Tweede evaluatie van VMS Programma in Nederlandse ziekenhuizen op basis van vier veiligheidsthema's: aangevuld met EPD Scan naar mogelijkheden voor toekomstige metingen. Utrecht/Amsterdam: NIVEL/EMGO+.[37*]	2016	To investigate the progress of the implementation of four safety themes from the Dutch national patient safety programme compared to the first evaluation study in 2011/2012 and assess the possibility of automatic monitoring of process indicators from	Data collection:<br>(i) Theme 'medication verification': review of patient records (n=1060) in 15 hospitals.<br>(ii) Theme 'high risk medication': observations of nurses (n=349) in 15 hospitals.	There was improvement in compliance for (i) and (ii) but still the target of 100% was not met; authors note that 100% compliance may not be desirable as in some cases deviation from guidelines could be beneficial for the patient. For (iii) compliance increased by 34% (to 73% against 90% target). For (iv) hospital mortality among severe sepsis patients

		electronic patient records for two of the themes.	Themes (iii) 'prevention of wound infections after surgery' and (iv) 'prevention of line sepsis and treatment of severe sepsis': data from national registries.	has fallen from 34% to 29% (15% relative mortality reduction) but for line sepsis positive results were observed only for the ICU departments on which the hospitals focused initially (incidence was 2.5 times higher in non-ICU).
Langelaan M et al. Monitor zorggerelateerde schade 2008: Dossieronderzoek in Nederlandse ziekenhuizen. Utrecht: NIVEL, Amsterdam: EMGO+ Instituut.[38]*	2010	To estimate and characterise potentially avoidable adverse events and deaths, and those related to the use of medical technology; to compare them to 2004.	Retrospective patient record review: stratified samples of 100 admissions of discharged patients and 100 of patients who died in hospital were drawn from each of the 20 participating hospitals. Psychiatry, obstetrics and patients < 1 year of age were excluded.	2.9% potentially avoidable adverse events (of all admissions). The change from 2004 was not statistically significant; 0.8% was related to the use of medical technology. Potentially avoidable mortality was 5.5% (95%CI 4.5% to 6.6%); the change from 2004 was not statistically significant. In absolute numbers, in 2008 there were 1960 (95%CI 1600 to 2460) patients in whom potentially avoidable harm contributed to the death.
Langelaan M et al. Monitor Zorggerelateerde Schade 2011/2012. Dossieronderzoek in Nederlandse ziekenhuizen. Amsterdam: VU medisch centrum & EMGO+ Instituut, Utrecht: NIVEL.[39]*	2013	To estimate and characterise potentially avoidable adverse events and mortality in Dutch hospitals, including those related to medical technology, and compare them to previous measurements.	Retrospective patient record review study based on data from 20 randomly selected Dutch hospitals (>4000 records in total); random samples were drawn from each hospital of 100 discharged patients and 100 patients who died in the hospital. Psychiatry,	Of all patients admitted in 2011/2012, 1.6% (95% CI: 1.1% to 2.2%) experienced potentially avoidable harm: 30% and 45% less than in 2004 and 2008, respectively. Potentially avoidable mortality decreased by 37% and 53% compared to 2004 and 2008, respectively, which expressed in absolute

		To estimate the costs of healthcare-related harm and the quality of file management.	obstetrics and patients < 1 year of age were excluded.	numbers and extrapolated to all Dutch hospitals was approx. 1,735 patients (95% CI: 1,482 to 2,032) in 2004, 1,960 patients (95% CI: 1,600 to 2,360) in 2008, and 970 (95% CI: 738 to 1,274) patients between 1st April 2011 and 31st March 2012. Other findings included harms related to the application of medical technology, costs related to adverse events and the quality of patient records.
Langelaan M et al. Monitor Zorggerelateerde Schade 2015/2016: Dossieronderzoek bij overleden patiënten in Nederlandse ziekenhuizen. Utrecht: NIVEL, Amsterdam: EMGO+ Instituut.[40]*	2017	To estimate and characterise potentially avoidable adverse events and mortality in Dutch hospitals, including those related to medical technology, and compare them to previous measurements.	Retrospective patient record review: a random sample of 150 patients who died in hospital was drawn from each of the 19 hospitals, randomly selected to participate in the study. Psychiatry, obstetrics and patients < 1 year of age were excluded.	Of all deceased patients, 4.3% (95%CI 3.6% to 5.1%) suffered potentially preventable adverse events and 3.1% (95%CI 2.5% to 3.8%) suffered potentially preventable mortality. The difference with 2011/12 was not statistically significant.<br>The respective results related to the use of medical technology were 1.7% and 1.3%, out of all cases in which technology was used.
Meulman M, Merten H, Wagner C. Het screenen van kwetsbare ouderen: onderzoek vanuit een Safety-I en Safety-II perspectief: evaluatie van het VMS thema	2021	To investigate the degree of compliance with the national Dutch patient safety programme screening	Functional Resonance Analysis Method was used, based on analysis of hospital protocols, interviews with nurses (n=31), and a feedback meeting. Additionally, a	Hospital protocols were more detailed than the national guidelines but largely similar. There were actions in the protocols that were not performed, and actions that were performed but not included

Kwetsbare ouderen. Utrecht: NIVEL[41]*		tool for vulnerable older people.	survey of employees' resilience (n=146), an evaluation interview in each department, and compliance data (the percentage of admitted patients aged ≥70 who were screened). Data were collected between 2020-2021, from 10 departments in 6 hospitals.	in the protocols. An average of 74% of admitted patients were screened. Individual resilience (66%) was rated more positively than team resilience (49%).
Meurs M, de Groot JF. Veiligheidsmanagement systeem (VMS) thema's kindzorg Pijn en (Lijn)sepsis: verbeterpunten vanuit het werkveld voor verdere kwaliteitsverbetering. Utrecht: NIVEL. [42]*	2020	To identify bottlenecks and areas of improvement for implementation of the Dutch national patient safety programme themes '(line) sepsis' and 'pain' within paediatric care hospitals.	Working groups with paediatricians and paediatric nurses, and a survey (n=209). Data was collected in 2019.	Theme '(line) sepsis': the main recommendation regarded earlier recognition and treatment of sepsis (e.g. through training).<br>Theme 'pain': the main recommendation for improvement was to revise guidelines to adopt a holistic approach to pain recognition and treatment.
Sambeeck SJV et al. Pediatric Early Warning System Scores: Lessons to be learned. *J Pediatr Intensive* Care. 2018;**7**(1):27-32.[43]	2018	To investigate how many hospitals complied with the national recommendation to implement a paediatric early warning system score and if so, the score they chose to implement.	Cross-sectional electronic survey of Dutch hospitals in 2014 (n=91).	Three-quarters of Dutch hospitals with a paediatric department implemented a score. These hospitals used 45 different versions of a paediatric early warning system score, the majority of which were not validated.

Schlinkert C, Jelsma J, Wagner C. Patiëntveiligheidscultuur in Nederlandse Ziekenhuizen. Van openheid naar reflectie en veerkracht. Utrecht: NIVEL.[44]*	2021	To measure the patient safety culture in Dutch hospitals in 2020 and compare it with previous measurements (before and after the implementation of the Dutch patient safety programme). To measure the employees' resilience.	Cross-sectional survey on patient safety culture (Dutch version of Hospital Survey on Patient Safety Culture) and employees' resilience. Data were collected in 2020; 3014 employees from 5 hospitals completed the survey. Comparison with data collected using the same survey in 2005-2007 and 2012.	62.5% of respondents rated patient safety in their own department 'very good' or 'excellent'. Teamwork was the highest rated dimension, 'Adequate staffing', 'Support from management' and 'Collaboration between departments' were rated lowest. Nursing staff rated patient safety more negatively than other professional groups. More respondents considered patient safety to be 'very good' or 'excellent' in 2020 compared to previous measurements.
van Dijk LM, van Eikenhorst L, Muns L, Wagner C. Medicatieverificatie bij ontslag: nieuwe benadering vanuit een Safety-I en Safety-II perspectief: derde evaluatie van het VMS-thema. Utrecht: NIVEL.[45]*	2021	To investigate the implementation of the 'medication verification at discharge' theme of the Dutch national patient safety programme.	Functional Resonance Analysis Method was used, based on hospital protocols, interviews with nurses (n=77), and a feedback meeting. A survey of employees' resilience (n=142) and an evaluation interview in each department were carried out, and compliance data were collected (percentage of discharged patients aged ≥18 for whom medication verification took place upon discharge). Data was collected between 2020-	Hospital protocols were more detailed than the national guidelines. There were variations in how the processes were carried out in practice, normally in response to circumstances in the work environment (e.g. IT problems, staff shortages). Compliance rates varied between 67%-100% across departments over the data collection period. Both individual (64%) and team (69%) resilience was rated positively.

			2021, from 9 departments in 8 hospitals (4 cardiology departments and 5 orthopaedics departments).	
van der Starre C et al. Paediatric critical incident analysis: lessons learnt on analysis, recommendations and implementation. *Eur J Pediatr*. 2014;**173**(11):1449-57.[46]	2014	To identify potential causal and contributing factors of serious paediatric patient safety incidents, and to report on and assess the extent of implementation of recommendations for improvement.	Retrospective review of critical incident analyses (n=17) from 2005–2010.	A median number of 5 causal and contributing factors was identified per incident and were mostly likely to be team or task-related. There was a median of 5 recommendations per analysis, only one-third of recommendations were implemented.
van Noord N, Zwijnenberg **I**, Wagner C. Patiëntveiligheidscultuur in Nederlandse Ziekenhuizen. Een stap in de goede richting. Utrecht: NIVEL.[47]*	2013	To examine whether the Dutch national patient safety programme improved patient safety culture.	Comparison of two cross-sectional safety culture surveys (Dutch version of Hospital Survey on Patient Safety Culture). Between 2005-2007, 3779 healthcare providers in 45 hospitals responded, in 2012, 6605 healthcare providers in 24 hospitals responded.	Patient safety culture improved between 2005-2007 and 2012, with 48.8% of respondents rating it ‘very good' or 'excellent’ in 2012. However, there was variation between hospitals and departments. ‘Teamwork’ was the highest rated dimension of safety; 'Collaboration between departments', ‘Reporting frequency' and 'Support from management' were lowest rated. Nursing staff rated patient safety more negatively than other professionals.
van Schoten S et al. The association between quality system development stage and	2018	To examine the association between the development stage	Analysis of a national survey on the development stage of hospital quality	No association was found between the development stage of a hospital quality system and

the implementation of process-level patient safety themes in Dutch hospitals: an observational study. *BMC Health Serv Res.* 2018;**18**:189.[48]		of hospital quality systems and the process-level implementation of patient safety themes within the Dutch patient safety programme.	systems (conducted in 2011) and an evaluation study of the national Dutch patient safety program (2011-2012). Data from 30 hospitals (12,485 observations) were included.	the process-level implementation of patient safety themes.
van Schoten S et al. Monitor Zorggerelateerde Schade 2019: Dossieronderzoek bij overleden patiënten in Nederlandse ziekenhuizen. Utrecht: NIVEL, Amsterdam: APH.[49*]	2022	To estimate and characterise potentially avoidable adverse events and mortality in Dutch hospitals, including those related to medical technology and diagnostic processes, and those in vulnerable older patients, and compare them to previous measurements. To assess the quality of file management.	Retrospective patient record review: a random sample of 150 patients who died in hospital was drawn from each of the 20 hospitals, randomly selected to participate in the study. Psychiatry, obstetrics and patients < 1 year of age were excluded.	14.6% (95%CI 13.3% to 16.0%) of patients who died in hospital suffered care-related harm. This is a significant increase compared to 2015/2016 (was 9.9% (95% CI 8.9% to 11.0%)). Potentially avoidable adverse events and mortality were 4.2% (95%CI 3.5% to 5.0%) and 3.1% (95% CI 2.5% to 3.9%), respectively, indicating that the change from the previous measurement was not statistically significant.
van Stralen S, Schutijser B, Vonk A, van Eikenhorst L, Wagner C. Tweede controle bij het klaarmaken en toedienen van high-risk medicatie: nieuwe benadering vanuit een Safety-I en Safety-II perspectief. Derde evaluatie van het VMS thema. Utrecht: NIVEL. [50*]	2021	To investigate the implementation of the 'high-risk medication' theme (specifically the second check of medication) of the Dutch national patient safety programme.	Functional Resonance Analysis Method was used, based on hospital protocols, interviews with nurses (n=77), and a feedback meeting. Additionally, a survey of employee resilience (n=142) and an evaluation interview in each	Compared to the national guidelines from the patient safety programme, hospital protocols were largely the same. There were variations in how nurses carried out the processes, normally in response to circumstances in the work environment (e.g. time pressures). Individual (average

			department were carried out, and compliance data collected. Data was collected between 2020-2021, from 10 departments in 9 hospitals (3 surgery departments, 3 internal medicine departments and 4 intensive care units).	score 3.21/9) and team resilience (average score 4.08/9) was rated highly.
Verbeek-van Noord I et al. A nation-wide transition in patient safety culture: a multilevel analysis on two cross-sectional surveys. *Int J Qual Health Care*. 2019;**31**(8):627–632.[51]	2019	To examine whether the Dutch national patient safety programme improved patient safety culture.	Comparison of two cross-sectional safety culture surveys (Dutch version of Hospital Survey on Patient Safety Culture). Between 2005-2007, 3779 healthcare providers in 45 hospitals responded, in 2012, 6605 healthcare providers in 24 hospitals responded.	Patient safety culture improved over the studied period. However, there was variation between hospitals and departments.
**Australia**				
Policy				
Australian Commission on Safety and Quality in Health Care. National model clinical governance framework.[63]	2017	This framework defines clinical governance as ‘a set of relationships and responsibilities established by a health service organisation between its state or territory department of health, governing body, executive, workforce, patients, consumers and other stakeholders’.(p.iii) The framework contains all the elements that acute health service organisations need to implement integrated corporate and clinical governance systems, with the aim of ensuring good clinical outcomes through the delivery of safe and quality care. It is based on the National Safety and Quality Health Service Standards (NSQHS) Clinical Governance and Partnering with Consumers (see below) and contains five components: governance, leadership and culture, patient safety and quality		

		improvement systems, clinical performance and effectiveness, safe environment for the delivery of care, and partnering with consumers.		
Australian Commission on Safety and Quality in Health Care. National safety and quality health service standards. 2nd edition.[64]	2021	Describe standards intended for organisation-wide implementation in all hospital and day procedure services to enable the delivery of nationally consistent, safe and quality care. There are eight Standards - Clinical Governance, Partnering with Consumers, Preventing and Controlling Healthcare-Associated Infection, Medication Safety, Comprehensive Care, Communicating for Safety, Blood Management and Recognising and Responding to Acute Deterioration. The Clinical Governance and Partnering with Consumers Standards set out overarching requirements for the implementation of the other six Standards. The Standards do not specify how they should be implemented to allow for local adaptation.		
Australian Commission on Safety and Quality in Health Care. National safety and quality primary and community healthcare standards.[65]	2021	Describes a framework which is intended for use in all primary and community healthcare services. The processes and structures in the Standards are intended to enable the delivery of, and improve, the quality and safety of services. There are three Standards - Clinical Governance, Partnering with Consumers, and Clinical Safety – with the former two Standards setting out overarching requirements for the implementation of the Clinical Safety Standards.		
Research		**Aims**	**Methods**	**Findings**
Australian Commission on Safety and Quality in Health Care. Creating safer, better healthcare - the impact of national safety and quality health service standards.[66]	2018	To provide an overview of changes associated with implementation of the first edition of the NSQHS Standards, identifying areas where improvement has been made and where further work is needed.	Draws on a numbers of research projects, including surveys of hospital staff, hospital board members, and specialist medical colleges, National Inpatient Medication Chart Audits, and a Consumer Participation Study involving interviews and focus groups.	Implementation of the NSQHS Standards has resulted in the prevention of harm, improvements in patient care including empowerment of consumers and patients, development of better governance systems, and reduction of waste.

Australian Commission on Safety and Quality in Health Care. The state of patient safety and quality in Australian hospitals 2019.[67]	2019	To report on safety and quality in public hospitals in Australia, including common patient safety risks, action taken to deliver appropriate care, and the Commission's approach to supporting value-based healthcare.	Draws on data on public hospitals from a wide range of sources, such as the Admitted Patient Care National Minimum Data Set, 2013–14 to 2017–18 and the Australian Atlas of Healthcare Variation.	There have been significant improvements in the safety and quality of care over the last 10 years. These include improved patient outcomes such as reductions in hospital acquired infections and preventable in-hospital cardiac arrests, improved patient experience and involvement in care, and better governance of clinical care.
**Canada**				
Policy				
Canadian Patient Safety Institute. Canadian disclosure guidelines. Being open with patients and family.[68]	2011	'The guidelines are intended to encourage and support healthcare providers, interdisciplinary teams, organizations and regulatory authorities in developing and implementing disclosure policies, practices and training methods.' (p. 13) The following guiding principles underpin the development of the guidelines: patient-centred healthcare, patient autonomy, safe healthcare, leadership support, disclosure is the right thing to do, honesty and transparency. It recommends the following elements to be included in a disclosure policy: policy statement/objectives, definitions of key terms, provision for patient support, provision for healthcare provider support and education, the disclosure process, provision for particular circumstances (Appendix C).		
Canadian Patient Safety Institute. Strengthening commitment for improvement together. A policy framework for patient safety.[69]	2019	A policy framework for patient safety aiming to stimulate conversation and action on the following policy levers: regulations, standards, organisational policies and public engagement.		
Health Standards Organisation & Canadian Patient Safety Institute. The Canadian quality and patient	2020	Defines five areas - people-centred care, safe care, accessible care, appropriate care, and integrated care – for health services to focus on to provide safe and quality care. These goal areas are intended 'to drive improvement and to align Canadian legislation, regulations, standards, organizational policies, and public engagement on patient safety		

safety framework for health services.[70]		and quality improvement.'(p.3) The framework includes actions guides customised to five stakeholder groups - the public, health teams, health leaders, board members and policymakers - which identify objectives and outcomes for each goal, indicators to track success, and resources.		
Healthcare Excellence Canada. Status of patient safety incident legislation and best practices across Canada. Summary report.[71]	2021	Summarises and compares the status of four types of patient safety incident legislation - mandatory reporting, mandatory disclosure, apology protection, and quality assurance - in federal, provincial and territorial jurisdictions across Canada. The report also describes important characteristics of each type of legislation and comments on current or potential changes in policy direction which may be significant for the future development of legislation.		
Healthcare Excellence Canada, Patients for Patient Safety Canada. Rethinking patient safety. A discussion guide for patients, healthcare providers and leaders.[72]	2023	Summarises discussions with health service users, healthcare professionals, and safety scientists. Describes a new approach to patient safety in Canada, involving (i) the development of a positive culture of safety, with everyone contributing to creating safe conditions, (ii) acknowledging that the absence of harm does not mean that care is safe, and (iii) that all forms of harm matter, including those that are not physical.		
Research		**Aims**	**Methods**	**Findings**
Goldman J, Rotteau L. Evaluation research of Measurement and Monitoring of Safety Framework collaborative.[73]	2020	To (i) understand the effectiveness of a learning collaborative in teaching participants about the Measurement and Monitoring of Safety Framework (MMSF), and (ii) examine the implementation of the MMSF.	Qualitative study with three types of data collection:<br>(i) Interviews (n=36, with participants from teams including emergency, surgery, medicine, cardiology, psychiatry, supportive living and long-term care settings).<br>(ii) Observations of sites (n=5) where MMSF was being implemented and learning sessions and calls	Participants found the sessions to learn about MMSF useful, but some felt overwhelmed by the amount of information. Coaching was felt to play a key role in the implementation of MMSF in participants' organisations. A range of context-specific strategies were used by participants to implement MMSF (e.g. teaching to groups of stakeholders, integrating its language into usual communication, using it to

			where participants learnt about MMSF (29 hours were spent on site visits and 33 hours at a learning session).<br>(iii) Documentary data collection (e.g. learning session resources, tools being used in MMSF implementation).	inform activities such as safety huddles and incident reporting). Most participants reported positive impacts from MMSF implementation (e.g. changes in thinking about safety and resulting behaviour change); they supported its wider spread but found opportunities to do this beyond their sites were variable.
Goldman J, Rotteau L, Flintoft V, *et al* Measurement and Monitoring of Safety Framework: a qualitative study of implementation through a Canadian learning collaborative *BMJ Quality & Safety* 2023; **32:**470-478.[74]	2023	Journal article summarising the report[73] described above.		
**Ireland**				
Policy				
Health Information and Quality Authority. National standards for safer better healthcare.[75]	2012	Describes eight standards: person-centred care and support, effective care and support, safe care and support, better health and wellbeing, leadership, governance and management, workforce, use of resources, and use of information. These were intended to set expectations for healthcare in Ireland and guide improvement of quality, safety, and reliability. They were replaced recently by a principles-based approach.[76]		
Health Information and Quality Authority. National standards for the conduct of reviews of patient safety incidents.[77]	2017	Details standards based around five themes: governance and accountability, person-centred approach to the review of patient safety incidents, workforce, reviews of patient safety incidents, and sharing the learning for improvement. These are intended to ensure a consistent national approach to reviews of patient safety incidents.		

Health Information and Quality Authority. Standards development framework: a principles-based approach.[76]	2021	Describes a set of principles which are designed to underpin all national standards for health and social care services, to be used as framework during their development. These principles are a human rights-based approach, safety and wellbeing, responsiveness, and accountability; they are all intended to ensure patients receive person-centred care and support.
Health Service Executive. Patient safety strategy 2019-2024.[78]	2019	Details eight patient safety commitments (including specific actions within each commitment, and the rationale and principles behind them): empowering and engaging patients to improve patient safety, empowering and engaging staff to improve patient safety, anticipating and responding to risks to patient safety, reducing common causes of harm, using information to improve patient safety, and leadership and governance to improve patient safety. Its primary purpose is to guide safety improvement at the service level.
Health Service Executive. Healthcare audit – quality assurance and verification. Standards.[79]	2019	Sets out a framework and basic principles – integrity, objectivity, confidentiality, and competency - for the conduct of healthcare audits in Ireland. All HSE healthcare organisations and private or voluntary bodies with a service level agreement with the HSE are required to be audited (in addition to their internal audit functions) by HSE Healthcare auditors, who are members of the Chartered Institute of Internal Auditors. Audits are independent, objective assessments of the delivery of healthcare services. They provide assurance that standards are being met and recommendations to drive improvement.
Health Service Executive. Incident management framework.[80]	2020	A framework that describes a practical approach all healthcare providers can use to identify, report and review incidents. The framework focuses on the system rather than individual. Six steps are described in the process: preventing incidents by supporting a culture where safety is a priority, identifying incidents and immediate actions, initial reporting and notification, categorisation and initial assessment (from category 1, a major incident, to category 3, a minor incident, which determines who needs to be notified and the level of review required), review and analysis, and improvement planning and monitoring.

de Bell S, *et al. BMJ Open* 2026; 16:e107772. doi: 10.1136/bmjopen-2025-107772

Health Service Executive. HSE Enterprise risk management policy and procedures 2023.[81]	2023	A practical resource for healthcare professionals, whether working in a clinical or management capacity, intended to be used in planning and strategy development, decision-making, and day-to-day management. It sets out policy, processes and procedures for the proactive management of risk in healthcare organisations, based on an enterprise approach (i.e. considers all risks, whether management or service delivery-related, together). This involves identifying and predicting uncertainties and threats, describing and analysing the level of the threat, responding where possible (e.g. through control measures), and reviewing and acting (e.g. communication and escalation regarding a threat).		
Research		**Aims**	**Methods**	**Findings**
Health Information and Quality Authority. Overview report of five years of HIQA monitoring in Irish public acute hospitals against national standards: 2015–2019.[82]	2020	To report key findings, examples of good practice and opportunities for improvement identified during monitoring of four national standards - infection prevention and control, medication safety, maternity services, and nutrition and hydration.	On-site inspections in hospitals between 2015-2019: infection prevention and control (n=161), medication safety (n=64), maternity services (n=22), and nutrition and hydration (n=5).	The greatest improvements were seen in relation to infection prevention and control and medication safety. However, there was variation between hospitals, affecting the quality of care provided. Factors such as insufficient resources hindered the implementation of the national standards.
**New Zealand**				
Policy				
Ministry of Health. Pae Ora (Healthy Futures) Act 2022.[85]	2022	The purpose of the Act is to provide for the public funding and provision of services in order to: (a) protect, promote, and improve the health of all New Zealanders; and (b) achieve equity in health outcomes among New Zealand's population groups, including striving to eliminate health disparities, in particular for Māori; and (c) build towards pae ora (healthy futures) for all New Zealanders.		

Simmonds S, Carter M, Haggie H, Mills V, Lyndon M, Tipene-Leach D. A Cultural Safety Training Plan for Vocational Medicine in Aotearoa. Te ORA and the Council of Medical Colleges.[86]	2023	Presents 'a plan for cultural safety training that medical colleges can employ in the development of their own cultural safety training programmes and in continuing professional development (CPD) with college fellows.'(p. 4) The plan is specific to the New Zealand context and 'focuses on cultural safety through the lens of Māori patients and their whānau.'(p. 4)
Standards New Zealand. NZS 8134:2021 Ngā Paerewa Health and disability services standard.[87]	2022	The standard promotes the safe provision of fair and equitable services for people and their whānau in New Zealand Aotearoa. It sets the foundations for promoting best practices and fostering continuous improvement in the quality (including safety) of health and disability services.
Te Tāhū Hauora Health Quality & Safety Commission. From knowledge to action: A framework for building quality and safety capability in the New Zealand health system.[88]	2016	'This document describes a high-level framework to guide the development of quality and safety capability across all levels in the health and disability sector'.(p. 4) It aims to address '…existing workforce needs, sustainably building the quality improvement capability of the future workforce, developing specialist roles in quality improvement science, supporting consumer/patient participation, ensuring decision-making is based on data and evidence, and supporting boards to provide leadership that encourages a quality improvement and patient safety focus throughout the sector.'(p. 4)
Te Tāhū Hauora Health Quality & Safety Commission. Clinical governance: guidance for health and disability providers.[89]	2017	Sets out a high-level framework for clinical governance in health and disability services. The values that underpin the framework are consumer wellbeing and safety. The guide is intended for use by all health and disability service providers, regardless of their size or complexity, and their staff, as well as other stakeholders.
Te Tāhū Hauora Health Quality & Safety Commission. Clinical governance framework: collaborating for quality (draft, November 2023).[90]	2023	A draft clinical governance framework to replace the clinical governance framework of 2017. Its purpose is '…to provide a comprehensive approach to enable the health sector to develop their own clinical governance infrastructure, adapted to their clinical context.' (p. 6) 'The framework is intended to support a culture of participatory leadership, where collaboration drives quality' and is '… deliberately inclusive of the health workforce, as opposed to being solely focussed on managers and clinicians, as it is applicable across the health sector and needs to be inclusive of all health environments.' The document also states that: 'The framework has been intentionally designed for the Aotearoa New Zealand context, to enact Te Tiriti o Waitangi [the founding document of Aotearoa New

		Zealand, signed by the Crown and many hapū and iwi leaders] and strive to achieve equity.'(p. 6)
Te Tāhū Hauora Health Quality & Safety Commission. Guide to doing a learning review.[91]	2023	To enable health providers to review harm by applying the learning review method, in which the reviewer aims 'to understand the realities of everyday work to uncover how harm occurs, minimise risk in the system and support health care workers to do the right thing'(p. 3), including identifying areas for improvement. This method is influenced by concepts such as human factors and resilient healthcare, and takes a systems approach to safety.
Te Tāhū Hauora Health Quality & Safety Commission. National adverse events reporting policy.[92]	2023	The purpose of the policy is to provide 'a consistent way to understand and improve through reporting, reviewing and learning from all types of harm.' (p. 5). It is intended to guide reporting to Te Tāhū Hauora [Health Quality and Safety Commission], and the use of information from learning reviews, and quality improvement approaches to strengthen system safety. The policy differs from the previous policy by shifting the focus to 'system safety' and restorative response in the context of partnership with consumers and whānau.
The National Collaborative for Restorative Initiatives in Health. He Maungarongo ki Ngā Iwi: Envisioning a restorative health system in Aotearoa New Zealand.[93]	2023	Provides an overview of the systems that mitigate and respond to healthcare harm in Aotearoa NZ, adopting a human-centred and relational approach. It presents a framework of principles and practices and defines restorative systems as being 'distinguished by an emphasis on relational principles, practices and goals that promote and restore human dignity and wellbeing.'(p. 10)

[a]The original NTA 8009 document was updated twice, in 2011 and 2014, before the most recent NEN 8009 (2018) version.

[*]In Dutch, accessed in translation.

Supplementary material 5: Search information

Medline via Ovid, 8/12/23:

1 (safety adj3 management adj3 system*1).ti,ab. (588)
2 (safety adj3 management adj3 program*).ti,ab. (93)
3 1 or 2 (670)
4 (patient*1 adj2 (safety or harm* or risk*)).ti,ab. (204300)
5 patient harm/ or patient safety/ (25896)
6 4 or 5 (219200)
7 ((safety or hazard* or risk or risks) adj2 (accountability or assess* or assurance* or commitment* or communicat* or control* or coordinat* or cultur* or education or framework* or guidance or implement* or improvement* or leadership* or management or measurement* or monitor* or plan or plans or planning or policy or policies or promot* or quality or regulat* or reporting or responsibilit* or strateg* or training)).ti,ab. (285563)
8 *Safety Management/ (13937)
9 7 or 8 (295644)
10 system*1.ab. /freq=2 (1032216)
11 system*1.ti. (682356)
12 10 or 11 (1448707)
13 6 and 9 and 12 (3003)
14 3 or 13 (3632)

Embase via Ovid, 9/2/2024

1 (safety adj3 management adj3 system*1).ti,ab. 924
2 (safety adj3 management adj3 program*).ti,ab. 128
3 1 or 2 1038
4 (patient*1 adj2 (safety or harm* or risk*)).ti,ab. 343333
5 patient safety/ or patient harm/ or patient risk/ 173057
6 4 or 5 469009
7 ((safety or hazard* or risk or risks) adj2 (cultur* or education or implement* or improvement* or management or measurement* or

monitor* or quality or regulat* or reporting or responsibilit* or strateg* or training)).ti,ab. 123921
8 System*1.ab. /freq=2 1290950
9 System*1.ti. 770274
10 8 or 9 1735239
11 6 and 7 and 10 3017
12 3 or 11 3986
13 limit 12 to conference abstract status 996
14 12 not 13 2957

CINAHL via EBSCOhost, 9/2/2024

#	Query	Results
S8	S1 OR S7	2,564
S7	S3 AND S4 AND S6	2,373
S6	S2 OR S5	107,816
S5	(MM "Patient Safety")	38,651
S4	TI (system or systems) OR AB (system or systems)	516,337
S3	TI (((safety or hazard* or risk or risks) N1 (cultur* or education or implement* or improvement* or management or measurement* or monitor* or quality or regulat* or reporting or responsibilit* or strateg* or training)).) OR AB (((safety or hazard* or risk or risks) N1 (cultur* or education or implement* or improvement* or management or measurement* or monitor* or quality or regulat* or reporting or responsibilit* or strateg* or training)).)	37,449
S2	TI ((patient* N1 (safety or harm* or risk*))) OR AB ((patient* N1 (safety or harm* or risk*)))	81,650

S1	TI safety N3 management N3 system* OR AB safety N3 management N3 system*	238

Web of Science: 10/2/2024

- WOS.SCI: 1900 to 2024
- WOS.AHCI: 1975 to 2024
- WOS.ESCI: 2015 to 2024
- WOS.ISTP: 1990 to 2024
- WOS.SSCI: 1900 to 2024
- WOS.ISSHP: 1990 to 2024

Searches:
1: TS=(safety near/1 management) AND TS=((management near/1 (system or systems))) Results:3363
2: TI=((patient* near/2 (safety or harm* or risk*))) Results: 61188
3: TS=(safety near/1 (cultur* or education or implement* or improvement* or management or measurement* or monitor* or quality or regulat* or reporting or responsibilit* or strateg* or training)) Results: 78695
4: TS=(hazard* near/1 (cultur* or education or implement* or improvement* or management or measurement* or monitor* or quality or regulat* or reporting or responsibilit* or strateg* or training)) Results: 8001
5: TS=(risk near/1 (cultur* or education or implement* or improvement* or management or measurement* or monitor* or quality or regulat* or reporting or responsibilit* or strateg* or training)) Results: 122887
6: TS=(risks near/1 (cultur* or education or implement* or improvement* or management or measurement* or monitor* or quality or regulat* or reporting or responsibilit* or strateg* or training)) Results: 122887
7: TS=(system or systems) Results: 11251037
8: #6 OR #5 OR #4 OR #3 Results: 205099
9: #7 AND #8 AND #2 Results: 1288
10: TS=(patient* or health* or hospital*) Results: 11662421
11: #10 AND #1 Results: 1116
12: #9 OR #11 Results: 2379

de Bell S, *et al. BMJ Open* 2026; 16:e107772. doi: 10.1136/bmjopen-2025-107772

Details of website searches of patient safety organisations in included countries

Organisation	URL	Search date		No. of hits	No. of includes
**Australia**					
Australian Commission on Safety and Quality in Health Care	https://www.safetyandquality.gov.au/	01/12/2023	safety management system	141	5
Department of Health and Aged Care	https://www.health.gov.au/	06/12/2023	safety management system	171	0
Australian Institute of Health and Welfare	https://www.achs.org.au/	06/12/2023	safety	63	0
Australian Council on Healthcare Standards	https://www.achs.org.au/	06/12/2023	safety	43	0
**Canada**					
Healthcare Excellence Canada	https://www.healthcareexcellence.ca/en	05/01/2024	safety	406 (200 screened)	4

Institute for Safe Medication Practices (ISMP) Canada	https://ismpcanada.ca	05/01/2024	safety management system	11	0
**Ireland**					
Health Information and Quality Authority (HIQA)	https://www.hiqa.ie	01/02/2024	Publications - safety	104	4
National Patient Safety Office, Ireland	https://www.gov.ie/en/campaigns/af4b6-national-patient-safety-office/	01/02/2024	safety	44	0
National Quality and Patient Safety Directorate	https://www2.healthservice.hse.ie/organisation/nqpsd/	01/02/2024	n/a	No search function	3
**Netherlands**					
NIVEL (The Netherlands Institute for Health Services Research)	https://www.nivel.nl/en	01/02/2024	safety management	76	2
Dutch Patient Safety Programme	https://www.vmszorg.nl/	05/12/2023		In Dutch	2
Netherlands Health and Youth Care Inspectorate, Ministry of Health Welfare and Sports	https://english.igj.nl	05/12/2023	safety	56	0
**New Zealand**					

Te Tāhū Hauora Health Quality and Safety Commission	https://www.hqsc.govt.nz/	01/02/2024	Resource library - safety management system	115	9

de Bell S, *et al. BMJ Open* 2026; 16:e107772. doi: 10.1136/bmjopen-2025-107772
