# Supplementary material for: Implementation of safety management systems (SMS) in healthcare: a systematic review and international comparison
Source: BMJ Open. 2026 May 12;16(5):e107772. doi: 10.1136/bmjopen-2025-107772 (PMC13223942; doi:10.1136/bmjopen-2025-107772)
Supplement: Author's manuscript [file bmjopen-2025-107772.draft_revisions.pdf]

BMJ Open is committed to open peer review. As part of this commitment we make the peer review history of every article we publish publicly available.

When an article is published we post the peer reviewers' comments and the authors' responses online. We also post the versions of the paper that were used during peer review. These are the versions that the peer review comments apply to.

The versions of the paper that follow are the versions that were submitted during the peer review process. They are not the versions of record or the final published versions. They should not be cited or distributed as the published version of this manuscript.

BMJ Open is an open access journal and the full, final, typeset and author-corrected version of record of the manuscript is available on our site with no access controls, subscription charges or pay-per-view fees (http://bmjopen.bmj.com).

If you have any questions on BMJ Open's open peer review process please email info.bmjopen@bmj.com

BMJ Open

The implementation of Safety Management Systems (SMS) in healthcare: a systematic review and international comparison

Journal:	*BMJ Open*
Manuscript ID	bmjopen-2025-107772
Article Type:	Original research
Date Submitted by the Author:	10-Jul-2025

Complete List of Authors:	de Bell, Siân ; University of Exeter, Exeter HS&DR Evidence Synthesis Centre, Department of Health & Community Sciences<br>Zhelev, Zhivko; University of Exeter, Exeter HS&DR Evidence Synthesis Centre, Department of Health & Community Sciences<br>Bethel, Alison; University of Exeter, Exeter HS&DR Evidence Synthesis Centre, Department of Health & Community Sciences<br>Clarke, Maria; University of Exeter, Exeter HS&DR Evidence Synthesis Centre, Department of Health & Community Sciences<br>Anderson, Rob; University of Exeter, Exeter HS&DR Evidence Synthesis Centre, Department of Health & Community Sciences<br>Thompson Coon, Jo; University of Exeter, Exeter HS&DR Evidence Synthesis Centre, Department of Health & Community Sciences

Keywords:	Systematic Review, Safety, Organisation of health services < HEALTH SERVICES ADMINISTRATION & MANAGEMENT

SCHOLARONE™
Manuscripts

I, the Submitting Author has the right to grant and does grant on behalf of all authors of the Work (as defined in the below author licence), an exclusive licence and/or a non-exclusive licence for contributions from authors who are: i) UK Crown employees; ii) where BMJ has agreed a CC-BY licence shall apply, and/or iii) in accordance with the terms applicable for US Federal Government officers or employees acting as part of their official duties; on a worldwide, perpetual, irrevocable, royalty-free basis to BMJ Publishing Group Ltd ("BMJ") its licensees and where the relevant Journal is co-owned by BMJ to the co-owners of the Journal, to publish the Work in this journal and any other BMJ products and to exploit all rights, as set out in our licence.

The Submitting Author accepts and understands that any supply made under these terms is made by BMJ to the Submitting Author unless you are acting as an employee on behalf of your employer or a postgraduate student of an affiliated institution which is paying any applicable article publishing charge ("APC") for Open Access articles. Where the Submitting Author wishes to make the Work available on an Open Access basis (and intends to pay the relevant APC), the terms of reuse of such Open Access shall be governed by a Creative Commons licence – details of these licences and which Creative Commons licence will apply to this Work are set out in our licence referred to above.

Other than as permitted in any relevant BMJ Author's Self Archiving Policies, I confirm this Work has not been accepted for publication elsewhere, is not being considered for publication elsewhere and does not duplicate material already published. I confirm all authors consent to publication of this Work and authorise the granting of this licence.

For peer review only

The implementation of Safety Management Systems (SMS) in healthcare: a systematic review and international comparison

Siân de Bell[1* a], Zhivko Zhelev[1*], Alison Bethel[1], Maria Clarke[1], Rob Anderson[1], Jo Thompson Coon[1]

[1]Exeter HS&DR Evidence Synthesis Centre, Department of Health & Community Sciences, University of Exeter Medical School, University of Exeter, Exeter, UK

*Joint first author

[a] Corresponding author: Sian de Bell s.c.de-bell@exeter.ac.uk

For peer review only

For peer review only - http://bmjopen.bmj.com/site/about/guidelines.xhtml

Abstract

Background

Many industries where safety is a priority (e.g. aviation) use safety management systems (SMSs) but evidence on their use in healthcare is needed to determine whether they could support patient safety improvement.

Objective

To investigate the application of national-level SMSs to patient safety in terms of effectiveness, implementation and experience.

Design: We conducted a systematic review of evidence from five high-income countries that have publicly funded healthcare systems with universal coverage: Australia, Canada, Ireland, New Zealand and the Netherlands.

Methods

We identified patient safety organisations in each country and searched their websites. We also searched MEDLINE (in December 2023) and Embase (via Ovid), CINAHL (EBSCO) and Web of Science (in February 2024). We included any evidence on the effectiveness, implementation or experience of the safety approach in each country.

Included evidence was summarised and mapped onto an initial analytical framework based on analysis of SMSs in high-risk industries, enabling cross-country comparisons. Drafts were shared with experts from patient safety organisations in each country for comment. Formal quality appraisal was not possible as most evidence was non-evaluative.

Results

Fifty-three publications were included, from Australia (5), Canada (7), Ireland (8), New Zealand (9), and the Netherlands (24). Only the Netherlands implemented a patient safety programme explicitly based on an SMS approach. Some aspects of patient safety in hospitals improved as a result but there was significant variation in both the implementation of the programme and attributed outcomes.

In the other four countries, the main components of an SMS were identified to some extent, along with evidence that their patient safety approaches had been influenced by concepts from high-risk industries and 'safety science' more widely.

Conclusions

Emerging ideas from high-risk industries (beyond SMSs) and broader safety science have influenced all countries, reflecting increasing awareness of the need for initiatives to be context-specific to be successful. However, their implementation and impact need further evaluation.

Registration: CRD42023487512

Key words
safety management systems; patient safety; healthcare; systematic review

Strength and limitations

- There is evidence that safety management systems have been effective in improving safety in high-risk industries (e.g. aviation); this is the first systematic review

For peer review only

investigating whether and how the approach has been used to improve patient safety in healthcare.

- We included a broad evidence base, searching the websites of patient safety organisations alongside searching for peer-reviewed articles, and consulting safety experts in included health systems to verify our understanding of their approach to safety management in healthcare.
- We focused on high-income countries with healthcare systems comparable to the UK. However, we were only able to include those where evidence (e.g. policy documents) was available in English.
- Our analysis was based mainly on non-evaluative documents, so we were not able to conduct quality appraisal or make firm conclusions about the impacts of adopting a safety management system approach in healthcare.

1. Background

All healthcare systems work in various ways to improve patient safety, from creating policies at national and organisational levels to implementing interventions as a result. However, patient safety improvement is not an easy process; for example, a national review of patient safety in England in 2024 found that, compared to the previous review in 2022, performance had worsened in a number of areas (e.g. maternity care).[1] Patients continue to be harmed by failures or errors, with examples of safety incidents including mistakes associated with the use of medication, such as in prescribing or dispensing, and wrong site surgery.[2] It was estimated in 2020 that 237 million medical errors occur in England each year, contributing to more than 1,700 deaths.[3]

Safety is also a significant priority in other industries - such as aviation, nuclear energy and oil and gas - where errors could have serious consequences for people or the environment.[2] Many of these 'safety-critical' industries, and companies within them, have improved safety by taking a systems approach: setting international or national policies and standards, with organisations operationalising these in an organised approach to managing safety known as a safety management system (SMS).[4] An SMS is generally considered to have four key components:

- Leadership commitment and safety policy – an expressed leadership commitment to safety with documentation of responsibilities and processes for safety within an organisation,[2, 5]
- Safety risk management – the identification of hazards and risk, and assessment of how to mitigate these,[2]
- Safety assurance – monitoring and measuring safety within the organisation and ensuring continuous improvement,[2]
- Safety promotion and culture – training, education and communication of safety to staff at all levels.[6]

The underlying theory behind SMSs is that by focusing on performance - monitoring and achieving desired outcomes - rather than solely on compliance, they facilitate improvement.[2] In 2012, a systematic review of SMSs in three safety-critical industries – aviation, marine and rail – concluded that the approach led to improvements in safety (e.g. reduced accident rates).[7]

The basic components of an SMS are quite generic and transferrable.[8] However, in order to be effective, the specifics need to be adapted to the context in which they are being used, as can be seen in SMSs in other high-risk industries.[9] For example, the aviation, nuclear, and oil and gas industries all have international guidelines regarding safety management.[5, 6, 10] In

For peer review only - http://bmjopen.bmj.com/site/about/guidelines.xhtml

aviation, these are used by countries to develop state safety programmes with detailed requirements for safety management by individual organisations. Nuclear safety is considered a national policy issue, with states responsible for the safety of their own nuclear facilities. The oil and gas industry differs from both in that it identifies and manages safety risks within the context of achieving performance goals and stakeholder benefits.[10] In all these industries, individual organisations implement policy and guidance according to their own ways of working. In this review, we focus on the high-level influence or coordination of the implementation of an SMS approach, rather than the operation of organisation-level SMSs.

1.2 Objectives

This systematic review investigated the application of SMSs to patient safety in healthcare, to consider whether and how they could be implemented more widely, asking:

- How are the components of an SMS reflected in the healthcare policy documents of the included countries?
- What research or other relevant evidence is available regarding the effectiveness, implementation or experience of SMSs within healthcare?
- What does evidence from beyond the UK tell us about the effectiveness, implementation or experience of SMSs within healthcare?

This review was commissioned by the National Institute for Health and Care Research (NIHR) Health Service Delivery Research (HSDR) programme on behalf of the NHS England Patient Safety Team and Department of Health & Social Care, to inform the further development of the NHS England's patient safety policy and practice. The UK was not included in the review as the intention was to learn from the experiences of other countries, with different starting points in terms of the organisation and management of healthcare, and different pre-existing (safety) system components, regarding their patient safety approaches.

2. Methods

This systematic review was registered on PROSPERO (CRD42023487512) and can be found in Supplementary Material 1. A summary of the methods is provided below; further detail can be found in the full report.[11]

2.1 Inclusion and exclusion criteria

Evidence from the following countries was eligible for inclusion in the review: Ireland, Australia, New Zealand, Canada and the Netherlands. These countries were selected as they are high-income countries[12] with healthcare systems comparable to that of the UK, and scoping searches had provided indicative evidence of the adoption of an SMS approach, or some of its key components, to improve patient safety.

As this is an emerging, policy-relevant topic, we included a broad range of types of evidence[13]:

- Policy documents, including those related to the implementation of an SMS,
- Published or unpublished research / evaluations (quantitative, qualitative or mixed methods),
- Other evidence (e.g. patient and staff experience from organisations' websites).

Evidence relating to any service user population and any healthcare setting were included in the review; study participants could include healthcare professionals, service users, or carers. In terms of focus, we included evidence on any policies, initiatives, or programmes

For peer review only - http://bmjopen.bmj.com/site/about/guidelines.xhtml

related to the key components of an SMS (as defined above). See Supplementary Material 2 for more information.

2.2 Search strategy and study selection

MEDLINE (from 1946) was searched in December 2023 and Embase (from 1974) (via Ovid), CINAHL Ultimate (from 1937) (EBSCO) and Web of Science (WoS): Science Citation Index (from 1990), Social Science Citation Index (from 1900), Arts and Humanities Index (from 1975), Conference Proceedings, Science, Social Science and Humanities (from 1990), Emerging Sources Citation Index (from 2015) in February 2024; (see search strategies in Supplementary Material 3. In March 2024, forwards citation searching was undertaken in Web of Science using eight papers (from the Netherlands, as the only country explicitly using an SMS approach). The reference lists of all publications that met our inclusion criteria were checked for relevant publications.

Key policy documents and evidence that might not be captured through database searches (e.g. programme reports) were identified by searching the websites of, and contacting, national patient safety organisations from each of the included countries (Supplementary Material 3). To understand the context within each country and identify further sources of evidence, we also held online meetings with patient safety/health policy experts from:

- Australia - Australian Commission on Safety and Quality in Health Care; Department of Health and Aged Care (federal government)
- Canada - Healthcare Excellence Canada
- Ireland - Health Information and Quality Authority (HIQA); Royal College of Surgeons Ireland
- Netherlands - Netherlands Institute for Health Services Research (NIVEL)
- New Zealand - Health Quality and Safety Commission

All results from the database searching were independently screened by two reviewers (SDB, ZZ). The full text of papers which were judged to meet the inclusion criteria were obtained, along with policy and other documents identified by web-searching. Two reviewers (SDB, ZZ) assessed each full text independently for inclusion. At both stages, disagreements were settled through discussion.

2.3 Data extraction and synthesis

Whilst creating the review protocol and searching for studies, we developed an analytical framework of the components of an SMS by reviewing policy documents and review articles from high-risk industries (focusing on aviation,[6] oil and gas[10] and nuclear energy[5]). Data extraction and thematic analysis of evidence from the healthcare systems of each included country was guided by this framework. A case study approach was taken, with the patient safety approach in each country described and analysed separately, and draft descriptions for each country shared with experts from patient safety organisations in the respective countries for correction or comment. In the final step of the analysis, we compared the experience and implementation of the SMS or safety approach in healthcare across countries. Included publications were not quality appraised as the majority were not empirical studies so quality appraisal would not have made a significant contribution to our understanding of the application of SMSs in healthcare. We did consider uncertainties in the evidence, stemming from methodological limitations, when reporting on the Netherlands (as the only country that had explicitly used an SMS approach).

2.3 Public and patient involvement and engagement

Due to the focus of review, we sought public collaborators who had some knowledge of the topic. We held meetings with three public collaborators, two of whom had knowledge of

For peer review only - http://bmjopen.bmj.com/site/about/guidelines.xhtml

safety management in other industries (chemical and police/counter-terrorism) and one who had experienced a safety incident in the NHS and has been campaigning to raise awareness of patient safety. In these meetings, we discussed their experiences of safety in other industries (where relevant) and of patient safety in the NHS, as well as their views on the improvement of patient safety. Our public collaborators provided important contextual information on safety management and SMS in other industries (e.g. the importance of clear accountability for safety) and their potential use in the NHS. They also informed specific aspects of the review (e.g. discussions of their own safety experiences in the NHS highlighted areas to consider in our data extraction and analysis, such as approaches to involving patients in safety policy and incident investigations and applying learning from safety incidents).

3. Results

Database searches resulted in 11,532 hits; 6,913 records were screened at title and abstract level. Of those, 158 were selected for full text screening and 89 were found to meet the inclusion criteria. Searches of websites resulted in 32 publications being included in the review, along with 19 documents from other sources (11 from the reference lists of included documents and nine from expert suggestions). This resulted in a total of 140 studies being included in the review. Further details can be found in Figure 1 and Supplementary Material 4.

Figure 1 PRISMA diagram of the screening process [figure uploaded separately]

Due to the quantity of evidence found, we grouped publications into two broad categories (Table 1):

- Descriptions of each country's approach to patient safety, including influences from high-risk industries (mainly policy documents but also outputs e.g. opinion papers).
- Evidence on the impact and implementation of a specific patient safety approach, or aspects of it (mainly research papers and project reports). These were categorised further as:
    - 'rich' – reporting on the impact or implementation of the approach, or an essential part of it, and
    - 'thin' – papers with a very narrow focus and limited relevance to our review question (e.g. the accuracy or acceptability of a risk assessment tool implemented as part of a patient safety programme); such studies were included to illustrate the way in which a programme was designed or implemented, and not because of their specific results.

Table 1 Number of evidence sources per category per country.

Country	'Rich' documents (n=53)			'Thin' documents (n=87)
	Policy	Research	Other*	
Netherlands	2	20	2	21
Australia	3	2	0	37[a]
Canada	5	2	0	25[a]

For peer review only

Ireland	7	1	0	2
New Zealand	9	0	0	6[a]

*Such as opinion papers.

[a]Some included evidence had information from multiple countries (Australia and Canada (n=2) and Australia and New Zealand (n=2)) so has been included in multiple categories.

Our analysis was based primarily on the descriptive and rich evidence. We focused on 53 publications, which can be found in Supplementary Material 5, from Australia (5), Canada (7), Ireland (8), New Zealand (9), and the Netherlands (24) (Table 1). Some 'thin' evidence is used to illustrate specific points (see full list in Supplementary Material 4). Below, we describe the patient safety approach in each country then compare these approaches.

3.1 Netherlands

The Netherlands was the only country to explicitly use an SMS approach, in the form of its national patient safety programme (PSP), which was launched in 2008 and ran until the end of 2012.[14] The programme was implemented in public hospitals and based on the premise that change in the structure or processes of an organisation is necessary to see a change in outcomes.[15] It had two pillars: (i) hospitals were required to implement a certified SMS, and (ii) a set of guidelines focusing on improving patient safety for ten priority themes.[16] The aim was a 50% reduction in adverse events and potentially preventable deaths over a period of 5 years.[16] The two pillars of the PSP continued to be implemented after 2012, with development and revisions based on evaluations of its success. A new safety programme, based on a Safety-II perspective, began in 2020.[17]

The basic SMS requirements were agreed by key stakeholders and published in 2007 (NTA 8009).[18] The complexity of implementing an SMS was acknowledged, with healthcare providers required to carry this out in stages. In Phase 1 the basic requirements of an SMS were developed (leadership, management, personnel, patient participation, prospective risk assessment, retrospective risk assessment and improvements), with further requirements added in Phase 2 (communication, third-party management, and control measures).[18, 19] The latest version of the document specifying these requirements, NEN 8009, was published in 2019; it introduces a new perspective on patient safety based on Safety II and resilience engineering (see Glossary). These concepts emphasise the importance of learning from successful, 'incident-free' practice as well as from the investigation and prevention of incidents.[19]

There were ten high-priority themes for patient safety, such as patient mix-up (see Glossary). Expert groups were convened for each theme, and guides were developed describing structure, processes, and outcomes. Interventions to improve patient safety were explained during national theme conferences. The programme also had a website where resources and patient-facing materials were available. Whilst the initial focus was on adult care, six of the patient safety themes were later expanded to include paediatric hospital care.

3.2 Australia

In Australia, patient safety is primarily a national (i.e. federal government) responsibility.[20] However, state and territory governments are responsible for their own public hospital systems, and the licencing and regulation of the private hospital sector, as recognised in the National Health Reform Act 2011 (NHRA - which specifies responsibilities for funding,

For peer review only - http://bmjopen.bmj.com/site/about/guidelines.xhtml

operating, managing and regulating the health system).[20] The systems and processes used to ensure safety therefore differ between territories and states.[20]

The Australian Commission for Safety and Quality in Health Care developed the National Safety and Quality Health Service (NSQHS) Standards with the aim of ensuring the relevant systems are in place within a healthcare organisation to ensure quality of care.[21] The first edition was published in 2012, the latest in 2021.[21] The NSQHS Standards detail requirements for healthcare organisations regarding clinical governance and partnering with consumers, as well as specific areas of clinical management. They require providers to have an organisation-wide clinical incident management and investigation system, with an open disclosure policy consistent with the Australian Open Disclosure Framework.[20] Certain indicators which should be measured, such as sentinel events, have been developed by the Commission, and the Australian Health Performance Framework details a common set of metrics for public reporting of safety and quality data.

Australia has not used an SMS approach in its healthcare system, although "*the healthcare sector began to draw on lessons from other high risk industries*" such as aviation and mining, as well as using human factors analysis, to develop its patient safety approach.[20] These concepts from high-risk industries have influenced specific elements of Australia's approach (e.g. surgical checklists).

3.3 Canada

The responsibility for delivering health services lies with the provincial and territorial governments, leading to variation in how patient safety is approached in Canada. The Canadian Quality and Patient Safety (CPQS) Framework was launched in 2020 and provides guidance to drive patient safety and quality improvement in all healthcare sectors and settings. Whilst it is intended to align Canadian legislation, regulations, standards, organizational policies, and public engagement nationally,[22] there is currently variation between governments. Not all jurisdictions have quality and safety plans or policies regarding key elements of patient safety.[23]

Healthcare Excellence Canada, an independent non-profit organisation funded primarily by Health Canada - a department of the federal government – has produced a discussion guide that details the recent change in Canada's approach to patient safety. There has been a move from 'traditional' patient safety improvement (e.g. scorecards and audits), to a focus on creating a proactive and inclusive environment, with a culture and processes that support continuous safety improvement.[24] Canada has drawn on learning from high-risk industries, with the Measurement and Monitoring of Safety Framework (MMSF) underpinning its new approach to patient safety but has not taken an SMS approach.[25]

3.4 Ireland

The Health Information and Quality Authority developed the National Standards for Safer Better Healthcare in 2012; these detail expectations for healthcare, and guide improvement of quality, safety, and reliability.[26] They were replaced by a principles-based approach in 2021.[27] However, the Health Service Executive has developed a national patient safety strategy which builds on the Standards, to be implemented and guide improvement at a local level.[28]

Irish healthcare providers are required to have an incident reporting system.[29] Incidents are entered into the national system and reviewed externally or internally depending on the severity of the incident.[29, 30] Providers are also expected to undertake clinical and non-clinical audits (which may be part of national or local programmes), implement improvements based on these,[28, 31] and assess and manage risks, with a formal process in place to do this.[32]

For peer review only - http://bmjopen.bmj.com/site/about/guidelines.xhtml

Sláintecare, an improvement plan and strategy intended to reform and develop health services in Ireland over the next decade and into the future, is currently being implemented in Ireland; the provision of healthcare, including the improvement of safety, is consequently evolving.[28]

Whilst Ireland has not taken an SMS approach, the influence of high-risk industries can be seen in various elements of their approach. The national patient safety strategy details an action regarding information and training for staff drawing on "*patient safety and reliability science, systems thinking, audit, quality improvement methodologies, change management, human factors and multidisciplinary team working for safety*",(p.13)[28] with an example of this being the delivery of simulation-based education and human factors training to improve patient safety (see Glossary).[33, 34]

3.5 New Zealand

Patient safety in New Zealand is a central focus of the healthcare system, with the Te Tāhū Hauora Health Quality & Safety Commission (Te Tāhū Hauora), established in 2010, playing a pivotal role in improving healthcare quality and safety across the country.[35] The Pae Ora (Healthy Futures) Act 2022 replaced the country's 20 District Health Boards, aiming to create a more centralised, effective and efficient healthcare system.[35] The Act[35] and the updated Nga Paerewa Standard Health and disability services standard[36] are associated with important changes in the New Zealand's patient safety policy, such as the 'Healing, Learning, and Improving from Harm: National Adverse Events Policy'[37] which took effect from 1st July 2023 and the new 'Clinical Governance Framework: Collaborating for quality' (which is still in a process of consultation).[38]

The new framework has been intentionally designed for the cultural context of New Zealand, with service users and their families seen as 'partners in care'.[38] Elements of safety are emphasised, such as the importance of having processes and systems in place to ensure quality and safety (e.g. clinical risk management systems to deal with non-compliance with clinical standards and policies), and of focusing on 'system safety' – managing risk within a systems perspective – and learning how the healthcare system shapes the experiences of consumers and staff.[38] The MMSF is suggested as a tool that could help decision makers to assess the overall safety of their organisation. To assess the quality of the health and disability system, Te Tāhū Hauora has adopted a range of baseline measures and indicators:

- Atlas of Healthcare Variation
- Quality and Safety Markers (QSMs)
- Quality Accounts
- Health Quality and Safety Indicators (HQSIs)
- Prevention quality indicators.

The current (2023) version of the clinical governance framework (in consultation) includes a number of ideas (e.g. systems safety, resilient organisations) which are linked to developments in high-risk industries.[38, 39] For example, the 'learning review' method was originally developed for the investigation of firefighter incidents in the context of the US Forest Service and draws on a number of concepts, such as human factors and ergonomics, systems safety and resilient organisations. It was specifically developed for 'adaptive complex systems' – complex systems with high level of uncertainty that interact, learn and adapt over time – such as healthcare.[39]

For peer review only - http://bmjopen.bmj.com/site/about/guidelines.xhtml

3.6 Comparison

Although the Netherlands was the only country to explicitly use an SMS approach, the main components of an SMS were identified, to varying extents, in the patient safety policies and initiatives of the other four countries, as described in Tables 2-4. Whilst the interdependence of these elements was acknowledged, they were often detailed in different policies and within the scope of different organisations. Key features that were seen in all five countries included:

- Leadership commitment to patient safety.
- National patient safety policy (separately or in some cases an aspect of wider quality improvement policy).
- Retrospective incident reporting and analysis linked to system for feedback and learning to ensure continuous improvement. Methods for categorising events (e.g. actual harm vs near misses) and their severity (high- and low-) were similar across countries and determined whether and how they were investigated (e.g. central vs local, such as in the Netherlands and New Zealand). Investigation differed (e.g. learning review and restorative practice in New Zealand, root cause analysis in the Netherlands and Australia, see Glossary).
- Prospective risk management, with risks identified using multiple sources of evidence (e.g. local, national, and international research). Countries had specific standards for high-priority patient safety topics (e.g. patient identity mix-up) as well as general guidelines.
- Monitoring of patient safety performance. Whilst systems for monitoring at the local level were less clear, all countries had a national system.
- Focus on developing a safety culture (including open communication and blame-free reporting).
- Patients' and families' involvement, both in the development of patient safety policies and safety at the local level.

For peer review only - http://bmjopen.bmj.com/site/about/guidelines.xhtml

Table 2 Comparison of the scope of the SMS or safety approach in each country

	Netherlands	Australia	Canada	Ireland	New Zealand
**Geographical**	National coverage.	National standards; state and territory governments are responsible for their own healthcare systems.	National framework; provincial and territorial governments are responsible for their own healthcare systems.	Standards and patient safety strategy are set at the national level, but implementation is local to allow for adaptation to context.	Transition from regional to national coverage.
**Organisational**	Hospital care.	All healthcare settings.	All healthcare settings.	Health and social care.	All healthcare settings; health and disability care.
**Overlap with quality management/systems**	SMS separate from the quality management and occupational health and safety systems.	Quality and safety are considered together.	Quality and safety are considered together.	Safety is considered part of other systems in the organisation (e.g. quality improvement).	Quality and safety are considered together.
**Extent of accountability for 'other' organisations**	Third party management (outsiders performing activities in hospital) included.	-	-	There should be formalised agreements in place for the provision of services provided externally, including quality and safety.	-

Table 3 Comparison of key features of the SMS or safety approach in each country relating to leadership commitment and safety policy and safety risk management

	Netherlands	Australia	Canada	Ireland	New Zealand
**Leadership commitment and safety policy**	BoD formulates multi-year safety policy and safety plan using SMART-formulated objectives; provides resources (funds, time, expertise, training etc); reviews and evaluates impact and promotes safety culture;	The Standards state that the governing body should provide leadership, set the strategic direction (including a framework with processes to drive improvement), and monitor and review safety and quality.	*"Board members are accountable for safety and for ensuring patient safety concerns and incidents are acted upon"*(p.42);[22] this includes quality and safety goals.	The national standards set out the need for "*A commitment to quality and safety articulated and demonstrated by those governing and leading the service*".(p.72)[26] There should be an identified	Leadership is one of the system drivers in the draft Clinical Governance framework. It enables the health workforce to be active members of the learning organisation by creating a culture of psychological safety and

	management translates the above into their own area of responsibility and reports to BoD.<br>All employees have responsibility and are encouraged to participate in improving safety.<br>Patient safety is included in the hospital's performance system	The governing body of the organisation should also ensure "*that roles and responsibilities are clearly defined for the governing body, management, clinicians and the workforce*"(p.6)[21] in relation to quality and safety, and that clinicians understand their role in patient safety.	Health leaders should encourage and support health teams to improve patient safety, whilst health workers should take an active role in creating a safe environment.	individual with accountability and responsibility for safety, as well as a commitment to safety from leaders at all levels.<br><br>The national patient safety strategy will "*put in place integrated governance structures with clear accountability for planning, managing and addressing the above patient safety priorities.*"(p.17)[28] i.e. boards, management, staff and relevant quality and safety committees within an organisation should all have defined responsibilities for safety.	trust. Organisations need to share leadership by valuing knowledge and expertise drawn from lived experience of consumer and whānau equally alongside clinical and other knowledge.<br>The National Adverse Events Policy states that health and disability service providers are accountable for ensuring the systems they implement recognise various degrees and types of harm; meet the national expectations for reporting, healing, learning and improving; involve consumers and whanau; and create local process to operationalise the policy.
**Safety risk management**[a]	Prospective risk assessment carried out by multi-disciplinary teams using accepted methods (e.g. failure modes, effects, and criticality analysis, FMECA), provided with sufficient time and resources; regularly reviewed.<br><br>Operational control measures: patient safety is integrated in daily business operations; management of change is an aspect of this.	Providers should identify and documents risks (including external and internal disasters and emergencies); take actions to reduce them; regularly review actions, and report on identified risks to staff and patients.	-	The national patient safety strategy details the need to "*proactively identify areas of risk to patient safety*"(p.15),[28] as do the Standards. Learning should also be taken from where things have gone wrong.<br><br>Governance for risk should be integrated into an organisation's management processes.	In the draft Clinical Governance framework, the focus is on system safety and 'learning together' culture. It suggests using the MMSF and actively seeking organisational data that challenges the sense that 'all is well'. A broad range of data sources are listed including quality monitoring, 'soft intelligence', operational data, health workforce and consumer and whanau experience.

For peer review only - http://bmjopen.bmj.com/site/about/guidelines.xhtml

	Focus on ten priority themes.	The NSQHS Standards (6 of which relate to high-risk areas of patient care) and clinical care standards.		Clinical standards.	Clinical standards.
[a] Including risk related to planned changes					

Table 4 Comparison of key features of the SMS or safety approach in each country relating to safety assurance and safety promotion and culture

	Netherlands	Australia	Canada	Ireland	New Zealand
**Safety assurance**[b]	Management is responsible for monitoring patient safety performance in the hospital and reporting of results (e.g. to BoD) at least annually.	Organisations should have quality improvement systems that identify, monitor and report on safety performance and outcomes	*"Ensure that avoidable death and hospital standardized mortality ratio (HSMR) data is tracked and reported so action can be taken to improve outcomes"*(p.34).[22]	Monitoring against National Standards for Safer Better Healthcare by HIQA, using national indicators where possible.	Monitoring against Te Tāhū Hauora's health quality and safety indicators. The Quality and Safety Measures are a mix of process and outcome measures, and focus on key safety priorities: falls, healthcare associated infections, surgical harms and medication safety.
	Using a multi-disciplinary approach, improvement measures are implemented and their impact assessed.	Organisations should identify areas for improvement and Implement and monitor improvement strategies.	*"Support an audit and feedback system to ensure that recommendations to improve patient safety are implemented"*(p.33).[22]	Requirement for annual audits, national and local. Learning from both incident reporting and monitoring should be used to improve quality and safety.	Continuous learning from adverse events is based on open reporting, investigation and meaningful analysis. Changes are implemented based on the learning process.
	Incident reporting and investigation				
	Requires safe, efficient and effective reporting processes and a system using accepted methods and pre-specified incident categories (reporting limited to hospital and to BoD). The information is used to improve safety by	NSQHS Standards require an organisation-wide clinical incident management and investigation system.	Health leaders should enable health teams to "*Report, learn from, and act on patient safety concerns and incidents*" (p.24)[22] by ensuring policies and reporting mechanisms are in place.	The Standards detail the need for "*Arrangements to identify, manage, respond to and report patient-safety incidents in a timely manner*".(p.66)[26] Incidents need to be entered on a national system, whether they are investigated internally or	The National Adverse Events policy 2023 requires serious adverse events to be reported to and investigated by the Te Tāhū Hauora. Less serious events are reported and managed locally.

For peer review only - http://bmjopen.bmj.com/site/about/guidelines.xhtml

	determining tasks, powers and responsibilities.		Organisational incident reporting and reporting to national systems for certain incidents.	externally depends on severity of incident.	
	Root cause analysis was recommended as an Investigation method initially.[18] NEN8009 (2018) does not recommend a method.[19]	Investigation method is root cause analysis.	-	Investigation method based on systems analysis.[29]	Investigation method is learning review.
	Staff involved in an incident are offered aftercare.	-	Health leaders and boards should ensure that health teams have access to psychological support.	Support (including psychological) for staff involved in a serious incident.	A theme in The National Adverse events policy 2023 'healing': providing emotional and practical support to affected individuals, families and healthcare workers.
Safety promotion and culture[c]	BoD responsible for promoting safety culture, including safe reporting.<br><br>Open communication in all directions.	Organisations should provide '*timely reports on safety and quality systems and performance*'(p.7)[21] to employees at all levels of the organisation, the public, and other healthcare organisations.	Recent move to create a proactive environment, with a culture and processes that support continuous safety improvement (e.g. safety huddles and other methods of open communication).	Systems for staff communication and engagement within organisations as well as with external agencies (e.g. HIQA) and support for programmes to promote a patient safety culture.	Cultural safety; integration of Maori worldview and safety science.
	BoD and management are responsible for providing training and monitoring whether staff need further training.	The organisation should provide access to training and monitor participation.	Health teams have a responsibility to undertake training and leaders and boards to provide training.	The Strategy identifies the importance *"designing and delivering safety information and training"* (p.13)[28] for staff.	Staff should be provided with learning opportunities.
	Patient participation in drawing up and implementing safety policy and reporting incidents, complaints and claims.	Patient participation in "*healthcare governance planning, design, measurement and evaluation*"(p.19)[21]	Patient participation in patient safety (e.g. through incident reporting and as public representatives in policy discussions).	Organisations should "*develop mechanisms to empower patients to contribute*"(p.11),[28] including to governance	*"Organisational clinical priorities, processes and evaluations are co-designed and developed collectively"*(p.11)[40] with patients.

For peer review only

				structures and reporting incidents.	

[b] Including accreditation, monitoring and reporting of outcomes as well as systematic implementation of improvement measures and evaluation of their impact, [c] Including patient and family involvement. Abbreviations: BoD – Board of Directors

For peer review only

For peer review only - http://bmjopen.bmj.com/site/about/guidelines.xhtml

There were, however, differences in the implementation of these common features between countries (e.g. incident investigation as described above and see Table 3). There were also other areas of variation, particularly relating to the scope of the patient safety approach (Table 2). In most cases, patient safety was considered alongside healthcare quality improvement, although in the Netherlands it was separate. Implementation of policies varied from national to regional, and their application to all or specific healthcare settings (e.g. hospital care only in the Netherlands, or all care settings in the other four countries). Additionally, a range of concepts from high-risk industries and safety science more broadly (e.g. Safety II, human factors, resilience engineering) had influenced the patient safety approaches of different countries.

All countries considered inequalities to some extent by targeting groups particularly at risk of unsafe care (e.g. older people). The most specific guidance was found in Australia, Canada and New Zealand. In these countries groups were identified in terms of social (e.g. those living in deprivation), ethnic (e.g. Māori and Pacific peoples in New Zealand), and protected characteristics (e.g. sexuality), and actions or objectives were specified to achieve outcomes for these groups. Ireland highlighted the need to consider a person's circumstances and background in their healthcare; the PSP in the Netherlands did not provide guidance for different patient groups beyond the need to pay special attention to older people (e.g. within the ten patient safety themes).

It is difficult to directly attribute changes in patient outcomes to specific policy and management changes, especially when different components are introduced incrementally, and often over many years; only three countries have conducted national evaluations of their approach. In the Netherlands, a longitudinal study comparing data from 2008[41] and 2011/12[42] reported a 45% decrease in potentially preventable adverse events over the course of the national safety programme. However, a more moderate 30% decrease in potentially preventable adverse events, which was not statistically significant, was found after adjusting for data clustering and changes in the patient mix between measurements.[43] There was also variation in the successful implementation of the ten themes between hospitals and departments, with a number of staff-related, organisational and topic-specific factors identified as potential modifiers.[15] Australia and Ireland both found improvements for certain aspects of patient safety,[20, 44, 45] with some of these in areas in which the Netherlands also saw improvement (e.g. medication safety).[16] Similarly, there were factors which all of these countries identified as facilitating better patient safety such as strong governance or leadership.[16, 20, 44, 45]

4. Discussion

Of the five included countries, only the Netherlands has formally adopted an SMS approach.[16] All SMS components were found in the policies of the other four countries, but not in the form of a single unified system. Across all five countries, there was a change in the patient safety discourse away from a narrow focus on reporting and learning from incidents. Whilst reporting was still considered important, the new approaches to patient safety adopted broader definitions of safety (e.g. including psychological and cultural safety) and harm (e.g. including harm resulting from social inequalities and structural oppression), and emphasised taking a systems perspective to safety, learning from successful practice as well as failures. They highlighted the need for wider involvement in the processes of assessing and creating safety, including patients and families as well as staff.

Healthcare is often thought to have more complex and unpredictable demands compared to other industries which have adopted an SMS approach, leading to debate over the suitability of SMSs in contexts and organisations in which service delivery is not stable or predictable.[4]

For peer review only - http://bmjopen.bmj.com/site/about/guidelines.xhtml

The Dutch patient safety programme had some positive impact on patient safety, suggesting an SMS approach is suitable for use in healthcare,[16] but this does not mean it is the only way to improve patient safety.[4] Whilst the elements of an SMS were present, to some extent, in the healthcare systems of all included countries, concepts from high-risk industries, and safety science more widely, had influenced the patient safety approach in all countries, with evidence of effectiveness in some cases (e.g. Ireland[44]).

The use of these concepts potentially reflects a new, emerging perspective in which healthcare and high-risk industries are seen as societal and corporate domains that share the same theoretical field – safety science – and can learn from each other. This is very different from the idea that, in terms of safety, healthcare is lagging behind and should learn and even copy the high-risk industries' approach to process safety, which has dominated the patient safety discourse.[4, 46] There were considerable differences in the implementation of SMS elements and safety concepts between countries, with patient safety systems reflecting different cultural and organisational realities and local preferences. What works, or makes sense, in one context may not work, or make sense, in another. Comparing and understanding such differences could advance the field of safety science as applied to healthcare and help countries review and improve their own policies and practice. For example, a national review of patient safety in England in 2024 found that, whilst individual healthcare organisations are prioritising common patient safety problems, there is a need for a more focused and coherent approach at the national level.[1] The experiences of a smaller country, such as the Netherlands, which was able to implement a single, highly structured patient safety programme, may be more applicable to the UK context than those of larger, federal countries such as Australia and Canada.

Within this general shift in thinking, different countries had preference for specific concepts and theories from safety science, the most prominent of which were the Measurement and Monitoring of Safety Framework (MMSF) and resilience engineering theory (see Glossary). Regardless of the theoretical underpinnings of a particular patient safety approach, it still needs to be both operationalised and adapted to a particular context. Whilst we focused in this review on how a national organisation can influence or coordinate the implementation of an SMS or safety approach, some policies and most interventions are implemented at the level of service commissioning and/or within health service delivery organisations. For example, the MMSF is a framework and a set of "guiding principles for safety measurement and monitoring"(p.70).[4] It does not provide details on implementation, which remains a challenge, as can be seen in the experience of Canadian healthcare providers of applying the MMSF in their healthcare system.[25] In terms of adaptation, current patient safety discourse recognises that both 'Safety I' (learning from incidents) and 'Safety II' (learning from success) are important and should be part of a modern patient safety approach. Safety II has important implications for the organisation of patient safety, particularly the use of a broader range of safety metrics including outcome measures and resilience indicators as well as adverse events.[47]

However, chosen measures and indicators need to be suitable for the context in which they are being used. As monitoring and evaluation are integral to a systems approach to safety, this is a key area for further research. Realist research, to understand the causal mechanisms through which patient safety initiatives work, and contextual factors that influence their successful implementation, could aid the choice of suitable methods of evaluation. Understanding the mechanisms behind different components of patient safety initiatives would also support their adaptation to different contexts. For example, the dominant focus of patient safety approaches in all countries was on hospital care (although all countries apart from the Netherlands included all healthcare settings in their patient safety

For peer review only - http://bmjopen.bmj.com/site/about/guidelines.xhtml

approaches), but there may be different challenges in other settings such as primary and community care, and for specific patient groups (e.g. patients with learning disabilities), requiring the tailoring of standards.

4.1 Limitations

Although we followed best practice for conducting a systematic review, some limitations should be acknowledged:

- The review is focused on patient safety in selected high-income, English-speaking countries with healthcare systems broadly comparable to that in the UK.
- Our interpretation is based on analysis of non-evaluative and administrative documents, many of which express aspirations regarding patient safety, rather than being empirically grounded evidence detailing outcomes. We tried to mitigate this limitation by talking to experts from each country and sharing our draft descriptions with them for comment.

5. Conclusions

Only the Dutch patient safety programme was explicitly based on an SMS approach and showed some longitudinal evidence of improving patient safety. Process safety as conceptualised in high-risk industries had some influence on approaches in the other countries, but this was less systematic and explicit. Approaches to patient safety in all countries also draw on a range of concepts from broader safety science. This reflects a shift from the view that healthcare needs to adopt high-risk industries' approach to safety, and increasing awareness that for initiatives to be successful, they need to be adapted to their context, especially through more collaborative learning between industries and sectors.

Glossary

Human factors is the "*study of the interrelationships between humans, the tools they use, and the environment in which they live and work*"(p.210).[46]

Learning review is a specific approach for investigating incidents where the focus is on collaboration and learning, and the system rather than on the individuals involved.[48]

Patient mix-up Errors regarding a patient's identity or their procedure (e.g. surgery involving the wrong side, wrong site, wrong procedure, or wrong patient).[16]

Resilience engineering theory "*looks for ways to enhance the ability of systems to succeed under varying conditions*" i.e. proactively building resilient systems.[4, 49]

Resilient systems/organisations can adjust their functioning before, during, or after events, sustaining required operations under both expected and unexpected conditions.[49]

Restorative practice in the context of the New Zealand patient safety policy *"…is a voluntary, relational process where skilled facilitators support all those affected by an adverse event in a safe and supportive environment. Participants can speak openly about what happened, to understand the human impacts and to clarify responsibility for the actions required for healing and learning.*"[50]

For peer review only - http://bmjopen.bmj.com/site/about/guidelines.xhtml

Root cause analysis is a process used to identify the root – or most fundamental -cause of a problem or incident (rather than the first viable cause).[5, 6]

Safety I is an approach to safety in which the focus is on preventing errors and incidents occurring by identifying the causes and responding either when an event occurs or when a risk is deemed unacceptable.[47]

Safety-II is an approach to safety which shifts the focus from what goes wrong to ensuring that things go right (learning from success).[4, 47]

Safety science is considered to be any research, education etc. concerning the science and technology of human safety.[51]

SMART stands for Specific, Measurable, Acceptable, Realistic and Time-bound, in relation to setting objectives.

System safety acknowledges that multiple factors, and the interactions between them, lead to safety, and that the system can be modified to improve safety and resilience.[4]

Wrong site surgery A surgical procedure carried out on the wrong side of the body, or on the wrong site on the body.[16]

Acknowledgements

The Exeter HSDR Evidence Synthesis Centre team would like to acknowledge and thank Tracey Herlihey, Matt Fogarty and Jason Cox for their support in developing this review, the experts who took the time to provide us with insight on patient safety in their countries, and our public collaborators for their valuable contributions to our understanding of the topic. We would also like to thank Sue Whiffin and Jenny Lowe for administrative support throughout this review.

Funding

This report presents independent research funded by the National Institute for Health Research (NIHR). This review (award NIHR136105) was commissioned by the Health Services and Delivery Research (HS&DR) programmes as part of a series of evidence syntheses under award NIHR130538. For more information, visit https://fundingawards.nihr.ac.uk/award/NIHR135660 and https://fundingawards.nihr.ac.uk/award/NIHR130538.

JTC and AB are also supported by the National Institute for Health Applied Research Collaboration South West Peninsula.

Author contributions

Role	Name
Conceptualisation	Sian de Bell (Equal), Zhivko Zhelev (Equal), Alison Bethel (Equal), Jo Thompson Coon (Equal), Rob Anderson (Equal)
Data Curation	Not applicable
Formal Analysis	Sian de Bell (Equal), Zhivko Zhelev (Equal)

For peer review only - http://bmjopen.bmj.com/site/about/guidelines.xhtml

Funding Acquisition	Jo Thompson Coon (Equal), Rob Anderson (Equal)
Investigation	Sian de Bell (Equal), Zhivko Zhelev (Equal), Alison Bethel (Supporting), Maria Clarke (Supporting)
Methodology	Not applicable
Project Administration	Jo Thompson Coon (Equal), Rob Anderson (Equal)
Resources	Not applicable
Software	Not applicable
Supervision	Not applicable
Validation	Not applicable
Visualisation	Zhivko Zhelev (Lead)
Writing – Original Draft	Sian de Bell (lead), Zhivko Zhelev (Supporting)
Writing – Editing and Reviewing	Sian de Bell (Equal), Zhivko Zhelev (Equal), Alison Bethel (Equal), Maria Clarke (Equal), Jo Thompson Coon (Equal), Rob Anderson (Equal)

Competing interests

Siân de Bell - Disclosure of interest: none declared

Zhivko Zhelev - Disclosure of interest: none declared

Alison Bethel - Disclosure of interest: none declared

Jo Thompson Coon

- Member of the NIHR HTA General Board (2019-2024)
- Member of NIHR PRU Commissioning Panel 2022
- Member of the NIHR RPSC Committee (2024 -)

Rob Anderson - Disclosure of interest: none declared

Data-sharing statement

This is an evidence synthesis study based on published primary research, it did not generate new data. All data extracted from the included publications, along with links to each publication, can be found in the report or Appendices. Further information can be obtained from the corresponding author.

Ethics statement

This was an evidence review, based on published primary studies, so did not require ethical approval.

Information governance statement

All data used in this paper is taken from published sources: no personal data was included.

References

1. Illingworth J, Fernandez Crespo R, Hasegawa K, Leis M, Howitt P, Darzi A. *The National State of Patient Safety 2024: Prioritising improvement efforts in a system under stress*. London: Imperial College London; 2024.

2. Health Services Safety Investigations Body. *Safety management systems - an introduction for healthcare*. 2023. URL: https://www.hssib.org.uk/patient-safety-investigations/safety-management-systems/investigation-report/ (accessed 24 April 2024).

For peer review only - http://bmjopen.bmj.com/site/about/guidelines.xhtml

3. Institute for Global Health Innovation. *National state of patient safety 2022: What we know about avoidable harm in England*: Imperial College London; 2022.
4. Vincent C, Burnett S, Carthey J. *The measurement and monitoring of safety*. London: The Health Foundation; 2013.
5. International Nuclear Safety Advisory Group. *Management of operational safety in nuclear power plants (INSAG-13)*. Vienna, Austria: International Nuclear Safety Advisory Group; 1999.
6. International Civil Aviation Organisation (ICAOSMM). *Safety management manual (Doc 9859, 4th Edition)*. Montreal, Canada: International Civil Aviation Organisation; 2018.
7. Thomas M. *A systematic review of the effectiveness of safety management systems. ATSB Transport Safety Report, Cross-modal Research Investigation, XR-2011-0*. Canberra: Australian Transport Safety Bureau; 2012.
8. Li Y, Guldenmund FW. Safety management systems: A broad overview of the literature. *Safety Science* 2018;**103**:94-123. https://doi.org/10.1016/j.ssci.2017.11.016
9. Kapur N, Parand A, Soukup T, Reader T, Sevdalis N. Aviation and healthcare: a comparative review with implications for patient safety. *JRSM Open* 2016;**7**. https://doi.org/10.1177/2054270415616548
10. OGP, IPIECA. *Operating Management System Framework for controlling risk and delivering high performance in the oil and gas industry, OGP Report No 510*. London, UK: OGP & IPIEC; 2014.
11. Zhelev Z, de Bell S, Bethel A, Clarke M, Anderson R, Thompson Coon J. The implementation of Safety Management Systems (SMS) in healthcare: a systematic review and international comparison. *Health and Social Care Delivery Research* 2025;**13**. https://doi.org/10.3310/QPLF8546
12. World Bank. *World Bank country and lending groups*. 2023. URL: https://datahelpdesk.worldbank.org/knowledgebase/articles/906519-world-bank-country-and-lending-groups (accessed 31 January 2024).
13. Braithwaite J, Zurynski Y, Ludlow K, Holt J, Augustsson H, Campbell M. Towards sustainable healthcare system performance in the 21st century in high-income countries: A protocol for a systematic review of the grey literature. *BMJ Open* 2019;**9**:e025892. https://doi.org/10.1136/bmjopen-2018-025892
14. Bal R, Wagner C. Chapter 27: Patient safety in Dutch hospitals: How can we explain success? In: Braithwaite J, Mannion R, Matsuyama Y, Shekelle P, Whittaker S, Al-Adawi S, editors. *Health systems improvement across the globe: Success stories from 60 countries.*Boca Raton, FL: CRC Press; 2018:205-12.
15. de Blok C, Koster E, Schilp J, Wagner C. *Implementation of the Dutch national Patient Safety Programme (VMS Veiligheidsprogrammesma)*. Utrecht/Amsterdam, Netherlands: NIVEL & EMGO+; 2013.
16. de Blok C, Koster E, Schilp J, Wagner C. *Implementatie VMS Veiligheidsprogramma. Evaluatieonderzoek in Nederlandse ziekenhuizen*. Utrecht /Amsterdam, Netherlands: NIVEL & EMGO+; 2013.
17. *Tijd voor Verbinding*. no date. URL: https://www.programmatvv.nl/ (accessed 25 April 2024).
18. Royal Netherlands Standards Institute. *Dutch Technical Agreement (NTA) 8009 'Safety management system for hospitals and institutions that provide hospital care'*. Delft, Netherlands: Royal Netherlands Standards Institute; 2007.
19. Royal Netherlands Standards Institute. *Dutch Technical Agreement (NEN) 8009 'Safety management system for hospitals and institutions that provide hospital care'*. Delft, Netherlands: Royal Netherlands Standards Institute; 2018.
20. Australian Commission on Safety and Quality in Health Care. *The state of patient safety and quality in Australian hospitals 2019*. Sydney, Australia: Australian Commission on Safety and Quality in Health Care; 2019.
21. Australian Commission on Safety and Quality in Health Care. *National safety and quality health service standards. 2nd edition*. Sydney, Australia: Australian Commission on Safety and Quality in Health Care; 2021.

For peer review only - http://bmjopen.bmj.com/site/about/guidelines.xhtml

22. Health Standards Organisation, Canadian Patient Safety Institute. *The Candanian quality and patient safety framework for health services*. 2020. URL: https://www.healthcareexcellence.ca/en/resources/canadian-quality-and-patient-safety-framework-for-health-services/ (accessed 24 April 2024).
23. Canadian Patient Safety Institute. *Strengthening commitment for improvement together. A policy framework for patient safety*. Edmonton, AB, Canada: Canadian Patient Safety Institute; 2019.
24. Healthcare Excellence Canada, Patients for Patient Safety Canada. *Rethinking patient safety. A discussion guide for patients, healthcare providers and leaders*. 2023. URL: https://www.healthcareexcellence.ca/media/gx4l3idd/rethinking-patient-safety.pdf (accessed 24 April 2024).
25. Goldman J, Rotteau L, Flintoft V, Jeffs L, Baker G. Measurement and monitoring of safety framework: a qualitative study of implementation through a Canadian learning collaborative. *BMJ Qual Saf* 2023;**32**:470. https://doi.org/10.1136/bmjqs-2022-015017
26. Health Information and Quality Authority. *National standards for safer better healthcare*. Dublin, Ireland: Health Information and Quality Authority; 2012.
27. Health Information and Quality Authority. *Standards development framework: a principles-based approach*. Dublin, Ireland: Health Information and Quality Authority; 2021.
28. Health Service Executive. *Patient safety strategy 2019-2024*. Dublin, Ireland: Health Service Executive; 2019.
29. Health Service Executive. *Incident management framework*. Dublin, Ireland: Health Service Executive; 2020.
30. Health Information and Quality Authority. *National standards for the conduct of reviews of patient safety incidents*. Dublin, Ireland: Health Information and Quality Authority; 2017.
31. Health Service Executive. *Healthcare audit – quality assurance and verification. Standards.* Dublin, Ireland: Health Service Executive; 2019.
32. Health Service Executive. *HSE Enterprise risk management policy and procedures 2023*. Dublin, Ireland: Health Service Executive; 2023.
33. Byrne D, O'Dowd E, Lydon S, McDermott BR, O'Connor P. *The national simulation strategic guide for the implementation of simulation on clinical sites*. Galway, Ireland: National University of Ireland Galway; 2021. https://doi.org/10.13025/cn0z-bp50
34. Irish Assoication for Simulation. *Irish Association for Simulation*. 2024. URL: https://www.iassim.com/about-us (accessed 08 April 2024).
35. Ministry of Health. *Pae Ora (Healthy Futures) Act 2022*. Wellington, New Zealand: New Zealand Government; 2022.
36. Standards New Zealand. *NZS 8134:2021 Ngā Paerewa Health and disability services standard*. Wellington: Standards New Zealand; 2021.
37. Te Tāhū Hauora Health Quality & Safety Commission. *Te Kaupapa Here hei tuku pūrongo mō ngā 'Aituā ā-Motu': National adverse events reporting policy*. Wellington, Aotearoa New Zealand: Te Tāhū Hauora Health Quality & Safety Commission; 2023.
38. Te Tāhū Hauora Health Quality & Safety Commission. *Clinical governance framework: collaborating for quality (draft, November 2023)*. Wellington, Aotearoa New Zealand: Te Tāhū Hauora Health Quality & Safety Commission; 2023.
39. Te Tāhū Hauora Health Quality & Safety Commission. *National adverse events reporting policy*. 2023).
40. Te Tāhū Hauora Health Quality & Safety Commission. *Clinical governance: guidance for health and disability providers*. Wellington, Aotearoa New Zealand: Te Tāhū Hauora Health Quality & Safety Commission; 2017.
41. Langelaan M, Baines RJ, Broekens MA, Siemerink KM, van de Steeg L, Asscheman H*, et al. Monitor zorggerelateerde schade 2008: Dossieronderzoek in Nederlandse ziekenhuizen*. Utrecht, Amsterdam: NIVEL en EMGO+ Instituut; 2010.
42. Langelaan M, de Bruijne MC, Baines RJ, Broekens MA, Hammink K, Schilp J*, et al. Monitor Zorggerelateerde Schade 2011/2012: Dossieronderzoek in Nederlandse ziekenhuizen* Utrecht, Amsterdam: NIVEL & EMGO+ Instituut; 2013.

For peer review only - http://bmjopen.bmj.com/site/about/guidelines.xhtml

43. Baines R, Langelaan M, de Bruijne M, Spreeuwenberg P, Wagner C. How effective are patient safety initiatives? A retrospective patient record review study of changes to patient safety over time. *BMJ Qual Saf* 2015;**24**:561-71. https://doi.org/10.1136/bmjqs-2014-003702
44. Health Information and Quality Authority. *Overview report of five years of HIQA monitoring in Irish public acute hospitals against national standards: 2015–2019*. Dublin, Ireland: Health Information and Quality Authority; 2020.
45. Australian Commission on Safety and Quality in Health Care. *Creating safer, better health care - the impact of the national safety and quality health service standards*. Sydney, Australia: Australian Commission on Safety and Quality in Health Care; 2018.
46. Institute of Medicine (US) Committee on Quality of Health Care in America. *To err is human: building a safer health system*. Washington, DC: National Academies Press (US); 2000.
47. Hollnagel E, Wears RL, Braithwaite J. *From safety-I to safety-II: A White Paper*: The Resilient Health Care Net: Published simultaneously by the University of Southern Denmark, Denmark, University of Florida, USA, and Macquarie University, Australia; 2015.
48. Te Tāhū Hauora Health Quality & Safety Commission. *Guide to doing a learning review*. Wellington, Aotearoa New Zealand: Te Tāhū Hauora Health Quality & Safety Commission; 2023.
49. Hollnagel E. *Resilience Assessment Grid (RAG)*. 2024. URL: https://erikhollnagel.com/ideas/resilience%20assessment%20grid.html (accessed 15/04/2024).
50. Te Tāhū Hauora Health Quality & Safety Commission. *Mahi haumanu, hohou te rongo. Restorative practice and hohou te rongo*. 2023. URL: https://www.hqsc.govt.nz/our-work/system-safety/healing-learning-and-improving-from-harm-policy/restorative-practice-and-hohou-te-rongo/ (accessed 22 April 2024).
51. Aven T. What is safety science? *Saf Sci* 2014;**67**:15-20. https://doi.org/doi.org/10.1016/j.ssci.2013.07.026

For peer review only - http://bmjopen.bmj.com/site/about/guidelines.xhtml

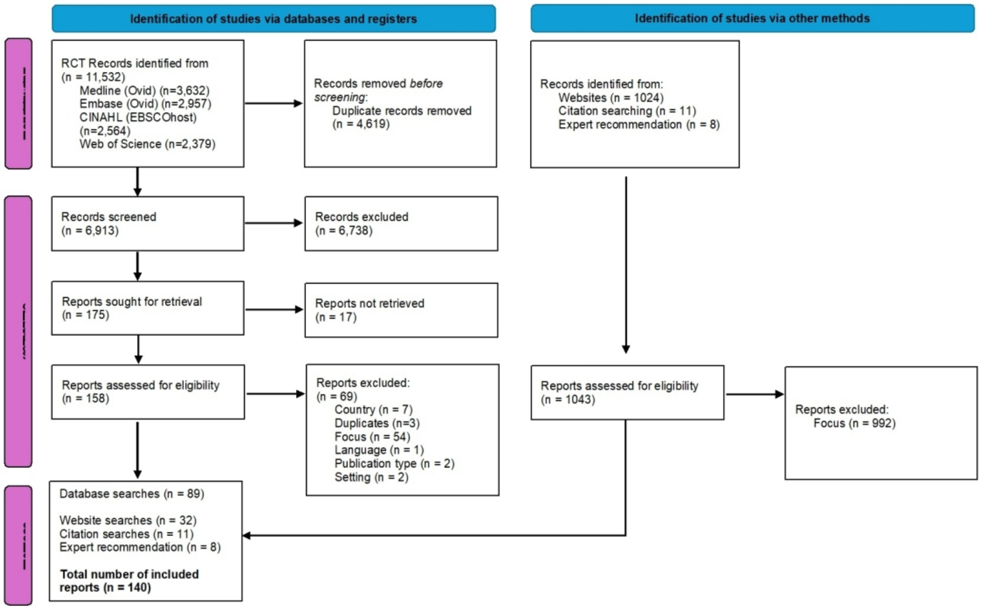

Figure 1 PRISMA diagram of the screening process

298x180mm (144 x 144 DPI)

Supplementary material 1: Protocol

University of Exeter

ESMI Evidence Synthesis & Modelling for Health Improvement

The implementation of Safety Management Systems (SMS) in healthcare

Systematic review protocol

Version 5

24 November 2023

For peer review only

For peer review only - http://bmjopen.bmj.com/site/about/guidelines.xhtml

**Produced by**	Exeter HS&DR Evidence Synthesis Centre<br>Department of Health & Community Sciences<br>South Cloisters<br>St Luke's Campus<br>Heavitree Road<br>Exeter<br>EX1 2LU<br>UK
**Authors**	**Zhivko Zhelev** Systematic Reviewer [1]<br>z.zhelev@exeter.ac.uk<br>**Sian de Bell** Systematic Reviewer [1]<br>s.c.de-bell@exeter.ac.uk<br>**Alison Bethel** Information Specialist[1]<br>a.bethel@exeter.ac.uk<br>**Maria Clarke** Information Specialist[1]<br>m.e.clarke2@exeter.ac.uk<br>**Rob Anderson** Senior/Academic Lead[1]<br>r.anderson@exeter.ac.uk<br>**Jo Thompson Coon** Senior/Academic Lead[1]<br>j.thompson-coon@exeter.ac.uk<br><br>[1] University of Exeter
**Correspondence to**	z.zhelev@exeter.ac.uk
**Date completed**	24 November 2023
**Source of funding**	This work was commissioned by the NIHR HSDR programme as a project (project code NIHR136105) within grant number NIHR130538.
**Role of funder/institution in protocol creation**	The aims and scope of this protocol and the planned work were informed by information provided by and discussions with staff in the NHS Patient Safety Team and Department of Health & Social Care

For peer review only - http://bmjopen.bmj.com/site/about/guidelines.xhtml

**Declared competing interests of the authors**	None
**Rider on responsibility for document**	The views and opinions expressed by authors in this publication are those of the authors and do not necessarily reflect those of the NHS, the NIHR, NETSCC, the HSDR programme or the Department of Health & Social Care. Any errors are the responsibility of the authors.
**PROSPERO registration number**	CRD42023487512

For peer review only

For peer review only - http://bmjopen.bmj.com/site/about/guidelines.xhtml

Table of Contents

1 Background ... 5
1.1 Development of the review ... 6
1.2 Research questions ... 7
2 Methods ... 8
2.1 Approach to evidence synthesis ... 8
2.2 Identification of studies ... 9
2.2.1 Searches ... 9
2.2.2 Inclusion and exclusion criteria ... 9
2.2.3 Process for applying inclusion criteria ... 11
2.3 Data extraction ... 11
2.4 Study quality assessment strategy ... 12
2.5 Data analysis and presentation ... 12
3 Stakeholder and patient/public involvement ... 14
4 Dissemination plans ... 15
5 References ... 16

For peer review only

For peer review only - http://bmjopen.bmj.com/site/about/guidelines.xhtml

1 Background

There are a number of industries, from aviation to nuclear power, in which safety is a priority. Within these industries, failures or errors could have serious consequences for people, causing loss of life or injury, and for the environment, in terms of environmental damage or harm to equipment or property.(1) Healthcare is now considered to be one of these 'safety-critical industries', with safety in this setting defined as:

> "The avoidance, prevention and amelioration of adverse outcomes or injuries stemming from the process of healthcare."(2)

Examples of safety incidents in healthcare include medication errors, wrong site surgery, and lack of a timely response to deterioration in a patient's condition.

High-risk industries have improved safety by moving from measuring and responding to specific incidents, to assessing the presence of, and managing, the conditions that lead to hazards.(3) This requires a systems thinking approach, considering interactions and intersections between social and technological elements, which are where safety events tend to occur.(3, 4) Change in some areas may be slow, such as to structural factors e.g. physical infrastructure or the organisation of departments, whereas in other areas of the system, particularly those dependent on more transient 'mediating' factors such as morale, teamwork, and individual performance, change may be affected more rapidly.(3)

Within many safety-critical industries, a systems approach to safety has been operationalised in the form of safety management systems (SMS): "an organised approach to managing safety, including the necessary organisational structures, accountabilities, policies and procedures".(5) These are generally considered to have four key components:

- Policy and objectives,
- Risk management,
- Safety assurance,
- Safety promotion (safety culture).(5)

These are sometimes expanded into six areas, offering further detail on considerations and requirements within the four key components identified above: (i) a safety policy; (ii) organisational arrangements to support safety e.g. supervision and training of staff in implementing the safety policy and processes; (iii) a safety plan, detailing standards and processes for safety, including risk assessment; (iv) measurement of safety performance; (v)

For peer review only - http://bmjopen.bmj.com/site/about/guidelines.xhtml

reviews of safety performance e.g. incident investigation, safety audits; and (vi) feedback loops to improve safety performance.(3)

A systematic review of SMS in three safety-critical industries - aviation, marine and rail – published in 2012 indicated that they led to improvements in safety e.g. reduced accident rates.(6) There is some debate over whether indicators such as accident rates are an appropriate way of measuring the effectiveness of SMS,(7) but this does suggest that they have the potential to improve patient safety in organisations providing healthcare. However, whilst the basic principles of SMS are widely accepted as transferrable,(4, 7) evidence from other industries suggests that the details (e.g. of accountability frameworks) need to be context-specific to be effective.(8) In healthcare, this might involve identifying and improving the management of known patient safety risks.(9) Components of SMS, such as national strategies for improving patient safety,(4) exist in most healthcare systems, and some countries, such as Australia (10) and the Netherlands,(11) are explicitly developing and promoting safety management systems. Their experiences offer opportunities for learning that could support the implementation of the principles of SMS in other healthcare systems, which will have different starting points in terms of the organisation and management of healthcare, and different pre-existing system components.

1.1 Development of the review

This review of research and other evidence has been commissioned by the NIHR HSDR programme to provide evidence and information to NHS England and the NHS Patient Safety Team. It is primarily intended to inform the further development of the NHS England's patient safety policy and practice as laid out in the NHS Patient Safety Strategy 2019(12) and related documents.

The policy questions of interest detailed in the project brief were:

Q1: What are the key attributes of a successful SMS for the NHS in England?

Q2: What are the links between a SMS and a Quality Management System?

Q3: What are the next steps for safety management in the NHS in England?

Evidence is emerging that could begin to answer these questions following the implementation of NHS Patient Safety Strategy 2019.(12) For example, the work of the National Patient Safety team is saving an estimated 160 lives per year (13), and evaluation of the Patient Safety Incident Response Framework (PSIRF) early adopter programme indicates it has led to improved safety cultures and identification of more effective risk reduction strategies in participating organisations.(13) A recent report published by the

For peer review only - http://bmjopen.bmj.com/site/about/guidelines.xhtml

Healthcare Safety Investigation Branch (HSIB)(14) has used evidence on the implementation of SMS in a range of high-risk industries, from aviation to the oil and gas industry, to describe key attributes of SMS at a broad level and make recommendations for the further development of a SMS in the NHS. However, more detailed evidence is needed to assess how a SMS approach might be applicable to the NHS in England.

The review described in this protocol aims to provide information on SMS in healthcare specifically, by investigating how they have been implemented in other countries. This will offer context to inform primary research into the questions raised above, and consideration of how NHS England's current patient safety policy, processes and practice could be extended into a full SMS.

1.2 Research questions

In high-income countries applying the SMS approach (as specified below), what principles have been used to inform healthcare policy, management and practice related to patient and staff safety and what is the evidence supporting their adoption and implementation?

Within this broader question, our specific questions are:

How are the components of SMS reflected in the healthcare policy documents of the above countries, or their regional healthcare systems?

What research (e.g. formal evaluations, or based on routine data) or other relevant evidence (e.g. staff and patient narratives) is available regarding the effectiveness, implementation or experience of SMS within healthcare?

What does existing research and other relevant evidence from beyond the UK tell us about the effectiveness, implementation or experience of SMS within healthcare?

For peer review only - http://bmjopen.bmj.com/site/about/guidelines.xhtml

2 Methods

2.1 Approach to evidence synthesis

As noted above, there is a paucity of empirical evidence on the use of SMS in healthcare. Also, the specific application of such systems in healthcare is likely to depend on the local context, reflecting pre-existing arrangements as well as current priorities and initiatives. To better understand the context of each application and given the expectation of limited empirical evidence, we will conduct a systematic review with a broader scope, including, in addition to research evidence, an analysis of policy documents, training materials and other relevant information (e.g. patients and healthcare professionals' narratives). This will allow better understanding of the logic and process of each implementation; more adequate interpretation of the available empirical evidence; better understanding of the similarities and differences across systems; and the identification of specific barriers, facilitators and challenges that need to be addressed in future research. The review will follow the process detailed below.

Firstly, we will take the four pillars of a SMS as defined by HSIB (14), and compare them to other key SMS literature e.g. documents from safety-critical industries such as aviation (5). In consultation with stakeholders, we will produce an expanded and more detailed framework of the components of a SMS. This will be used to inform data extraction and analysis, to allow us to identify the aspects of SMS that have and have not been adopted by different healthcare systems.

Secondly, we will identify national or regional healthcare systems in which SMS principles have been used to inform policy and practice related to patient and staff safety. For pragmatic reasons, we will focus first on high-income English-speaking countries and, if necessary and feasible, include other countries after discussion with stakeholders. If key policy documents do not state explicitly that a SMS approach was used, then we will contact relevant organisations to enquire about this. Only healthcare systems where such a link and commitment are explicitly stated will be included in the review.

Thirdly, we will identify relevant policy documents, research and other relevant information (e.g. patient stories) from the organisations' websites and by focused searches of electronic databases and other sources. Each organisation will be contacted to ascertain that all relevant information has been captured and request additional data that may have been missed or is not available online.

Fourthly, we will develop a data extraction form organised around the core principles of SMS (as defined in the background) and the different types of evidence we are including: policy

For peer review only - http://bmjopen.bmj.com/site/about/guidelines.xhtml

documents, research and other evidence. If possible, data will be extracted verbatim or, if too long, summarised with reference to its location in the source document. Rather than just depicting a snapshot of the *status quo*, as much as possible, the information gathered will attempt to distinguish the sequence and timings of the adoption and application of different core principles of SMS, or of the main strategies and processes to implement and embed them in a particular country or region/province. This therefore aims to shed light on which principles and systems/processes may have pre-existed the formal policy commitment to adopting a SMS approach, which were seen as most critical for expanding the scope and depth of the SMS, and interactions between the different components of SMS.

Finally, information on the implementation of SMS in the eligible healthcare systems will be synthesised narratively and summarised in tables and graphs. Common themes across different healthcare systems will be identified, evidence gaps will be highlighted, and recommendations on how to address those will be made.

2.2 Identification of studies

2.2.1 Searches

The main method for identifying relevant policy documents, research and other evidence will be through searching the websites of, and direct contact with, relevant organisations (e.g. Australian Commission on Safety and Quality in Healthcare). Website searching will be carried out either by browsing publication lists or using site search functionality. Search parameters (e.g. keywords, publication date) will be pre-specified and the search process will be documented (e.g. number of hits, number of potentially relevant titles) to allow transparency and reproducibility.

The above will be complemented by focused database searches using search strategies developed by an information specialist (AB) in consultation with the review team and the commissioning group. The database search strategies will use both controlled vocabulary when available and relevant (e.g. MeSH in MEDLINE) and free-text searching. Search terms will be partly derived from the literature and from stakeholders and experts' input. Searches will be limited to English-language texts published after a pre-specified date (to be agreed with stakeholders and experts). The reference lists of all relevant documents will be hand-searched for additional titles.

2.2.2 Inclusion and exclusion criteria

The following overarching inclusion and exclusion criteria will be applied:

For peer review only - http://bmjopen.bmj.com/site/about/guidelines.xhtml

- **Safety management system:** Safety management systems are defined by the following key elements:

    - policy and objectives
    - risk management
    - safety assurance
    - safety promotion (safety culture).

  Texts will be excluded if they refer to different concepts or models without direct link to the above definition of a safety management system.

- **Application of SMS** in the healthcare system of a high-income country (as defined by the World Bank). For pragmatic reasons, we will focus on English-speaking countries; if necessary and feasible, and after discussion with our policy costumer, we may extend the scope to include other, selected high-income countries.

- **Type of evidence:** we will include three distinct types of evidence:

    - Relevant policy documents, including documents related to the implementation of SMS, such as staff training and patient-facing materials

    - Published or unpublished research / evaluations (quantitative, qualitative or mixed methods),

    - Other evidence, e.g. patient and staff experience or opinions published on the organisations' websites.

- **Provenance of evidence:** As mentioned earlier, for pragmatic reasons we will focus first on high-income English-speaking countries as evidence from such healthcare systems is more likely to be relevant to the UK and all relevant policy documents and research will be available in English. Therefore, evidence related to the implementation of SMS in the healthcare of the following countries will be considered first: Ireland, Australia, New Zealand, Canada and the Netherlands. The list will be revised / amended once the initial searches have been completed and the volume, type and quality of evidence is assessed; or, it has been decided in discussion with experts and stakeholders that including a specific country or expanding the list is desirable and feasible.

For peer review only - http://bmjopen.bmj.com/site/about/guidelines.xhtml

- **Language:** we will include texts available in English; if the scope of the review is extended (see above) we may decide to include texts in other languages, if necessary and feasible.

2.2.3 Process for applying the inclusion criteria

A small sample (n ~ 10) of potentially relevant documents from the three types of evidence will be used to calibrate the selection process. Each document will be reviewed by multiple reviewers. Decisions will be compared and discussed in a group meeting to ensure consistent application of criteria. If necessary, definitions will be updated and explanatory notes added to aid the process.

The full text of all potentially relevant documents identified through the website searches and direct contact with the healthcare organisations will be retrieved and checked for relevance against the inclusion criteria independently by two reviewers.

The results from the database searches will be screened following the standard process. Two reviewers will independently apply the inclusion and exclusion criteria to the title and abstract of each identified citation. We will obtain the full text of papers where either reviewer judges it to be a potential inclusion. Two reviewers will assess the full text of each record independently for inclusion, with disagreements settled through discussion or by a third reviewer. The full texts of all potentially relevant publications identified from the website searches will be obtained and screened as per the above process.

The study selection process will be detailed using a PRISMA-style flowchart, with a reason reported for exclusion of each record assessed at full text.

2.3 Data extraction

A data extraction form will be developed and piloted independently by two reviewers on a small sample of included documents. Data will be extracted by one reviewer and checked by a second reviewer. Disagreements will be resolved through discussion.

The following data will be extracted:

- Country
- Healthcare organisation (purpose and remit, website)
- Type of document (policy, research, anecdotal evidence)
- Aims

For peer review only - http://bmjopen.bmj.com/site/about/guidelines.xhtml

- Relation to SMS (e.g. explicitly stated or implied, description)
- Information regarding specific SMS components:
    - Policy and objectives
    - Risk management
    - Safety assurance
    - Safety promotion (safety culture).
- Information regarding implementation and/or impact
- **Research:** research design, aim, methods, results, conclusions
- **Other evidence:** type of document, informant (patient, healthcare professional, policy maker), perspective (relationship to the programme), main points.

The NIHR-INCLUDE guidelines (15) were used to reflect on Equality, Diversity and Inclusion (EDI) whilst designing the protocol. EDI is important to consider in relation to the topic of safety management systems, as there may be differences in safety incidents between social and demographic groups and resulting inequities in health outcomes. However, it is likely this information will be difficult to ascertain from the types of evidence e.g. policy documents, we will be including in the review. We will consider PROGRESS-Plus characteristics during the process of data extraction and analysis (16, 17) and, where possible, use this information to inform the findings and recommendations of the review.

2.4 Study quality assessment strategy

The methodological quality of all research studies will be assessed using validated quality assessment tools according to the specific research design. Quality will be assessed by two reviewers independently and disagreements resolved through discussion or arbitration. No quality appraisal will be carried out on policy documents or non-research evidence.

2.5 Data analysis and presentation

The type of information identified for each healthcare system will be summarised and the following information will be reported:

- The way in which the SMS approach has informed patient and staff safety policy and practice.
- Information related to each of the SMS components (see above).

For peer review only - http://bmjopen.bmj.com/site/about/guidelines.xhtml

- Information related to the process and timing of implementing SMS (e.g. steps, processes etc.).
- Evidence related to the effectiveness / impact and implementation of SMS.
- Stakeholders' perceptions and experience.

Comparisons across healthcare systems will be carried out to identify similarities and differences, including barriers, facilitators and challenges; the volume, type and quality of the available evidence and any evidence gaps that need to be addressed in future research. The results will be summarised in tables and diagrams and presented narratively, considering their relevance to the application of SMS in the NHS of England.

For peer review only

For peer review only - http://bmjopen.bmj.com/site/about/guidelines.xhtml

3 Stakeholder and patient/public involvement and engagement

The following stakeholders will be involved:

- NHS England's Patient Safety Team and the Department of Health and Social Care (as commissioners of the work)
- Topic experts (including from other countries and other industries, if possible)
- Any other relevant Patient Safety organisations (NHS or external) e.g. local patient safety teams

Due to the focus of the review, we will consult Public and Patient Involvement and Engagement representatives who have some knowledge of the topic e.g. members of local patient safety teams, or PPIE representatives who have worked with the National Patient Safety Team.

We will consult stakeholders throughout the review process, to discuss topics such as the focus of the review, analysis, and findings. Meetings will be arranged to suit the project progress and stakeholder availability. We will also ask for their input on dissemination plans and materials as described below.

For peer review only

4 Dissemination plans

We will produce a report on the implementation of safety management systems in healthcare in high-income countries, available as an open access in the NIHR Journals Library.

Further materials for dissemination will be finalised after discussion with the project's advisory group. These are likely to include:

- an evidence briefing, giving a plain language summary of the report and its findings (primarily aimed at policy makers and health service managers);
- an article in an academic journal identified as being relevant to stakeholders for this review; and
- presentations at key national and regional meetings.

Outputs will be disseminated via the Exeter HSDR Evidence Synthesis Centre webpage and social media. Additional material may be produced to promote them, such as a blog post based on the evidence briefing and report.

For peer review only

For peer review only - http://bmjopen.bmj.com/site/about/guidelines.xhtml

5 References

1. Saunders F. Safety-critical industries: definitions, tensions and tradeoffs 2015 [Available from: http://fionasaunders.co.uk/safety-critical-industries-definitions-tensions-and-tradeoffs/#:~:text=Combining%20these%20two%20definitions%2C%20a%20safety-critical%20industry%20can,environmental%20damage%2C%20or%20harm%20to%20plant%20or%20property.

2. Vincent C. Patient safety. 2nd edition ed. Chichester: John Wiley and Sons; 2010.

3. Vincent C, Burnett S, Carthey J. The measurement and monitoring of safety. London: The Health Foundation; 2013.

4. Macchi L, Pietikäinen E, Reiman T, Heikkilä J, Ruuhilehto K. Patient safety management: Available models and systems. VTT; 2011.

5. International Civil Aviation Organisation Safety Management Manual (ICAOSMM). 2nd ed. Doc 9859, Safety Management Manual (SMM) 2009 [Available from: www2.icao.int/en/ism/Guidance%20Materials/DOC_9859_FULL_EN.pdf.

6. Thomas M. A systematic review of the effectiveness of safety management systems. ATSB Transport Safety Report, Cross-modal Research Investigation, XR-2011-0. Canberra: Australian Transport Safety Bureau; 2012.

7. Li Y, Guldenmund FW. Safety management systems: A broad overview of the literature. Safety Science. 2018;103:94-123.

8. Kapur N, Parand A, Soukup T, Reader T, Sevdalis N. Aviation and healthcare: a comparative review with implications for patient safety. JRSM Open. 2016;7.

9. HSIB. A thematic analysis of HSIB's first 22 national investigations. Healthcare Safety Investigation Branch I2020/016; 2021.

10. Australian Commission on Safety and Quality in Health Care. The state of patient safety and quality in Australian hospitals 2019. Sydney: Australian Commission on Safety and Quality in Health Care; 2019.

11. Bolk M. Final report: Embedding patient safety in education & training. VMS Safety Program; 2013.

12. NHS England. The NHS Patient Safety Strategy no date [Available from: https://www.england.nhs.uk/patient-safety/the-nhs-patient-safety-strategy/#key-docs.

13. NHS England. NHS patient safety strategy – progress so far no date [Available from: https://www.england.nhs.uk/patient-safety/the-nhs-patient-safety-strategy/nhs-patient-safety-strategy-progress-so-far/.

14. HSIB. Safety management systems - an introduction for healthcare. 2023.

15. National Institute for Health Research. Improving inclusion of under-served groups in clinical research: Guidance from the NIHR-INCLUDE project. UK: National Institute for Health Research; 2020.

For peer review only - http://bmjopen.bmj.com/site/about/guidelines.xhtml

16. O'Neill J, Tabish H, Welch V, Petticrew M, Pottie K, Clarke M, et al. Applying an equity lens to interventions: using PROGRESS ensures consideration of socially stratifying factors to illuminate inequities in health. J Clin Epidemiol. 2014;67:56-64.

17. Cochrane Methods Equity. PROGRESS-Plus no date [Available from: https://methods.cochrane.org/equity/projects/evidence-equity/progress-plus.

For peer review only

For peer review only - http://bmjopen.bmj.com/site/about/guidelines.xhtml

Supplementary material 2: Inclusion criteria

	Include	Exclude
**Population**	Populations with any health need.	-
**Initiative**	Any use of an SMS approach in a healthcare setting, with an SMS being defined by four key components:<br>• policy and objectives<br>• risk management<br>• safety assurance<br>• safety promotion (safety culture).<br><br>Where it is not clear whether a country is using an SMS approach, we will include studies if they relate to one or more components of an SMS.	Studies of safety where this is not linked to an SMS component.<br>Studies of occupational health and safety.
**Setting**	Any healthcare setting:<br>- Secondary care,<br>- Primary care.	Non-healthcare settings:<br>- Care homes,<br>- Home care.
**Country**	Australia, Canada, Ireland, the Netherlands, New Zealand.<br><br>Studies which included other countries were included if the results for these countries were reported separately.	Any other country.
**Language**	English or Dutch.	Any other language.

For peer review only

For peer review only - http://bmjopen.bmj.com/site/about/guidelines.xhtml

	Include	Exclude
**Type of document**	• Relevant policy documents, including documents related to the implementation of SMS, such as staff training and patient-facing materials<br>• Published or unpublished research / evaluations (quantitative, qualitative or mixed methods),<br>• Other evidence (e.g. patient and staff experience or opinions published on the organisations' websites).	Theoretical

For peer review only

For peer review only - http://bmjopen.bmj.com/site/about/guidelines.xhtml

Supplementary material 3: Excluded and thin studies

Excluded papers from database searching (n=66)

Excluded on Country (n=7)

Ma E, Wei W, Ho C. An environmental scan of patient safety reporting and learning systems in community healthcare for multi- disciplinary teams. *Can J Hosp Pharm*. 2022;**75**(2):135.

Coffey M, Marino M, Lyren A, Purcell D, Hoffman JM, Brilli R, et al. Association between hospital-acquired harm outcomes and membership in a national patient safety collaborative. *JAMA Pediatrics*. 2022;**176**(9):924-32.

Kristensen S, Hammer A, Bartels P, Sunol R, Groene O, Thompson CA, et al. Quality management and perceptions of teamwork and safety climate in European hospitals. *Int J Qual Health Care*. 2015;**27**(6):499-506.

Holmstrom AR, Laaksonen R, Airaksinen M. How to make medication error reporting systems work - Factors associated with their successful development and implementation. *Health Policy*. 2015;**119**(8):1046-54.

Windsor JA, Maxim S. Patient safety in medicine: Are surgeons ready for checklists? *ANZ Journal of Surgery*. 2010;**80**(1-2):3-5.

Sunol R, Vallejo P, Groene O, Escaramis G, Thompson A, Kutryba B, et al. Implementation of patient safety strategies in European hospitals. *Qual Saf Health Care. 2009*;**18**(Suppl 1):i57-61.

Shaw C, Kutryba B, Crisp H, Vallejo P, Sunol R. Do European hospitals have quality and safety governance systems and structures in place? *Qual Saf Health Care*. 2009;**18**(Suppl 1):i51-6.

Excluded on focus (n=54)

Ong N, Lucien A, Long JC, Weise J, Walton M, Burgess A. What do healthcare staff think about the quality and safety of care provided to children and young people with an intellectual disability? A qualitative study using the framework method of analysis. *BMJ Open*. 2023;**13**(7):e071494.

Kaud Y, McKeon D, Lydon S, O'Connor P. Measuring and monitoring patient safety in hospitals in the Republic of Ireland. *Irish J Med Sci*. 2023;1**92:**2581–259.

For peer review only - http://bmjopen.bmj.com/site/about/guidelines.xhtml

Cords CI, van Baar ME, Nieuwenhuis MK, Pijpe A, van der Vlies CH. Reliability and validity of a frailty assessment tool in specialized burn care, a retrospective multicentre cohort study. *Burns*. 2023;**49**(7):1621-31.

Terry D, Kim J-a, Gilbert J, Jang S, Nguyen H. "Thank You for Listening": An Exploratory Study Regarding the Lived Experience and Perception of Medical Errors Among Those Who Receive Care. *International Journal of Health Services*. 2022;**52**(2):292-302.

Arnold A, Ward I, Gandhidasan S. Incident review in radiation oncology. *J Med Imaging Radiat Oncol*. 2022;**66**(2):291-8.

Malik RF, Buljac-Samardzic M, Amajjar I, Hilders C, Scheele F. Open organisational culture: what does it entail? Healthcare stakeholders reaching consensus by means of a Delphi technique. *BMJ Open*. 2021;**11**(9):e045515.

Connell CJ, Cooper S, Endacott R. Measuring the safety climate in an Australian emergency department. *Int Emerg Nurs*. 2021;**58**:101048.

Adie K, Fois RA, McLachlan AJ, Walpola RL, Chen TF. The nature, severity and causes of medication incidents from an Australian community pharmacy incident reporting system: The QUMwatch study. *Br J Clin Pharmacol*. 2021;**87**(12):4809-22.

Wright KM, Bonser M. The Essential Steps of Medication Administration Practices Project medication administration improvement practices among acute inpatients in a tertiary hospital: a best practice implementation project. *International Journal of Evidence-Based Healthcare*. 2020;**18**(4):408-19.

Floyd E, Hodgkins R, Naylon L, Noon M, Sirotti L, Monaro S. The costs and benefits of pressure injury point prevalence auditing. *Wound Practice & Research*. 2020;**28**(2):66-74.

Emond Y, Wolff AP, Peters YAS, Bloo GJA, Westert GP, Damen J, et al. Reducing work pressure and IT problems and facilitating IT integration and audit & feedback help adherence to perioperative safety guidelines: a survey among 95 perioperative professionals. *Implement*. 2020;**1**:49.

Clay-Williams R, Taylor N, Winata T, Ting HP, Arnolda G, Braithwaite J. Organization quality systems and department-level strategies: refinement of the Deepening our Understanding in Quality in Australia (DUQuA) organization and department-level scales. *Int J Qual Health Care*. 2020;**32**(Supplement_1):22-34.

Braithwaite J, Taylor N, Clay-Williams R, Ting HP, Arnolda G. Conclusion: the road ahead: where should we go now to improve healthcare quality in acute settings? *Int J Qual Health Care*. 2020;**32**(Supplement_1):99-103.

Ward M, Ni She E, De Brun A, Korpos C, Hamza M, Burke E, et al. The co-design, implementation and evaluation of a serious board game 'PlayDecide patient safety' to educate junior doctors about patient safety and the importance of reporting safety concerns. *BMC Med Educ*. 2019;**19**(1):232.

For peer review only - http://bmjopen.bmj.com/site/about/guidelines.xhtml

McHugh S, Droog E, Foley C, Boyce M, Healy O, Browne JP. Understanding the impetus for major systems change: A multiple case study of decisions and non-decisions to reconfigure emergency and urgent care services. *Health Policy*. 2019;**123**(8):728-36.

Liao E, Eisenberg N, Kaushal A, Montbriand J, Tan KT, Roche-Nagle G. Utility of the Vascular Quality Initiative in improving quality of care in Canadian patients undergoing vascular surgery. *Can J Surg*. 2019;**62**(1):66-9.

Eljiz K, Greenfield D, Derrett A, Radmore S. Health system redesign: Changing thoughts, values, and behaviours for the co-production of a safety culture. *Int J Health Plann Manage*. 2019;**34**(4):1477-84.

Veitch D. One province, one healthcare system: A decade of healthcare transformation in Alberta. *Healthc Manage Forum*. 2018;**31**(5):167-71.

Sinclair JE, Austin MA, Bourque C, Kortko J, Maloney J, Dionne R, et al. Barriers to self-reporting patient safety incidents by paramedics: A mixed methods study. *Prehospital Emergency Care*. 2018;**22**(6):762-72.

Khalil H, Lee S. Medication safety challenges in primary care: Nurses' perspective. *Journal of Clinical Nursing*. 2018;**27**(9-10):2072-82.

Debono D, Taylor N, Lipworth W, Greenfield D, Travaglia J, Black D, et al. Applying the Theoretical Domains Framework to identify barriers and targeted interventions to enhance nurses' use of electronic medication management systems in two Australian hospitals. *Implement Sci*. 2017;**12**(1):42.

Davies JM, Delaney G. Can the Aviation Industry be Useful in Teaching Oncology about Safety? *Clin Oncol*. 2017;**29**(10):669-75.

Blijleven V, Koelemeijer K, Wetzels M, Jaspers M. Workarounds emerging from electronic health record system usage: Consequences for patient safety, effectiveness of care, and efficiency of care. *JMIR Hum Factors*. 2017;**4**(4):e27.

Wagner C, Merten H, Zwaan L, Lubberding S, Timmermans D, Smits M. Unit-based incident reporting and root cause analysis: variation at three hospital unit types. *BMJ Open*. 2016;**6**(6):e011277.

Ng J, Andrew P, Crawley M, Pevreal W, Peach J. Assessing a hospital medication system for patient safety: findings and lessons learnt from trialling an Australian modified tool at Waitemata District Health Board. *N Z Med J*. 2016;**129**(1430):63-77.

Gill FJ, Leslie GD, Marshall AP. Family initiated escalation of care for the deteriorating patient in hospital: Family centred care or just "box ticking". *Aust Crit Care*. 2016;**29**(4):195-200.

Yates M, Reddy M, Machumpurath B, Phelps G, Hampson SA. Modification of the National Inpatient Medication Chart improves venous thromboembolism prophylaxis rates in high-risk medical patients. *Intern Med J*. 2014;**44**(2):190-4.

For peer review only - http://bmjopen.bmj.com/site/about/guidelines.xhtml

Wong KS, Ryan DP, Liu BA. A system-wide analysis using a senior-friendly hospital framework identifies current practices and opportunities for improvement in the care of hospitalized older adults. *J Am Geriatr Soc*. 2014;**62**(11):2163-70.

Teare GF. Measurement of quality and safety in healthcare: the past decade and the next. *Healthc Q*. 2014;**17 Spec No**:45-50.

Russell L, Dawda P. Lessons for the Australian healthcare system from the Berwick report. *Aust Health Rev*. 2014;**38**(1):106-8.

Berg M, Black G. A Canadian perspective on clinical governance. *Clinical Governance: An International Journal*. 2014;**19**(4):314-21.

van Beuzekom M, Boer F, Akerboom S, Dahan A. Perception of patient safety differs by clinical area and discipline. *Br J Anaesth*. 2013;**110**(1):107-14.

Levi M. Towards a hospital-wide integrated system for quality and safety of health care. Neth J Med. 2013;**71**(1):2-3.

Marsden J, van Dijk M, Doris P, Krause C, Cochrane D. Improving care for British Columbians: the critical role of physician engagement. *Healthc Q*. 2012;**15 Spec No**:51-5.

Krause C, Cochrane D. BC Patient Safety & Quality Council: using network and social movement theory to improve healthcare. *Healthc Manage Forum*. 2012;**25**(4):181-4.

Ilan R, Squires M, Panopoulos C, Day A. Increasing patient safety event reporting in 2 intensive care units: a prospective interventional study. *J Crit Care*. 2011;**26**(4):431.e11-8.

Green E, White R, Janes K, Fields A, Easty A. Courage, collaboration, complexity and chemotherapy safety: the view from the sharp end. *Canadian Oncology Nursing Journal*. 2011;**21**(2):81-90.

Duckers ML, Wagner C, Vos L, Groenewegen PP. Understanding organisational development, sustainability, and diffusion of innovations within hospitals participating in a multilevel quality collaborative. *Implement Sci*. 2011;**6**:18.

van Gaal BG, Schoonhoven L, Hulscher ME, Mintjes JA, Borm GF, Koopmans RT, et al. The design of the SAFE or SORRY? study: a cluster randomised trial on the development and testing of an evidence based inpatient safety program for the prevention of adverse events. *BMC Health Serv Res*. 2009;**9**:58.

Relihan E, Silke B, O'Grady F. Internally-developed electronic reporting system for medication errors. *Ir Med J*. 2009;**102**(7):223-4.

For peer review only - http://bmjopen.bmj.com/site/about/guidelines.xhtml

Michel J, Nghiem HD, Jackson TJ. Using ICD-10-AM codes to characterise hospital-acquired complications. *Health Inf Manag*. 2009;**38**(3):18-25.

Neudorf K, Dyck N, Scott D, Davidson Dick D. Nursing education: a catalyst for the patient safety movement. *Healthc Q*. 2008;**11**(3 Spec No.):35-9.

Makeham MA, Saltman DC, Kidd MR. Lessons from the TAPS study--recall and reminder systems. *Aust Fam Physician*. 2008;**37**(11):923-4.

Makeham MA, Cooper C, Kidd MR. Lessons from the TAPS study - message handling and appointment systems. *Aust Fam Physician*. 2008;**37**(6):438-9.

Duckett S, Daniels S, Kamp M, Stockwell A, Walker G, Ward M. Pay for performance in Australia: Queensland's new Clinical Practice Improvement Payment. *Journal of Health Services Research and Policy*. 2008;**13**(3):174-7.

Booth BJ, Snowdon T, Harris MF, Tomlins R. Safety and quality in primary care: The view from general practice. *Australian Journal of Primary Health*. 2008;**14**(2):19-27.

David U. Medication safety... Your call to make a difference. *Alta RN*. 2007;**63**(8):10-1.

Runciman WB, Williamson JA, Deakin A, Benveniste KA, Bannon K, Hibbert PD. An integrated framework for safety, quality and risk management: an information and incident management system based on a universal patient safety classification. *Qual Saf Health Care*. 2006;**15**(Suppl 1):i82-90.

Greenberg A, Kramer S, Welch V, O'Sullivan E, Hall S. Cancer Care Ontario's computerized physician order entry system: a province-wide patient safety innovation. *Healthc Q*. 2006;**9 Spec No**:108-13.

Yassi A, Hancock T. Patient safety--worker safety: building a culture of safety to improve healthcare worker and patient well-being. *Healthc Q*. 2005;**8 Spec No**:32-8.

Greenall J, U D, Lam R. An effective tool to enhance a culture of patient safety and assess the risks of medication use systems. *Healthc Q*. 2005;**8 Spec No**:53-8.

Court D. Quest for patient safety in a challenging environment. *Aust N Z J Obstet Gynaecol*. 2003;**43**(2):97-100.

Nicklin W, McVeety JE. Canadian nurses' perceptions of patient safety in hospitals. *Can J Nurs Leadersh*. 2002;**15**(3):11-21.

For peer review only - http://bmjopen.bmj.com/site/about/guidelines.xhtml

For peer review only

Baldwin I, Beckman U, Shaw L, Morrison A. Australian Incident Monitoring Study in intensive care: local unit review meetings and report management. *Anaesth Intensive Care*. 1998;**26**(3):294-7.

Excluded on language (n=1)

Broeke RT, Lammers R, Ackerman E. The safety management system for medication contributes to a 'safe culture' in the hospital: The pharmacist participates in the medical round at the nursing department. *Pharmaceutisch Weekblad*. 2008;**143**(17):30-2.

Excluded on publication type (n=2)

Hanskamp-Sebregts M, Zegers M, Boeijen W, Westert GP, van Gurp PJ, Wollersheim H. Effects of auditing patient safety in hospital care: design of a mixed-method evaluation. BMC Health Serv Res. 2013;13:226.

Barker A, Mengersen K, Morton A. What is the value of hospital mortality indicators, and are there ways to do better? Aust Health Rev. 2012;36(4):374-7.

Excluded on setting (n=2)

Masotti P, Green M, McColl MA. Adverse events in community care: implications for practice, policy and research. *Healthc Q*. 2009;**12**(1):69-76.

Lang A, Edwards N, Fleiszer A. Safety in home care: a broadened perspective of patient safety. *Int J Qual Health Care*. 2008;**20**(2):130-5.

List of 'thin' studies

Australia

Abou Elnour A, Hernan AL, Ford D, Clark S, Fuller J, Johnson JK, et al. Surveyors' perceptions of the impact of accreditation on patient safety in general practice. *Med J Aust*. 2014;**201**(3):S56-S9. https://dx.doi.org/10.5694/mja14.00198

Adamson L, Beldham-Collins R, Sykes J, Thwaites D. Evaluating incident learning systems and safety culture in two radiation oncology departments. *J Med Radiat Sci*. 2022;**69**(2):208-17. https://dx.doi.org/10.1002/jmrs.563

Adie K, Fois RA, McLachlan AJ, Chen TF. Medication incident recovery and prevention utilising an Australian community pharmacy incident reporting system: the QUMwatch study. *Eur J Clin Pharmacol*. 2021;**77**(9):1381-95. https://dx.doi.org/10.1007/s00228-020-03075-9

For peer review only - http://bmjopen.bmj.com/site/about/guidelines.xhtml

For peer review only

Avery MJ, Cripps AW, Rogers GD. Health boards' governance of quality and risk: quality improvement agenda for the board. *Int J Health Gov*. 2021;**26**(3):292-306. https://dx.doi.org/10.1108/IJHG-01-2021-0006

Braithwaite J, Westbrook MT, Mallock NA, Travaglia JF, Iedema RA. Experiences of health professionals who conducted root cause analyses after undergoing a safety improvement programme. *Qual Saf Health Care*. 2006;**15**(6):393-9. https://dx.doi.org/10.1136/qshc.2005.017525

Braithwaite J, Westbrook MT, Travaglia JF, Iedema R, Mallock NA, Long D, et al. Are health systems changing in support of patient safety? A multi-methods evaluation of education, attitudes and practice. *Int J Health Care Qual Assur*. 2007;**20**(7):585-601. https://dx.doi.org/10.1108/09526860710822725

Braithwaite J, Westbrook M, Travaglia J. Attitudes toward the large-scale implementation of an incident reporting system. *Int J Qual Health Care*. 2008;**20**(3):184-91. https://dx.doi.org/10.1093/intqhc/mzn004

Brand C, Ibrahim J, Bain C, Jones C, King B. Engineering a safe landing: engaging medical practitioners in a systems approach to patient safety. *Intern Med J*. 2007;**37**(5):295-302. https://dx.doi.org/10.1111/j.1445-5994.2007.01310.x

Canaway R, Bismark M, Dunt D, Kelaher M. Medical directors' perspectives on strengthening hospital quality and safety. *J Health Organ Manag*. 2017;**31**(7-8):696-712. https://dx.doi.org/10.1108/JHOM-05-2017-0109

Canaway R, Bismark M, Dunt D, Kelaher M. Public reporting of hospital performance data: views of senior medical directors in Victoria, Australia. *Aust Health Rev*. 2018;**42**(5):591-9. https://dx.doi.org/10.1071/AH17120

Clay-Williams R, Taylor N, Ting HP, Arnolda G, Winata T, Braithwaite J. Do quality management systems influence clinical safety culture and leadership? A study in 32 Australian hospitals. *Int J Qual Health Care*. 2020;**32**(Supplement_1):60-6. https://dx.doi.org/10.1093/intqhc/mzz107

Clay-Williams R, Taylor N, Ting HP, Winata T, Arnolda G, Austin E, et al. The relationships between quality management systems, safety culture and leadership and patient outcomes in Australian Emergency Departments. *Int J Qual Health Care*. 2020;**32**(Supplement_1):43-51. https://dx.doi.org/10.1093/intqhc/mzz105

Graudins LV, Dooley MJ. Medication safety: Experiential learning for pharmacy students and staff in a hospital setting. *Pharmacy*. 2016;**4**(4):17. https://dx.doi.org/10.3390/pharmacy4040038

Greenfield D, Hinchcliff R, Banks M, Mumford V, Hogden A, Debono D, et al. Analysing 'big picture' policy reform mechanisms: the Australian health service safety and quality accreditation scheme. *Health Expect*. 2015;**18**(6):3110-22. https://dx.doi.org/10.1111/hex.12300

For peer review only - http://bmjopen.bmj.com/site/about/guidelines.xhtml

Hanley E, Quoyle C. Approaches to surveillance of staphylococcus aureus bacteraemia and clostridium difficile infection in Australian states and territories. *Healthc Infect*. 2014;**19**(4):141-6. https://dx.doi.org/10.1071/HI14019

Hughes CF, Mackay P. Sea change: Public reporting and the safety and quality of the Australian health care system. *Med J Aust*. 2006;**184**(10S):S44-S7. https://dx.doi.org/10.5694/j.1326-5377.2006.tb00361.x

Johnson A, Clay-Williams R, Lane P. Framework for better care: reconciling approaches to patient safety and quality. *Aust Health Rev*. 2019;**43**(6):653-5. https://dx.doi.org/10.1071/AH18050

Jones D. Improving clinical leadership in patient safety: from strategic principle to active participation -- the Western Australian approach. *Int J Clin Leadersh*. 2008;**16**(3):157-62.

Leggat SG, Balding C. Effective quality systems: implementation in Australian public hospitals. *Int J Health Care Qual Assur*. 2018;**31**(8):1044-57. https://dx.doi.org/10.1108/IJHCQA-02-2017-0037

Lucas J, Leggat SG, Taylor NF. Association between use of clinical governance systems at the frontline and patient safety: a pre-post study. *Int J Health Gov*. 2022;**27**(3):282-95. https://dx.doi.org/10.1108/IJHG-02-2022-0023

Ong N, Lucien A, Long J, Weise J, Burgess A, Walton M. What do parents think about the quality and safety of care provided by hospitals to children and young people with an intellectual disability? A qualitative study using thematic analysis. *Health Expect*. 2023;**27**(1):e13925. https://dx.doi.org/10.1111/hex.13925

Pain C, Green M, Duff C, Hyland D, Pantle A, Fitzpatrick K, et al. Between the flags: implementing a safety-net system at scale to recognise and manage deteriorating patients in the New South Wales Public Health System. *Int J Qual Health Care*. 2017;**29**(1):130-6. https://dx.doi.org/10.1093/intqhc/mzw132

Rubin GL, Leeder SR. Health care safety: what needs to be done? *Med J Aust*. 2005;**183**(10):529-31. https://dx.doi.org/10.5694/j.1326-5377.2005.tb07155.x

Runciman WB. Lessons from the Australian Patient Safety Foundation: setting up a national patient safety surveillance system--is this the right model? *Qual Saf Health Care*. 2002;**11**(3):246-51. https://dx.doi.org/10.1136/qhc.11.3.246

Sinni SV, Wallace EM, Cross WM. Perinatal staff perceptions of safety and quality in their service. *BMC Health Serv Res*. 2014;**14**:591. https://dx.doi.org/10.1186/s12913-014-0591-4

For peer review only - http://bmjopen.bmj.com/site/about/guidelines.xhtml

Spigelman AD, Swan J. Review of the Australian incident monitoring system. *ANZ J Surg*. 2005;**75**(8):657-61. https://dx.doi.org/10.1111/j.1445-2197.2005.03482.x

Spigelman AD, Rendalls S. Clinical governance in Australia. *Clin Gov*. 2015;**20**(2):56-73. https://doi.org/10.1108/CGIJ-03-2015-0008

Stark HE, Graudins LV, McGuire TM, Lee CYY, Duguid MJ. Implementing a sustainable medication reconciliation process in Australian hospitals: The World Health Organization High 5s project. *Res Social Adm Pharm*. 2020;**16**(3):290-8. https://doi.org/10.1016/j.sapharm.2019.05.011

Stride P, Seleem M, Nath N, Horne A, Kapitsalas C. Integration of patient safety systems in a suburban hospital. *Aust Health Rev*. 2012;**36**(4):359-62. https://doi.org/10.1071/AH11099

Taitz J, Genn K, Brooks V, Ross D, Ryan K, Shumack B, et al. System-wide learning from root cause analysis: a report from the New South Wales Root Cause Analysis Review Committee. *Qual Saf Health Care*. 2010;**19**(6):e63. https://doi.org/10.1136/qshc.2008.032144

Taylor KA, Durrheim DN, Merritt T, Massey P, Ferguson J, Ryan N, et al. Multidisciplinary analysis of invasive meningococcal disease as a framework for continuous quality and safety improvement in regional Australia. *BMJ Open Qual*. 2018;**7**(1):e000077. https://doi.org/10.1136/bmjoq-2017-000077

Travaglia JF, Westbrook MT, Braithwaite J. Implementation of a patient safety incident management system as viewed by doctors, nurses and allied health professionals. *Health*. 2009;**13**(3):277-96. https://doi.org/10.1177/1363459308101804

Westbrook MT, Braithwaite J, Travaglia JF, Long D, Jorm C, Iedema RA. Promoting safety: longer-term responses of three health professional groups to a safety improvement programme. Int *J Health Care Qual Assur*. 2007;**20**(7):555-71. https://doi.org/10.1108/09526860710822707

Australia & Canada

Arah OA, Klazinga NS. How safe is the safety paradigm? *Qual Saf Health Care*. 2004;**13**(3):226-32. https://doi.org/10.1136/qshc.2003.007070

Cheng L, Sun N, Li Y, Zhang Z, Wang L, Zhou J, et al. International comparative analyses of incidents reporting systems for healthcare risk management. *J Evid Based Med*. 2011;**4**(1):32-47. https://doi.org/10.1111/j.1756-5391.2011.01119.x

Australia & New Zealand

Adamson L, Beldham-Collins R, Sykes J, Thwaites D. Safety culture and incident learning systems in radiation oncology: Staff perceptions across Australia and New Zealand. *J Med Imaging Radiat Oncol*. 2022;**66**(2):299-309. https://doi.org/10.1111/1754-9485.13335

For peer review only - http://bmjopen.bmj.com/site/about/guidelines.xhtml

Harrison R, Lee H, Sharma A. A survey of the impact of patient adverse events and near misses on anaesthetists in Australia and New Zealand. *Anaesth Intensive Care*. 2018;**46**(5):510-5. https://doi.org/10.1177/0310057X1804600513

Canada

Baker RG. Governance, policy and system-level efforts to support safer healthcare. *Healthc Q*. 2014;**17 Spec no**:21-6. https://doi.org/10.12927/hcq.2014.23955

Beard P, Smyrski L. Reporting for learning and improvement: the Manitoba and Saskatchewan experience. *Healthc Q*. 2006;**9**:61-4. https://doi.org/10.12927/hcq..18461

Beesoon S, Robert J, White J. Surgery Strategic Clinical Network: Improving quality, safety and access to surgical care in Alberta. *CMAJ*. 2019;**191**:S27-S9. https://doi.org/10.1503/cmaj.190590

Chartier LB, Ovens H, Hayes E, Davis B, Calder L, Schull M, et al. Improving quality of care through a mandatory provincial audit program: Ontario's emergency department return visit quality program. *Ann Emerg Med*. 2021;**77**(2):193-202. https://doi.org/10.1016/j.annemergmed.2020.09.449

Cochrane D, Taylor A, Miller G, Hait V, Matsui I, Bharadwaj M, et al. Establishing a provincial patient safety and learning system: pilot project results and lessons learned. *Healthc Q*. 2009;**12**:147-53. https://doi.org/10.12927/hcq.2009.20717

Cowell J, Harvie M. HQCA: building a credible, transparent, and independent healthcare quality and safety organization in Alberta. *Healthc Manage Forum*. 2012;**25**(4):185-7. https://doi.org/10.1016/j.hcmf.2012.09.011

Espin S, D'Arpino M, Indar A, Gross M. Incident management in health care: A pan-Canadian perspective. *J Nurs Care Qual*. 2022;**37**(1):E15-E21. https://doi.org/10.1097/NCQ.0000000000000572

Hewitt T, Chreim S, Forster A. Sociocultural factors influencing incident reporting among physicians and nurses: understanding frames underlying self- and peer-reporting practices. *J Patient Saf*. 2017;**13**(3):129-37. https://doi.org/10.1097/PTS.0000000000000130

Jeffs L, Baker GR, Taggar R, Hubley P, Richards J, Merkley J, et al. Attributes and actions required to advance quality and safety in hospitals: Insights from nurse executives. *Nurs Leadersh*. 2018;**31**(2):20-31. https://doi.org/10.12927/cjnl.2018.25606

Jeffs L, Bruno F, Zeng RL, Schonewille N, Kinder K, De Souza G, et al. Integrating implementation science in a quality and patient safety improvement learning collaborative: Essential ingredients and impact. *Jt Comm J Qual Patient Saf*. 2023;**49**(5):255-64. https://doi.org/10.1016/j.jcjq.2023.02.001

For peer review only - http://bmjopen.bmj.com/site/about/guidelines.xhtml

Kassam A, Sharma N, Harvie M, O'Beirne M, Topps M. Patient safety principles in family medicine residency accreditation standards and curriculum objectives: Implications for primary care. *Can Fam Physician*. 2016;**62**(12):e731-e9.

Law MP, Zimmerman R, Baker GR, Smith T. Assessment of safety culture maturity in a hospital setting. *Healthc Q*. 2010;**13**:110-5. https://doi.org/10.12927/hcq.2010.21975

Milligan C, Allin S, Farr M, Farmanova E, Peckham A, Byrd J, et al. Mandatory reporting legislation in Canada: improving systems for patient safety? *Health Econ Policy Law*. 2021;**16**(3):355-70. https://doi.org/10.1017/S1744133121000050

Milne JK, Lalonde AB. Patient safety in women's health-care: professional colleges can make a difference. The Society of Obstetricians and Gynaecologists of Canada MORE(OB) program. *Best Pract Res Clin Obstet Gynaecol*. 2007;**21**(4):565-79. https://doi.org/10.1016/j.bpobgyn.2007.01.013

Mitchell JI, Izad Shenas SA, Kuziemsky C. Governance standards: A roadmap for increasing safety at care transitions. *Healthc Manage Forum*. 2015;**28**(1):28-33. https://doi.org/10.1177/0840470414551894

Nicklin W, Mass H, Affonso DD, O'Connor P, Ferguson-Paré M, Jeffs L, et al. Patient safety culture and leadership within Canada's Academic Health Science Centres: towards the development of a collaborative position paper. Nurs Leadersh. 2004;**17**(1):22-34. https://doi.org/10.12927/cjnl.2004.16243

O'Beirne M, Reid R, Zwicker K, Sterling P, Sokol E, Flemons W, et al. The costs of developing, implementing, and operating a safety learning system in community practice. *J Patient Saf*. 2013;**9**(4):211-8. https://doi.org/10.1097/PTS.0000000000000052

O'Beirne M, Sterling PD. Medical safety and community practice: necessary elements and barriers to implement a safety learning system. *Healthc Q*. 2009;**12**:141-6. https://doi.org/10.12927/hcq.2009.20982

O'Beirne M, Sterling PD, Zwicker K, Hebert P, Norton PG. Safety incidents in family medicine. *BMJ Qual Saf*. 2011;**20**(12):1005-10. 10.1136/bmjqs-2011-000105

Skutezky T, Small SS, Peddie D, Balka E, Hohl CM. Beliefs and perceptions of patient safety event reporting in a Canadian Emergency Department: a qualitative study. *CJEM*. 2022;**24**(8):867-75. https://doi.org/10.1007/s43678-022-00400-2

Weaver RR. Seeking high reliability in primary care: Leadership, tools, and organization. *Health Care Manage Rev*. 2015;**40**(3):183-92. https://doi.org/10.1097/HMR.0000000000000022

For peer review only - http://bmjopen.bmj.com/site/about/guidelines.xhtml

Williams A. Nursing informaticians address patient safety to improve usability of health information technologies. *Stud Health Technol Inform*. 2019;**257**:501-7.

Zecevic AA, Ho-Ting Li A, Ngo C, Halligan M, Kothari A, Li AH-T. Improving safety culture in hospitals: Facilitators and barriers to implementation of Systemic Falls Investigative Method (SFIM). *Int J Qual Health Care*. 2017;**29**(3):371-7. https://doi.org/10.1093/intqhc/mzx034

Ireland

Flynn MA, Burgess T, Crowley P. Supporting and activating clinical governance development in Ireland: sharing our learning. *J Health Organ Manag*. 2015;**29**(4):455-81. https://doi.org/10.1108/JHOM-03-2014-0046

O'Connor P, O'Malley R, Kaud Y, St Pierre E, Dunne R, Byrne D, et al. A scoping review of patient safety research carried out in the Republic of Ireland. *Irish J Med Sci*. 2023;**192**(1):1-9. https://doi.org/10.1007/s11845-022-02930-1

Netherlands

Bilijam CW. Patient safety in general practice. *Tijdschrift voor Kindergeneeskunde*. 2014;**82**(5):155-9.

Cords CI, van der Vlies CH, Stoop M, Nieuwenhuis MK, Boudestein K, Mattace-Raso FUS, et al. Frailty screening practice in specialized burn care-a retrospective multicentre cohort study. *Eur Burn J*. 2023;**4**(1):87-100. https://doi.org/10.3390/ebj4010009

de Korne DF, Van Wijngaarden JDH, Van Dyck C, Hiddema UF, Klazinga NS. Evaluation of aviation-based safety team training in a hospital in The Netherlands. *J Health Organ Manag*. 2014;**28**(6):731-53. https://doi.org/10.1108/jhom-01-2013-0008

Harmsen M, Gaal S, van Dulmen S, de Feijter E, Giesen P, Jacobs A, et al. Patient safety in Dutch primary care: a study protocol. *Implement Sci*. 2010;**5**:50. https://doi.org/10.1186/1748-5908-5-50 Heim N, van Fenema EM, Weverling-Rijnsburger AW, Tuijl JP, Jue P, Oleksik AM, et al. Optimal screening for increased risk for adverse outcomes in hospitalised older adults. *Age Ageing*. 2015;**44**(2):239-44. https://doi.org/10.1093/ageing/afu187

Hermans MPJ, Eindhoven DC, van Winden LAM, de Grooth GJ, Blauw GJ, Muller M, et al. Frailty score for elderly patients is associated with short-term clinical outcomes in patients with ST-segment elevated myocardial infarction treated with primary percutaneous coronary intervention. *Neth Heart J*. 2019;**27**(3):127-33. https://doi.org/10.1007/s12471-019-1240-7

Jepma P, Verweij L, Tijssen A, Heymans MW, Flierman I, Latour CHM, et al. The performance of the Dutch Safety Management System frailty tool to predict the risk of readmission or mortality in older hospitalised cardiac patients. *BMC Geriatr*. 2021;**21**(1):299. https://doi.org/10.1186/s12877-021-02243-5

For peer review only - http://bmjopen.bmj.com/site/about/guidelines.xhtml

Jessurun JG, Hunfeld NGM, Van Rosmalen J, Van Dijk M, Van Den Bemt P. Effect of automated unit dose dispensing with barcode scanning on medication administration errors: an uncontrolled before-and-after study. *Int J Qual Health Care*. 2021;**33**(4):13. https://doi.org/10.1093/intqhc/mzab142

Oud FMM, Schut MC, Spies PE, van der Zaag-Loonen HJ, de Rooij SE, Abu-Hanna A, et al. Interaction between geriatric syndromes in predicting three months mortality risk. *Arch Gerontol Geriatr*. 2022;**103:**104774. https://doi.org/10.1016/j.archger.2022.104774

Oud FMM, Wolzak NK, Spies PE, Zaag-Loonen HJV, van Munster BC. The predictive value of the 'VMS frail older patients' for adverse outcomes in geriatric inpatients. *Arch Gerontol Geriatr*. 2021;**97**:104514. https://doi.org/10.1016/j.archger.2021.104514

Schuijt HJ, Oud FMM, Bruns EJR, van Duijvendijk P, Van der Zaag-Loonen HJ, Spies PE, et al. Does the Dutch Safety Management Program predict adverse outcomes for older patients in the emergency department? *Neth J Med*. 2020;**78**(5):244-50.

Snijders BMG, Emmelot-Vonk MH, Souwer ETD, Kaasjager HAH, van den Bos F. Prognostic value of screening instrument based on the Dutch national VMS guidelines for older patients in the emergency department. *Eur Geriatr Med*. 2021;**12**(1):143-50. https://doi.org/10.1007/s41999-020-00385-0

Snijders C. Patient safety in the neonatal intensive care unit: A look back on the NEOSAFE study. *Tijdschrift voor Kindergeneeskunde*. 2014;**82**(5):160-5.

van Dam CS, Trappenburg MC, Ter Wee MM, Hoogendijk EO, de Vet HC, Smulders YM, et al. The accuracy of four frequently used frailty instruments for the prediction of adverse health outcomes among older adults at two Dutch emergency departments: Findings of the AmsterGEM study. *Ann Emerg Med*. 2021;**78**(4):538-48. https://doi.org/10.1016/j.annemergmed.2021.04.027

van der Starre, C., van Dijk, M. & Tibboel, D. Real-time registration of adverse events in Dutch hospitalized children in general pediatric units: first experiences. *Eur J Pediatr.* 2012;**171**:553–558. https://doi.org/10.1007/s00431-011-1608-1

van der Zanden V, Paarlberg KM, van der Zaag-Loonen HJ, Meijer WJ, Mourits MJE, van Munster BC. Risk assessment for postoperative outcomes in a mixed hospitalized gynecological population by the Dutch safety management system (Veiligheidsmanagementsysteem, VMS) screening tool 'frail elderly'. *Arch Gynecol Obstet*. 2021;**304**(2):465-73. https://doi.org/10.1007/s00404-021-06073-z

van Munster BC, Drost D, Kalf A, Vogtlander NP. Discriminative value of frailty screening instruments in end-stage renal disease. *Clin Kidney J*. 2016;**9**(4):606-10. https://doi.org/10.1093/ckj/sfw061

For peer review only - http://bmjopen.bmj.com/site/about/guidelines.xhtml

van Schoten SM, Baines RJ, Spreeuwenberg P, de Bruijne MC, Groenewegen PP, Groeneweg J, et al. The ecometric properties of a measurement instrument for prospective risk analysis in hospital departments. *BMC Health Serv Res*. 2014;**14**:103. https://doi.org/10.1186/1472-6963-14-103

Verstappen W, Gaal S, Bowie P, Parker D, Lainer M, Valderas JM, et al. A research agenda on patient safety in primary care. Recommendations by the LINNEAUS collaboration on patient safety in primary care. *Eur J Gen Pract*. 2015;**21**(sup1):72-7. https://doi.org/10.3109/13814788.2015.1043726

Warnier RMJ, van Rossum E, van Kuijk SMJ, Magdelijns F, Schols J, Kempen G. Frailty screening in hospitalised older adults: How does the brief Dutch National Safety Management Program perform compared to a more extensive approach? *J Clin Nurs*. 2020;**29**(7-8):1064-73. https://doi.org/10.1111/jocn.15148

Weske U, Boselie P, van Rensen E, Schneider M. Physician compliance with quality and patient safety regulations: The role of perceived enforcement approaches and commitment. *Health Serv Manage Res*. 2019;**32**(2):103-12. https://doi.org/10.1177/0951484818813324

Weske U, Boselie P, van Rensen ELJ, Schneider MME. Using regulatory enforcement theory to explain compliance with quality and patient safety regulations: the case of internal audits. *BMC Health Serv Res*. 2018;**18**(1):62. https://doi.org/10.1186/s12913-018-2865-8

New Zealand

Raymont A, Graham P, Hider PN, Finlayson MP, Fraser J, Cumming JM. Variation in the adoption of patient safety practices among New Zealand district health boards. *Aust Health Rev*. 2012;**36**(2):163-8. https://doi.org/10.1071/AH10972

Robin G, Horsburgh S. Healthcare professional perspectives on quality and safety in New Zealand public hospitals: findings from a national survey. *Aust Health Rev*. 2014;**38**(1):109-14. https://doi.org/10.1071/AH13116

Shuker C, Bohm G, Bramley D, Frost S, Galler D, Hamblin R, et al. The Health Quality and Safety Commission: making good health care better. *N Z Med J*. 2015;**128**(1408):97-109.

Mobius Research & Strategy. The Health Quality & Safety Commission Surgical Safety Culture Survey Research Report. 2017. URL: https://www.hqsc.govt.nz/assets/Our-work/Improved-service-delivery/Safe-surgery/Publications-resources/2017_Surgical_Safety_Culture_Survey_Report_-_FINAL.pdf (accessed 24 April 2024).

For peer review only - http://bmjopen.bmj.com/site/about/guidelines.xhtml

Supplementary material 4: Rich and descriptive studies

Documents relating to patient safety and/or health services in each country included in the review

**Document**	**Year of publication**	**Description of document**
**Netherlands**		
Policy		
NTA 8009 'Safety management system for hospitals and institutions that provide hospital care' (initial technical agreement).[29*]	2007	Describes the basic requirements for an SMS for hospitals and institutions that provide hospital care. It focuses on managing risks and reducing harm to patients. It is based on the Rein Willems report '*Here you work safely or you don't work here'* (2004) and the improvement cycle 'plan-do-check-act' on which other SMS are based. The structure of the document includes: 1. Subject and scope, 2. Terms and definitions, 3. Responsibilities of the Board of Directors, and 4. Basic requirements for the SMS. Additional information is provided in the appendices on the organisations involved in the drafting of the document; the reporting matrix (roles and responsibilities for reporting different types of incidents such as near misses and serious incidents); conversion tables that explain how the NTA relates to other standards (e.g. ISO 9001: 2000 Quality Management System standard); examples of prospective and retrospective risk assessment; and safety culture (which is not explicitly included in the basic requirements).
NEN 8009 'Safety management system for hospitals and institutions that provide hospital care' (replaces NTA 8009 2014).[30*]	2018	This is the third update of the original NTA 8009 (following updates in 2011 and 2014). It supplements, without replacing, the original SMS requirements with Safety-II concepts that promote a proactive safety culture and encourage learning from daily activities with desired outcomes as well as incidents. It acknowledges that improvement could vary from continuous improvements in small steps to significant changes at organisational level and highlights the importance of encouraging employees and patients to play an active role, identify opportunities and successful practices and contribute to the overall process of organisational learning.

For peer review only - http://bmjopen.bmj.com/site/about/guidelines.xhtml

Other				
Bal R, Wagner C. The Netherlands: Patient safety in Dutch hospitals: How can we explain success, in Health systems improvement across the Globe: Success stories from 60 countries, eds. Braithwaite J et al. Boca Raton: CRC Press.[31]	2018	Provides an overview of the Dutch patient safety programme and tries to explain its success, accounting for the broader socio-cultural and historical context, and identifying the 'conditions under which such a programme was able to succeed' (p. 208). It argues that five such factors contributed to the success of the Dutch patient safety programme: experience in project work, market competition, public pressure, activities of regulatory agencies, and public reporting of adverse events and mortality.		
Bolk M. Final report: Embedding patient safety in education and training. Utrecht: VMS Safety Programme.[32]	2013	Reports on the '*Embedding patient safety in education and training*' project which aimed 'to support, facilitate and accelerate education for doctors, nurses and the supporting professions (medical and otherwise), with the help of a national incentive program and the corresponding support structure' (p. 4). As a result, a national 'patient safety competencies framework' was developed; all findings from the project were compiled in a digital portal and campaign website and a 'patient safety toolkit' was developed; examples for good patient safety education were collected; and the attention generated regarding patient safety resulted in the project linking to other educational initiatives already working on patient safety. The project made a number of recommendations for embedding patient safety in various educational and learning processes and programmes.		
Research		**Aims**	**Methods**	**Findings**
Baines R et al. How effective are patient safety initiatives? A retrospective patient record review study of changes to patient safety over time. *BMJ Qual Saf*. 2015;**24**(9):561-71.[33]	2015	To assess whether hospital care in 2011/12 was safer than in 2004 and 2008.	Retrospective patient record review. 15, 997 patient admissions were included, 7926 from 2004, 4023 from 2008 and 4048 from 2011/2012.	In comparison with 2008, uncorrected crude overall adverse event rates showed no change in 2011/2012 but preventable adverse event rates showed a reduction of 45%. After multilevel corrections, the

For peer review only

For peer review only - http://bmjopen.bmj.com/site/about/guidelines.xhtml

				decrease was still visible but not statistically significant.
de Blok C et al. Implementation of the Dutch National Patient Safety Programme (VMS Veiligheidsprogrammesma). Utrecht/Amsterdam: NIVEL/EMGO+.[34]	2013	English summary of de Blok[21] (see below).		
de Blok C, Koster E, Schilp J, Wagner C. Implementatie VMS Veiligheidsprogrammesma. Evaluatieonderzoek in Nederlandse ziekenhuizen. Utrecht / Amsterdam: NIVEL/EMGO+.[21*]	2013	To assess the extent to which the ten themes of the Dutch patient safety programme have been implemented and gain insight into the factors that influence the degree of implementation and compliance with the advice.	Data collection varied across themes and included observations and semi-structured interviews, review of patient records and registry data. A large proportion of Dutch hospitals were involved in the evaluation, which varied across themes.	Implementation objectives set in 2008 were almost achieved for some of the themes (e.g. 'Preventing renal insufficiency during intravascular use of iodinated contrast agent' and subtheme 'Treatment of severe sepsis'). There was a positive trend for other themes, but considerable variation was observed across and within hospitals. The study identified a range of organisational, staff- and theme-related factors associated with the success of implementation.
de Bruijne MC, Zegers M, Hoonhout LHF, Wagner C. Onbedoelde schade in Nederlandse ziekenhuizen: Dossieronderzoek van ziekenhuisopnames in 2004. Amsterdam: EMGO+ Instituut, Utrech: NIVEL[35*]	2007	To establish a baseline measurement in the field of patient safety.	Retrospective patient record review; 200 patient records from patients who were discharged and 200 from patients who died in hospitals in 2004 were randomly selected from each of the participating 21	Of all admissions: (i) 5.7% suffered unintentional healthcare-related harm, 5% of which experienced permanent harm and 8% died partly as a result of this; (ii) 2.3% suffered preventable adverse events. Of the patients who died in hospital,

For peer review only - http://bmjopen.bmj.com/site/about/guidelines.xhtml

			Dutch hospitals. Patients admitted to psychiatry and obstetrics and those less then one year of age were excluded as the assessment system was not suitable for such patients.	10.7% suffered unintentional healthcare-related events; of those, 4.1% were related to preventable adverse events during admission. It was estimated that in 2004, there were 1735 (95%CI 1482 to 2032) potentially preventable deaths in Dutch hospitals.
de Groot JF et al. Implementing paediatric early warning scores systems in the Netherlands: future implications. *BMC Pediatr*. 2018;**18**(1):128.[36]	2018	(i) To evaluate the implementation of paediatric early warning score systems in five hospitals.<br>(ii) To gain insight into the attitudes of professionals towards the system.	(i) Retrospective patient record review (n = 554) at the start, mid and endpoint of implementation.<br>(ii) Semi-structured interviews with professionals (n = 8).	(i) None of the hospitals started with a system but after 1 year, they were recorded in 69.2% of patient records and resulted in appropriate action in 49.1%.<br>(ii) Sustainable implementation might be hindered by staff concerns about validity, effectiveness and communication with other hospitals during transfers to higher levels of care.
Klopotowska J, Schutijser B, de Bruijne M, Wagner C. Tweede evaluatie van VMS Programma in Nederlandse ziekenhuizen op basis van vier veiligheidsthema's: aangevuld met EPD Scan naar mogelijkheden voor toekomstige metingen. Utrecht/Amsterdam: NIVEL/EMGO+.[37]*	2016	To investigate the progress of the implementation of four safety themes from the Dutch national patient safety programme compared to the first evaluation study in 2011/2012 and assess the possibility of automatic monitoring of process indicators from	Data collection:<br>(i) Theme 'medication verification': review of patient records (n=1060) in 15 hospitals.<br>(ii) Theme 'high risk medication': observations of nurses (n=349) in 15 hospitals.	There was improvement in compliance for (i) and (ii) but still the target of 100% was not met; authors note that 100% compliance may not be desirable as in some cases deviation from guidelines could be beneficial for the patient. For (iii) compliance increased by 34% (to 73% against 90% target). For (iv) hospital mortality among severe sepsis patients

For peer review only - http://bmjopen.bmj.com/site/about/guidelines.xhtml

		electronic patient records for two of the themes.	Themes (iii) 'prevention of wound infections after surgery' and (iv) 'prevention of line sepsis and treatment of severe sepsis': data from national registries.	has fallen from 34% to 29% (15% relative mortality reduction) but for line sepsis positive results were observed only for the ICU departments on which the hospitals focused initially (incidence was 2.5 times higher in non-ICU).
Langelaan M et al. Monitor zorggerelateerde schade 2008: Dossieronderzoek in Nederlandse ziekenhuizen. Utrecht: NIVEL, Amsterdam: EMGO+ Instituut.[38]*	2010	To estimate and characterise potentially avoidable adverse events and deaths, and those related to the use of medical technology; to compare them to 2004.	Retrospective patient record review: stratified samples of 100 admissions of discharged patients and 100 of patients who died in hospital were drawn from each of the 20 participating hospitals. Psychiatry, obstetrics and patients < 1 year of age were excluded.	2.9% potentially avoidable adverse events (of all admissions). The change from 2004 was not statistically significant; 0.8% was related to the use of medical technology. Potentially avoidable mortality was 5.5% (95%CI 4.5% to 6.6%); the change from 2004 was not statistically significant. In absolute numbers, in 2008 there were 1960 (95%CI 1600 to 2460) patients in whom potentially avoidable harm contributed to the death.
Langelaan M et al. Monitor Zorggerelateerde Schade 2011/2012. Dossieronderzoek in Nederlandse ziekenhuizen. Amsterdam: VU medisch centrum & EMGO+ Instituut, Utrecht: NIVEL.[39]*	2013	To estimate and characterise potentially avoidable adverse events and mortality in Dutch hospitals, including those related to medical technology, and compare them to previous measurements.	Retrospective patient record review study based on data from 20 randomly selected Dutch hospitals (>4000 records in total); random samples were drawn from each hospital of 100 discharged patients and 100 patients who died in the hospital. Psychiatry,	Of all patients admitted in 2011/2012, 1.6% (95% CI: 1.1% to 2.2%) experienced potentially avoidable harm: 30% and 45% less than in 2004 and 2008, respectively. Potentially avoidable mortality decreased by 37% and 53% compared to 2004 and 2008, respectively, which expressed in absolute

For peer review only - http://bmjopen.bmj.com/site/about/guidelines.xhtml

		To estimate the costs of healthcare-related harm and the quality of file management.	obstetrics and patients < 1 year of age were excluded.	numbers and extrapolated to all Dutch hospitals was approx. 1,735 patients (95% CI: 1,482 to 2,032) in 2004, 1,960 patients (95% CI: 1,600 to 2,360) in 2008, and 970 (95% CI: 738 to 1,274) patients between 1st April 2011 and 31st March 2012. Other findings included harms related to the application of medical technology, costs related to adverse events and the quality of patient records.
Langelaan M et al. Monitor Zorggerelateerde Schade 2015/2016: Dossieronderzoek bij overleden patiënten in Nederlandse ziekenhuizen. Utrecht: NIVEL, Amsterdam: EMGO+ Instituut.[40]*	2017	To estimate and characterise potentially avoidable adverse events and mortality in Dutch hospitals, including those related to medical technology, and compare them to previous measurements.	Retrospective patient record review: a random sample of 150 patients who died in hospital was drawn from each of the 19 hospitals, randomly selected to participate in the study. Psychiatry, obstetrics and patients < 1 year of age were excluded.	Of all deceased patients, 4.3% (95%CI 3.6% to 5.1%) suffered potentially preventable adverse events and 3.1% (95%CI 2.5% to 3.8%) suffered potentially preventable mortality. The difference with 2011/12 was not statistically significant.<br>The respective results related to the use of medical technology were 1.7% and 1.3%, out of all cases in which technology was used.
Meulman M, Merten H, Wagner C. Het screenen van kwetsbare ouderen: onderzoek vanuit een Safety-I en Safety-II perspectief: evaluatie van het VMS thema	2021	To investigate the degree of compliance with the national Dutch patient safety programme screening	Functional Resonance Analysis Method was used, based on analysis of hospital protocols, interviews with nurses (n=31), and a feedback meeting. Additionally, a	Hospital protocols were more detailed than the national guidelines but largely similar. There were actions in the protocols that were not performed, and actions that were performed but not included

For peer review only - http://bmjopen.bmj.com/site/about/guidelines.xhtml

Kwetsbare ouderen. Utrecht: NIVEL[41]*		tool for vulnerable older people.	survey of employees' resilience (n=146), an evaluation interview in each department, and compliance data (the percentage of admitted patients aged ≥70 who were screened). Data were collected between 2020-2021, from 10 departments in 6 hospitals.	in the protocols. An average of 74% of admitted patients were screened. Individual resilience (66%) was rated more positively than team resilience (49%).
Meurs M, de Groot JF. Veiligheidsmanagement systeem (VMS) thema's kindzorg Pijn en (Lijn)sepsis: verbeterpunten vanuit het werkveld voor verdere kwaliteitsverbetering. Utrecht: NIVEL. [42]*	2020	To identify bottlenecks and areas of improvement for implementation of the Dutch national patient safety programme themes '(line) sepsis' and 'pain' within paediatric care hospitals.	Working groups with paediatricians and paediatric nurses, and a survey (n=209). Data was collected in 2019.	Theme '(line) sepsis': the main recommendation regarded earlier recognition and treatment of sepsis (e.g. through training).<br>Theme 'pain': the main recommendation for improvement was to revise guidelines to adopt a holistic approach to pain recognition and treatment.
Sambeeck SJV et al. Pediatric Early Warning System Scores: Lessons to be learned. *J Pediatr Intensive* Care. 2018;**7**(1):27-32.[43]	2018	To investigate how many hospitals complied with the national recommendation to implement a paediatric early warning system score and if so, the score they chose to implement.	Cross-sectional electronic survey of Dutch hospitals in 2014 (n=91).	Three-quarters of Dutch hospitals with a paediatric department implemented a score. These hospitals used 45 different versions of a paediatric early warning system score, the majority of which were not validated.

For peer review only - http://bmjopen.bmj.com/site/about/guidelines.xhtml

Schlinkert C, Jelsma J, Wagner C. Patiëntveiligheidscultuur in Nederlandse Ziekenhuizen. Van openheid naar reflectie en veerkracht. Utrecht: NIVEL.[44]*	2021	To measure the patient safety culture in Dutch hospitals in 2020 and compare it with previous measurements (before and after the implementation of the Dutch patient safety programme). To measure the employees' resilience.	Cross-sectional survey on patient safety culture (Dutch version of Hospital Survey on Patient Safety Culture) and employees' resilience. Data were collected in 2020; 3014 employees from 5 hospitals completed the survey. Comparison with data collected using the same survey in 2005-2007 and 2012.	62.5% of respondents rated patient safety in their own department 'very good' or 'excellent'. Teamwork was the highest rated dimension, 'Adequate staffing', 'Support from management' and 'Collaboration between departments' were rated lowest. Nursing staff rated patient safety more negatively than other professional groups. More respondents considered patient safety to be 'very good' or 'excellent' in 2020 compared to previous measurements.
van Dijk LM, van Eikenhorst L, Muns L, Wagner C. Medicatieverificatie bij ontslag: nieuwe benadering vanuit een Safety-I en Safety-II perspectief: derde evaluatie van het VMS-thema. Utrecht: NIVEL.[45]*	2021	To investigate the implementation of the 'medication verification at discharge' theme of the Dutch national patient safety programme.	Functional Resonance Analysis Method was used, based on hospital protocols, interviews with nurses (n=77), and a feedback meeting. A survey of employees' resilience (n=142) and an evaluation interview in each department were carried out, and compliance data were collected (percentage of discharged patients aged ≥18 for whom medication verification took place upon discharge). Data was collected between 2020-	Hospital protocols were more detailed than the national guidelines. There were variations in how the processes were carried out in practice, normally in response to circumstances in the work environment (e.g. IT problems, staff shortages). Compliance rates varied between 67%-100% across departments over the data collection period. Both individual (64%) and team (69%) resilience was rated positively.

For peer review only - http://bmjopen.bmj.com/site/about/guidelines.xhtml

			2021, from 9 departments in 8 hospitals (4 cardiology departments and 5 orthopaedics departments).	
van der Starre C et al. Paediatric critical incident analysis: lessons learnt on analysis, recommendations and implementation. *Eur J Pediatr*. 2014;**173**(11):1449-57.[46]	2014	To identify potential causal and contributing factors of serious paediatric patient safety incidents, and to report on and assess the extent of implementation of recommendations for improvement.	Retrospective review of critical incident analyses (n=17) from 2005–2010.	A median number of 5 causal and contributing factors was identified per incident and were mostly likely to be team or task-related. There was a median of 5 recommendations per analysis, only one-third of recommendations were implemented.
van Noord N, Zwijnenberg I, Wagner C. Patiëntveiligheidscultuur in Nederlandse Ziekenhuizen. Een stap in de goede richting. Utrecht: NIVEL.[47]*	2013	To examine whether the Dutch national patient safety programme improved patient safety culture.	Comparison of two cross-sectional safety culture surveys (Dutch version of Hospital Survey on Patient Safety Culture). Between 2005-2007, 3779 healthcare providers in 45 hospitals responded, in 2012, 6605 healthcare providers in 24 hospitals responded.	Patient safety culture improved between 2005-2007 and 2012, with 48.8% of respondents rating it 'very good' or 'excellent' in 2012. However, there was variation between hospitals and departments. 'Teamwork' was the highest rated dimension of safety; 'Collaboration between departments', 'Reporting frequency' and 'Support from management' were lowest rated. Nursing staff rated patient safety more negatively than other professionals.
van Schoten S et al. The association between quality system development stage and	2018	To examine the association between the development stage	Analysis of a national survey on the development stage of hospital quality	No association was found between the development stage of a hospital quality system and

For peer review only - http://bmjopen.bmj.com/site/about/guidelines.xhtml

the implementation of process-level patient safety themes in Dutch hospitals: an observational study. *BMC Health Serv Res.* 2018;**18**:189.[48]		of hospital quality systems and the process-level implementation of patient safety themes within the Dutch patient safety programme.	systems (conducted in 2011) and an evaluation study of the national Dutch patient safety program (2011-2012). Data from 30 hospitals (12,485 observations) were included.	the process-level implementation of patient safety themes.
van Schoten S et al. Monitor Zorggerelateerde Schade 2019: Dossieronderzoek bij overleden patiënten in Nederlandse ziekenhuizen. Utrecht: NIVEL, Amsterdam: APH.[49*]	2022	To estimate and characterise potentially avoidable adverse events and mortality in Dutch hospitals, including those related to medical technology and diagnostic processes, and those in vulnerable older patients, and compare them to previous measurements.<br><br>To assess the quality of file management.	Retrospective patient record review: a random sample of 150 patients who died in hospital was drawn from each of the 20 hospitals, randomly selected to participate in the study. Psychiatry, obstetrics and patients < 1 year of age were excluded.	14.6% (95%CI 13.3% to 16.0%) of patients who died in hospital suffered care-related harm. This is a significant increase compared to 2015/2016 (was 9.9% (95% CI 8.9% to 11.0%)). Potentially avoidable adverse events and mortality were 4.2% (95%CI 3.5% to 5.0%) and 3.1% (95% CI 2.5% to 3.9%), respectively, indicating that the change from the previous measurement was not statistically significant.
van Stralen S, Schutijser B, Vonk A, van Eikenhorst L, Wagner C. Tweede controle bij het klaarmaken en toedienen van high-risk medicatie: nieuwe benadering vanuit een Safety-I en Safety-II perspectief. Derde evaluatie van het VMS thema. Utrecht: NIVEL.[50*]	2021	To investigate the implementation of the 'high-risk medication' theme (specifically the second check of medication) of the Dutch national patient safety programme.	Functional Resonance Analysis Method was used, based on hospital protocols, interviews with nurses (n=77), and a feedback meeting. Additionally, a survey of employee resilience (n=142) and an evaluation interview in each	Compared to the national guidelines from the patient safety programme, hospital protocols were largely the same. There were variations in how nurses carried out the processes, normally in response to circumstances in the work environment (e.g. time pressures). Individual (average

For peer review only - http://bmjopen.bmj.com/site/about/guidelines.xhtml

			department were carried out, and compliance data collected. Data was collected between 2020-2021, from 10 departments in 9 hospitals (3 surgery departments, 3 internal medicine departments and 4 intensive care units).	score 3.21/9) and team resilience (average score 4.08/9) was rated highly.
Verbeek-van Noord I et al. A nation-wide transition in patient safety culture: a multilevel analysis on two cross-sectional surveys. *Int J Qual Health Care*. 2019;**31**(8):627–632.[51]	2019	To examine whether the Dutch national patient safety programme improved patient safety culture.	Comparison of two cross-sectional safety culture surveys (Dutch version of Hospital Survey on Patient Safety Culture). Between 2005-2007, 3779 healthcare providers in 45 hospitals responded, in 2012, 6605 healthcare providers in 24 hospitals responded.	Patient safety culture improved over the studied period. However, there was variation between hospitals and departments.
**Australia**				
Policy				
Australian Commission on Safety and Quality in Health Care. National model clinical governance framework.[63]	2017	This framework defines clinical governance as 'a set of relationships and responsibilities established by a health service organisation between its state or territory department of health, governing body, executive, workforce, patients, consumers and other stakeholders'.(p.iii) The framework contains all the elements that acute health service organisations need to implement integrated corporate and clinical governance systems, with the aim of ensuring good clinical outcomes through the delivery of safe and quality care. It is based on the National Safety and Quality Health Service Standards (NSQHS) Clinical Governance and Partnering with Consumers (see below) and contains five components: governance, leadership and culture, patient safety and quality		

For peer review only - http://bmjopen.bmj.com/site/about/guidelines.xhtml

		improvement systems, clinical performance and effectiveness, safe environment for the delivery of care, and partnering with consumers.		
Australian Commission on Safety and Quality in Health Care. National safety and quality health service standards. 2nd edition.[64]	2021	Describe standards intended for organisation-wide implementation in all hospital and day procedure services to enable the delivery of nationally consistent, safe and quality care. There are eight Standards - Clinical Governance, Partnering with Consumers, Preventing and Controlling Healthcare-Associated Infection, Medication Safety, Comprehensive Care, Communicating for Safety, Blood Management and Recognising and Responding to Acute Deterioration. The Clinical Governance and Partnering with Consumers Standards set out overarching requirements for the implementation of the other six Standards. The Standards do not specify how they should be implemented to allow for local adaptation.		
Australian Commission on Safety and Quality in Health Care. National safety and quality primary and community healthcare standards.[65]	2021	Describes a framework which is intended for use in all primary and community healthcare services. The processes and structures in the Standards are intended to enable the delivery of, and improve, the quality and safety of services. There are three Standards - Clinical Governance, Partnering with Consumers, and Clinical Safety – with the former two Standards setting out overarching requirements for the implementation of the Clinical Safety Standards.		
Research		**Aims**	**Methods**	**Findings**
Australian Commission on Safety and Quality in Health Care. Creating safer, better healthcare - the impact of national safety and quality health service standards.[66]	2018	To provide an overview of changes associated with implementation of the first edition of the NSQHS Standards, identifying areas where improvement has been made and where further work is needed.	Draws on a numbers of research projects, including surveys of hospital staff, hospital board members, and specialist medical colleges, National Inpatient Medication Chart Audits, and a Consumer Participation Study involving interviews and focus groups.	Implementation of the NSQHS Standards has resulted in the prevention of harm, improvements in patient care including empowerment of consumers and patients, development of better governance systems, and reduction of waste.

For peer review only

Australian Commission on Safety and Quality in Health Care. The state of patient safety and quality in Australian hospitals 2019.[67]	2019	To report on safety and quality in public hospitals in Australia, including common patient safety risks, action taken to deliver appropriate care, and the Commission's approach to supporting value-based healthcare.	Draws on data on public hospitals from a wide range of sources, such as the Admitted Patient Care National Minimum Data Set, 2013–14 to 2017–18 and the Australian Atlas of Healthcare Variation.	There have been significant improvements in the safety and quality of care over the last 10 years. These include improved patient outcomes such as reductions in hospital acquired infections and preventable in-hospital cardiac arrests, improved patient experience and involvement in care, and better governance of clinical care.
**Canada**				
Policy				
Canadian Patient Safety Institute. Canadian disclosure guidelines. Being open with patients and family.[68]	2011	'The guidelines are intended to encourage and support healthcare providers, interdisciplinary teams, organizations and regulatory authorities in developing and implementing disclosure policies, practices and training methods.' (p. 13) The following guiding principles underpin the development of the guidelines: patient-centred healthcare, patient autonomy, safe healthcare, leadership support, disclosure is the right thing to do, honesty and transparency. It recommends the following elements to be included in a disclosure policy: policy statement/objectives, definitions of key terms, provision for patient support, provision for healthcare provider support and education, the disclosure process, provision for particular circumstances (Appendix C).		
Canadian Patient Safety Institute. Strengthening commitment for improvement together. A policy framework for patient safety.[69]	2019	A policy framework for patient safety aiming to stimulate conversation and action on the following policy levers: regulations, standards, organisational policies and public engagement.		
Health Standards Organisation & Canadian Patient Safety Institute. The Canadian quality and patient	2020	Defines five areas - people-centred care, safe care, accessible care, appropriate care, and integrated care – for health services to focus on to provide safe and quality care. These goal areas are intended 'to drive improvement and to align Canadian legislation, regulations, standards, organizational policies, and public engagement on patient safety		

For peer review only - http://bmjopen.bmj.com/site/about/guidelines.xhtml

safety framework for health services.[70]		and quality improvement.'(p.3) The framework includes actions guides customised to five stakeholder groups - the public, health teams, health leaders, board members and policymakers - which identify objectives and outcomes for each goal, indicators to track success, and resources.		
Healthcare Excellence Canada. Status of patient safety incident legislation and best practices across Canada. Summary report.[71]	2021	Summarises and compares the status of four types of patient safety incident legislation - mandatory reporting, mandatory disclosure, apology protection, and quality assurance - in federal, provincial and territorial jurisdictions across Canada. The report also describes important characteristics of each type of legislation and comments on current or potential changes in policy direction which may be significant for the future development of legislation.		
Healthcare Excellence Canada, Patients for Patient Safety Canada. Rethinking patient safety. A discussion guide for patients, healthcare providers and leaders.[72]	2023	Summarises discussions with health service users, healthcare professionals, and safety scientists. Describes a new approach to patient safety in Canada, involving (i) the development of a positive culture of safety, with everyone contributing to creating safe conditions, (ii) acknowledging that the absence of harm does not mean that care is safe, and (iii) that all forms of harm matter, including those that are not physical.		
Research		**Aims**	**Methods**	**Findings**
Goldman J, Rotteau L. Evaluation research of Measurement and Monitoring of Safety Framework collaborative.[73]	2020	To (i) understand the effectiveness of a learning collaborative in teaching participants about the Measurement and Monitoring of Safety Framework (MMSF), and (ii) examine the implementation of the MMSF.	Qualitative study with three types of data collection:<br>(i) Interviews (n=36, with participants from teams including emergency, surgery, medicine, cardiology, psychiatry, supportive living and long-term care settings).<br>(ii) Observations of sites (n=5) where MMSF was being implemented and learning sessions and calls	Participants found the sessions to learn about MMSF useful, but some felt overwhelmed by the amount of information. Coaching was felt to play a key role in the implementation of MMSF in participants' organisations. A range of context-specific strategies were used by participants to implement MMSF (e.g. teaching to groups of stakeholders, integrating its language into usual communication, using it to

For peer review only

			where participants learnt about MMSF (29 hours were spent on site visits and 33 hours at a learning session).<br>(iii) Documentary data collection (e.g. learning session resources, tools being used in MMSF implementation).	inform activities such as safety huddles and incident reporting). Most participants reported positive impacts from MMSF implementation (e.g. changes in thinking about safety and resulting behaviour change); they supported its wider spread but found opportunities to do this beyond their sites were variable.
Goldman J, Rotteau L, Flintoft V, *et al* Measurement and Monitoring of Safety Framework: a qualitative study of implementation through a Canadian learning collaborative *BMJ Quality & Safety* 2023; **32:**470-478.[74]	2023	Journal article summarising the report[73] described above.		
**Ireland**				
Policy				
Health Information and Quality Authority. National standards for safer better healthcare.[75]	2012	Describes eight standards: person-centred care and support, effective care and support, safe care and support, better health and wellbeing, leadership, governance and management, workforce, use of resources, and use of information. These were intended to set expectations for healthcare in Ireland and guide improvement of quality, safety, and reliability. They were replaced recently by a principles-based approach.[76]		
Health Information and Quality Authority. National standards for the conduct of reviews of patient safety incidents.[77]	2017	Details standards based around five themes: governance and accountability, person-centred approach to the review of patient safety incidents, workforce, reviews of patient safety incidents, and sharing the learning for improvement. These are intended to ensure a consistent national approach to reviews of patient safety incidents.		

For peer review only - http://bmjopen.bmj.com/site/about/guidelines.xhtml

Health Information and Quality Authority. Standards development framework: a principles-based approach.[76]	2021	Describes a set of principles which are designed to underpin all national standards for health and social care services, to be used as framework during their development. These principles are a human rights-based approach, safety and wellbeing, responsiveness, and accountability; they are all intended to ensure patients receive person-centred care and support.
Health Service Executive. Patient safety strategy 2019-2024.[78]	2019	Details eight patient safety commitments (including specific actions within each commitment, and the rationale and principles behind them): empowering and engaging patients to improve patient safety, empowering and engaging staff to improve patient safety, anticipating and responding to risks to patient safety, reducing common causes of harm, using information to improve patient safety, and leadership and governance to improve patient safety. Its primary purpose is to guide safety improvement at the service level.
Health Service Executive. Healthcare audit – quality assurance and verification. Standards.[79]	2019	Sets out a framework and basic principles – integrity, objectivity, confidentiality, and competency - for the conduct of healthcare audits in Ireland. All HSE healthcare organisations and private or voluntary bodies with a service level agreement with the HSE are required to be audited (in addition to their internal audit functions) by HSE Healthcare auditors, who are members of the Chartered Institute of Internal Auditors. Audits are independent, objective assessments of the delivery of healthcare services. They provide assurance that standards are being met and recommendations to drive improvement.
Health Service Executive. Incident management framework.[80]	2020	A framework that describes a practical approach all healthcare providers can use to identify, report and review incidents. The framework focuses on the system rather than individual. Six steps are described in the process: preventing incidents by supporting a culture where safety is a priority, identifying incidents and immediate actions, initial reporting and notification, categorisation and initial assessment (from category 1, a major incident, to category 3, a minor incident, which determines who needs to be notified and the level of review required), review and analysis, and improvement planning and monitoring.

For peer review only - http://bmjopen.bmj.com/site/about/guidelines.xhtml

Health Service Executive. HSE Enterprise risk management policy and procedures 2023.[81]	2023	A practical resource for healthcare professionals, whether working in a clinical or management capacity, intended to be used in planning and strategy development, decision-making, and day-to-day management. It sets out policy, processes and procedures for the proactive management of risk in healthcare organisations, based on an enterprise approach (i.e. considers all risks, whether management or service delivery-related, together). This involves identifying and predicting uncertainties and threats, describing and analysing the level of the threat, responding where possible (e.g. through control measures), and reviewing and acting (e.g. communication and escalation regarding a threat).		
Research		**Aims**	**Methods**	**Findings**
Health Information and Quality Authority. Overview report of five years of HIQA monitoring in Irish public acute hospitals against national standards: 2015–2019.[82]	2020	To report key findings, examples of good practice and opportunities for improvement identified during monitoring of four national standards - infection prevention and control, medication safety, maternity services, and nutrition and hydration.	On-site inspections in hospitals between 2015-2019: infection prevention and control (n=161), medication safety (n=64), maternity services (n=22), and nutrition and hydration (n=5).	The greatest improvements were seen in relation to infection prevention and control and medication safety. However, there was variation between hospitals, affecting the quality of care provided. Factors such as insufficient resources hindered the implementation of the national standards.
**New Zealand**				
Policy				
Ministry of Health. Pae Ora (Healthy Futures) Act 2022.[85]	2022	The purpose of the Act is to provide for the public funding and provision of services in order to: (a) protect, promote, and improve the health of all New Zealanders; and (b) achieve equity in health outcomes among New Zealand's population groups, including striving to eliminate health disparities, in particular for Māori; and (c) build towards pae ora (healthy futures) for all New Zealanders.		

For peer review only - http://bmjopen.bmj.com/site/about/guidelines.xhtml

Simmonds S, Carter M, Haggie H, Mills V, Lyndon M, Tipene-Leach D. A Cultural Safety Training Plan for Vocational Medicine in Aotearoa. Te ORA and the Council of Medical Colleges.[86]	2023	Presents 'a plan for cultural safety training that medical colleges can employ in the development of their own cultural safety training programmes and in continuing professional development (CPD) with college fellows.'(p. 4) The plan is specific to the New Zealand context and 'focuses on cultural safety through the lens of Māori patients and their whānau.'(p. 4)
Standards New Zealand. NZS 8134:2021 Ngā Paerewa Health and disability services standard.[87]	2022	The standard promotes the safe provision of fair and equitable services for people and their whānau in New Zealand Aotearoa. It sets the foundations for promoting best practices and fostering continuous improvement in the quality (including safety) of health and disability services.
Te Tāhū Hauora Health Quality & Safety Commission. From knowledge to action: A framework for building quality and safety capability in the New Zealand health system.[88]	2016	'This document describes a high-level framework to guide the development of quality and safety capability across all levels in the health and disability sector'.(p. 4) It aims to address '…existing workforce needs, sustainably building the quality improvement capability of the future workforce, developing specialist roles in quality improvement science, supporting consumer/patient participation, ensuring decision-making is based on data and evidence, and supporting boards to provide leadership that encourages a quality improvement and patient safety focus throughout the sector.'(p. 4)
Te Tāhū Hauora Health Quality & Safety Commission. Clinical governance: guidance for health and disability providers.[89]	2017	Sets out a high-level framework for clinical governance in health and disability services. The values that underpin the framework are consumer wellbeing and safety. The guide is intended for use by all health and disability service providers, regardless of their size or complexity, and their staff, as well as other stakeholders.
Te Tāhū Hauora Health Quality & Safety Commission. Clinical governance framework: collaborating for quality (draft, November 2023).[90]	2023	A draft clinical governance framework to replace the clinical governance framework of 2017. Its purpose is '…to provide a comprehensive approach to enable the health sector to develop their own clinical governance infrastructure, adapted to their clinical context.' (p. 6) 'The framework is intended to support a culture of participatory leadership, where collaboration drives quality' and is '… deliberately inclusive of the health workforce, as opposed to being solely focussed on managers and clinicians, as it is applicable across the health sector and needs to be inclusive of all health environments.' The document also states that: 'The framework has been intentionally designed for the Aotearoa New Zealand context, to enact Te Tiriti o Waitangi [the founding document of Aotearoa New

For peer review only - http://bmjopen.bmj.com/site/about/guidelines.xhtml

		Zealand, signed by the Crown and many hapū and iwi leaders] and strive to achieve equity.'(p. 6)
Te Tāhū Hauora Health Quality & Safety Commission. Guide to doing a learning review.[91]	2023	To enable health providers to review harm by applying the learning review method, in which the reviewer aims 'to understand the realities of everyday work to uncover how harm occurs, minimise risk in the system and support health care workers to do the right thing'(p. 3), including identifying areas for improvement. This method is influenced by concepts such as human factors and resilient healthcare, and takes a systems approach to safety.
Te Tāhū Hauora Health Quality & Safety Commission. National adverse events reporting policy.[92]	2023	The purpose of the policy is to provide 'a consistent way to understand and improve through reporting, reviewing and learning from all types of harm.' (p. 5). It is intended to guide reporting to Te Tāhū Hauora [Health Quality and Safety Commission], and the use of information from learning reviews, and quality improvement approaches to strengthen system safety. The policy differs from the previous policy by shifting the focus to 'system safety' and restorative response in the context of partnership with consumers and whānau.
The National Collaborative for Restorative Initiatives in Health. He Maungarongo ki Ngā Iwi: Envisioning a restorative health system in Aotearoa New Zealand.[93]	2023	Provides an overview of the systems that mitigate and respond to healthcare harm in Aotearoa NZ, adopting a human-centred and relational approach. It presents a framework of principles and practices and defines restorative systems as being 'distinguished by an emphasis on relational principles, practices and goals that promote and restore human dignity and wellbeing.'(p. 10)
[a]The original NTA 8009 document was updated twice, in 2011 and 2014, before the most recent NEN 8009 (2018) version.<br>[*]In Dutch, accessed in translation.		

For peer review only - http://bmjopen.bmj.com/site/about/guidelines.xhtml

Supplementary material 5: Search information

Medline via Ovid, 8/12/23:

1 (safety adj3 management adj3 system*1).ti,ab. (588)
2 (safety adj3 management adj3 program*).ti,ab. (93)
3 1 or 2 (670)
4 (patient*1 adj2 (safety or harm* or risk*)).ti,ab. (204300)
5 patient harm/ or patient safety/ (25896)
6 4 or 5 (219200)
7 ((safety or hazard* or risk or risks) adj2 (accountability or assess* or assurance* or commitment* or communicat* or control* or coordinat* or cultur* or education or framework* or guidance or implement* or improvement* or leadership* or management or measurement* or monitor* or plan or plans or planning or policy or policies or promot* or quality or regulat* or reporting or responsibilit* or strateg* or training)).ti,ab. (285563)
8 *Safety Management/ (13937)
9 7 or 8 (295644)
10 system*1.ab. /freq=2 (1032216)
11 system*1.ti. (682356)
12 10 or 11 (1448707)
13 6 and 9 and 12 (3003)
14 3 or 13 (3632)

Embase via Ovid, 9/2/2024

1	(safety adj3 management adj3 system*1).ti,ab.	924
2	(safety adj3 management adj3 program*).ti,ab.	128
3	1 or 2	1038
4	(patient*1 adj2 (safety or harm* or risk*)).ti,ab.	343333
5	patient safety/ or patient harm/ or patient risk/	173057
6	4 or 5	469009
7	((safety or hazard* or risk or risks) adj2 (cultur* or education or implement* or improvement* or management or measurement* or	

For peer review only - http://bmjopen.bmj.com/site/about/guidelines.xhtml

monitor* or quality or regulat* or reporting or responsibilit* or strateg* or training)).ti,ab. 123921
8 System*1.ab. /freq=2 1290950
9 System*1.ti. 770274
10 8 or 9 1735239
11 6 and 7 and 10 3017
12 3 or 11 3986
13 limit 12 to conference abstract status 996
14 12 not 13 2957

CINAHL via EBSCOhost, 9/2/2024

#	Query	Results
S8	S1 OR S7	2,564
S7	S3 AND S4 AND S6	2,373
S6	S2 OR S5	107,816
S5	(MM "Patient Safety")	38,651
S4	TI (system or systems) OR AB (system or systems)	516,337
S3	TI (((safety or hazard* or risk or risks) N1 (cultur* or education or implement* or improvement* or management or measurement* or monitor* or quality or regulat* or reporting or responsibilit* or strateg* or training)).) OR AB (((safety or hazard* or risk or risks) N1 (cultur* or education or implement* or improvement* or management or measurement* or monitor* or quality or regulat* or reporting or responsibilit* or strateg* or training)).)	37,449
S2	TI ((patient* N1 (safety or harm* or risk*))) OR AB ((patient* N1 (safety or harm* or risk*)))	81,650

For peer review only - http://bmjopen.bmj.com/site/about/guidelines.xhtml

S1	TI safety N3 management N3 system* OR AB safety N3 management N3 system*	238

Web of Science: 10/2/2024

- WOS.SCI: 1900 to 2024
- WOS.AHCI: 1975 to 2024
- WOS.ESCI: 2015 to 2024
- WOS.ISTP: 1990 to 2024
- WOS.SSCI: 1900 to 2024
- WOS.ISSHP: 1990 to 2024

Searches:
1: TS=(safety near/1 management) AND TS=((management near/1 (system or systems))) Results:3363
2: TI=((patient* near/2 (safety or harm* or risk*))) Results: 61188
3: TS=(safety near/1 (cultur* or education or implement* or improvement* or management or measurement* or monitor* or quality or regulat* or reporting or responsibilit* or strateg* or training)) Results: 78695
4: TS=(hazard* near/1 (cultur* or education or implement* or improvement* or management or measurement* or monitor* or quality or regulat* or reporting or responsibilit* or strateg* or training)) Results: 8001
5: TS=(risk near/1 (cultur* or education or implement* or improvement* or management or measurement* or monitor* or quality or regulat* or reporting or responsibilit* or strateg* or training)) Results: 122887
6: TS=(risks near/1 (cultur* or education or implement* or improvement* or management or measurement* or monitor* or quality or regulat* or reporting or responsibilit* or strateg* or training)) Results: 122887
7: TS=(system or systems) Results: 11251037
8: #6 OR #5 OR #4 OR #3 Results: 205099
9: #7 AND #8 AND #2 Results: 1288
10: TS=(patient* or health* or hospital*) Results: 11662421
11: #10 AND #1 Results: 1116
12: #9 OR #11 Results: 2379

For peer review only - http://bmjopen.bmj.com/site/about/guidelines.xhtml

Details of website searches of patient safety organisations in included countries

Organisation	URL	Search date		No. of hits	No. of includes
**Australia**					
Australian Commission on Safety and Quality in Health Care	https://www.safetyandquality.gov.au/	01/12/2023	safety management system	141	5
Department of Health and Aged Care	https://www.health.gov.au/	06/12/2023	safety management system	171	0
Australian Institute of Health and Welfare	https://www.achs.org.au/	06/12/2023	safety	63	0
Australian Council on Healthcare Standards	https://www.achs.org.au/	06/12/2023	safety	43	0
**Canada**					
Healthcare Excellence Canada	https://www.healthcareexcellence.ca/en	05/01/2024	safety	406 (200 screened)	4

For peer review only - http://bmjopen.bmj.com/site/about/guidelines.xhtml

Institute for Safe Medication Practices (ISMP) Canada	https://ismpcanada.ca	05/01/2024	safety management system	11	0
**Ireland**					
Health Information and Quality Authority (HIQA)	https://www.hiqa.ie	01/02/2024	Publications - safety	104	4
National Patient Safety Office, Ireland	https://www.gov.ie/en/campaigns/af4b6-national-patient-safety-office/	01/02/2024	safety	44	0
National Quality and Patient Safety Directorate	https://www2.healthservice.hse.ie/organisation/nqpsd/	01/02/2024	n/a	No search function	3
**Netherlands**					
NIVEL (The Netherlands Institute for Health Services Research)	https://www.nivel.nl/en	01/02/2024	safety management	76	2
Dutch Patient Safety Programme	https://www.vmszorg.nl/	05/12/2023		In Dutch	2
Netherlands Health and Youth Care Inspectorate, Ministry of Health Welfare and Sports	https://english.igj.nl	05/12/2023	safety	56	0
**New Zealand**					

For peer review only - http://bmjopen.bmj.com/site/about/guidelines.xhtml

Te Tāhū Hauora Health Quality and Safety Commission	https://www.hqsc.govt.nz/	01/02/2024	Resource library - safety management system	115	9

For peer review only

For peer review only - http://bmjopen.bmj.com/site/about/guidelines.xhtml

BMJ Open

The implementation of Safety Management Systems (SMS) in healthcare: a systematic review and international comparison

Journal:	*BMJ Open*
Manuscript ID	bmjopen-2025-107772.R1
Article Type:	Original research
Date Submitted by the Author:	22-Jan-2026

Complete List of Authors:	de Bell, Siân ; University of Exeter, Exeter HS&DR Evidence Synthesis Centre, Department of Health & Community Sciences<br>Zhelev, Zhivko; University of Exeter, Exeter HS&DR Evidence Synthesis Centre, Department of Health & Community Sciences<br>Bethel, Alison; University of Exeter, Exeter HS&DR Evidence Synthesis Centre, Department of Health & Community Sciences<br>Clarke, Maria; University of Exeter, Exeter HS&DR Evidence Synthesis Centre, Department of Health & Community Sciences<br>Anderson, Rob; University of Exeter, Exeter HS&DR Evidence Synthesis Centre, Department of Health & Community Sciences<br>Thompson Coon, Jo; University of Exeter, Exeter HS&DR Evidence Synthesis Centre, Department of Health & Community Sciences

**Primary Subject Heading**:	Health policy
Secondary Subject Heading:	Health services research
Keywords:	Systematic Review, Safety, Organisation of health services < HEALTH SERVICES ADMINISTRATION & MANAGEMENT

SCHOLARONE™
Manuscripts

I, the Submitting Author has the right to grant and does grant on behalf of all authors of the Work (as defined in the below author licence), an exclusive licence and/or a non-exclusive licence for contributions from authors who are: i) UK Crown employees; ii) where BMJ has agreed a CC-BY licence shall apply, and/or iii) in accordance with the terms applicable for US Federal Government officers or employees acting as part of their official duties; on a worldwide, perpetual, irrevocable, royalty-free basis to BMJ Publishing Group Ltd ("BMJ") its licensees and where the relevant Journal is co-owned by BMJ to the co-owners of the Journal, to publish the Work in this journal and any other BMJ products and to exploit all rights, as set out in our licence.

The Submitting Author accepts and understands that any supply made under these terms is made by BMJ to the Submitting Author unless you are acting as an employee on behalf of your employer or a postgraduate student of an affiliated institution which is paying any applicable article publishing charge ("APC") for Open Access articles. Where the Submitting Author wishes to make the Work available on an Open Access basis (and intends to pay the relevant APC), the terms of reuse of such Open Access shall be governed by a Creative Commons licence – details of these licences and which Creative Commons licence will apply to this Work are set out in our licence referred to above.

Other than as permitted in any relevant BMJ Author's Self Archiving Policies, I confirm this Work has not been accepted for publication elsewhere, is not being considered for publication elsewhere and does not duplicate material already published. I confirm all authors consent to publication of this Work and authorise the granting of this licence.

For peer review only

The implementation of Safety Management Systems (SMS) in healthcare: a systematic review and international comparison

Siân de Bell[1*a], Zhivko Zhelev[1*], Alison Bethel[1], Maria Clarke[1], Rob Anderson[1], Jo Thompson Coon[1]

[1]Exeter HS&DR Evidence Synthesis Centre, Department of Health & Community Sciences, University of Exeter Medical School, University of Exeter, Exeter, UK

*Joint first author

[a] Corresponding author: Sian de Bell s.c.de-bell@exeter.ac.uk

For peer review only

For peer review only - http://bmjopen.bmj.com/site/about/guidelines.xhtml

Abstract

Objectives Many industries where safety is a priority (e.g. aviation) use safety management systems (SMSs) but evidence on their use in healthcare is needed to determine whether they could support patient safety improvement. We investigated the application of national-level SMSs to patient safety in terms of effectiveness, implementation and experience.

Design Systematic review using a case study approach.

Data sources We identified patient safety organisations in each country and searched their websites. We also searched MEDLINE (in December 2023) and Embase (via Ovid), CINAHL (EBSCO) and Web of Science (in February 2024).

Eligibility criteria Any evidence from five high-income countries that have publicly funded healthcare systems with universal coverage: Australia, Canada, Ireland, New Zealand and the Netherlands. We included publications on the effectiveness, implementation or experience of the patient safety approach in each country.

Data extraction and synthesis Included evidence was summarised and mapped onto an initial analytical framework based on analysis of SMSs in high-risk industries, enabling cross-country comparisons. Drafts were shared with experts from patient safety organisations in each country for comment. Formal quality appraisal was not possible as most evidence was non-evaluative.

Results Fifty-three publications were included, from Australia (5), Canada (7), Ireland (8), New Zealand (9), and the Netherlands (24). Only the Netherlands implemented a patient safety programme explicitly based on an SMS approach. Some aspects of patient safety in hospitals improved as a result but there was significant variation in both the implementation of the programme and attributed outcomes.

In the other four countries, the main components of an SMS were identified to some extent, along with evidence that their patient safety approaches had been influenced by concepts from high-risk industries and ‘safety science’ more widely.

Conclusions Emerging ideas from high-risk industries (beyond SMSs) and broader safety science have influenced all countries, reflecting increasing awareness of the need for initiatives to be context-specific to be successful. However, their implementation and impact need further evaluation.

Registration: CRD42023487512

Key words
safety management systems; patient safety; healthcare; systematic review

Strength and limitations

- This systematic review developed a framework defining the key components of a safety management system, from their use to improve safety in high-risk industries (e.g. aviation), and applied this to analyse national patient safety approaches in healthcare.
- We included a broad evidence base, searching the websites of patient safety organisations alongside searching for peer-reviewed articles, and consulting safety experts in included health systems to verify our understanding of their approach to safety management in healthcare.

For peer review only - http://bmjopen.bmj.com/site/about/guidelines.xhtml

- We focused on high-income countries with healthcare systems comparable to the UK. However, we were only able to include those where evidence (e.g. policy documents) was available in English.
- Our analysis was based mainly on non-evaluative documents, so we were not able to conduct quality appraisal or make firm conclusions about the impacts of adopting a safety management system approach in healthcare.

1. Background

All healthcare systems work in various ways to improve patient safety, from creating policies at national and organisational levels to implementing interventions as a result. The challenges of ensuring safe healthcare are recognised by the World Health Organisation (WHO), which has developed the Global Patient Safety Action Plan 2021-30 to provide countries with a framework to improve their national patient safety approaches.[1] However, patient safety improvement is not an easy process; for example, a national review of patient safety in England in 2024 found that, compared to the previous review in 2022, performance had worsened in a number of areas (e.g. maternity care).[2] Globally, patients continue to be harmed by failures or errors, with examples of safety incidents including mistakes associated with the use of medication, such as in prescribing or dispensing, and wrong site surgery.[3] In high income countries, one in ten patients are thought to be subject to an adverse event whilst receiving hospital care each year,[1] whilst in England, it is estimated that 237 million medical errors occur annually, contributing to more than 1,700 deaths.[4]

Safety is also a significant priority in other industries - such as aviation, nuclear energy and oil and gas - where errors could have serious consequences for people or the environment.[3] Many of these 'safety-critical' industries, and companies within them, have improved safety by taking a systems approach – considering the many interacting organisation and process-level factors (e.g. culture, technology, team relationships) that may result in errors or adverse events.[1] This has led to the development of international or national policies and standards for safety, with organisations operationalising these in an organised approach to managing safety known as a safety management system (SMS).[5] There is no single definition for an SMS, but they are generally considered to have four key components:

- Leadership commitment and safety policy – an expressed leadership commitment to safety with documentation of responsibilities and processes for safety within an organisation,[3, 6]
- Safety risk management – the identification of hazards and risk, and assessment of how to mitigate these,[3]
- Safety assurance – monitoring and measuring safety within the organisation and ensuring continuous improvement,[3]
- Safety promotion and culture – training, education and communication of safety to staff at all levels.[7]

The underlying theory behind SMSs is that by focusing on performance - monitoring and achieving desired outcomes - rather than solely on compliance, they facilitate improvement.[3] In 2012, a systematic review of SMSs in three safety-critical industries – aviation, marine and rail – concluded that the approach led to improvements in safety (e.g. reduced accident rates).[8] This suggests that they have the potential to improve safety in other sectors where safety is paramount, such as healthcare. The WHO Global Patient Safety Action Plan 2021-30 recognises "the strong parallels [of healthcare] with the experience of other high-risk industries, thus creating opportunities for transfer learning" (p2).[1]

For peer review only - http://bmjopen.bmj.com/site/about/guidelines.xhtml

The basic components of an SMS (as described above, with further detail given in Table 1) are quite generic and transferrable.[9] It is not possible to provide a more detailed description of a single SMS, as in order to be effective, the specifics need to be adapted to the context in which they are being used, as can be seen from SMSs in other high-risk industries.[10] For example, the aviation, nuclear, and oil and gas industries all have international guidelines regarding safety management.[6, 7, 11] In aviation, these are used by countries to develop state safety programmes with detailed requirements for safety management (i.e. SMSs) by individual organisations. Nuclear safety is considered a national policy issue, with states responsible for the safety of their own nuclear facilities. The oil and gas industry differs from both in that it identifies and manages safety risks within the context of achieving performance goals and stakeholder benefits.[11] In all these industries, individual organisations implement policy and guidance according to their own ways of working.

Whilst healthcare systems globally are working to improve patient safety, countries have different starting points in terms of the organisation and management of their healthcare, and different pre-existing system components (e.g. approaches to monitoring patient safety incidents). Most healthcare systems have some components of an SMS, such as policies for improving patient safety,[12] with some countries - notably the Netherlands[13] - explicitly developing and promoting an SMS approach. Their experiences offer opportunities for learning, and determining whether the principles of SMS are effective in improving safety in healthcare, and if so, how they can be adapted from industries in which they are used, and implemented within a healthcare system. In this review, we focus on the high-level influence or coordination of the implementation of an SMS approach, rather than the operation of organisation-level SMSs.

1.2 Objectives

This systematic review investigated the application of SMSs to patient safety in healthcare, to consider whether and how they could be implemented more widely, asking:

- How are the components of an SMS reflected in the healthcare policy documents of the included countries?
- What research or other relevant evidence is available regarding the effectiveness, implementation or experience of SMSs within healthcare?
- What does evidence from beyond the UK tell us about the effectiveness, implementation or experience of SMSs within healthcare?

This review was commissioned by the National Institute for Health and Care Research (NIHR) Health Service Delivery Research (HSDR) programme on behalf of the NHS England Patient Safety Team and Department of Health & Social Care, to inform the further development of the NHS England's patient safety policy and practice. The UK was not included in the review as: (i) the NHS has not implemented an SMS; and (ii) the intention was to learn from the experiences of other countries, with different starting points in terms of the organisation and management of healthcare, and different pre-existing (safety) system components, regarding their patient safety approaches.

2. Methods

This systematic review was registered on PROSPERO (CRD42023487512) and can be found in Supplementary Material 1. A summary of the methods is provided below; further detail can be found in the full report.[14]

For peer review only - http://bmjopen.bmj.com/site/about/guidelines.xhtml

2.1 Inclusion and exclusion criteria

Evidence from the following countries was eligible for inclusion in the review: Ireland, Australia, New Zealand, Canada and the Netherlands. These countries were selected as they are comparable to the UK, in that they are high-income countries[15] with national healthcare systems, and scoping searches had provided indicative evidence of the adoption of an SMS approach, or some of its key components, to improve patient safety. We also restricted the review to these countries as we had limited ability to translate documents.

As this is an emerging, policy-relevant topic, we included a broad range of types of evidence[16]:

- Policy documents, including those related to the implementation of an SMS or patient safety approach,
- Published or unpublished research / evaluations (quantitative, qualitative or mixed methods),
- Other evidence (e.g. patient and staff experience from organisations' websites).

Evidence relating to any service user population and any healthcare setting were included in the review; study participants could include healthcare professionals, service users, or carers. In terms of focus, we included evidence on any policies, initiatives, or programmes related to the key components of an SMS (as defined above) or patient safety approach. See Supplementary Material 2 for more information.

2.2 Search strategy and study selection

MEDLINE (from 1946) was searched in December 2023 and Embase (from 1974) (via Ovid), CINAHL Ultimate (from 1937) (EBSCO) and Web of Science (WoS): Science Citation Index (from 1990), Social Science Citation Index (from 1900), Arts and Humanities Index (from 1975), Conference Proceedings, Science, Social Science and Humanities (from 1990), Emerging Sources Citation Index (from 2015) in February 2024; (see search strategies in Supplementary Material 3. In March 2024, forwards citation searching was undertaken in Web of Science using eight papers (from the Netherlands, as the only country explicitly using an SMS approach). The reference lists of all publications that met our inclusion criteria were checked for relevant publications.

Key policy documents and evidence that might not be captured through database searches (e.g. programme reports) were identified by searching the websites of, and contacting, national patient safety organisations from each of the included countries (Supplementary Material 3). To understand the context within each country and identify further sources of evidence, we also held online meetings with patient safety/health policy experts from:

- Australia - Australian Commission on Safety and Quality in Health Care; Department of Health and Aged Care (federal government)
- Canada - Healthcare Excellence Canada
- Ireland - Health Information and Quality Authority (HIQA); Royal College of Surgeons Ireland
- Netherlands - Netherlands Institute for Health Services Research (NIVEL)
- New Zealand - Health Quality and Safety Commission

All results from the database searching were independently screened by two reviewers (SDB, ZZ). The full text of papers which were judged to meet the inclusion criteria were obtained, along with policy and other documents identified by web-searching. Two reviewers (SDB, ZZ) assessed each full text independently for inclusion. At both stages, disagreements were settled through discussion.

For peer review only - http://bmjopen.bmj.com/site/about/guidelines.xhtml

In December 2025, we checked that patient safety policy documents from the included countries remained the same, and conducted further forward citation chasing on included studies.

2.3 Data extraction and synthesis

Whilst creating the review protocol and searching for studies, we developed an analytical framework of the components of an SMS by reviewing policy documents and review articles from high-risk industries (focusing on aviation,[7] oil and gas[11] and nuclear energy[6], see Table 1). Data extraction and thematic analysis of evidence from the healthcare systems of each included country was guided by this framework.

Our protocol specified that we would only include countries which explicitly stated that they were using an SMS approach in their healthcare system, with our scoping searches suggesting that Australia, Canada, Ireland, the Netherlands and New Zealand all met this criterion. However, after screening studies for inclusion, we found that the Netherlands was the only country where this commitment to an SMS approach was explicit. Following consultation with our main stakeholders, the NHS Patient Safety Team, we continued to include the other four countries in the review, extracting data on the use of components of an SMS in their healthcare systems, as 'the principles underpinning SMS may help healthcare organisations to respond more effectively to patient safety risks' (p. 8).[3]

A case study approach was taken, with the patient safety approach in each country described and analysed separately, and draft descriptions for each country shared with experts from patient safety organisations in the respective countries for correction or comment. In the final step of the analysis, we compared the experience and implementation of the SMS or patient safety approach across countries. This involved development of the initial framework - the four main components of an SMS remained the same, but subcategories were revised based on the information in the included evidence. Included publications were not quality appraised as the majority were not empirical studies so quality appraisal would not have made a significant contribution to our understanding of the application of SMSs in healthcare. We did consider uncertainties in the evidence, stemming from methodological limitations, when reporting on the Netherlands (as the only country that had explicitly used an SMS approach).

For peer review only - http://bmjopen.bmj.com/site/about/guidelines.xhtml

Table 1 Framework of the components of an SMS

Key component	Subcategory	Definition
Leadership commitment and safety policy	Management commitment and accountability	Individuals in the organisation who are accountable for safety, and their exact role and responsibilities, should be clearly identified.[3] These individuals should ensure that the people they are managing have the capabilities to complete their required activities safely and provide support for them to do this (e.g. resources).[6]
	High-level (policy) documents	The safety policy should outline the aims and objectives of an organisation in terms of safety.[3]
	1. Implementation plan	There should also be a supporting document which details how these will be fulfilled i.e. a framework for the management of safety, including safety standards detailing what needs to be done and by whom, and an improvement strategy.[6]
	2. External and internal boundaries of SMS	External boundaries might include requirements for contractors to have an SMS in place or be considered within the SMS of the organisation. Internally, the relation of the SMS to other management systems (e.g. quality) needs to be defined.
	Coordination of the emergency response plan	Planning for immediate activities in response to an unplanned event e.g. an accident.[7]
Safety risk management	Hazard identification processes	There should be formal processes, of which there are likely to be more than one (e.g. audits, incident reporting), in place to identify hazards.[7]

For peer review only - http://bmjopen.bmj.com/site/about/guidelines.xhtml

	Risk assessment and mitigation processes	This will identify the acceptability of the risk and appropriate measures to control it.[6]
Safety assurance	Safety performance monitoring and measurement	This involves the development of measures, both reactive and proactive, to routinely monitor safety performance within the organisation. It may also involve periodic audits and reviews of performance (including compliance), whether internally or externally (e.g. by regulators).
	Internal safety investigations	Systems are needed to allow the reporting of safety incidents, and processes to investigate their causes.
	Continuous improvement	Organisations should have arrangements to ensure that actions are taken if issues are identified as a result of monitoring and investigations, including ensuring that these are completed within appropriate timescales.
Safety promotion and culture	Training and education	Training may be specific to an individual's role, or to gain a wider understanding of safety issues, but is essential to ensure competence.[6] It should be identified, documented, and reviewed and revised regularly.
	Safety communication	This may be formal (e.g. safety meetings), or informal (e.g. mention of issues in team briefings), but needs to be open and allow individuals to understand and accept why standards of safety are required, and provide feedback.[6]
	Presence of safety culture	Also referred to as a just culture, allowing open communication and reporting of incidents or hazards.

For peer review only

2.4 Public and patient involvement and engagement

Due to the focus of review, we sought public collaborators who had some knowledge of the topic. We held meetings with three public collaborators, two of whom had knowledge of safety management in other industries (chemical and police/counter-terrorism) and one who had experienced a safety incident in the NHS and has been campaigning to raise awareness of patient safety. In these meetings, we discussed their experiences of safety in other industries (where relevant) and of patient safety in the NHS, as well as their views on the improvement of patient safety. Our public collaborators provided important contextual information on safety management and SMS in other industries (e.g. the importance of clear accountability for safety) and their potential use in the NHS. They also informed specific aspects of the review (e.g. discussions of their own safety experiences in the NHS highlighted areas to consider in our data extraction and analysis, such as approaches to involving patients in safety policy and incident investigations and applying learning from safety incidents).

3. Results

Database searches resulted in 11,532 hits; 6,913 records were screened at title and abstract level. Of those, 158 were selected for full text screening and 89 were found to meet the inclusion criteria. Searches of websites resulted in 32 publications being included in the review, along with 22 documents from other sources (14 from the reference lists of included documents and eight from expert suggestions). This resulted in a total of 143 studies being included in the review. Further details can be found in Figure 1 and Supplementary Material 4.

Figure 1 PRISMA diagram of the screening process [figure uploaded separately]

Due to the quantity of evidence found, we grouped publications into two broad categories (Table 2):

- Descriptions of each country's approach to patient safety, including influences from high-risk industries (mainly policy documents but also outputs e.g. opinion papers).
- Evidence on the impact and implementation of a specific patient safety approach, or aspects of it (mainly research papers and project reports). These were categorised further as:
    - 'rich' – reporting on the impact or implementation of the approach, or an essential part of it, and
    - 'thin' – papers with a very narrow focus and limited relevance to our review question (e.g. the accuracy or acceptability of a risk assessment tool implemented as part of a patient safety programme).

Our analysis concentrates on the rich evidence due to our focus on the implementation of patient safety approaches nationally and the need, therefore, for detailed, explanatory accounts of implementation at this level. Thin studies were included to illustrate the way in which a programme was designed or implemented, and not because of their specific results, as they did not provide the necessary conceptual richness, tending to focus on settings and outcomes that were not generalisable (e.g. specific organisations, small-scale interventions, or outcomes less relevant to our research questions such as the accuracy of a tool).

Table 2 Number of evidence sources per category per country.

Country	'Rich' documents (n=53)	

For peer review only - http://bmjopen.bmj.com/site/about/guidelines.xhtml

	Policy	Research	Other*	'Thin' documents (n=90)
Netherlands	2	20	2	22
Australia	3	2	0	37[a]
Canada	5	2	0	27[a]
Ireland	7	1	0	2
New Zealand	9	0	0	6[a]

*Such as opinion papers.

[a]Some included evidence had information from multiple countries (Australia and Canada (n=2) and Australia and New Zealand (n=2)) so has been included in multiple categories.

Our analysis was based primarily on the descriptive and rich evidence. We focused on 53 publications, which can be found in Supplementary Material 5, from Australia (5), Canada (7), Ireland (8), New Zealand (9), and the Netherlands (24) (Table 1). Some 'thin' evidence is used to illustrate specific points (see full list in Supplementary Material 4). Below, we describe the patient safety approach in each country then compare these approaches.

3.1 Netherlands

The Netherlands was the only country to explicitly use an SMS approach, in the form of its national patient safety programme (PSP), which was launched in 2008 and ran until the end of 2012.[17] The programme was implemented in public hospitals and based on the premise that change in the structure or processes of an organisation is necessary to see a change in outcomes.[18] It had two pillars: (i) hospitals were required to implement a certified SMS, and (ii) a set of guidelines focusing on improving patient safety for ten priority themes.[13] The aim was a 50% reduction in adverse events and potentially preventable deaths over a period of 5 years.[13] The two pillars of the PSP continued to be implemented after 2012, with development and revisions based on evaluations of its success. A new safety programme, based on a Safety-II perspective (see Glossary), began in 2020.[19]

The basic SMS requirements were agreed by key stakeholders and published in 2007 (NTA 8009).[20] The complexity of implementing an SMS was acknowledged, with healthcare providers required to carry this out in stages. In Phase 1 the basic requirements of an SMS were developed (leadership, management, personnel, patient participation, prospective risk assessment, retrospective risk assessment and improvements), with further requirements added in Phase 2 (communication, third-party management, and control measures).[20, 21] The latest version of the document specifying these requirements, NEN 8009, was published in 2019; it introduces a new perspective on patient safety based on Safety-II and resilience engineering (see Glossary). These concepts emphasise the importance of learning from successful, 'incident-free' practice as well as from the investigation and prevention of incidents.[21]

For peer review only - http://bmjopen.bmj.com/site/about/guidelines.xhtml

There were ten high-priority themes for patient safety, such as patient mix-up (see Glossary). Expert groups were convened for each theme, and guides were developed describing structure, processes, and outcomes. Interventions to improve patient safety were explained during national theme conferences. The programme also had a website where resources and patient-facing materials were available. Whilst the initial focus was on adult care, six of the patient safety themes were later expanded to include paediatric hospital care.

3.2 Australia

In Australia, patient safety is primarily a national (i.e. federal government) responsibility.[22] However, state and territory governments are responsible for their own public hospital systems, and the licencing and regulation of the private hospital sector, as recognised in the National Health Reform Act 2011 (NHRA - which specifies responsibilities for funding, operating, managing and regulating the health system).[22] The systems and processes used to ensure safety therefore differ between territories and states.[22]

The Australian Commission for Safety and Quality in Health Care developed the National Safety and Quality Health Service (NSQHS) Standards with the aim of ensuring the relevant systems are in place within a healthcare organisation to ensure quality of care.[23] The first edition was published in 2012, the latest in 2021.[23] The NSQHS Standards detail requirements for healthcare organisations regarding clinical governance and partnering with consumers, as well as specific areas of clinical management. They require providers to have an organisation-wide clinical incident management and investigation system, with an open disclosure policy consistent with the Australian Open Disclosure Framework.[22] Certain indicators which should be measured, such as sentinel events, have been developed by the Commission, and the Australian Health Performance Framework details a common set of metrics for public reporting of safety and quality data.

Australia has not used an SMS approach in its healthcare system, although "*the healthcare sector began to draw on lessons from other high risk industries*" such as aviation and mining, as well as using human factors analysis, to develop its patient safety approach.[22] These concepts from high-risk industries have influenced specific elements of Australia's approach (e.g. surgical checklists).

3.3 Canada

The responsibility for delivering health services lies with the provincial and territorial governments, leading to variation in how patient safety is approached in Canada. The Canadian Quality and Patient Safety (CPQS) Framework was launched in 2020 and provides guidance to drive patient safety and quality improvement in all healthcare sectors and settings. Whilst it is intended to align Canadian legislation, regulations, standards, organizational policies, and public engagement nationally,[24] there is currently variation between governments. Not all jurisdictions have quality and safety plans or policies regarding key elements of patient safety.[25]

Healthcare Excellence Canada, an independent non-profit organisation funded primarily by Health Canada - a department of the federal government – has produced a discussion guide that details the recent change in Canada's approach to patient safety. There has been a move from 'traditional' patient safety improvement (e.g. scorecards and audits), to a focus on creating a proactive and inclusive environment, with a culture and processes that support continuous safety improvement.[26] Canada has drawn on learning from high-risk industries, with the Measurement and Monitoring of Safety Framework (MMSF) underpinning its new approach to patient safety but has not taken an SMS approach.[27]

For peer review only - http://bmjopen.bmj.com/site/about/guidelines.xhtml

3.4 Ireland

The Health Information and Quality Authority developed the National Standards for Safer Better Healthcare in 2012; these detail expectations for healthcare, and guide improvement of quality, safety, and reliability.[28] They were replaced by a principles-based approach in 2021.[29] However, the Health Service Executive has developed a national patient safety strategy which builds on the Standards, to be implemented and guide improvement at a local level.[30]

Irish healthcare providers are required to have an incident reporting system.[31] Incidents are entered into the national system and reviewed externally or internally depending on the severity of the incident.[31, 32] Providers are also expected to undertake clinical and non-clinical audits (which may be part of national or local programmes), implement improvements based on these,[30, 33] and assess and manage risks, with a formal process in place to do this.[34] Sláintecare, an improvement plan and strategy intended to reform and develop health services in Ireland over the next decade and into the future, is currently being implemented in Ireland; the provision of healthcare, including the improvement of safety, is consequently evolving.[30]

Whilst Ireland has not taken an SMS approach, the influence of high-risk industries can be seen in various elements of their approach. The national patient safety strategy details an action regarding information and training for staff drawing on "*patient safety and reliability science, systems thinking, audit, quality improvement methodologies, change management, human factors and multidisciplinary team working for safety*",(p.13)[30] with an example of this being the delivery of simulation-based education and human factors training to improve patient safety (see Glossary).[35, 36]

3.5 New Zealand

Patient safety in New Zealand is a central focus of the healthcare system, with the Te Tāhū Hauora Health Quality & Safety Commission (Te Tāhū Hauora), established in 2010, playing a pivotal role in improving healthcare quality and safety across the country.[37] The Pae Ora (Healthy Futures) Act 2022 replaced the country's 20 District Health Boards, aiming to create a more centralised, effective and efficient healthcare system.[37] The Act[37] and the updated Nga Paerewa Standard Health and disability services standard[38] are associated with important changes in the New Zealand's patient safety policy, such as the 'Healing, Learning, and Improving from Harm: National Adverse Events Policy'[39] which took effect from 1st July 2023 and the new 'Clinical Governance Framework: Collaborating for quality' (which is still in a process of consultation).[40]

The new framework has been intentionally designed for the cultural context of New Zealand, with service users and their families seen as 'partners in care'.[40] Elements of safety are emphasised, such as the importance of having processes and systems in place to ensure quality and safety (e.g. clinical risk management systems to deal with non-compliance with clinical standards and policies), and of focusing on 'system safety' – managing risk within a systems perspective – and learning how the healthcare system shapes the experiences of consumers and staff.[40] The MMSF is suggested as a tool that could help decision makers to assess the overall safety of their organisation. To assess the quality of the health and disability system, Te Tāhū Hauora has adopted a range of baseline measures and indicators:

- Atlas of Healthcare Variation
- Quality and Safety Markers (QSMs)
- Quality Accounts

For peer review only - http://bmjopen.bmj.com/site/about/guidelines.xhtml

- Health Quality and Safety Indicators (HQSIs)
- Prevention quality indicators.

The current (2023) version of the clinical governance framework (in consultation) includes a number of ideas (e.g. systems safety, resilient organisations) which are linked to developments in high-risk industries.[40, 41] For example, the 'learning review' method was originally developed for the investigation of firefighter incidents in the context of the US Forest Service and draws on a number of concepts, such as human factors and ergonomics, systems safety and resilient organisations. It was specifically developed for 'adaptive complex systems' – complex systems with high level of uncertainty that interact, learn and adapt over time – such as healthcare.[41]

3.6 Comparison

Although the Netherlands was the only country to explicitly use an SMS approach, the main components of an SMS were identified, to varying extents, in the patient safety policies and initiatives of the other four countries, as described in Tables 3-5. Whilst the interdependence of these elements was acknowledged, they were often detailed in different policies and within the scope of different organisations. Key features that were seen in all five countries, organised according to the components of an SMS, included:

Leadership commitment and safety policy

- Leadership commitment to patient safety.
- National patient safety policy (separately or in some cases an aspect of wider quality improvement policy).

Safety risk management

- Prospective risk management, with risks identified using multiple sources of evidence (e.g. local, national, and international research). Countries had specific standards for high-priority patient safety topics (e.g. patient identity mix-up) as well as general guidelines.

Safety assurance

- Retrospective incident reporting and analysis linked to system for feedback and learning to ensure continuous improvement. Methods for categorising events (e.g. actual harm vs near misses) and their severity (high- and low-) were similar across countries and determined whether and how they were investigated (e.g. central vs local, such as in the Netherlands and New Zealand). Investigation differed (e.g. learning review and restorative practice in New Zealand, root cause analysis in the Netherlands and Australia, see Glossary).
- Monitoring of patient safety performance. Whilst systems for monitoring at the local level were less clear, all countries had a national system.

Safety promotion and culture

- Focus on developing a safety culture (including open communication and blame-free reporting).
- Patients' and families' involvement, both in the development of patient safety policies and safety at the local level.

For peer review only - http://bmjopen.bmj.com/site/about/guidelines.xhtml

Table 3 Comparison of the scope of the SMS or safety approach in each country

	Netherlands	Australia	Canada	Ireland	New Zealand
**Geographical**	National coverage.	National standards; state and territory governments are responsible for their own healthcare systems.	National framework; provincial and territorial governments are responsible for their own healthcare systems.	Standards and patient safety strategy are set at the national level, but implementation is local to allow for adaptation to context.	Transition from regional to national coverage.
**Organisational**	Hospital care.	All healthcare settings.	All healthcare settings.	Health and social care.	All healthcare settings; health and disability care.
**Overlap with quality management/systems**	SMS separate from the quality management and occupational health and safety systems.	Quality and safety are considered together.	Quality and safety are considered together.	Safety is considered part of other systems in the organisation (e.g. quality improvement).	Quality and safety are considered together.
**Extent of accountability for 'other' organisations**	Third party management (outsiders performing activities in hospital) included.	-	-	There should be formalised agreements in place for the provision of services provided externally, including quality and safety.	-

Table 4 Comparison of key features of the SMS or safety approach in each country relating to leadership commitment and safety policy and safety risk management

	Netherlands	Australia	Canada	Ireland	New Zealand
**Leadership commitment and safety policy**	BoD formulates multi-year safety policy and safety plan using SMART-formulated objectives; provides resources (funds, time, expertise, training etc); reviews and evaluates impact and promotes safety culture;	The Standards state that the governing body should provide leadership, set the strategic direction (including a framework with processes to drive improvement), and monitor and review safety and quality.	*"Board members are accountable for safety and for ensuring patient safety concerns and incidents are acted upon"*(p.42);[24] this includes quality and safety goals.	The national standards set out the need for "*A commitment to quality and safety articulated and demonstrated by those governing and leading the service*".(p.72)[28] There should be an identified	Leadership is one of the system drivers in the draft Clinical Governance framework. It enables the health workforce to be active members of the learning organisation by creating a culture of psychological safety and

For peer review only

	management translates the above into their own area of responsibility and reports to BoD. All employees have responsibility and are encouraged to participate in improving safety. Patient safety is included in the hospital's performance system	The governing body of the organisation should also ensure "*that roles and responsibilities are clearly defined for the governing body, management, clinicians and the workforce*"(p.6)[23] in relation to quality and safety, and that clinicians understand their role in patient safety.	Health leaders should encourage and support health teams to improve patient safety, whilst health workers should take an active role in creating a safe environment.	individual with accountability and responsibility for safety, as well as a commitment to safety from leaders at all levels.<br><br>The national patient safety strategy will "*put in place integrated governance structures with clear accountability for planning, managing and addressing the above patient safety priorities.*"(p.17)[30] i.e. boards, management, staff and relevant quality and safety committees within an organisation should all have defined responsibilities for safety.	trust. Organisations need to share leadership by valuing knowledge and expertise drawn from lived experience of consumer and whānau equally alongside clinical and other knowledge. The National Adverse Events Policy states that health and disability service providers are accountable for ensuring the systems they implement recognise various degrees and types of harm; meet the national expectations for reporting, healing, learning and improving; involve consumers and whanau; and create local process to operationalise the policy.
**Safety risk management[a]**	Prospective risk assessment carried out by multi-disciplinary teams using accepted methods (e.g. failure modes, effects, and criticality analysis, FMECA), provided with sufficient time and resources; regularly reviewed.<br><br>Operational control measures: patient safety is integrated in daily business operations; management of change is an aspect of this.	Providers should identify and documents risks (including external and internal disasters and emergencies); take actions to reduce them; regularly review actions, and report on identified risks to staff and patients.	-	The national patient safety strategy details the need to "*proactively identify areas of risk to patient safety*"(p.15),[30] as do the Standards. Learning should also be taken from where things have gone wrong.<br><br>Governance for risk should be integrated into an organisation's management processes.	In the draft Clinical Governance framework, the focus is on system safety and 'learning together' culture. It suggests using the MMSF and actively seeking organisational data that challenges the sense that 'all is well'. A broad range of data sources are listed including quality monitoring, 'soft intelligence', operational data, health workforce and consumer and whanau experience.

For peer review only - http://bmjopen.bmj.com/site/about/guidelines.xhtml

	Focus on ten priority themes.	The NSQHS Standards (6 of which relate to high-risk areas of patient care) and clinical care standards.		Clinical standards.	Clinical standards.
[a] Including risk related to planned changes					

Table 5 Comparison of key features of the SMS or safety approach in each country relating to safety assurance (including subcategory on incident reporting and investigation) and safety promotion and culture

	**Netherlands**	**Australia**	**Canada**	**Ireland**	**New Zealand**
**Safety assurance[b]**					
Safety performance monitoring and measurement	Management is responsible for monitoring patient safety performance in the hospital and reporting of results (e.g. to BoD) at least annually.	Organisations should have quality improvement systems that identify, monitor and report on safety performance and outcomes	*"Ensure that avoidable death and hospital standardized mortality ratio (HSMR) data is tracked and reported so action can be taken to improve outcomes"*(p.34).[24]	Monitoring against National Standards for Safer Better Healthcare by HIQA, using national indicators where possible.	Monitoring against Te Tāhū Hauora's health quality and safety indicators. The Quality and Safety Measures are a mix of process and outcome measures, and focus on key safety priorities: falls, healthcare associated infections, surgical harms and medication safety.
Continuous improvement	Using a multi-disciplinary approach, improvement measures are implemented and their impact assessed.	Organisations should identify areas for improvement and Implement and monitor improvement strategies.	*"Support an audit and feedback system to ensure that recommendations to improve patient safety are implemented"*(p.33).[24]	Requirement for annual audits, national and local. Learning from both incident reporting and monitoring should be used to improve quality and safety.	Continuous learning from adverse events is based on open reporting, investigation and meaningful analysis. Changes are implemented based on the learning process.
Incident reporting and investigation					
Reporting system	Requires safe, efficient and effective reporting processes and a system using accepted methods and pre-specified incident categories (reporting limited to hospital and to BoD). The information is used to improve safety by	NSQHS Standards require an organisation-wide clinical incident management and investigation system.	Health leaders should enable health teams to "*Report, learn from, and act on patient safety concerns and incidents*" (p.24)[24] by ensuring policies and reporting mechanisms are in place.	The Standards detail the need for "*Arrangements to identify, manage, respond to and report patient-safety incidents in a timely manner*".(p.66)[28]<br><br>Incidents need to be entered on a national system, whether they are	The National Adverse Events policy 2023 requires serious adverse events to be reported to and investigated by the Te Tāhū Hauora. Less serious events are reported and managed locally.

For peer review only - http://bmjopen.bmj.com/site/about/guidelines.xhtml

	determining tasks, powers and responsibilities.		Organisational incident reporting and reporting to national systems for certain incidents.	investigated internally or externally depends on severity of incident.	
Method of investigation	Root cause analysis was recommended as an Investigation method initially.[20] NEN8009 (2018) does not recommend a method.[21]	Investigation method is root cause analysis.	-	Investigation method based on systems analysis.[31]	Investigation method is learning review.
Post-incident support	Staff involved in an incident are offered aftercare.	-	Health leaders and boards should ensure that health teams have access to psychological support.	Support (including psychological) for staff involved in a serious incident.	A theme in The National Adverse events policy 2023 'healing': providing emotional and practical support to affected individuals, families and healthcare workers.
**Safety promotion and culture[c]**					
Presence of safety culture	BoD responsible for promoting safety culture, including safe reporting.<br><br>Open communication in all directions.	Organisations should provide '*timely reports on safety and quality systems and performance*'(p.7)[23] to employees at all levels of the organisation, the public, and other healthcare organisations.	Recent move to create a proactive environment, with a culture and processes that support continuous safety improvement (e.g. safety huddles and other methods of open communication).	Systems for staff communication and engagement within organisations as well as with external agencies (e.g. HIQA) and support for programmes to promote a patient safety culture.	Cultural safety; integration of Maori worldview and safety science.
Training and education	BoD and management are responsible for providing training and monitoring whether staff need further training.	The organisation should provide access to training and monitor participation.	Health teams have a responsibility to undertake training and leaders and boards to provide training.	The Strategy identifies the importance *"designing and delivering safety information and training"* (p.13)[30] for staff.	Staff should be provided with learning opportunities.
Patient participation	Patient participation in drawing up and implementing safety policy	Patient participation in "*healthcare governance planning, design, measurement and evaluation*"(p.19)[23]	Patient participation in patient safety (e.g. through incident reporting and as public representatives in policy discussions).	Organisations should "*develop mechanisms to empower patients to contribute*"(p.11),[30] including to governance	*"Organisational clinical priorities, processes and evaluations are co-designed and developed*

For peer review only - http://bmjopen.bmj.com/site/about/guidelines.xhtml

	and reporting incidents, complaints and claims.			structures and reporting incidents.	*collectively"*(p.11)[42] with patients.

[b] Including accreditation, monitoring and reporting of outcomes as well as systematic implementation of improvement measures and evaluation of their impact, [c] Including patient and family involvement. Abbreviations: BoD – Board of Directors

For peer review only

For peer review only - http://bmjopen.bmj.com/site/about/guidelines.xhtml

There were, however, differences in the implementation of these common features between countries (e.g. incident investigation as described above and see Table 5). There were also other areas of variation, particularly relating to the scope of the patient safety approach (Table 3). In most cases, patient safety was considered alongside healthcare quality improvement, although in the Netherlands it was separate. Implementation of policies varied from national to regional, and their application to all or specific healthcare settings (e.g. hospital care only in the Netherlands, or all care settings in the other four countries). Additionally, a range of concepts from high-risk industries and safety science (see Glossary) more broadly (e.g. Safety II, human factors, resilience engineering; see Glossary) had influenced the patient safety approaches of different countries.

All countries considered inequalities to some extent by targeting groups particularly at risk of unsafe care (e.g. older people). The most specific guidance was found in Australia, Canada and New Zealand. In these countries groups were identified in terms of social (e.g. those living in deprivation), ethnic (e.g. Māori and Pacific peoples in New Zealand), and protected characteristics (e.g. sexuality), and actions or objectives were specified to achieve outcomes for these groups. Ireland highlighted the need to consider a person's circumstances and background in their healthcare; the PSP in the Netherlands did not provide guidance for different patient groups beyond the need to pay special attention to older people (e.g. within the ten patient safety themes).

It is difficult to directly attribute changes in patient outcomes to specific policy and management changes, especially when different components are introduced incrementally, and often over many years; only three countries have conducted national evaluations of their approach. In the Netherlands, a longitudinal study comparing data from 2008[43] and 2011/12[44] reported a 45% decrease in potentially preventable adverse events over the course of the national safety programme. However, a more moderate 30% decrease in potentially preventable adverse events, which was not statistically significant, was found after adjusting for data clustering and changes in the patient mix between measurements.[45] There was also variation in the successful implementation of the ten themes between hospitals and departments, with a number of staff-related, organisational and topic-specific factors identified as potential modifiers.[18] Australia and Ireland both found improvements for certain aspects of patient safety,[22, 46, 47] with some of these in areas in which the Netherlands also saw improvement (e.g. medication safety).[13] Similarly, there were factors which all of these countries identified as facilitating better patient safety such as strong governance or leadership.[13, 22, 46, 47]

4. Discussion

Of the five included countries, only the Netherlands has formally adopted an SMS approach.[13] All SMS components were found in the policies of the other four countries, but not in the form of a single unified system. Across all five countries, there was a change in the patient safety discourse away from a narrow focus on reporting and learning from incidents. Whilst reporting was still considered important, the new approaches to patient safety adopted broader definitions of safety (e.g. including psychological and cultural safety) and harm (e.g. including harm resulting from social inequalities and structural oppression), and emphasised taking a systems perspective to safety, learning from successful practice as well as failures. They highlighted the need for wider involvement in the processes of assessing and creating safety, including patients and families as well as staff.

Healthcare is often thought to have more complex and unpredictable demands compared to other industries which have adopted an SMS approach, leading to debate over the suitability of SMSs in contexts and organisations in which service delivery is not stable or predictable.[5]

For peer review only - http://bmjopen.bmj.com/site/about/guidelines.xhtml

The Dutch patient safety programme had some positive impact on patient safety, suggesting an SMS approach is suitable for use in healthcare.[13] However, the evaluation focused on the safety programme as a whole, rather than the SMS specifically, making it difficult to determine whether there are specific components of an SMS that are particularly effective in improving safety. Whilst improvement was seen as part of the programme, this does not mean it is the only way to improve patient safety.[5] The elements of an SMS were present, to some extent, in the healthcare systems of all included countries, but other concepts used by high-risk industries, and from safety science more widely, had influenced the patient safety approach in all countries, with evidence of effectiveness in some cases (e.g. Ireland[46]).

The use of these concepts potentially reflects a new, emerging perspective in which healthcare and high-risk industries are seen as societal and corporate domains that share the same theoretical field – safety science – and can learn from each other, as recognised in documents such as the WHO Global Patient Safety Action Plan 2021-30.[1] This is very different from the idea that, in terms of safety, healthcare is lagging behind and should learn and even copy the high-risk industries' approach to process safety, which has dominated the patient safety discourse.[5, 48] There were considerable differences in the implementation of SMS elements and safety concepts between countries, with patient safety systems reflecting different cultural and organisational realities and local preferences. What works, or makes sense, in one context may not work, or make sense, in another. Comparing and understanding such differences could advance the field of safety science as applied to healthcare and help countries review and improve their own policies and practice. For example, a national review of patient safety in England in 2024 found that, whilst individual healthcare organisations are prioritising common patient safety problems, there is a need for a more focused and coherent approach at the national level.[2] The experiences of a smaller country, such as the Netherlands, which was able to implement a single, highly structured patient safety programme, may be more applicable to the UK context than those of larger, federal countries such as Australia and Canada.

Within this general shift in thinking, different countries had preference for specific concepts and theories from safety science, the most prominent of which were the Measurement and Monitoring of Safety Framework (MMSF) and resilience engineering theory (see Glossary). Regardless of the theoretical underpinnings of a particular patient safety approach, it still needs to be both operationalised and adapted to a particular context. Whilst we focused in this review on how a national organisation can influence or coordinate the implementation of an SMS or safety approach, some policies and most interventions are implemented at the level of service commissioning and/or within health service delivery organisations. For example, the MMSF is a framework and a set of "guiding principles for safety measurement and monitoring"(p.70).[5] It does not provide details on implementation, which remains a challenge, as can be seen in the experience of Canadian healthcare providers of applying the MMSF in their healthcare system.[27] In terms of adaptation, current patient safety discourse recognises that both 'Safety I' (learning from incidents) and 'Safety II' (learning from success) are important and should be part of a modern patient safety approach. Safety II has important implications for the organisation of patient safety, particularly the use of a broader range of safety metrics including outcome measures and resilience indicators as well as adverse events.[49]

However, chosen measures and indicators need to be suitable for the context in which they are being used. As monitoring and evaluation are integral to a systems approach to safety, this is a key area for further research. Realist research, to understand the causal mechanisms through which patient safety initiatives work, and contextual factors that influence their successful implementation, could aid the choice of suitable methods of

For peer review only - http://bmjopen.bmj.com/site/about/guidelines.xhtml

evaluation. Understanding the mechanisms behind different components of patient safety initiatives would also support their adaptation to different contexts. For example, the dominant focus of patient safety approaches in all countries was on hospital care (although all countries apart from the Netherlands included all healthcare settings in their patient safety approaches), but there may be different challenges in other settings such as primary and community care, and for specific patient groups (e.g. patients with learning disabilities), requiring the tailoring of standards.

4.1 Limitations

Although we followed best practice for conducting a systematic review, some limitations should be acknowledged:

- The review is focused on patient safety in selected high-income, English-speaking countries with healthcare systems broadly comparable to that in the UK. Although we conducted scoping searches to decide on our countries of focus, this means that there may be different contexts (e.g. lower income countries) in which SMS are being implemented within the healthcare system.
- Our interpretation is based on analysis of non-evaluative and administrative documents, many of which express aspirations regarding patient safety, rather than being empirically grounded evidence detailing outcomes. We tried to mitigate this limitation by talking to experts from each country and sharing our draft descriptions with them for comment.

5. Conclusions

Only the Dutch patient safety programme was explicitly based on an SMS approach and showed some longitudinal evidence of improving patient safety. Process safety as conceptualised in high-risk industries had some influence on approaches in the other countries, but this was less systematic and explicit. Approaches to patient safety in all countries also draw on a range of concepts from broader safety science. This reflects a shift from the view that healthcare needs to adopt high-risk industries' approach to safety, and increasing awareness that for initiatives to be successful, they need to be adapted to their context, especially through more collaborative learning between industries and sectors.

Glossary

Human factors is the "*study of the interrelationships between humans, the tools they use, and the environment in which they live and work*"(p.210).[48]

Learning review is a specific approach for investigating incidents where the focus is on collaboration and learning, and the system rather than on the individuals involved.[50]

Patient mix-up Errors regarding a patient's identity or their procedure (e.g. surgery involving the wrong side, wrong site, wrong procedure, or wrong patient).[13]

Patient safety "a framework for organised activities that creates cultures, process, procedures, behaviours, technologies and environments in healthcare that consistently and sustainability lower risk, reduce the occurrence of avoidable harm, make errors less likely and reduce impact of harm when it does occur".[1]

For peer review only - http://bmjopen.bmj.com/site/about/guidelines.xhtml

Resilience engineering theory "*looks for ways to enhance the ability of systems to succeed under varying conditions*" i.e. proactively building resilient systems.[5, 51]

Resilient systems/organisations can adjust their functioning before, during, or after events, sustaining required operations under both expected and unexpected conditions.[51]

Restorative practice in the context of the New Zealand patient safety policy *"…is a voluntary, relational process where skilled facilitators support all those affected by an adverse event in a safe and supportive environment. Participants can speak openly about what happened, to understand the human impacts and to clarify responsibility for the actions required for healing and learning.*"[52]

Root cause analysis is a process used to identify the root – or most fundamental -cause of a problem or incident (rather than the first viable cause).[6, 7]

Safety I is an approach to safety in which the focus is on preventing errors and incidents occurring by identifying the causes and responding either when an event occurs or when a risk is deemed unacceptable.[49]

Safety-II is an approach to safety which shifts the focus from what goes wrong to ensuring that things go right (learning from success).[5, 49]

Safety science is considered to be any research, education etc. concerning the science and technology of human safety.[53]

SMART stands for Specific, Measurable, Acceptable, Realistic and Time-bound, in relation to setting objectives.

System safety acknowledges that multiple factors, and the interactions between them, lead to safety, and that the system can be modified to improve safety and resilience.[5]

Systems approach considers multiple levels, i.e. "the interaction between individuals, organisations and sociotechnical system systems" (p.25).[1]

Wrong site surgery A surgical procedure carried out on the wrong side of the body, or on the wrong site on the body.[13]

Acknowledgements

The Exeter HSDR Evidence Synthesis Centre team would like to acknowledge and thank Tracey Herlihey, Matt Fogarty and Jason Cox for their support in developing this review, the experts who took the time to provide us with insight on patient safety in their countries, and our public collaborators for their valuable contributions to our understanding of the topic. We would also like to thank Sue Whiffin and Jenny Lowe for administrative support throughout this review.

For peer review only - http://bmjopen.bmj.com/site/about/guidelines.xhtml

Funding

This report presents independent research funded by the National Institute for Health Research (NIHR). This review (award NIHR136105) was commissioned by the Health Services and Delivery Research (HS&DR) programmes as part of a series of evidence syntheses under award NIHR130538. For more information, visit https://fundingawards.nihr.ac.uk/award/NIHR135660 and https://fundingawards.nihr.ac.uk/award/NIHR130538.

JTC and AB are also supported by the National Institute for Health Applied Research Collaboration South West Peninsula.

Author contributions

Role	Name
Conceptualisation	Sian de Bell (Equal), Zhivko Zhelev (Equal), Alison Bethel (Equal), Jo Thompson Coon (Equal), Rob Anderson (Equal)
Data Curation	Not applicable
Formal Analysis	Sian de Bell (Equal), Zhivko Zhelev (Equal)
Funding Acquisition	Jo Thompson Coon (Equal), Rob Anderson (Equal)
Investigation	Sian de Bell (Equal), Zhivko Zhelev (Equal), Alison Bethel (Supporting), Maria Clarke (Supporting)
Methodology	Not applicable
Project Administration	Jo Thompson Coon (Equal), Rob Anderson (Equal)
Resources	Not applicable
Software	Not applicable
Supervision	Not applicable
Validation	Not applicable
Visualisation	Zhivko Zhelev (Lead)
Writing – Original Draft	Sian de Bell (lead), Zhivko Zhelev (Supporting)
Writing – Editing and Reviewing	Sian de Bell (Equal), Zhivko Zhelev (Equal), Alison Bethel (Equal), Maria Clarke (Equal), Jo Thompson Coon (Equal), Rob Anderson (Equal)

Competing interests

Siân de Bell - Disclosure of interest: none declared

Zhivko Zhelev - Disclosure of interest: none declared

Alison Bethel - Disclosure of interest: none declared

Maria Clarke - Disclosure of interest: none declared

Jo Thompson Coon

- Member of the NIHR HTA General Board (2019-2024)
- Member of NIHR PRU Commissioning Panel 2022
- Member of the NIHR RPSC Committee (2024 -)

Rob Anderson - Disclosure of interest: none declared

For peer review only - http://bmjopen.bmj.com/site/about/guidelines.xhtml

Data-sharing statement
This is an evidence synthesis study based on published primary research, it did not generate new data. All data extracted from the included publications, along with links to each publication, can be found in the report or Appendices. Further information can be obtained from the corresponding author.

Ethics statement
This was an evidence review, based on published primary studies, so did not require ethical approval.

Information governance statement
All data used in this paper is taken from published sources: no personal data was included.

References

1. World Health Organisation. *Global patient safety action plan 2021-2030 - Towards eliminating avoidable harm in health care*. 2021. URL: https://iris.who.int/server/api/core/bitstreams/a28c34c0-089c-4f5d-a0b1-5d9c35a3cd67/content (accessed 15 December 2025).
2. Illingworth J, Fernandez Crespo R, Hasegawa K, Leis M, Howitt P, Darzi A. *The National State of Patient Safety 2024: Prioritising improvement efforts in a system under stress*. London: Imperial College London; 2024.
3. Health Services Safety Investigations Body. *Safety management systems - an introduction for healthcare*. 2023. URL: https://www.hssib.org.uk/patient-safety-investigations/safety-management-systems/investigation-report/ (accessed 24 April 2024).
4. Institute for Global Health Innovation. *National state of patient safety 2022: What we know about avoidable harm in England*: Imperial College London; 2022.
5. Vincent C, Burnett S, Carthey J. *The measurement and monitoring of safety*. London: The Health Foundation; 2013.
6. International Nuclear Safety Advisory Group. *Management of operational safety in nuclear power plants (INSAG-13)*. Vienna, Austria: International Nuclear Safety Advisory Group; 1999.
7. International Civil Aviation Organisation (ICAOSMM). *Safety management manual (Doc 9859, 4th Edition)*. Montreal, Canada: International Civil Aviation Organisation; 2018.
8. Thomas M. *A systematic review of the effectiveness of safety management systems. ATSB Transport Safety Report, Cross-modal Research Investigation, XR-2011-0*. Canberra: Australian Transport Safety Bureau; 2012.
9. Li Y, Guldenmund FW. Safety management systems: A broad overview of the literature. *Safety Science* 2018;**103**:94-123. https://doi.org/10.1016/j.ssci.2017.11.016
10. Kapur N, Parand A, Soukup T, Reader T, Sevdalis N. Aviation and healthcare: a comparative review with implications for patient safety. *JRSM Open* 2016;**7**. https://doi.org/10.1177/2054270415616548
11. OGP, IPIECA. *Operating Management System Framework for controlling risk and delivering high performance in the oil and gas industry, OGP Report No 510*. London, UK: OGP & IPIEC; 2014.
12. Macchi L, Pietikäinen E, Reiman T, Heikkilä J, Ruuhilehto K. *Patient safety management: Available models and systems*. Espoo, Finland: VTT Technical Research Centre of Finland; 2011.
13. de Blok C, Koster E, Schilp J, Wagner C. *Implementatie VMS Veiligheidsprogramma. Evaluatieonderzoek in Nederlandse ziekenhuizen*. Utrecht /Amsterdam, Netherlands: NIVEL & EMGO+; 2013.
14. Zhelev Z, de Bell S, Bethel A, Clarke M, Anderson R, Thompson Coon J. The implementation of Safety Management Systems (SMS) in healthcare: a systematic review and international comparison. *Health and Social Care Delivery Research* 2025;**13**. https://doi.org/10.3310/QPLF8546

For peer review only

15. World Bank. *World Bank country and lending groups*. 2023. URL: https://datahelpdesk.worldbank.org/knowledgebase/articles/906519-world-bank-country-and-lending-groups (accessed 31 January 2024).
16. Braithwaite J, Zurynski Y, Ludlow K, Holt J, Augustsson H, Campbell M. Towards sustainable healthcare system performance in the 21st century in high-income countries: A protocol for a systematic review of the grey literature. *BMJ Open* 2019;**9**:e025892. https://doi.org/10.1136/bmjopen-2018-025892
17. Bal R, Wagner C. Chapter 27: Patient safety in Dutch hospitals: How can we explain success? In: Braithwaite J, Mannion R, Matsuyama Y, Shekelle P, Whittaker S, Al-Adawi S, editors. *Health systems improvement across the globe: Success stories from 60 countries.*Boca Raton, FL: CRC Press; 2018:205-12.
18. de Blok C, Koster E, Schilp J, Wagner C. *Implementation of the Dutch national Patient Safety Programme (VMS Veiligheidsprogrammesma)*. Utrecht/Amsterdam, Netherlands: NIVEL & EMGO+; 2013.
19. *Tijd voor Verbinding*. no date. URL: https://www.programmatvv.nl/ (accessed 25 April 2024).
20. Royal Netherlands Standards Institute. *Dutch Technical Agreement (NTA) 8009 'Safety management system for hospitals and institutions that provide hospital care'*. Delft, Netherlands: Royal Netherlands Standards Institute; 2007.
21. Royal Netherlands Standards Institute. *Dutch Technical Agreement (NEN) 8009 'Safety management system for hospitals and institutions that provide hospital care'*. Delft, Netherlands: Royal Netherlands Standards Institute; 2018.
22. Australian Commission on Safety and Quality in Health Care. *The state of patient safety and quality in Australian hospitals 2019*. Sydney, Australia: Australian Commission on Safety and Quality in Health Care; 2019.
23. Australian Commission on Safety and Quality in Health Care. *National safety and quality health service standards. 2nd edition*. Sydney, Australia: Australian Commission on Safety and Quality in Health Care; 2021.
24. Health Standards Organisation, Canadian Patient Safety Institute. *The Candanian quality and patient safety framework for health services*. 2020. URL: https://www.healthcareexcellence.ca/en/resources/canadian-quality-and-patient-safety-framework-for-health-services/ (accessed 24 April 2024).
25. Canadian Patient Safety Institute. *Strengthening commitment for improvement together. A policy framework for patient safety*. Edmonton, AB, Canada: Canadian Patient Safety Institute; 2019.
26. Healthcare Excellence Canada, Patients for Patient Safety Canada. *Rethinking patient safety. A discussion guide for patients, healthcare providers and leaders*. 2023. URL: https://www.healthcareexcellence.ca/media/gx4l3idd/rethinking-patient-safety.pdf (accessed 24 April 2024).
27. Goldman J, Rotteau L, Flintoft V, Jeffs L, Baker G. Measurement and monitoring of safety framework: a qualitative study of implementation through a Canadian learning collaborative. *BMJ Qual Saf* 2023;**32**:470. https://doi.org/10.1136/bmjqs-2022-015017
28. Health Information and Quality Authority. *National standards for safer better healthcare*. Dublin, Ireland: Health Information and Quality Authority; 2012.
29. Health Information and Quality Authority. *Standards development framework: a principles-based approach*. Dublin, Ireland: Health Information and Quality Authority; 2021.
30. Health Service Executive. *Patient safety strategy 2019-2024*. Dublin, Ireland: Health Service Executive; 2019.
31. Health Service Executive. *Incident management framework*. Dublin, Ireland: Health Service Executive; 2020.
32. Health Information and Quality Authority. *National standards for the conduct of reviews of patient safety incidents*. Dublin, Ireland: Health Information and Quality Authority; 2017.
33. Health Service Executive. *Healthcare audit – quality assurance and verification. Standards.* Dublin, Ireland: Health Service Executive; 2019.

For peer review only - http://bmjopen.bmj.com/site/about/guidelines.xhtml

34. Health Service Executive. *HSE Enterprise risk management policy and procedures 2023*. Dublin, Ireland: Health Service Executive; 2023.
35. Byrne D, O'Dowd E, Lydon S, McDermott BR, O'Connor P. *The national simulation strategic guide for the implementation of simulation on clinical sites*. Galway, Ireland: National University of Ireland Galway; 2021. https://doi.org/10.13025/cn0z-bp50
36. Irish Assoication for Simulation. *Irish Association for Simulation*. 2024. URL: https://www.iassim.com/about-us (accessed 08 April 2024).
37. Ministry of Health. *Pae Ora (Healthy Futures) Act 2022*. Wellington, New Zealand: New Zealand Government; 2022.
38. Standards New Zealand. *NZS 8134:2021 Ngā Paerewa Health and disability services standard*. Wellington: Standards New Zealand; 2021.
39. Te Tāhū Hauora Health Quality & Safety Commission. *Te Kaupapa Here hei tuku pūrongo mō ngā 'Aituā ā-Motu': National adverse events reporting policy*. Wellington, Aotearoa New Zealand: Te Tāhū Hauora Health Quality & Safety Commission; 2023.
40. Te Tāhū Hauora Health Quality & Safety Commission. *Clinical governance framework: collaborating for quality (draft, November 2023)*. Wellington, Aotearoa New Zealand: Te Tāhū Hauora Health Quality & Safety Commission; 2023.
41. Te Tāhū Hauora Health Quality & Safety Commission. *National adverse events reporting policy*. 2023).
42. Te Tāhū Hauora Health Quality & Safety Commission. *Clinical governance: guidance for health and disability providers*. Wellington, Aotearoa New Zealand: Te Tāhū Hauora Health Quality & Safety Commission; 2017.
43. Langelaan M, Baines RJ, Broekens MA, Siemerink KM, van de Steeg L, Asscheman H*, et al. Monitor zorggerelateerde schade 2008: Dossieronderzoek in Nederlandse ziekenhuizen*. Utrecht, Amsterdam: NIVEL en EMGO+ Instituut; 2010.
44. Langelaan M, de Bruijne MC, Baines RJ, Broekens MA, Hammink K, Schilp J*, et al. Monitor Zorggerelateerde Schade 2011/2012: Dossieronderzoek in Nederlandse ziekenhuizen* Utrecht, Amsterdam: NIVEL & EMGO+ Instituut; 2013.
45. Baines R, Langelaan M, de Bruijne M, Spreeuwenberg P, Wagner C. How effective are patient safety initiatives? A retrospective patient record review study of changes to patient safety over time. *BMJ Qual Saf* 2015;**24**:561-71. https://doi.org/10.1136/bmjqs-2014-003702
46. Health Information and Quality Authority. *Overview report of five years of HIQA monitoring in Irish public acute hospitals against national standards: 2015–2019*. Dublin, Ireland: Health Information and Quality Authority; 2020.
47. Australian Commission on Safety and Quality in Health Care. *Creating safer, better health care - the impact of the national safety and quality health service standards*. Sydney, Australia: Australian Commission on Safety and Quality in Health Care; 2018.
48. Institute of Medicine (US) Committee on Quality of Health Care in America. *To err is human: building a safer health system*. Washington, DC: National Academies Press (US); 2000.
49. Hollnagel E, Wears RL, Braithwaite J. *From safety-I to safety-II: A White Paper*: The Resilient Health Care Net: Published simultaneously by the University of Southern Denmark, Denmark, University of Florida, USA, and Macquarie University, Australia; 2015.
50. Te Tāhū Hauora Health Quality & Safety Commission. *Guide to doing a learning review*. Wellington, Aotearoa New Zealand: Te Tāhū Hauora Health Quality & Safety Commission; 2023.
51. Hollnagel E. *Resilience Assessment Grid (RAG)*. 2024. URL: https://erikhollnagel.com/ideas/resilience%20assessment%20grid.html (accessed 15/04/2024).
52. Te Tāhū Hauora Health Quality & Safety Commission. *Mahi haumanu, hohou te rongo. Restorative practice and hohou te rongo*. 2023. URL: https://www.hqsc.govt.nz/our-work/system-safety/healing-learning-and-improving-from-harm-policy/restorative-practice-and-hohou-te-rongo/ (accessed 22 April 2024).

For peer review only - http://bmjopen.bmj.com/site/about/guidelines.xhtml

53. Aven T. What is safety science? *Saf Sci* 2014;**67**:15-20. https://doi.org/doi.org/10.1016/j.ssci.2013.07.026

For peer review only

For peer review only - http://bmjopen.bmj.com/site/about/guidelines.xhtml

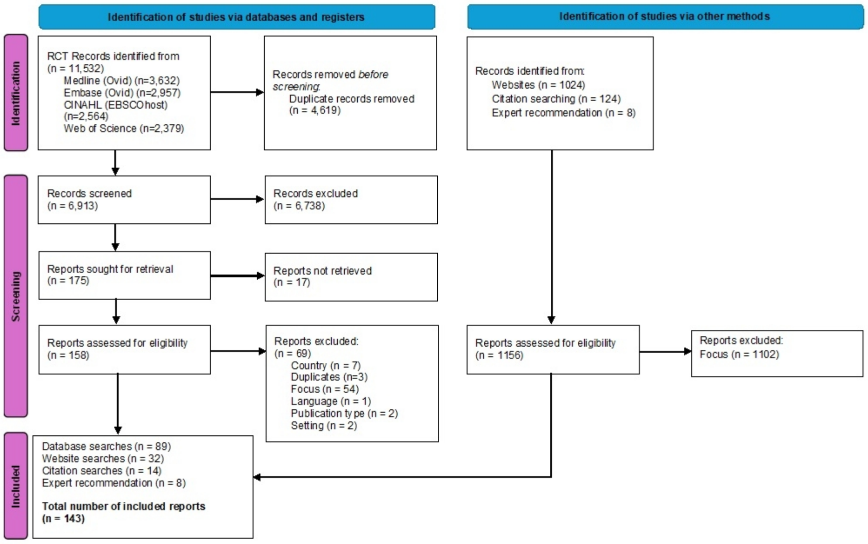

Figure 1 PRISMA flow diagram of the screening process

304x184mm (96 x 96 DPI)

Supplementary material 1: Protocol

The implementation of Safety Management Systems (SMS) in healthcare

Systematic review protocol

Version 5

24 November 2023

For peer review only - http://bmjopen.bmj.com/site/about/guidelines.xhtml

**Produced by**	Exeter HS&DR Evidence Synthesis Centre<br>Department of Health & Community Sciences<br>South Cloisters<br>St Luke's Campus<br>Heavitree Road<br>Exeter<br>EX1 2LU<br>UK
**Authors**	**Zhivko Zhelev** Systematic Reviewer [1]<br>z.zhelev@exeter.ac.uk<br>**Sian de Bell** Systematic Reviewer [1]<br>s.c.de-bell@exeter.ac.uk<br>**Alison Bethel** Information Specialist[1]<br>a.bethel@exeter.ac.uk<br>**Maria Clarke** Information Specialist[1]<br>m.e.clarke2@exeter.ac.uk<br>**Rob Anderson** Senior/Academic Lead[1]<br>r.anderson@exeter.ac.uk<br>**Jo Thompson Coon** Senior/Academic Lead[1]<br>j.thompson-coon@exeter.ac.uk<br><br>[1] University of Exeter
**Correspondence to**	z.zhelev@exeter.ac.uk
**Date completed**	24 November 2023
**Source of funding**	This work was commissioned by the NIHR HSDR programme as a project (project code NIHR136105) within grant number NIHR130538.
**Role of funder/institution in protocol creation**	The aims and scope of this protocol and the planned work were informed by information provided by and discussions with staff in the NHS Patient Safety Team and Department of Health & Social Care

For peer review only - http://bmjopen.bmj.com/site/about/guidelines.xhtml

**Declared competing interests of the authors**	None
**Rider on responsibility for document**	The views and opinions expressed by authors in this publication are those of the authors and do not necessarily reflect those of the NHS, the NIHR, NETSCC, the HSDR programme or the Department of Health & Social Care. Any errors are the responsibility of the authors.
**PROSPERO registration number**	CRD42023487512

For peer review only

For peer review only - http://bmjopen.bmj.com/site/about/guidelines.xhtml

Table of Contents

1 Background....5

1.1 Development of the review....6

1.2 Research questions....7

2 Methods....8

2.1 Approach to evidence synthesis....8

2.2 Identification of studies....9

2.2.1 Searches....9

2.2.2 Inclusion and exclusion criteria....9

2.2.3 Process for applying inclusion criteria....11

2.3 Data extraction....11

2.4 Study quality assessment strategy....12

2.5 Data analysis and presentation....12

3 Stakeholder and patient/public involvement....14

4 Dissemination plans....15

5 References....16

For peer review only

For peer review only - http://bmjopen.bmj.com/site/about/guidelines.xhtml

1 Background

There are a number of industries, from aviation to nuclear power, in which safety is a priority. Within these industries, failures or errors could have serious consequences for people, causing loss of life or injury, and for the environment, in terms of environmental damage or harm to equipment or property.(1) Healthcare is now considered to be one of these 'safety-critical industries', with safety in this setting defined as:

> "The avoidance, prevention and amelioration of adverse outcomes or injuries stemming from the process of healthcare."(2)

Examples of safety incidents in healthcare include medication errors, wrong site surgery, and lack of a timely response to deterioration in a patient's condition.

High-risk industries have improved safety by moving from measuring and responding to specific incidents, to assessing the presence of, and managing, the conditions that lead to hazards.(3) This requires a systems thinking approach, considering interactions and intersections between social and technological elements, which are where safety events tend to occur.(3, 4) Change in some areas may be slow, such as to structural factors e.g. physical infrastructure or the organisation of departments, whereas in other areas of the system, particularly those dependent on more transient 'mediating' factors such as morale, teamwork, and individual performance, change may be affected more rapidly.(3)

Within many safety-critical industries, a systems approach to safety has been operationalised in the form of safety management systems (SMS): "an organised approach to managing safety, including the necessary organisational structures, accountabilities, policies and procedures".(5) These are generally considered to have four key components:

- Policy and objectives,
- Risk management,
- Safety assurance,
- Safety promotion (safety culture).(5)

These are sometimes expanded into six areas, offering further detail on considerations and requirements within the four key components identified above: (i) a safety policy; (ii) organisational arrangements to support safety e.g. supervision and training of staff in implementing the safety policy and processes; (iii) a safety plan, detailing standards and processes for safety, including risk assessment; (iv) measurement of safety performance; (v)

For peer review only - http://bmjopen.bmj.com/site/about/guidelines.xhtml

reviews of safety performance e.g. incident investigation, safety audits; and (vi) feedback loops to improve safety performance.(3)

A systematic review of SMS in three safety-critical industries - aviation, marine and rail – published in 2012 indicated that they led to improvements in safety e.g. reduced accident rates.(6) There is some debate over whether indicators such as accident rates are an appropriate way of measuring the effectiveness of SMS,(7) but this does suggest that they have the potential to improve patient safety in organisations providing healthcare. However, whilst the basic principles of SMS are widely accepted as transferrable,(4, 7) evidence from other industries suggests that the details (e.g. of accountability frameworks) need to be context-specific to be effective.(8) In healthcare, this might involve identifying and improving the management of known patient safety risks.(9) Components of SMS, such as national strategies for improving patient safety,(4) exist in most healthcare systems, and some countries, such as Australia (10) and the Netherlands,(11) are explicitly developing and promoting safety management systems. Their experiences offer opportunities for learning that could support the implementation of the principles of SMS in other healthcare systems, which will have different starting points in terms of the organisation and management of healthcare, and different pre-existing system components.

1.1 Development of the review

This review of research and other evidence has been commissioned by the NIHR HSDR programme to provide evidence and information to NHS England and the NHS Patient Safety Team. It is primarily intended to inform the further development of the NHS England's patient safety policy and practice as laid out in the NHS Patient Safety Strategy 2019(12) and related documents.

The policy questions of interest detailed in the project brief were:

Q1: What are the key attributes of a successful SMS for the NHS in England?

Q2: What are the links between a SMS and a Quality Management System?

Q3: What are the next steps for safety management in the NHS in England?

Evidence is emerging that could begin to answer these questions following the implementation of NHS Patient Safety Strategy 2019.(12) For example, the work of the National Patient Safety team is saving an estimated 160 lives per year (13), and evaluation of the Patient Safety Incident Response Framework (PSIRF) early adopter programme indicates it has led to improved safety cultures and identification of more effective risk reduction strategies in participating organisations.(13) A recent report published by the

For peer review only - http://bmjopen.bmj.com/site/about/guidelines.xhtml

Healthcare Safety Investigation Branch (HSIB)(14) has used evidence on the implementation of SMS in a range of high-risk industries, from aviation to the oil and gas industry, to describe key attributes of SMS at a broad level and make recommendations for the further development of a SMS in the NHS. However, more detailed evidence is needed to assess how a SMS approach might be applicable to the NHS in England.

The review described in this protocol aims to provide information on SMS in healthcare specifically, by investigating how they have been implemented in other countries. This will offer context to inform primary research into the questions raised above, and consideration of how NHS England's current patient safety policy, processes and practice could be extended into a full SMS.

1.2 Research questions

In high-income countries applying the SMS approach (as specified below), what principles have been used to inform healthcare policy, management and practice related to patient and staff safety and what is the evidence supporting their adoption and implementation?

Within this broader question, our specific questions are:

How are the components of SMS reflected in the healthcare policy documents of the above countries, or their regional healthcare systems?

What research (e.g. formal evaluations, or based on routine data) or other relevant evidence (e.g. staff and patient narratives) is available regarding the effectiveness, implementation or experience of SMS within healthcare?

What does existing research and other relevant evidence from beyond the UK tell us about the effectiveness, implementation or experience of SMS within healthcare?

For peer review only - http://bmjopen.bmj.com/site/about/guidelines.xhtml

2 Methods

2.1 Approach to evidence synthesis

As noted above, there is a paucity of empirical evidence on the use of SMS in healthcare. Also, the specific application of such systems in healthcare is likely to depend on the local context, reflecting pre-existing arrangements as well as current priorities and initiatives. To better understand the context of each application and given the expectation of limited empirical evidence, we will conduct a systematic review with a broader scope, including, in addition to research evidence, an analysis of policy documents, training materials and other relevant information (e.g. patients and healthcare professionals' narratives). This will allow better understanding of the logic and process of each implementation; more adequate interpretation of the available empirical evidence; better understanding of the similarities and differences across systems; and the identification of specific barriers, facilitators and challenges that need to be addressed in future research. The review will follow the process detailed below.

Firstly, we will take the four pillars of a SMS as defined by HSIB (14), and compare them to other key SMS literature e.g. documents from safety-critical industries such as aviation (5). In consultation with stakeholders, we will produce an expanded and more detailed framework of the components of a SMS. This will be used to inform data extraction and analysis, to allow us to identify the aspects of SMS that have and have not been adopted by different healthcare systems.

Secondly, we will identify national or regional healthcare systems in which SMS principles have been used to inform policy and practice related to patient and staff safety. For pragmatic reasons, we will focus first on high-income English-speaking countries and, if necessary and feasible, include other countries after discussion with stakeholders. If key policy documents do not state explicitly that a SMS approach was used, then we will contact relevant organisations to enquire about this. Only healthcare systems where such a link and commitment are explicitly stated will be included in the review.

Thirdly, we will identify relevant policy documents, research and other relevant information (e.g. patient stories) from the organisations' websites and by focused searches of electronic databases and other sources. Each organisation will be contacted to ascertain that all relevant information has been captured and request additional data that may have been missed or is not available online.

Fourthly, we will develop a data extraction form organised around the core principles of SMS (as defined in the background) and the different types of evidence we are including: policy

For peer review only - http://bmjopen.bmj.com/site/about/guidelines.xhtml

documents, research and other evidence. If possible, data will be extracted verbatim or, if too long, summarised with reference to its location in the source document. Rather than just depicting a snapshot of the *status quo*, as much as possible, the information gathered will attempt to distinguish the sequence and timings of the adoption and application of different core principles of SMS, or of the main strategies and processes to implement and embed them in a particular country or region/province. This therefore aims to shed light on which principles and systems/processes may have pre-existed the formal policy commitment to adopting a SMS approach, which were seen as most critical for expanding the scope and depth of the SMS, and interactions between the different components of SMS.

Finally, information on the implementation of SMS in the eligible healthcare systems will be synthesised narratively and summarised in tables and graphs. Common themes across different healthcare systems will be identified, evidence gaps will be highlighted, and recommendations on how to address those will be made.

2.2 Identification of studies

2.2.1 Searches

The main method for identifying relevant policy documents, research and other evidence will be through searching the websites of, and direct contact with, relevant organisations (e.g. Australian Commission on Safety and Quality in Healthcare). Website searching will be carried out either by browsing publication lists or using site search functionality. Search parameters (e.g. keywords, publication date) will be pre-specified and the search process will be documented (e.g. number of hits, number of potentially relevant titles) to allow transparency and reproducibility.

The above will be complemented by focused database searches using search strategies developed by an information specialist (AB) in consultation with the review team and the commissioning group. The database search strategies will use both controlled vocabulary when available and relevant (e.g. MeSH in MEDLINE) and free-text searching. Search terms will be partly derived from the literature and from stakeholders and experts' input. Searches will be limited to English-language texts published after a pre-specified date (to be agreed with stakeholders and experts). The reference lists of all relevant documents will be hand-searched for additional titles.

2.2.2 Inclusion and exclusion criteria

The following overarching inclusion and exclusion criteria will be applied:

For peer review only - http://bmjopen.bmj.com/site/about/guidelines.xhtml

- **Safety management system:** Safety management systems are defined by the following key elements:
  - policy and objectives
  - risk management
  - safety assurance
  - safety promotion (safety culture).

  Texts will be excluded if they refer to different concepts or models without direct link to the above definition of a safety management system.

- **Application of SMS** in the healthcare system of a high-income country (as defined by the World Bank). For pragmatic reasons, we will focus on English-speaking countries; if necessary and feasible, and after discussion with our policy costumer, we may extend the scope to include other, selected high-income countries.

- **Type of evidence:** we will include three distinct types of evidence:
  - Relevant policy documents, including documents related to the implementation of SMS, such as staff training and patient-facing materials
  - Published or unpublished research / evaluations (quantitative, qualitative or mixed methods),
  - Other evidence, e.g. patient and staff experience or opinions published on the organisations' websites.

- **Provenance of evidence:** As mentioned earlier, for pragmatic reasons we will focus first on high-income English-speaking countries as evidence from such healthcare systems is more likely to be relevant to the UK and all relevant policy documents and research will be available in English. Therefore, evidence related to the implementation of SMS in the healthcare of the following countries will be considered first: Ireland, Australia, New Zealand, Canada and the Netherlands. The list will be revised / amended once the initial searches have been completed and the volume, type and quality of evidence is assessed; or, it has been decided in discussion with experts and stakeholders that including a specific country or expanding the list is desirable and feasible.

For peer review only

- **Language:** we will include texts available in English; if the scope of the review is extended (see above) we may decide to include texts in other languages, if necessary and feasible.

2.2.3 Process for applying the inclusion criteria

A small sample (n ~ 10) of potentially relevant documents from the three types of evidence will be used to calibrate the selection process. Each document will be reviewed by multiple reviewers. Decisions will be compared and discussed in a group meeting to ensure consistent application of criteria. If necessary, definitions will be updated and explanatory notes added to aid the process.

The full text of all potentially relevant documents identified through the website searches and direct contact with the healthcare organisations will be retrieved and checked for relevance against the inclusion criteria independently by two reviewers.

The results from the database searches will be screened following the standard process. Two reviewers will independently apply the inclusion and exclusion criteria to the title and abstract of each identified citation. We will obtain the full text of papers where either reviewer judges it to be a potential inclusion. Two reviewers will assess the full text of each record independently for inclusion, with disagreements settled through discussion or by a third reviewer. The full texts of all potentially relevant publications identified from the website searches will be obtained and screened as per the above process.

The study selection process will be detailed using a PRISMA-style flowchart, with a reason reported for exclusion of each record assessed at full text.

2.3 Data extraction

A data extraction form will be developed and piloted independently by two reviewers on a small sample of included documents. Data will be extracted by one reviewer and checked by a second reviewer. Disagreements will be resolved through discussion.

The following data will be extracted:

- Country
- Healthcare organisation (purpose and remit, website)
- Type of document (policy, research, anecdotal evidence)
- Aims

For peer review only - http://bmjopen.bmj.com/site/about/guidelines.xhtml

- Relation to SMS (e.g. explicitly stated or implied, description)
- Information regarding specific SMS components:
    - Policy and objectives
    - Risk management
    - Safety assurance
    - Safety promotion (safety culture).
- Information regarding implementation and/or impact
- **Research:** research design, aim, methods, results, conclusions
- **Other evidence:** type of document, informant (patient, healthcare professional, policy maker), perspective (relationship to the programme), main points.

The NIHR-INCLUDE guidelines (15) were used to reflect on Equality, Diversity and Inclusion (EDI) whilst designing the protocol. EDI is important to consider in relation to the topic of safety management systems, as there may be differences in safety incidents between social and demographic groups and resulting inequities in health outcomes. However, it is likely this information will be difficult to ascertain from the types of evidence e.g. policy documents, we will be including in the review. We will consider PROGRESS-Plus characteristics during the process of data extraction and analysis (16, 17) and, where possible, use this information to inform the findings and recommendations of the review.

2.4 Study quality assessment strategy

The methodological quality of all research studies will be assessed using validated quality assessment tools according to the specific research design. Quality will be assessed by two reviewers independently and disagreements resolved through discussion or arbitration. No quality appraisal will be carried out on policy documents or non-research evidence.

2.5 Data analysis and presentation

The type of information identified for each healthcare system will be summarised and the following information will be reported:

- The way in which the SMS approach has informed patient and staff safety policy and practice.
- Information related to each of the SMS components (see above).

For peer review only

For peer review only - http://bmjopen.bmj.com/site/about/guidelines.xhtml

- Information related to the process and timing of implementing SMS (e.g. steps, processes etc.).
- Evidence related to the effectiveness / impact and implementation of SMS.
- Stakeholders' perceptions and experience.

Comparisons across healthcare systems will be carried out to identify similarities and differences, including barriers, facilitators and challenges; the volume, type and quality of the available evidence and any evidence gaps that need to be addressed in future research. The results will be summarised in tables and diagrams and presented narratively, considering their relevance to the application of SMS in the NHS of England.

For peer review only

For peer review only - http://bmjopen.bmj.com/site/about/guidelines.xhtml

3 Stakeholder and patient/public involvement and engagement

The following stakeholders will be involved:

- NHS England's Patient Safety Team and the Department of Health and Social Care (as commissioners of the work)
- Topic experts (including from other countries and other industries, if possible)
- Any other relevant Patient Safety organisations (NHS or external) e.g. local patient safety teams

Due to the focus of the review, we will consult Public and Patient Involvement and Engagement representatives who have some knowledge of the topic e.g. members of local patient safety teams, or PPIE representatives who have worked with the National Patient Safety Team.

We will consult stakeholders throughout the review process, to discuss topics such as the focus of the review, analysis, and findings. Meetings will be arranged to suit the project progress and stakeholder availability. We will also ask for their input on dissemination plans and materials as described below.

For peer review only

For peer review only - http://bmjopen.bmj.com/site/about/guidelines.xhtml

4 Dissemination plans

We will produce a report on the implementation of safety management systems in healthcare in high-income countries, available as an open access in the NIHR Journals Library.

Further materials for dissemination will be finalised after discussion with the project's advisory group. These are likely to include:

- an evidence briefing, giving a plain language summary of the report and its findings (primarily aimed at policy makers and health service managers);
- an article in an academic journal identified as being relevant to stakeholders for this review; and
- presentations at key national and regional meetings.

Outputs will be disseminated via the Exeter HSDR Evidence Synthesis Centre webpage and social media. Additional material may be produced to promote them, such as a blog post based on the evidence briefing and report.

For peer review only

For peer review only - http://bmjopen.bmj.com/site/about/guidelines.xhtml

5 References

1. Saunders F. Safety-critical industries: definitions, tensions and tradeoffs 2015 [Available from: http://fionasaunders.co.uk/safety-critical-industries-definitions-tensions-and-tradeoffs/#:~:text=Combining%20these%20two%20definitions%2C%20a%20safety-critical%20industry%20can,environmental%20damage%2C%20or%20harm%20to%20plant%20or%20property.

2. Vincent C. Patient safety. 2nd edition ed. Chichester: John Wiley and Sons; 2010.

3. Vincent C, Burnett S, Carthey J. The measurement and monitoring of safety. London: The Health Foundation; 2013.

4. Macchi L, Pietikäinen E, Reiman T, Heikkilä J, Ruuhilehto K. Patient safety management: Available models and systems. VTT; 2011.

5. International Civil Aviation Organisation Safety Management Manual (ICAOSMM). 2nd ed. Doc 9859, Safety Management Manual (SMM) 2009 [Available from: www2.icao.int/en/ism/Guidance%20Materials/DOC_9859_FULL_EN.pdf.

6. Thomas M. A systematic review of the effectiveness of safety management systems. ATSB Transport Safety Report, Cross-modal Research Investigation, XR-2011-0. Canberra: Australian Transport Safety Bureau; 2012.

7. Li Y, Guldenmund FW. Safety management systems: A broad overview of the literature. Safety Science. 2018;103:94-123.

8. Kapur N, Parand A, Soukup T, Reader T, Sevdalis N. Aviation and healthcare: a comparative review with implications for patient safety. JRSM Open. 2016;7.

9. HSIB. A thematic analysis of HSIB's first 22 national investigations. Healthcare Safety Investigation Branch I2020/016; 2021.

10. Australian Commission on Safety and Quality in Health Care. The state of patient safety and quality in Australian hospitals 2019. Sydney: Australian Commission on Safety and Quality in Health Care; 2019.

11. Bolk M. Final report: Embedding patient safety in education & training. VMS Safety Program; 2013.

12. NHS England. The NHS Patient Safety Strategy no date [Available from: https://www.england.nhs.uk/patient-safety/the-nhs-patient-safety-strategy/#key-docs.

13. NHS England. NHS patient safety strategy – progress so far no date [Available from: https://www.england.nhs.uk/patient-safety/the-nhs-patient-safety-strategy/nhs-patient-safety-strategy-progress-so-far/.

14. HSIB. Safety management systems - an introduction for healthcare. 2023.

15. National Institute for Health Research. Improving inclusion of under-served groups in clinical research: Guidance from the NIHR-INCLUDE project. UK: National Institute for Health Research; 2020.

For peer review only - http://bmjopen.bmj.com/site/about/guidelines.xhtml

16. O'Neill J, Tabish H, Welch V, Petticrew M, Pottie K, Clarke M, et al. Applying an equity lens to interventions: using PROGRESS ensures consideration of socially stratifying factors to illuminate inequities in health. J Clin Epidemiol. 2014;67:56-64.

17. Cochrane Methods Equity. PROGRESS-Plus no date [Available from: https://methods.cochrane.org/equity/projects/evidence-equity/progress-plus.

For peer review only

For peer review only - http://bmjopen.bmj.com/site/about/guidelines.xhtml

Supplementary material 2: Inclusion criteria

	Include	Exclude
**Population**	Populations with any health need.	-
**Initiative**	Any use of an SMS approach in a healthcare setting, with an SMS being defined by four key components:<br>• policy and objectives<br>• risk management<br>• safety assurance<br>• safety promotion (safety culture).<br><br>Where it is not clear whether a country is using an SMS approach, we will include studies if they relate to one or more components of an SMS.	Studies of safety where this is not linked to an SMS component.<br>Studies of occupational health and safety.
**Setting**	Any healthcare setting:<br>- Secondary care,<br>- Primary care.	Non-healthcare settings:<br>- Care homes,<br>- Home care.
**Country**	Australia, Canada, Ireland, the Netherlands, New Zealand.<br><br>Studies which included other countries were included if the results for these countries were reported separately.	Any other country.
**Language**	English or Dutch.	Any other language.

For peer review only

For peer review only - http://bmjopen.bmj.com/site/about/guidelines.xhtml

	Include	Exclude
**Type of document**	• Relevant policy documents, including documents related to the implementation of SMS, such as staff training and patient-facing materials<br>• Published or unpublished research / evaluations (quantitative, qualitative or mixed methods),<br>• Other evidence (e.g. patient and staff experience or opinions published on the organisations' websites).	Theoretical

For peer review only

Supplementary material 3: Excluded and thin studies

Excluded papers from database searching (n=68)

Excluded on country (n=7)

Ma E, Wei W, Ho C. An environmental scan of patient safety reporting and learning systems in community healthcare for multi- disciplinary teams. *Can J Hosp Pharm*. 2022;**75**(2):135.

Coffey M, Marino M, Lyren A, Purcell D, Hoffman JM, Brilli R, et al. Association between hospital-acquired harm outcomes and membership in a national patient safety collaborative. *JAMA Pediatrics*. 2022;**176**(9):924-32.

Kristensen S, Hammer A, Bartels P, Sunol R, Groene O, Thompson CA, et al. Quality management and perceptions of teamwork and safety climate in European hospitals. *Int J Qual Health Care*. 2015;**27**(6):499-506.

Holmstrom AR, Laaksonen R, Airaksinen M. How to make medication error reporting systems work - Factors associated with their successful development and implementation. *Health Policy*. 2015;**119**(8):1046-54.

Windsor JA, Maxim S. Patient safety in medicine: Are surgeons ready for checklists? *ANZ Journal of Surgery*. 2010;**80**(1-2):3-5.

Sunol R, Vallejo P, Groene O, Escaramis G, Thompson A, Kutryba B, et al. Implementation of patient safety strategies in European hospitals. *Qual Saf Health Care. 2009*;**18**(Suppl 1):i57-61.

Shaw C, Kutryba B, Crisp H, Vallejo P, Sunol R. Do European hospitals have quality and safety governance systems and structures in place? *Qual Saf Health Care*. 2009;**18**(Suppl 1):i51-6.

Excluded on focus (n=54)

Ong N, Lucien A, Long JC, Weise J, Walton M, Burgess A. What do healthcare staff think about the quality and safety of care provided to children and young people with an intellectual disability? A qualitative study using the framework method of analysis. *BMJ Open*. 2023;**13**(7):e071494.

Kaud Y, McKeon D, Lydon S, O'Connor P. Measuring and monitoring patient safety in hospitals in the Republic of Ireland. *Irish J Med Sci*. 2023;1**92:**2581–259.

For peer review only - http://bmjopen.bmj.com/site/about/guidelines.xhtml

Cords CI, van Baar ME, Nieuwenhuis MK, Pijpe A, van der Vlies CH. Reliability and validity of a frailty assessment tool in specialized burn care, a retrospective multicentre cohort study. *Burns*. 2023;**49**(7):1621-31.

Terry D, Kim J-a, Gilbert J, Jang S, Nguyen H. "Thank You for Listening": An Exploratory Study Regarding the Lived Experience and Perception of Medical Errors Among Those Who Receive Care. *International Journal of Health Services*. 2022;**52**(2):292-302.

Arnold A, Ward I, Gandhidasan S. Incident review in radiation oncology. *J Med Imaging Radiat Oncol*. 2022;**66**(2):291-8.

Malik RF, Buljac-Samardzic M, Amajjar I, Hilders C, Scheele F. Open organisational culture: what does it entail? Healthcare stakeholders reaching consensus by means of a Delphi technique. *BMJ Open*. 2021;**11**(9):e045515.

Connell CJ, Cooper S, Endacott R. Measuring the safety climate in an Australian emergency department. *Int Emerg Nurs*. 2021;**58**:101048.

Adie K, Fois RA, McLachlan AJ, Walpola RL, Chen TF. The nature, severity and causes of medication incidents from an Australian community pharmacy incident reporting system: The QUMwatch study. *Br J Clin Pharmacol*. 2021;**87**(12):4809-22.

Wright KM, Bonser M. The Essential Steps of Medication Administration Practices Project medication administration improvement practices among acute inpatients in a tertiary hospital: a best practice implementation project. *International Journal of Evidence-Based Healthcare*. 2020;**18**(4):408-19.

Floyd E, Hodgkins R, Naylon L, Noon M, Sirotti L, Monaro S. The costs and benefits of pressure injury point prevalence auditing. *Wound Practice & Research*. 2020;**28**(2):66-74.

Emond Y, Wolff AP, Peters YAS, Bloo GJA, Westert GP, Damen J, et al. Reducing work pressure and IT problems and facilitating IT integration and audit & feedback help adherence to perioperative safety guidelines: a survey among 95 perioperative professionals. *Implement*. 2020;**1**:49.

Clay-Williams R, Taylor N, Winata T, Ting HP, Arnolda G, Braithwaite J. Organization quality systems and department-level strategies: refinement of the Deepening our Understanding in Quality in Australia (DUQuA) organization and department-level scales. *Int J Qual Health Care*. 2020;**32**(Supplement_1):22-34.

Braithwaite J, Taylor N, Clay-Williams R, Ting HP, Arnolda G. Conclusion: the road ahead: where should we go now to improve healthcare quality in acute settings? *Int J Qual Health Care*. 2020;**32**(Supplement_1):99-103.

Ward M, Ni She E, De Brun A, Korpos C, Hamza M, Burke E, et al. The co-design, implementation and evaluation of a serious board game 'PlayDecide patient safety' to educate junior doctors about patient safety and the importance of reporting safety concerns. *BMC Med Educ*. 2019;**19**(1):232.

For peer review only - http://bmjopen.bmj.com/site/about/guidelines.xhtml

For peer review only

McHugh S, Droog E, Foley C, Boyce M, Healy O, Browne JP. Understanding the impetus for major systems change: A multiple case study of decisions and non-decisions to reconfigure emergency and urgent care services. *Health Policy*. 2019;**123**(8):728-36.

Liao E, Eisenberg N, Kaushal A, Montbriand J, Tan KT, Roche-Nagle G. Utility of the Vascular Quality Initiative in improving quality of care in Canadian patients undergoing vascular surgery. *Can J Surg*. 2019;**62**(1):66-9.

Eljiz K, Greenfield D, Derrett A, Radmore S. Health system redesign: Changing thoughts, values, and behaviours for the co-production of a safety culture. *Int J Health Plann Manage*. 2019;**34**(4):1477-84.

Veitch D. One province, one healthcare system: A decade of healthcare transformation in Alberta. *Healthc Manage Forum*. 2018;**31**(5):167-71.

Sinclair JE, Austin MA, Bourque C, Kortko J, Maloney J, Dionne R, et al. Barriers to self-reporting patient safety incidents by paramedics: A mixed methods study. *Prehospital Emergency Care*. 2018;**22**(6):762-72.

Khalil H, Lee S. Medication safety challenges in primary care: Nurses' perspective. *Journal of Clinical Nursing*. 2018;**27**(9-10):2072-82.

Debono D, Taylor N, Lipworth W, Greenfield D, Travaglia J, Black D, et al. Applying the Theoretical Domains Framework to identify barriers and targeted interventions to enhance nurses' use of electronic medication management systems in two Australian hospitals. *Implement Sci*. 2017;**12**(1):42.

Davies JM, Delaney G. Can the Aviation Industry be Useful in Teaching Oncology about Safety? *Clin Oncol*. 2017;**29**(10):669-75.

Blijleven V, Koelemeijer K, Wetzels M, Jaspers M. Workarounds emerging from electronic health record system usage: Consequences for patient safety, effectiveness of care, and efficiency of care. *JMIR Hum Factors*. 2017;**4**(4):e27.

Wagner C, Merten H, Zwaan L, Lubberding S, Timmermans D, Smits M. Unit-based incident reporting and root cause analysis: variation at three hospital unit types. *BMJ Open*. 2016;**6**(6):e011277.

Ng J, Andrew P, Crawley M, Pevreal W, Peach J. Assessing a hospital medication system for patient safety: findings and lessons learnt from trialling an Australian modified tool at Waitemata District Health Board. *N Z Med J*. 2016;**129**(1430):63-77.

Gill FJ, Leslie GD, Marshall AP. Family initiated escalation of care for the deteriorating patient in hospital: Family centred care or just "box ticking". *Aust Crit Care*. 2016;**29**(4):195-200.

Yates M, Reddy M, Machumpurath B, Phelps G, Hampson SA. Modification of the National Inpatient Medication Chart improves venous thromboembolism prophylaxis rates in high-risk medical patients. *Intern Med J*. 2014;**44**(2):190-4.

For peer review only - http://bmjopen.bmj.com/site/about/guidelines.xhtml

Wong KS, Ryan DP, Liu BA. A system-wide analysis using a senior-friendly hospital framework identifies current practices and opportunities for improvement in the care of hospitalized older adults. *J Am Geriatr Soc*. 2014;**62**(11):2163-70.

Teare GF. Measurement of quality and safety in healthcare: the past decade and the next. *Healthc Q*. 2014;**17 Spec No**:45-50.

Russell L, Dawda P. Lessons for the Australian healthcare system from the Berwick report. *Aust Health Rev*. 2014;**38**(1):106-8.

Berg M, Black G. A Canadian perspective on clinical governance. *Clinical Governance: An International Journal*. 2014;**19**(4):314-21.

van Beuzekom M, Boer F, Akerboom S, Dahan A. Perception of patient safety differs by clinical area and discipline. *Br J Anaesth*. 2013;**110**(1):107-14.

Levi M. Towards a hospital-wide integrated system for quality and safety of health care. Neth J Med. 2013;**71**(1):2-3.

Marsden J, van Dijk M, Doris P, Krause C, Cochrane D. Improving care for British Columbians: the critical role of physician engagement. *Healthc Q*. 2012;**15 Spec No**:51-5.

Krause C, Cochrane D. BC Patient Safety & Quality Council: using network and social movement theory to improve healthcare. *Healthc Manage Forum*. 2012;**25**(4):181-4.

Ilan R, Squires M, Panopoulos C, Day A. Increasing patient safety event reporting in 2 intensive care units: a prospective interventional study. *J Crit Care*. 2011;**26**(4):431.e11-8.

Green E, White R, Janes K, Fields A, Easty A. Courage, collaboration, complexity and chemotherapy safety: the view from the sharp end. *Canadian Oncology Nursing Journal*. 2011;**21**(2):81-90.

Duckers ML, Wagner C, Vos L, Groenewegen PP. Understanding organisational development, sustainability, and diffusion of innovations within hospitals participating in a multilevel quality collaborative. *Implement Sci*. 2011;**6**:18.

van Gaal BG, Schoonhoven L, Hulscher ME, Mintjes JA, Borm GF, Koopmans RT, et al. The design of the SAFE or SORRY? study: a cluster randomised trial on the development and testing of an evidence based inpatient safety program for the prevention of adverse events. *BMC Health Serv Res*. 2009;**9**:58.

Relihan E, Silke B, O'Grady F. Internally-developed electronic reporting system for medication errors. *Ir Med J*. 2009;**102**(7):223-4.

For peer review only - http://bmjopen.bmj.com/site/about/guidelines.xhtml

For peer review only

Michel J, Nghiem HD, Jackson TJ. Using ICD-10-AM codes to characterise hospital-acquired complications. *Health Inf Manag*. 2009;**38**(3):18-25.

Neudorf K, Dyck N, Scott D, Davidson Dick D. Nursing education: a catalyst for the patient safety movement. *Healthc Q*. 2008;**11**(3 Spec No.):35-9.

Makeham MA, Saltman DC, Kidd MR. Lessons from the TAPS study--recall and reminder systems. *Aust Fam Physician*. 2008;**37**(11):923-4.

Makeham MA, Cooper C, Kidd MR. Lessons from the TAPS study - message handling and appointment systems. *Aust Fam Physician*. 2008;**37**(6):438-9.

Duckett S, Daniels S, Kamp M, Stockwell A, Walker G, Ward M. Pay for performance in Australia: Queensland's new Clinical Practice Improvement Payment. *Journal of Health Services Research and Policy*. 2008;**13**(3):174-7.

Booth BJ, Snowdon T, Harris MF, Tomlins R. Safety and quality in primary care: The view from general practice. *Australian Journal of Primary Health*. 2008;**14**(2):19-27.

David U. Medication safety... Your call to make a difference. *Alta RN*. 2007;**63**(8):10-1.

Runciman WB, Williamson JA, Deakin A, Benveniste KA, Bannon K, Hibbert PD. An integrated framework for safety, quality and risk management: an information and incident management system based on a universal patient safety classification. *Qual Saf Health Care*. 2006;**15**(Suppl 1):i82-90.

Greenberg A, Kramer S, Welch V, O'Sullivan E, Hall S. Cancer Care Ontario's computerized physician order entry system: a province-wide patient safety innovation. *Healthc Q*. 2006;**9 Spec No**:108-13.

Yassi A, Hancock T. Patient safety--worker safety: building a culture of safety to improve healthcare worker and patient well-being. *Healthc Q*. 2005;**8 Spec No**:32-8.

Greenall J, U D, Lam R. An effective tool to enhance a culture of patient safety and assess the risks of medication use systems. *Healthc Q*. 2005;**8 Spec No**:53-8.

Court D. Quest for patient safety in a challenging environment. *Aust N Z J Obstet Gynaecol*. 2003;**43**(2):97-100.

Nicklin W, McVeety JE. Canadian nurses' perceptions of patient safety in hospitals. *Can J Nurs Leadersh*. 2002;**15**(3):11-21.

For peer review only - http://bmjopen.bmj.com/site/about/guidelines.xhtml

For peer review only

Baldwin I, Beckman U, Shaw L, Morrison A. Australian Incident Monitoring Study in intensive care: local unit review meetings and report management. *Anaesth Intensive Care*. 1998;**26**(3):294-7.

Excluded on language (n=1)

Broeke RT, Lammers R, Ackerman E. The safety management system for medication contributes to a 'safe culture' in the hospital: The pharmacist participates in the medical round at the nursing department. *Pharmaceutisch Weekblad*. 2008;**143**(17):30-2.

Excluded on publication type (n=2)

Hanskamp-Sebregts M, Zegers M, Boeijen W, Westert GP, van Gurp PJ, Wollersheim H. Effects of auditing patient safety in hospital care: design of a mixed-method evaluation. BMC Health Serv Res. 2013;13:226.

Barker A, Mengersen K, Morton A. What is the value of hospital mortality indicators, and are there ways to do better? Aust Health Rev. 2012;36(4):374-7.

Excluded on setting (n=2)

Masotti P, Green M, McColl MA. Adverse events in community care: implications for practice, policy and research. *Healthc Q*. 2009;**12**(1):69-76.

Lang A, Edwards N, Fleiszer A. Safety in home care: a broadened perspective of patient safety. *Int J Qual Health Care*. 2008;**20**(2):130-5.

Update search

Excluded on focus (n=2)

Lam JYJ, Barras M, Scott IA, Abdel-Hafez A, Snoswell C, Gordon E, Morris C, Long D, Wang A, Falconer N. Impact evaluation of the modified adverse inpatient medication event (AIME-Frail) model in hospitalised adults. *Res Soc Ad Pharm.* 2025;**21**:687-696.

McLennan C, Sherrington C, Naganathan V, Tilden W, Richards B, McVeigh T, Hallahan A, Nayak V, Jennings M, Hassett L, Haynes A. Supported implementation of tailored multicomponent fall prevention interventions in hospital: a feasibility study. *BMJ Open Qual.* 2025:**14:**e003313.

For peer review only - http://bmjopen.bmj.com/site/about/guidelines.xhtml

For peer review only

List of 'thin' studies

Australia

Abou Elnour A, Hernan AL, Ford D, Clark S, Fuller J, Johnson JK, et al. Surveyors' perceptions of the impact of accreditation on patient safety in general practice. *Med J Aust*. 2014;**201**(3):S56-S9. https://dx.doi.org/10.5694/mja14.00198

Adamson L, Beldham-Collins R, Sykes J, Thwaites D. Evaluating incident learning systems and safety culture in two radiation oncology departments. *J Med Radiat Sci*. 2022;**69**(2):208-17. https://dx.doi.org/10.1002/jmrs.563

Adie K, Fois RA, McLachlan AJ, Chen TF. Medication incident recovery and prevention utilising an Australian community pharmacy incident reporting system: the QUMwatch study. *Eur J Clin Pharmacol*. 2021;**77**(9):1381-95. https://dx.doi.org/10.1007/s00228-020-03075-9

Avery MJ, Cripps AW, Rogers GD. Health boards' governance of quality and risk: quality improvement agenda for the board. *Int J Health Gov*. 2021;**26**(3):292-306. https://dx.doi.org/10.1108/IJHG-01-2021-0006

Braithwaite J, Westbrook MT, Mallock NA, Travaglia JF, Iedema RA. Experiences of health professionals who conducted root cause analyses after undergoing a safety improvement programme. *Qual Saf Health Care*. 2006;**15**(6):393-9. https://dx.doi.org/10.1136/qshc.2005.017525

Braithwaite J, Westbrook MT, Travaglia JF, Iedema R, Mallock NA, Long D, et al. Are health systems changing in support of patient safety? A multi-methods evaluation of education, attitudes and practice. *Int J Health Care Qual Assur*. 2007;**20**(7):585-601. https://dx.doi.org/10.1108/09526860710822725

Braithwaite J, Westbrook M, Travaglia J. Attitudes toward the large-scale implementation of an incident reporting system. *Int J Qual Health Care*. 2008;**20**(3):184-91. https://dx.doi.org/10.1093/intqhc/mzn004

Brand C, Ibrahim J, Bain C, Jones C, King B. Engineering a safe landing: engaging medical practitioners in a systems approach to patient safety. *Intern Med J*. 2007;**37**(5):295-302. https://dx.doi.org/10.1111/j.1445-5994.2007.01310.x

Canaway R, Bismark M, Dunt D, Kelaher M. Medical directors' perspectives on strengthening hospital quality and safety. *J Health Organ Manag*. 2017;**31**(7-8):696-712. https://dx.doi.org/10.1108/JHOM-05-2017-0109

Canaway R, Bismark M, Dunt D, Kelaher M. Public reporting of hospital performance data: views of senior medical directors in Victoria, Australia. *Aust Health Rev*. 2018;**42**(5):591-9. https://dx.doi.org/10.1071/AH17120

For peer review only - http://bmjopen.bmj.com/site/about/guidelines.xhtml

Clay-Williams R, Taylor N, Ting HP, Arnolda G, Winata T, Braithwaite J. Do quality management systems influence clinical safety culture and leadership? A study in 32 Australian hospitals. *Int J Qual Health Care*. 2020;**32**(Supplement_1):60-6. https://dx.doi.org/10.1093/intqhc/mzz107

Clay-Williams R, Taylor N, Ting HP, Winata T, Arnolda G, Austin E, et al. The relationships between quality management systems, safety culture and leadership and patient outcomes in Australian Emergency Departments. *Int J Qual Health Care*. 2020;**32**(Supplement_1):43-51. https://dx.doi.org/10.1093/intqhc/mzz105

Graudins LV, Dooley MJ. Medication safety: Experiential learning for pharmacy students and staff in a hospital setting. *Pharmacy*. 2016;**4**(4):17. https://dx.doi.org/10.3390/pharmacy4040038

Greenfield D, Hinchcliff R, Banks M, Mumford V, Hogden A, Debono D, et al. Analysing 'big picture' policy reform mechanisms: the Australian health service safety and quality accreditation scheme. *Health Expect*. 2015;**18**(6):3110-22. https://dx.doi.org/10.1111/hex.12300

Hanley E, Quoyle C. Approaches to surveillance of staphylococcus aureus bacteraemia and clostridium difficile infection in Australian states and territories. *Healthc Infect*. 2014;**19**(4):141-6. https://dx.doi.org/10.1071/HI14019

Hughes CF, Mackay P. Sea change: Public reporting and the safety and quality of the Australian health care system. *Med J Aust*. 2006;**184**(10S):S44-S7. https://dx.doi.org/10.5694/j.1326-5377.2006.tb00361.x

Johnson A, Clay-Williams R, Lane P. Framework for better care: reconciling approaches to patient safety and quality. *Aust Health Rev*. 2019;**43**(6):653-5. https://dx.doi.org/10.1071/AH18050

Jones D. Improving clinical leadership in patient safety: from strategic principle to active participation -- the Western Australian approach. *Int J Clin Leadersh*. 2008;**16**(3):157-62.

Leggat SG, Balding C. Effective quality systems: implementation in Australian public hospitals. *Int J Health Care Qual Assur*. 2018;**31**(8):1044-57. https://dx.doi.org/10.1108/IJHCQA-02-2017-0037

Lucas J, Leggat SG, Taylor NF. Association between use of clinical governance systems at the frontline and patient safety: a pre-post study. *Int J Health Gov*. 2022;**27**(3):282-95. https://dx.doi.org/10.1108/IJHG-02-2022-0023

Ong N, Lucien A, Long J, Weise J, Burgess A, Walton M. What do parents think about the quality and safety of care provided by hospitals to children and young people with an intellectual disability? A qualitative study using thematic analysis. *Health Expect*. 2023;**27**(1):e13925. https://dx.doi.org/10.1111/hex.13925

For peer review only - http://bmjopen.bmj.com/site/about/guidelines.xhtml

Pain C, Green M, Duff C, Hyland D, Pantle A, Fitzpatrick K, et al. Between the flags: implementing a safety-net system at scale to recognise and manage deteriorating patients in the New South Wales Public Health System. *Int J Qual Health Care*. 2017;**29**(1):130-6. https://dx.doi.org/10.1093/intqhc/mzw132

Rubin GL, Leeder SR. Health care safety: what needs to be done? *Med J Aust*. 2005;**183**(10):529-31. https://dx.doi.org/10.5694/j.1326-5377.2005.tb07155.x

Runciman WB. Lessons from the Australian Patient Safety Foundation: setting up a national patient safety surveillance system--is this the right model? *Qual Saf Health Care*. 2002;**11**(3):246-51. https://dx.doi.org/10.1136/qhc.11.3.246

Sinni SV, Wallace EM, Cross WM. Perinatal staff perceptions of safety and quality in their service. *BMC Health Serv Res*. 2014;**14**:591. https://dx.doi.org/10.1186/s12913-014-0591-4

Spigelman AD, Swan J. Review of the Australian incident monitoring system. *ANZ J Surg*. 2005;**75**(8):657-61. https://dx.doi.org/10.1111/j.1445-2197.2005.03482.x

Spigelman AD, Rendalls S. Clinical governance in Australia. *Clin Gov*. 2015;**20**(2):56-73. https://doi.org/10.1108/CGIJ-03-2015-0008

Stark HE, Graudins LV, McGuire TM, Lee CYY, Duguid MJ. Implementing a sustainable medication reconciliation process in Australian hospitals: The World Health Organization High 5s project. *Res Social Adm Pharm*. 2020;**16**(3):290-8. https://doi.org/10.1016/j.sapharm.2019.05.011

Stride P, Seleem M, Nath N, Horne A, Kapitsalas C. Integration of patient safety systems in a suburban hospital. *Aust Health Rev*. 2012;**36**(4):359-62. https://doi.org/10.1071/AH11099

Taitz J, Genn K, Brooks V, Ross D, Ryan K, Shumack B, et al. System-wide learning from root cause analysis: a report from the New South Wales Root Cause Analysis Review Committee. *Qual Saf Health Care*. 2010;**19**(6):e63. https://doi.org/10.1136/qshc.2008.032144

Taylor KA, Durrheim DN, Merritt T, Massey P, Ferguson J, Ryan N, et al. Multidisciplinary analysis of invasive meningococcal disease as a framework for continuous quality and safety improvement in regional Australia. *BMJ Open Qual*. 2018;**7**(1):e000077. https://doi.org/10.1136/bmjoq-2017-000077

Travaglia JF, Westbrook MT, Braithwaite J. Implementation of a patient safety incident management system as viewed by doctors, nurses and allied health professionals. *Health*. 2009;**13**(3):277-96. https://doi.org/10.1177/1363459308101804

For peer review only - http://bmjopen.bmj.com/site/about/guidelines.xhtml

Westbrook MT, Braithwaite J, Travaglia JF, Long D, Jorm C, Iedema RA. Promoting safety: longer-term responses of three health professional groups to a safety improvement programme. Int *J Health Care Qual Assur*. 2007;**20**(7):555-71. https://doi.org/10.1108/09526860710822707

Australia & Canada

Arah OA, Klazinga NS. How safe is the safety paradigm? *Qual Saf Health Care*. 2004;**13**(3):226-32. https://doi.org/10.1136/qshc.2003.007070

Cheng L, Sun N, Li Y, Zhang Z, Wang L, Zhou J, et al. International comparative analyses of incidents reporting systems for healthcare risk management. *J Evid Based Med*. 2011;**4**(1):32-47. https://doi.org/10.1111/j.1756-5391.2011.01119.x

Australia & New Zealand

Adamson L, Beldham-Collins R, Sykes J, Thwaites D. Safety culture and incident learning systems in radiation oncology: Staff perceptions across Australia and New Zealand. *J Med Imaging Radiat Oncol*. 2022;**66**(2):299-309. https://doi.org/10.1111/1754-9485.13335

Harrison R, Lee H, Sharma A. A survey of the impact of patient adverse events and near misses on anaesthetists in Australia and New Zealand. *Anaesth Intensive Care*. 2018;**46**(5):510-5. https://doi.org/10.1177/0310057X1804600513

Canada

Baker RG. Governance, policy and system-level efforts to support safer healthcare. *Healthc Q*. 2014;**17 Spec no**:21-6. https://doi.org/10.12927/hcq.2014.23955

Beard P, Smyrski L. Reporting for learning and improvement: the Manitoba and Saskatchewan experience. *Healthc Q*. 2006;**9**:61-4. https://doi.org/10.12927/hcq..18461

Beesoon S, Robert J, White J. Surgery Strategic Clinical Network: Improving quality, safety and access to surgical care in Alberta. *CMAJ*. 2019;**191**:S27-S9. https://doi.org/10.1503/cmaj.190590

Chartier LB, Ovens H, Hayes E, Davis B, Calder L, Schull M, et al. Improving quality of care through a mandatory provincial audit program: Ontario's emergency department return visit quality program. *Ann Emerg Med*. 2021;**77**(2):193-202. https://doi.org/10.1016/j.annemergmed.2020.09.449

Cochrane D, Taylor A, Miller G, Hait V, Matsui I, Bharadwaj M, et al. Establishing a provincial patient safety and learning system: pilot project results and lessons learned. *Healthc Q*. 2009;**12**:147-53. https://doi.org/10.12927/hcq.2009.20717

For peer review only - http://bmjopen.bmj.com/site/about/guidelines.xhtml

Cowell J, Harvie M. HQCA: building a credible, transparent, and independent healthcare quality and safety organization in Alberta. *Healthc Manage Forum*. 2012;**25**(4):185-7. https://doi.org/10.1016/j.hcmf.2012.09.011

Espin S, D'Arpino M, Indar A, Gross M. Incident management in health care: A pan-Canadian perspective. *J Nurs Care Qual*. 2022;**37**(1):E15-E21. https://doi.org/10.1097/NCQ.0000000000000572

Hewitt T, Chreim S, Forster A. Sociocultural factors influencing incident reporting among physicians and nurses: understanding frames underlying self- and peer-reporting practices. *J Patient Saf*. 2017;**13**(3):129-37. https://doi.org/10.1097/PTS.0000000000000130

Jeffs L, Baker GR, Taggar R, Hubley P, Richards J, Merkley J, et al. Attributes and actions required to advance quality and safety in hospitals: Insights from nurse executives. *Nurs Leadersh*. 2018;**31**(2):20-31. https://doi.org/10.12927/cjnl.2018.25606

Jeffs L, Bruno F, Zeng RL, Schonewille N, Kinder K, De Souza G, et al. Integrating implementation science in a quality and patient safety improvement learning collaborative: Essential ingredients and impact. *Jt Comm J Qual Patient Saf*. 2023;**49**(5):255-64. https://doi.org/10.1016/j.jcjq.2023.02.001

Kassam A, Sharma N, Harvie M, O'Beirne M, Topps M. Patient safety principles in family medicine residency accreditation standards and curriculum objectives: Implications for primary care. *Can Fam Physician*. 2016;**62**(12):e731-e9.

Law MP, Zimmerman R, Baker GR, Smith T. Assessment of safety culture maturity in a hospital setting. *Healthc Q*. 2010;**13**:110-5. https://doi.org/10.12927/hcq.2010.21975

Milligan C, Allin S, Farr M, Farmanova E, Peckham A, Byrd J, et al. Mandatory reporting legislation in Canada: improving systems for patient safety? *Health Econ Policy Law*. 2021;**16**(3):355-70. https://doi.org/10.1017/S1744133121000050

Milne JK, Lalonde AB. Patient safety in women's health-care: professional colleges can make a difference. The Society of Obstetricians and Gynaecologists of Canada MORE(OB) program. *Best Pract Res Clin Obstet Gynaecol*. 2007;**21**(4):565-79. https://doi.org/10.1016/j.bpobgyn.2007.01.013

Mitchell JI, Izad Shenas SA, Kuziemsky C. Governance standards: A roadmap for increasing safety at care transitions. *Healthc Manage Forum*. 2015;**28**(1):28-33. https://doi.org/10.1177/0840470414551894

Nicklin W, Mass H, Affonso DD, O'Connor P, Ferguson-Paré M, Jeffs L, et al. Patient safety culture and leadership within Canada's Academic Health Science Centres: towards the development of a collaborative position paper. Nurs Leadersh. 2004;**17**(1):22-34. https://doi.org/10.12927/cjnl.2004.16243

For peer review only - http://bmjopen.bmj.com/site/about/guidelines.xhtml

O'Beirne M, Reid R, Zwicker K, Sterling P, Sokol E, Flemons W, et al. The costs of developing, implementing, and operating a safety learning system in community practice. *J Patient Saf*. 2013;**9**(4):211-8. https://doi.org/10.1097/PTS.0000000000000052

O'Beirne M, Sterling PD. Medical safety and community practice: necessary elements and barriers to implement a safety learning system. *Healthc Q*. 2009;**12**:141-6. https://doi.org/10.12927/hcq.2009.20982

O'Beirne M, Sterling PD, Zwicker K, Hebert P, Norton PG. Safety incidents in family medicine. *BMJ Qual Saf*. 2011;**20**(12):1005-10. 10.1136/bmjqs-2011-000105

Skutezky T, Small SS, Peddie D, Balka E, Hohl CM. Beliefs and perceptions of patient safety event reporting in a Canadian Emergency Department: a qualitative study. *CJEM*. 2022;**24**(8):867-75. https://doi.org/10.1007/s43678-022-00400-2

Weaver RR. Seeking high reliability in primary care: Leadership, tools, and organization. *Health Care Manage Rev*. 2015;**40**(3):183-92. https://doi.org/10.1097/HMR.0000000000000022

Williams A. Nursing informaticians address patient safety to improve usability of health information technologies. *Stud Health Technol Inform*. 2019;**257**:501-7.

Zecevic AA, Ho-Ting Li A, Ngo C, Halligan M, Kothari A, Li AH-T. Improving safety culture in hospitals: Facilitators and barriers to implementation of Systemic Falls Investigative Method (SFIM). *Int J Qual Health Care*. 2017;**29**(3):371-7. https://doi.org/10.1093/intqhc/mzx034

Update search:

Kuluski K, Asselbergs M, Baker R, Burns KKK, Bruno F, Saragosa M, MacLaurin A, Flintoft V, Jeffs L. 'Safety is about partnership': Safety through the lens of patients and caregivers. *Health Expect*. 2024;**27**:e13939.

Recsky C, Rush KL, MacPhee M, Stowe M, Blackburn L, Muniak A, Currie LM. Clinical informatics team members' perspectives on health information technology safety after experiential learning and safety process development: Qualitative descriptive study. *JMIR Formative Research*. 2024;**5:**e53302.

Ireland

Flynn MA, Burgess T, Crowley P. Supporting and activating clinical governance development in Ireland: sharing our learning. *J Health Organ Manag*. 2015;**29**(4):455-81. https://doi.org/10.1108/JHOM-03-2014-0046

For peer review only - http://bmjopen.bmj.com/site/about/guidelines.xhtml

O'Connor P, O'Malley R, Kaud Y, St Pierre E, Dunne R, Byrne D, et al. A scoping review of patient safety research carried out in the Republic of Ireland. *Irish J Med Sci*. 2023;**192**(1):1-9. https://doi.org/10.1007/s11845-022-02930-1

Netherlands

Bilijam CW. Patient safety in general practice. *Tijdschrift voor Kindergeneeskunde*. 2014;**82**(5):155-9.

Cords CI, van der Vlies CH, Stoop M, Nieuwenhuis MK, Boudestein K, Mattace-Raso FUS, et al. Frailty screening practice in specialized burn care-a retrospective multicentre cohort study. *Eur Burn J*. 2023;**4**(1):87-100. https://doi.org/10.3390/ebj4010009

de Korne DF, Van Wijngaarden JDH, Van Dyck C, Hiddema UF, Klazinga NS. Evaluation of aviation-based safety team training in a hospital in The Netherlands. *J Health Organ Manag*. 2014;**28**(6):731-53. https://doi.org/10.1108/jhom-01-2013-0008

Harmsen M, Gaal S, van Dulmen S, de Feijter E, Giesen P, Jacobs A, et al. Patient safety in Dutch primary care: a study protocol. *Implement Sci*. 2010;**5**:50. https://doi.org/10.1186/1748-5908-5-50 Heim N, van Fenema EM, Weverling-Rijnsburger AW, Tuijl JP, Jue P, Oleksik AM, et al. Optimal screening for increased risk for adverse outcomes in hospitalised older adults. *Age Ageing*. 2015;**44**(2):239-44. https://doi.org/10.1093/ageing/afu187

Hermans MPJ, Eindhoven DC, van Winden LAM, de Grooth GJ, Blauw GJ, Muller M, et al. Frailty score for elderly patients is associated with short-term clinical outcomes in patients with ST-segment elevated myocardial infarction treated with primary percutaneous coronary intervention. *Neth Heart J*. 2019;**27**(3):127-33. https://doi.org/10.1007/s12471-019-1240-7

Jepma P, Verweij L, Tijssen A, Heymans MW, Flierman I, Latour CHM, et al. The performance of the Dutch Safety Management System frailty tool to predict the risk of readmission or mortality in older hospitalised cardiac patients. *BMC Geriatr*. 2021;**21**(1):299. https://doi.org/10.1186/s12877-021-02243-5

Jessurun JG, Hunfeld NGM, Van Rosmalen J, Van Dijk M, Van Den Bemt P. Effect of automated unit dose dispensing with barcode scanning on medication administration errors: an uncontrolled before-and-after study. *Int J Qual Health Care*. 2021;**33**(4):13. https://doi.org/10.1093/intqhc/mzab142

Oud FMM, Schut MC, Spies PE, van der Zaag-Loonen HJ, de Rooij SE, Abu-Hanna A, et al. Interaction between geriatric syndromes in predicting three months mortality risk. *Arch Gerontol Geriatr*. 2022;**103:**104774. https://doi.org/10.1016/j.archger.2022.104774

Oud FMM, Wolzak NK, Spies PE, Zaag-Loonen HJV, van Munster BC. The predictive value of the 'VMS frail older patients' for adverse outcomes in geriatric inpatients. *Arch Gerontol Geriatr*. 2021;**97**:104514. https://doi.org/10.1016/j.archger.2021.104514

For peer review only - http://bmjopen.bmj.com/site/about/guidelines.xhtml

Schuijt HJ, Oud FMM, Bruns EJR, van Duijvendijk P, Van der Zaag-Loonen HJ, Spies PE, et al. Does the Dutch Safety Management Program predict adverse outcomes for older patients in the emergency department? *Neth J Med*. 2020;**78**(5):244-50.

Snijders BMG, Emmelot-Vonk MH, Souwer ETD, Kaasjager HAH, van den Bos F. Prognostic value of screening instrument based on the Dutch national VMS guidelines for older patients in the emergency department. *Eur Geriatr Med*. 2021;**12**(1):143-50. https://doi.org/10.1007/s41999-020-00385-0

Snijders C. Patient safety in the neonatal intensive care unit: A look back on the NEOSAFE study. *Tijdschrift voor Kindergeneeskunde*. 2014;**82**(5):160-5.

van Dam CS, Trappenburg MC, Ter Wee MM, Hoogendijk EO, de Vet HC, Smulders YM, et al. The accuracy of four frequently used frailty instruments for the prediction of adverse health outcomes among older adults at two Dutch emergency departments: Findings of the AmsterGEM study. *Ann Emerg Med*. 2021;**78**(4):538-48. https://doi.org/10.1016/j.annemergmed.2021.04.027

van der Starre, C., van Dijk, M. & Tibboel, D. Real-time registration of adverse events in Dutch hospitalized children in general pediatric units: first experiences. *Eur J Pediatr.* 2012;**171**:553–558. https://doi.org/10.1007/s00431-011-1608-1

van der Zanden V, Paarlberg KM, van der Zaag-Loonen HJ, Meijer WJ, Mourits MJE, van Munster BC. Risk assessment for postoperative outcomes in a mixed hospitalized gynecological population by the Dutch safety management system (Veiligheidsmanagementsysteem, VMS) screening tool 'frail elderly'. *Arch Gynecol Obstet*. 2021;**304**(2):465-73. https://doi.org/10.1007/s00404-021-06073-z

van Munster BC, Drost D, Kalf A, Vogtlander NP. Discriminative value of frailty screening instruments in end-stage renal disease. *Clin Kidney J*. 2016;**9**(4):606-10. https://doi.org/10.1093/ckj/sfw061

van Schoten SM, Baines RJ, Spreeuwenberg P, de Bruijne MC, Groenewegen PP, Groeneweg J, et al. The ecometric properties of a measurement instrument for prospective risk analysis in hospital departments. *BMC Health Serv Res*. 2014;**14**:103. https://doi.org/10.1186/1472-6963-14-103

Verstappen W, Gaal S, Bowie P, Parker D, Lainer M, Valderas JM, et al. A research agenda on patient safety in primary care. Recommendations by the LINNEAUS collaboration on patient safety in primary care. *Eur J Gen Pract*. 2015;**21**(sup1):72-7. https://doi.org/10.3109/13814788.2015.1043726

Warnier RMJ, van Rossum E, van Kuijk SMJ, Magdelijns F, Schols J, Kempen G. Frailty screening in hospitalised older adults: How does the brief Dutch National Safety Management Program perform compared to a more extensive approach? *J Clin Nurs*. 2020;**29**(7-8):1064-73. https://doi.org/10.1111/jocn.15148

For peer review only - http://bmjopen.bmj.com/site/about/guidelines.xhtml

Weske U, Boselie P, van Rensen E, Schneider M. Physician compliance with quality and patient safety regulations: The role of perceived enforcement approaches and commitment. *Health Serv Manage Res*. 2019;**32**(2):103-12. https://doi.org/10.1177/0951484818813324

Weske U, Boselie P, van Rensen ELJ, Schneider MME. Using regulatory enforcement theory to explain compliance with quality and patient safety regulations: the case of internal audits. *BMC Health Serv Res*. 2018;**18**(1):62. https://doi.org/10.1186/s12913-018-2865-8

Update search:

Oud FMM, Meulman MD, Merten H, Wagner C, van Munster BC. Value of the Safety Management System (VMS) frailty instrument as a frailty screener in care for older hospital patients: a systematic review. *Eur Geriat Med*. 2024;**15**:609-620.

New Zealand

Raymont A, Graham P, Hider PN, Finlayson MP, Fraser J, Cumming JM. Variation in the adoption of patient safety practices among New Zealand district health boards. *Aust Health Rev*. 2012;**36**(2):163-8. https://doi.org/10.1071/AH10972

Robin G, Horsburgh S. Healthcare professional perspectives on quality and safety in New Zealand public hospitals: findings from a national survey. *Aust Health Rev*. 2014;**38**(1):109-14. https://doi.org/10.1071/AH13116

Shuker C, Bohm G, Bramley D, Frost S, Galler D, Hamblin R, et al. The Health Quality and Safety Commission: making good health care better. *N Z Med J*. 2015;**128**(1408):97-109.

Mobius Research & Strategy. The Health Quality & Safety Commission Surgical Safety Culture Survey Research Report. 2017. URL: https://www.hqsc.govt.nz/assets/Our-work/Improved-service-delivery/Safe-surgery/Publications-resources/2017_Surgical_Safety_Culture_Survey_Report_-_FINAL.pdf (accessed 24 April 2024).

For peer review only - http://bmjopen.bmj.com/site/about/guidelines.xhtml

Supplementary material 4: Rich and descriptive studies

Documents relating to patient safety and/or health services in each country included in the review

Document	Year of publication	Description of document
**Netherlands**		
Policy		
NTA 8009 'Safety management system for hospitals and institutions that provide hospital care' (initial technical agreement).[29*]	2007	Describes the basic requirements for an SMS for hospitals and institutions that provide hospital care. It focuses on managing risks and reducing harm to patients. It is based on the Rein Willems report '*Here you work safely or you don't work here'* (2004) and the improvement cycle 'plan-do-check-act' on which other SMS are based. The structure of the document includes: 1. Subject and scope, 2. Terms and definitions, 3. Responsibilities of the Board of Directors, and 4. Basic requirements for the SMS. Additional information is provided in the appendices on the organisations involved in the drafting of the document; the reporting matrix (roles and responsibilities for reporting different types of incidents such as near misses and serious incidents); conversion tables that explain how the NTA relates to other standards (e.g. ISO 9001: 2000 Quality Management System standard); examples of prospective and retrospective risk assessment; and safety culture (which is not explicitly included in the basic requirements).
NEN 8009 'Safety management system for hospitals and institutions that provide hospital care' (replaces NTA 8009 2014).[30*]	2018	This is the third update of the original NTA 8009 (following updates in 2011 and 2014). It supplements, without replacing, the original SMS requirements with Safety-II concepts that promote a proactive safety culture and encourage learning from daily activities with desired outcomes as well as incidents. It acknowledges that improvement could vary from continuous improvements in small steps to significant changes at organisational level and highlights the importance of encouraging employees and patients to play an active role, identify opportunities and successful practices and contribute to the overall process of organisational learning.

For peer review only - http://bmjopen.bmj.com/site/about/guidelines.xhtml

Other				
Bal R, Wagner C. The Netherlands: Patient safety in Dutch hospitals: How can we explain success, in Health systems improvement across the Globe: Success stories from 60 countries, eds. Braithwaite J et al. Boca Raton: CRC Press.[31]	2018	Provides an overview of the Dutch patient safety programme and tries to explain its success, accounting for the broader socio-cultural and historical context, and identifying the ‘conditions under which such a programme was able to succeed’ (p. 208). It argues that five such factors contributed to the success of the Dutch patient safety programme: experience in project work, market competition, public pressure, activities of regulatory agencies, and public reporting of adverse events and mortality.		
Bolk M. Final report: Embedding patient safety in education and training. Utrecht: VMS Safety Programme.[32]	2013	Reports on the ‘*Embedding patient safety in education and training*’ project which aimed ‘to support, facilitate and accelerate education for doctors, nurses and the supporting professions (medical and otherwise), with the help of a national incentive program and the corresponding support structure’ (p. 4). As a result, a national ‘patient safety competencies framework’ was developed; all findings from the project were compiled in a digital portal and campaign website and a ‘patient safety toolkit’ was developed; examples for good patient safety education were collected; and the attention generated regarding patient safety resulted in the project linking to other educational initiatives already working on patient safety. The project made a number of recommendations for embedding patient safety in various educational and learning processes and programmes.		
Research		**Aims**	**Methods**	**Findings**
Baines R et al. How effective are patient safety initiatives? A retrospective patient record review study of changes to patient safety over time. *BMJ Qual Saf*. 2015;**24**(9):561-71.[33]	2015	To assess whether hospital care in 2011/12 was safer than in 2004 and 2008.	Retrospective patient record review. 15, 997 patient admissions were included, 7926 from 2004, 4023 from 2008 and 4048 from 2011/2012.	In comparison with 2008, uncorrected crude overall adverse event rates showed no change in 2011/2012 but preventable adverse event rates showed a reduction of 45%. After multilevel corrections, the

For peer review only - http://bmjopen.bmj.com/site/about/guidelines.xhtml

				decrease was still visible but not statistically significant.
de Blok C et al. Implementation of the Dutch National Patient Safety Programme (VMS Veiligheidsprogrammesma). Utrecht/Amsterdam: NIVEL/EMGO+.[34]	2013	English summary of de Blok[21] (see below).		
de Blok C, Koster E, Schilp J, Wagner C. Implementatie VMS Veiligheidsprogrammesma. Evaluatieonderzoek in Nederlandse ziekenhuizen. Utrecht / Amsterdam: NIVEL/EMGO+.[21]*	2013	To assess the extent to which the ten themes of the Dutch patient safety programme have been implemented and gain insight into the factors that influence the degree of implementation and compliance with the advice.	Data collection varied across themes and included observations and semi-structured interviews, review of patient records and registry data. A large proportion of Dutch hospitals were involved in the evaluation, which varied across themes.	Implementation objectives set in 2008 were almost achieved for some of the themes (e.g. 'Preventing renal insufficiency during intravascular use of iodinated contrast agent' and subtheme 'Treatment of severe sepsis'). There was a positive trend for other themes, but considerable variation was observed across and within hospitals. The study identified a range of organisational, staff- and theme-related factors associated with the success of implementation.
de Bruijne MC, Zegers M, Hoonhout LHF, Wagner C. Onbedoelde schade in Nederlandse ziekenhuizen: Dossieronderzoek van ziekenhuisopnames in 2004. Amsterdam: EMGO+ Instituut, Utrech: NIVEL[35]*	2007	To establish a baseline measurement in the field of patient safety.	Retrospective patient record review; 200 patient records from patients who were discharged and 200 from patients who died in hospitals in 2004 were randomly selected from each of the participating 21	Of all admissions: (i) 5.7% suffered unintentional healthcare-related harm, 5% of which experienced permanent harm and 8% died partly as a result of this; (ii) 2.3% suffered preventable adverse events. Of the patients who died in hospital,

For peer review only - http://bmjopen.bmj.com/site/about/guidelines.xhtml

			Dutch hospitals. Patients admitted to psychiatry and obstetrics and those less then one year of age were excluded as the assessment system was not suitable for such patients.	10.7% suffered unintentional healthcare-related events; of those, 4.1% were related to preventable adverse events during admission. It was estimated that in 2004, there were 1735 (95%CI 1482 to 2032) potentially preventable deaths in Dutch hospitals.
de Groot JF et al. Implementing paediatric early warning scores systems in the Netherlands: future implications. *BMC Pediatr*. 2018;**18**(1):128.[36]	2018	(i) To evaluate the implementation of paediatric early warning score systems in five hospitals.<br>(ii) To gain insight into the attitudes of professionals towards the system.	(i) Retrospective patient record review (n = 554) at the start, mid and endpoint of implementation.<br>(ii) Semi-structured interviews with professionals (n = 8).	(i) None of the hospitals started with a system but after 1 year, they were recorded in 69.2% of patient records and resulted in appropriate action in 49.1%.<br>(ii) Sustainable implementation might be hindered by staff concerns about validity, effectiveness and communication with other hospitals during transfers to higher levels of care.
Klopotowska J, Schutijser B, de Bruijne M, Wagner C. Tweede evaluatie van VMS Programma in Nederlandse ziekenhuizen op basis van vier veiligheidsthema's: aangevuld met EPD Scan naar mogelijkheden voor toekomstige metingen. Utrecht/Amsterdam: NIVEL/EMGO+.[37*]	2016	To investigate the progress of the implementation of four safety themes from the Dutch national patient safety programme compared to the first evaluation study in 2011/2012 and assess the possibility of automatic monitoring of process indicators from	Data collection:<br>(i) Theme 'medication verification': review of patient records (n=1060) in 15 hospitals.<br>(ii) Theme 'high risk medication': observations of nurses (n=349) in 15 hospitals.	There was improvement in compliance for (i) and (ii) but still the target of 100% was not met; authors note that 100% compliance may not be desirable as in some cases deviation from guidelines could be beneficial for the patient. For (iii) compliance increased by 34% (to 73% against 90% target). For (iv) hospital mortality among severe sepsis patients

For peer review only - http://bmjopen.bmj.com/site/about/guidelines.xhtml

		electronic patient records for two of the themes.	Themes (iii) ‘prevention of wound infections after surgery’ and (iv) ‘prevention of line sepsis and treatment of severe sepsis’: data from national registries.	has fallen from 34% to 29% (15% relative mortality reduction) but for line sepsis positive results were observed only for the ICU departments on which the hospitals focused initially (incidence was 2.5 times higher in non-ICU).
Langelaan M et al. Monitor zorggerelateerde schade 2008: Dossieronderzoek in Nederlandse ziekenhuizen. Utrecht: NIVEL, Amsterdam: EMGO+ Instituut.[38]*	2010	To estimate and characterise potentially avoidable adverse events and deaths, and those related to the use of medical technology; to compare them to 2004.	Retrospective patient record review: stratified samples of 100 admissions of discharged patients and 100 of patients who died in hospital were drawn from each of the 20 participating hospitals. Psychiatry, obstetrics and patients < 1 year of age were excluded.	2.9% potentially avoidable adverse events (of all admissions). The change from 2004 was not statistically significant; 0.8% was related to the use of medical technology. Potentially avoidable mortality was 5.5% (95%CI 4.5% to 6.6%); the change from 2004 was not statistically significant. In absolute numbers, in 2008 there were 1960 (95%CI 1600 to 2460) patients in whom potentially avoidable harm contributed to the death.
Langelaan M et al. Monitor Zorggerelateerde Schade 2011/2012. Dossieronderzoek in Nederlandse ziekenhuizen. Amsterdam: VU medisch centrum & EMGO+ Instituut, Utrecht: NIVEL.[39]*	2013	To estimate and characterise potentially avoidable adverse events and mortality in Dutch hospitals, including those related to medical technology, and compare them to previous measurements.	Retrospective patient record review study based on data from 20 randomly selected Dutch hospitals (>4000 records in total); random samples were drawn from each hospital of 100 discharged patients and 100 patients who died in the hospital. Psychiatry,	Of all patients admitted in 2011/2012, 1.6% (95% CI: 1.1% to 2.2%) experienced potentially avoidable harm: 30% and 45% less than in 2004 and 2008, respectively. Potentially avoidable mortality decreased by 37% and 53% compared to 2004 and 2008, respectively, which expressed in absolute

For peer review only - http://bmjopen.bmj.com/site/about/guidelines.xhtml

		To estimate the costs of healthcare-related harm and the quality of file management.	obstetrics and patients < 1 year of age were excluded.	numbers and extrapolated to all Dutch hospitals was approx. 1,735 patients (95% CI: 1,482 to 2,032) in 2004, 1,960 patients (95% CI: 1,600 to 2,360) in 2008, and 970 (95% CI: 738 to 1,274) patients between 1st April 2011 and 31st March 2012. Other findings included harms related to the application of medical technology, costs related to adverse events and the quality of patient records.
Langelaan M et al. Monitor Zorggerelateerde Schade 2015/2016: Dossieronderzoek bij overleden patiënten in Nederlandse ziekenhuizen. Utrecht: NIVEL, Amsterdam: EMGO+ Instituut.[40*]	2017	To estimate and characterise potentially avoidable adverse events and mortality in Dutch hospitals, including those related to medical technology, and compare them to previous measurements.	Retrospective patient record review: a random sample of 150 patients who died in hospital was drawn from each of the 19 hospitals, randomly selected to participate in the study. Psychiatry, obstetrics and patients < 1 year of age were excluded.	Of all deceased patients, 4.3% (95%CI 3.6% to 5.1%) suffered potentially preventable adverse events and 3.1% (95%CI 2.5% to 3.8%) suffered potentially preventable mortality. The difference with 2011/12 was not statistically significant.<br>The respective results related to the use of medical technology were 1.7% and 1.3%, out of all cases in which technology was used.
Meulman M, Merten H, Wagner C. Het screenen van kwetsbare ouderen: onderzoek vanuit een Safety-I en Safety-II perspectief: evaluatie van het VMS thema	2021	To investigate the degree of compliance with the national Dutch patient safety programme screening	Functional Resonance Analysis Method was used, based on analysis of hospital protocols, interviews with nurses (n=31), and a feedback meeting. Additionally, a	Hospital protocols were more detailed than the national guidelines but largely similar. There were actions in the protocols that were not performed, and actions that were performed but not included

For peer review only - http://bmjopen.bmj.com/site/about/guidelines.xhtml

Kwetsbare ouderen. Utrecht: NIVEL[41]*		tool for vulnerable older people.	survey of employees' resilience (n=146), an evaluation interview in each department, and compliance data (the percentage of admitted patients aged ≥70 who were screened). Data were collected between 2020-2021, from 10 departments in 6 hospitals.	in the protocols. An average of 74% of admitted patients were screened. Individual resilience (66%) was rated more positively than team resilience (49%).
Meurs M, de Groot JF. Veiligheidsmanagement systeem (VMS) thema's kindzorg Pijn en (Lijn)sepsis: verbeterpunten vanuit het werkveld voor verdere kwaliteitsverbetering. Utrecht: NIVEL. [42]*	2020	To identify bottlenecks and areas of improvement for implementation of the Dutch national patient safety programme themes '(line) sepsis' and 'pain' within paediatric care hospitals.	Working groups with paediatricians and paediatric nurses, and a survey (n=209). Data was collected in 2019.	Theme '(line) sepsis': the main recommendation regarded earlier recognition and treatment of sepsis (e.g. through training).<br>Theme 'pain': the main recommendation for improvement was to revise guidelines to adopt a holistic approach to pain recognition and treatment.
Sambeeck SJV et al. Pediatric Early Warning System Scores: Lessons to be learned. *J Pediatr Intensive* Care. 2018;**7**(1):27-32.[43]	2018	To investigate how many hospitals complied with the national recommendation to implement a paediatric early warning system score and if so, the score they chose to implement.	Cross-sectional electronic survey of Dutch hospitals in 2014 (n=91).	Three-quarters of Dutch hospitals with a paediatric department implemented a score. These hospitals used 45 different versions of a paediatric early warning system score, the majority of which were not validated.

For peer review only - http://bmjopen.bmj.com/site/about/guidelines.xhtml

Schlinkert C, Jelsma J, Wagner C. Patiëntveiligheidscultuur in Nederlandse Ziekenhuizen. Van openheid naar reflectie en veerkracht. Utrecht: NIVEL.[44]*	2021	To measure the patient safety culture in Dutch hospitals in 2020 and compare it with previous measurements (before and after the implementation of the Dutch patient safety programme). To measure the employees' resilience.	Cross-sectional survey on patient safety culture (Dutch version of Hospital Survey on Patient Safety Culture) and employees' resilience. Data were collected in 2020; 3014 employees from 5 hospitals completed the survey. Comparison with data collected using the same survey in 2005-2007 and 2012.	62.5% of respondents rated patient safety in their own department 'very good' or 'excellent'. Teamwork was the highest rated dimension, 'Adequate staffing', 'Support from management' and 'Collaboration between departments' were rated lowest. Nursing staff rated patient safety more negatively than other professional groups. More respondents considered patient safety to be 'very good' or 'excellent' in 2020 compared to previous measurements.
van Dijk LM, van Eikenhorst L, Muns L, Wagner C. Medicatieverificatie bij ontslag: nieuwe benadering vanuit een Safety-I en Safety-II perspectief: derde evaluatie van het VMS-thema. Utrecht: NIVEL.[45]*	2021	To investigate the implementation of the 'medication verification at discharge' theme of the Dutch national patient safety programme.	Functional Resonance Analysis Method was used, based on hospital protocols, interviews with nurses (n=77), and a feedback meeting. A survey of employees' resilience (n=142) and an evaluation interview in each department were carried out, and compliance data were collected (percentage of discharged patients aged ≥18 for whom medication verification took place upon discharge). Data was collected between 2020-	Hospital protocols were more detailed than the national guidelines. There were variations in how the processes were carried out in practice, normally in response to circumstances in the work environment (e.g. IT problems, staff shortages). Compliance rates varied between 67%-100% across departments over the data collection period. Both individual (64%) and team (69%) resilience was rated positively.

For peer review only - http://bmjopen.bmj.com/site/about/guidelines.xhtml

			2021, from 9 departments in 8 hospitals (4 cardiology departments and 5 orthopaedics departments).	
van der Starre C et al. Paediatric critical incident analysis: lessons learnt on analysis, recommendations and implementation. *Eur J Pediatr*. 2014;**173**(11):1449-57.[46]	2014	To identify potential causal and contributing factors of serious paediatric patient safety incidents, and to report on and assess the extent of implementation of recommendations for improvement.	Retrospective review of critical incident analyses (n=17) from 2005–2010.	A median number of 5 causal and contributing factors was identified per incident and were mostly likely to be team or task-related. There was a median of 5 recommendations per analysis, only one-third of recommendations were implemented.
van Noord N, Zwijnenberg I, Wagner C. Patiëntveiligheidscultuur in Nederlandse Ziekenhuizen. Een stap in de goede richting. Utrecht: NIVEL.[47]*	2013	To examine whether the Dutch national patient safety programme improved patient safety culture.	Comparison of two cross-sectional safety culture surveys (Dutch version of Hospital Survey on Patient Safety Culture). Between 2005-2007, 3779 healthcare providers in 45 hospitals responded, in 2012, 6605 healthcare providers in 24 hospitals responded.	Patient safety culture improved between 2005-2007 and 2012, with 48.8% of respondents rating it 'very good' or 'excellent' in 2012. However, there was variation between hospitals and departments. 'Teamwork' was the highest rated dimension of safety; 'Collaboration between departments', 'Reporting frequency' and 'Support from management' were lowest rated. Nursing staff rated patient safety more negatively than other professionals.
van Schoten S et al. The association between quality system development stage and	2018	To examine the association between the development stage	Analysis of a national survey on the development stage of hospital quality	No association was found between the development stage of a hospital quality system and

For peer review only - http://bmjopen.bmj.com/site/about/guidelines.xhtml

the implementation of process-level patient safety themes in Dutch hospitals: an observational study. *BMC Health Serv Res.* 2018;**18**:189.[48]		of hospital quality systems and the process-level implementation of patient safety themes within the Dutch patient safety programme.	systems (conducted in 2011) and an evaluation study of the national Dutch patient safety program (2011-2012). Data from 30 hospitals (12,485 observations) were included.	the process-level implementation of patient safety themes.
van Schoten S et al. Monitor Zorggerelateerde Schade 2019: Dossieronderzoek bij overleden patiënten in Nederlandse ziekenhuizen. Utrecht: NIVEL, Amsterdam: APH.[49*]	2022	To estimate and characterise potentially avoidable adverse events and mortality in Dutch hospitals, including those related to medical technology and diagnostic processes, and those in vulnerable older patients, and compare them to previous measurements.<br><br>To assess the quality of file management.	Retrospective patient record review: a random sample of 150 patients who died in hospital was drawn from each of the 20 hospitals, randomly selected to participate in the study. Psychiatry, obstetrics and patients < 1 year of age were excluded.	14.6% (95%CI 13.3% to 16.0%) of patients who died in hospital suffered care-related harm. This is a significant increase compared to 2015/2016 (was 9.9% (95% CI 8.9% to 11.0%)). Potentially avoidable adverse events and mortality were 4.2% (95%CI 3.5% to 5.0%) and 3.1% (95% CI 2.5% to 3.9%), respectively, indicating that the change from the previous measurement was not statistically significant.
van Stralen S, Schutijser B, Vonk A, van Eikenhorst L, Wagner C. Tweede controle bij het klaarmaken en toedienen van high-risk medicatie: nieuwe benadering vanuit een Safety-I en Safety-II perspectief. Derde evaluatie van het VMS thema. Utrecht: NIVEL. [50*]	2021	To investigate the implementation of the 'high-risk medication' theme (specifically the second check of medication) of the Dutch national patient safety programme.	Functional Resonance Analysis Method was used, based on hospital protocols, interviews with nurses (n=77), and a feedback meeting. Additionally, a survey of employee resilience (n=142) and an evaluation interview in each	Compared to the national guidelines from the patient safety programme, hospital protocols were largely the same. There were variations in how nurses carried out the processes, normally in response to circumstances in the work environment (e.g. time pressures). Individual (average

For peer review only - http://bmjopen.bmj.com/site/about/guidelines.xhtml

			department were carried out, and compliance data collected. Data was collected between 2020-2021, from 10 departments in 9 hospitals (3 surgery departments, 3 internal medicine departments and 4 intensive care units).	score 3.21/9) and team resilience (average score 4.08/9) was rated highly.
Verbeek-van Noord I et al. A nation-wide transition in patient safety culture: a multilevel analysis on two cross-sectional surveys. *Int J Qual Health Care*. 2019;**31**(8):627–632.[51]	2019	To examine whether the Dutch national patient safety programme improved patient safety culture.	Comparison of two cross-sectional safety culture surveys (Dutch version of Hospital Survey on Patient Safety Culture). Between 2005-2007, 3779 healthcare providers in 45 hospitals responded, in 2012, 6605 healthcare providers in 24 hospitals responded.	Patient safety culture improved over the studied period. However, there was variation between hospitals and departments.
**Australia**				
Policy				
Australian Commission on Safety and Quality in Health Care. National model clinical governance framework.[63]	2017	This framework defines clinical governance as 'a set of relationships and responsibilities established by a health service organisation between its state or territory department of health, governing body, executive, workforce, patients, consumers and other stakeholders'.(p.iii) The framework contains all the elements that acute health service organisations need to implement integrated corporate and clinical governance systems, with the aim of ensuring good clinical outcomes through the delivery of safe and quality care. It is based on the National Safety and Quality Health Service Standards (NSQHS) Clinical Governance and Partnering with Consumers (see below) and contains five components: governance, leadership and culture, patient safety and quality		

For peer review only - http://bmjopen.bmj.com/site/about/guidelines.xhtml

		improvement systems, clinical performance and effectiveness, safe environment for the delivery of care, and partnering with consumers.		
Australian Commission on Safety and Quality in Health Care. National safety and quality health service standards. 2nd edition.[64]	2021	Describe standards intended for organisation-wide implementation in all hospital and day procedure services to enable the delivery of nationally consistent, safe and quality care. There are eight Standards - Clinical Governance, Partnering with Consumers, Preventing and Controlling Healthcare-Associated Infection, Medication Safety, Comprehensive Care, Communicating for Safety, Blood Management and Recognising and Responding to Acute Deterioration. The Clinical Governance and Partnering with Consumers Standards set out overarching requirements for the implementation of the other six Standards. The Standards do not specify how they should be implemented to allow for local adaptation.		
Australian Commission on Safety and Quality in Health Care. National safety and quality primary and community healthcare standards.[65]	2021	Describes a framework which is intended for use in all primary and community healthcare services. The processes and structures in the Standards are intended to enable the delivery of, and improve, the quality and safety of services. There are three Standards - Clinical Governance, Partnering with Consumers, and Clinical Safety – with the former two Standards setting out overarching requirements for the implementation of the Clinical Safety Standards.		
Research		**Aims**	**Methods**	**Findings**
Australian Commission on Safety and Quality in Health Care. Creating safer, better healthcare - the impact of national safety and quality health service standards.[66]	2018	To provide an overview of changes associated with implementation of the first edition of the NSQHS Standards, identifying areas where improvement has been made and where further work is needed.	Draws on a numbers of research projects, including surveys of hospital staff, hospital board members, and specialist medical colleges, National Inpatient Medication Chart Audits, and a Consumer Participation Study involving interviews and focus groups.	Implementation of the NSQHS Standards has resulted in the prevention of harm, improvements in patient care including empowerment of consumers and patients, development of better governance systems, and reduction of waste.

For peer review only - http://bmjopen.bmj.com/site/about/guidelines.xhtml

Australian Commission on Safety and Quality in Health Care. The state of patient safety and quality in Australian hospitals 2019.[67]	2019	To report on safety and quality in public hospitals in Australia, including common patient safety risks, action taken to deliver appropriate care, and the Commission's approach to supporting value-based healthcare.	Draws on data on public hospitals from a wide range of sources, such as the Admitted Patient Care National Minimum Data Set, 2013–14 to 2017–18 and the Australian Atlas of Healthcare Variation.	There have been significant improvements in the safety and quality of care over the last 10 years. These include improved patient outcomes such as reductions in hospital acquired infections and preventable in-hospital cardiac arrests, improved patient experience and involvement in care, and better governance of clinical care.
**Canada**				
Policy				
Canadian Patient Safety Institute. Canadian disclosure guidelines. Being open with patients and family.[68]	2011	'The guidelines are intended to encourage and support healthcare providers, interdisciplinary teams, organizations and regulatory authorities in developing and implementing disclosure policies, practices and training methods.' (p. 13) The following guiding principles underpin the development of the guidelines: patient-centred healthcare, patient autonomy, safe healthcare, leadership support, disclosure is the right thing to do, honesty and transparency. It recommends the following elements to be included in a disclosure policy: policy statement/objectives, definitions of key terms, provision for patient support, provision for healthcare provider support and education, the disclosure process, provision for particular circumstances (Appendix C).		
Canadian Patient Safety Institute. Strengthening commitment for improvement together. A policy framework for patient safety.[69]	2019	A policy framework for patient safety aiming to stimulate conversation and action on the following policy levers: regulations, standards, organisational policies and public engagement.		
Health Standards Organisation & Canadian Patient Safety Institute. The Canadian quality and patient	2020	Defines five areas - people-centred care, safe care, accessible care, appropriate care, and integrated care – for health services to focus on to provide safe and quality care. These goal areas are intended 'to drive improvement and to align Canadian legislation, regulations, standards, organizational policies, and public engagement on patient safety		

For peer review only - http://bmjopen.bmj.com/site/about/guidelines.xhtml

safety framework for health services.[70]		and quality improvement.'(p.3) The framework includes actions guides customised to five stakeholder groups - the public, health teams, health leaders, board members and policymakers - which identify objectives and outcomes for each goal, indicators to track success, and resources.		
Healthcare Excellence Canada. Status of patient safety incident legislation and best practices across Canada. Summary report.[71]	2021	Summarises and compares the status of four types of patient safety incident legislation - mandatory reporting, mandatory disclosure, apology protection, and quality assurance - in federal, provincial and territorial jurisdictions across Canada. The report also describes important characteristics of each type of legislation and comments on current or potential changes in policy direction which may be significant for the future development of legislation.		
Healthcare Excellence Canada, Patients for Patient Safety Canada. Rethinking patient safety. A discussion guide for patients, healthcare providers and leaders.[72]	2023	Summarises discussions with health service users, healthcare professionals, and safety scientists. Describes a new approach to patient safety in Canada, involving (i) the development of a positive culture of safety, with everyone contributing to creating safe conditions, (ii) acknowledging that the absence of harm does not mean that care is safe, and (iii) that all forms of harm matter, including those that are not physical.		
Research		**Aims**	**Methods**	**Findings**
Goldman J, Rotteau L. Evaluation research of Measurement and Monitoring of Safety Framework collaborative.[73]	2020	To (i) understand the effectiveness of a learning collaborative in teaching participants about the Measurement and Monitoring of Safety Framework (MMSF), and (ii) examine the implementation of the MMSF.	Qualitative study with three types of data collection: (i) Interviews (n=36, with participants from teams including emergency, surgery, medicine, cardiology, psychiatry, supportive living and long-term care settings). (ii) Observations of sites (n=5) where MMSF was being implemented and learning sessions and calls	Participants found the sessions to learn about MMSF useful, but some felt overwhelmed by the amount of information. Coaching was felt to play a key role in the implementation of MMSF in participants' organisations. A range of context-specific strategies were used by participants to implement MMSF (e.g. teaching to groups of stakeholders, integrating its language into usual communication, using it to

For peer review only - http://bmjopen.bmj.com/site/about/guidelines.xhtml

			where participants learnt about MMSF (29 hours were spent on site visits and 33 hours at a learning session).<br>(iii) Documentary data collection (e.g. learning session resources, tools being used in MMSF implementation).	inform activities such as safety huddles and incident reporting). Most participants reported positive impacts from MMSF implementation (e.g. changes in thinking about safety and resulting behaviour change); they supported its wider spread but found opportunities to do this beyond their sites were variable.
Goldman J, Rotteau L, Flintoft V, *et al* Measurement and Monitoring of Safety Framework: a qualitative study of implementation through a Canadian learning collaborative *BMJ Quality & Safety* 2023; **32:**470-478.[74]	2023	Journal article summarising the report[73] described above.		
**Ireland**				
Policy				
Health Information and Quality Authority. National standards for safer better healthcare.[75]	2012	Describes eight standards: person-centred care and support, effective care and support, safe care and support, better health and wellbeing, leadership, governance and management, workforce, use of resources, and use of information. These were intended to set expectations for healthcare in Ireland and guide improvement of quality, safety, and reliability. They were replaced recently by a principles-based approach.[76]		
Health Information and Quality Authority. National standards for the conduct of reviews of patient safety incidents.[77]	2017	Details standards based around five themes: governance and accountability, person-centred approach to the review of patient safety incidents, workforce, reviews of patient safety incidents, and sharing the learning for improvement. These are intended to ensure a consistent national approach to reviews of patient safety incidents.		

For peer review only

For peer review only - http://bmjopen.bmj.com/site/about/guidelines.xhtml

Health Information and Quality Authority. Standards development framework: a principles-based approach.[76]	2021	Describes a set of principles which are designed to underpin all national standards for health and social care services, to be used as framework during their development. These principles are a human rights-based approach, safety and wellbeing, responsiveness, and accountability; they are all intended to ensure patients receive person-centred care and support.
Health Service Executive. Patient safety strategy 2019-2024.[78]	2019	Details eight patient safety commitments (including specific actions within each commitment, and the rationale and principles behind them): empowering and engaging patients to improve patient safety, empowering and engaging staff to improve patient safety, anticipating and responding to risks to patient safety, reducing common causes of harm, using information to improve patient safety, and leadership and governance to improve patient safety. Its primary purpose is to guide safety improvement at the service level.
Health Service Executive. Healthcare audit – quality assurance and verification. Standards.[79]	2019	Sets out a framework and basic principles – integrity, objectivity, confidentiality, and competency - for the conduct of healthcare audits in Ireland. All HSE healthcare organisations and private or voluntary bodies with a service level agreement with the HSE are required to be audited (in addition to their internal audit functions) by HSE Healthcare auditors, who are members of the Chartered Institute of Internal Auditors. Audits are independent, objective assessments of the delivery of healthcare services. They provide assurance that standards are being met and recommendations to drive improvement.
Health Service Executive. Incident management framework.[80]	2020	A framework that describes a practical approach all healthcare providers can use to identify, report and review incidents. The framework focuses on the system rather than individual. Six steps are described in the process: preventing incidents by supporting a culture where safety is a priority, identifying incidents and immediate actions, initial reporting and notification, categorisation and initial assessment (from category 1, a major incident, to category 3, a minor incident, which determines who needs to be notified and the level of review required), review and analysis, and improvement planning and monitoring.

For peer review only.

Health Service Executive. HSE Enterprise risk management policy and procedures 2023.[81]	2023	A practical resource for healthcare professionals, whether working in a clinical or management capacity, intended to be used in planning and strategy development, decision-making, and day-to-day management. It sets out policy, processes and procedures for the proactive management of risk in healthcare organisations, based on an enterprise approach (i.e. considers all risks, whether management or service delivery-related, together). This involves identifying and predicting uncertainties and threats, describing and analysing the level of the threat, responding where possible (e.g. through control measures), and reviewing and acting (e.g. communication and escalation regarding a threat).		
Research		**Aims**	**Methods**	**Findings**
Health Information and Quality Authority. Overview report of five years of HIQA monitoring in Irish public acute hospitals against national standards: 2015–2019.[82]	2020	To report key findings, examples of good practice and opportunities for improvement identified during monitoring of four national standards - infection prevention and control, medication safety, maternity services, and nutrition and hydration.	On-site inspections in hospitals between 2015-2019: infection prevention and control (n=161), medication safety (n=64), maternity services (n=22), and nutrition and hydration (n=5).	The greatest improvements were seen in relation to infection prevention and control and medication safety. However, there was variation between hospitals, affecting the quality of care provided. Factors such as insufficient resources hindered the implementation of the national standards.
**New Zealand**				
Policy				
Ministry of Health. Pae Ora (Healthy Futures) Act 2022.[85]	2022	The purpose of the Act is to provide for the public funding and provision of services in order to: (a) protect, promote, and improve the health of all New Zealanders; and (b) achieve equity in health outcomes among New Zealand's population groups, including striving to eliminate health disparities, in particular for Māori; and (c) build towards pae ora (healthy futures) for all New Zealanders.		

For peer review only

For peer review only - http://bmjopen.bmj.com/site/about/guidelines.xhtml

Simmonds S, Carter M, Haggie H, Mills V, Lyndon M, Tipene-Leach D. A Cultural Safety Training Plan for Vocational Medicine in Aotearoa. Te ORA and the Council of Medical Colleges.[86]	2023	Presents 'a plan for cultural safety training that medical colleges can employ in the development of their own cultural safety training programmes and in continuing professional development (CPD) with college fellows.'(p. 4) The plan is specific to the New Zealand context and 'focuses on cultural safety through the lens of Māori patients and their whānau.'(p. 4)
Standards New Zealand. NZS 8134:2021 Ngā Paerewa Health and disability services standard.[87]	2022	The standard promotes the safe provision of fair and equitable services for people and their whānau in New Zealand Aotearoa. It sets the foundations for promoting best practices and fostering continuous improvement in the quality (including safety) of health and disability services.
Te Tāhū Hauora Health Quality & Safety Commission. From knowledge to action: A framework for building quality and safety capability in the New Zealand health system.[88]	2016	'This document describes a high-level framework to guide the development of quality and safety capability across all levels in the health and disability sector'.(p. 4) It aims to address '…existing workforce needs, sustainably building the quality improvement capability of the future workforce, developing specialist roles in quality improvement science, supporting consumer/patient participation, ensuring decision-making is based on data and evidence, and supporting boards to provide leadership that encourages a quality improvement and patient safety focus throughout the sector.'(p. 4)
Te Tāhū Hauora Health Quality & Safety Commission. Clinical governance: guidance for health and disability providers.[89]	2017	Sets out a high-level framework for clinical governance in health and disability services. The values that underpin the framework are consumer wellbeing and safety. The guide is intended for use by all health and disability service providers, regardless of their size or complexity, and their staff, as well as other stakeholders.
Te Tāhū Hauora Health Quality & Safety Commission. Clinical governance framework: collaborating for quality (draft, November 2023).[90]	2023	A draft clinical governance framework to replace the clinical governance framework of 2017. Its purpose is '…to provide a comprehensive approach to enable the health sector to develop their own clinical governance infrastructure, adapted to their clinical context.' (p. 6) 'The framework is intended to support a culture of participatory leadership, where collaboration drives quality' and is '… deliberately inclusive of the health workforce, as opposed to being solely focussed on managers and clinicians, as it is applicable across the health sector and needs to be inclusive of all health environments.' The document also states that: 'The framework has been intentionally designed for the Aotearoa New Zealand context, to enact Te Tiriti o Waitangi [the founding document of Aotearoa New

For peer review only - http://bmjopen.bmj.com/site/about/guidelines.xhtml

		Zealand, signed by the Crown and many hapū and iwi leaders] and strive to achieve equity.'(p. 6)
Te Tāhū Hauora Health Quality & Safety Commission. Guide to doing a learning review.[91]	2023	To enable health providers to review harm by applying the learning review method, in which the reviewer aims 'to understand the realities of everyday work to uncover how harm occurs, minimise risk in the system and support health care workers to do the right thing'(p. 3), including identifying areas for improvement. This method is influenced by concepts such as human factors and resilient healthcare, and takes a systems approach to safety.
Te Tāhū Hauora Health Quality & Safety Commission. National adverse events reporting policy.[92]	2023	The purpose of the policy is to provide 'a consistent way to understand and improve through reporting, reviewing and learning from all types of harm.' (p. 5). It is intended to guide reporting to Te Tāhū Hauora [Health Quality and Safety Commission], and the use of information from learning reviews, and quality improvement approaches to strengthen system safety. The policy differs from the previous policy by shifting the focus to 'system safety' and restorative response in the context of partnership with consumers and whānau.
The National Collaborative for Restorative Initiatives in Health. He Maungarongo ki Ngā Iwi: Envisioning a restorative health system in Aotearoa New Zealand.[93]	2023	Provides an overview of the systems that mitigate and respond to healthcare harm in Aotearoa NZ, adopting a human-centred and relational approach. It presents a framework of principles and practices and defines restorative systems as being 'distinguished by an emphasis on relational principles, practices and goals that promote and restore human dignity and wellbeing.'(p. 10)

[a]The original NTA 8009 document was updated twice, in 2011 and 2014, before the most recent NEN 8009 (2018) version.

*In Dutch, accessed in translation.

For peer review only - http://bmjopen.bmj.com/site/about/guidelines.xhtml

Supplementary material 5: Search information

Medline via Ovid, 8/12/23:

1 (safety adj3 management adj3 system*1).ti,ab. (588)
2 (safety adj3 management adj3 program*).ti,ab. (93)
3 1 or 2 (670)
4 (patient*1 adj2 (safety or harm* or risk*)).ti,ab. (204300)
5 patient harm/ or patient safety/ (25896)
6 4 or 5 (219200)
7 ((safety or hazard* or risk or risks) adj2 (accountability or assess* or assurance* or commitment* or communicat* or control* or coordinat* or cultur* or education or framework* or guidance or implement* or improvement* or leadership* or management or measurement* or monitor* or plan or plans or planning or policy or policies or promot* or quality or regulat* or reporting or responsibilit* or strateg* or training)).ti,ab. (285563)
8 *Safety Management/ (13937)
9 7 or 8 (295644)
10 system*1.ab. /freq=2 (1032216)
11 system*1.ti. (682356)
12 10 or 11 (1448707)
13 6 and 9 and 12 (3003)
14 3 or 13 (3632)

Embase via Ovid, 9/2/2024

1 (safety adj3 management adj3 system*1).ti,ab. 924
2 (safety adj3 management adj3 program*).ti,ab. 128
3 1 or 2 1038
4 (patient*1 adj2 (safety or harm* or risk*)).ti,ab. 343333
5 patient safety/ or patient harm/ or patient risk/ 173057
6 4 or 5 469009
7 ((safety or hazard* or risk or risks) adj2 (cultur* or education or implement* or improvement* or management or measurement* or

For peer review only - http://bmjopen.bmj.com/site/about/guidelines.xhtml

monitor* or quality or regulat* or reporting or responsibilit* or strateg* or training)).ti,ab. 123921
8 System*1.ab. /freq=2 1290950
9 System*1.ti. 770274
10 8 or 9 1735239
11 6 and 7 and 10 3017
12 3 or 11 3986
13 limit 12 to conference abstract status 996
14 12 not 13 2957

CINAHL via EBSCOhost, 9/2/2024

#	Query	Results
S8	S1 OR S7	2,564
S7	S3 AND S4 AND S6	2,373
S6	S2 OR S5	107,816
S5	(MM "Patient Safety")	38,651
S4	TI (system or systems) OR AB (system or systems)	516,337
S3	TI (((safety or hazard* or risk or risks) N1 (cultur* or education or implement* or improvement* or management or measurement* or monitor* or quality or regulat* or reporting or responsibilit* or strateg* or training)).) OR AB (((safety or hazard* or risk or risks) N1 (cultur* or education or implement* or improvement* or management or measurement* or monitor* or quality or regulat* or reporting or responsibilit* or strateg* or training)).)	37,449
S2	TI ((patient* N1 (safety or harm* or risk*))) OR AB ((patient* N1 (safety or harm* or risk*)))	81,650

For peer review only - http://bmjopen.bmj.com/site/about/guidelines.xhtml

S1	TI safety N3 management N3 system* OR AB safety N3 management N3 system*	238

Web of Science: 10/2/2024

- WOS.SCI: 1900 to 2024
- WOS.AHCI: 1975 to 2024
- WOS.ESCI: 2015 to 2024
- WOS.ISTP: 1990 to 2024
- WOS.SSCI: 1900 to 2024
- WOS.ISSHP: 1990 to 2024

Searches:
1: TS=(safety near/1 management) AND TS=((management near/1 (system or systems))) Results:3363
2: TI=((patient* near/2 (safety or harm* or risk*))) Results: 61188
3: TS=(safety near/1 (cultur* or education or implement* or improvement* or management or measurement* or monitor* or quality or regulat* or reporting or responsibilit* or strateg* or training)) Results: 78695
4: TS=(hazard* near/1 (cultur* or education or implement* or improvement* or management or measurement* or monitor* or quality or regulat* or reporting or responsibilit* or strateg* or training)) Results: 8001
5: TS=(risk near/1 (cultur* or education or implement* or improvement* or management or measurement* or monitor* or quality or regulat* or reporting or responsibilit* or strateg* or training)) Results: 122887
6: TS=(risks near/1 (cultur* or education or implement* or improvement* or management or measurement* or monitor* or quality or regulat* or reporting or responsibilit* or strateg* or training)) Results: 122887
7: TS=(system or systems) Results: 11251037
8: #6 OR #5 OR #4 OR #3 Results: 205099
9: #7 AND #8 AND #2 Results: 1288
10: TS=(patient* or health* or hospital*) Results: 11662421
11: #10 AND #1 Results: 1116
12: #9 OR #11 Results: 2379

For peer review only - http://bmjopen.bmj.com/site/about/guidelines.xhtml

For peer review only

Details of website searches of patient safety organisations in included countries

Organisation	URL	Search date		No. of hits	No. of includes
**Australia**					
Australian Commission on Safety and Quality in Health Care	https://www.safetyandquality.gov.au/	01/12/2023	safety management system	141	5
Department of Health and Aged Care	https://www.health.gov.au/	06/12/2023	safety management system	171	0
Australian Institute of Health and Welfare	https://www.achs.org.au/	06/12/2023	safety	63	0
Australian Council on Healthcare Standards	https://www.achs.org.au/	06/12/2023	safety	43	0
**Canada**					
Healthcare Excellence Canada	https://www.healthcareexcellence.ca/en	05/01/2024	safety	406 (200 screened)	4

For peer review only - http://bmjopen.bmj.com/site/about/guidelines.xhtml

Institute for Safe Medication Practices (ISMP) Canada	https://ismpcanada.ca	05/01/2024	safety management system	11	0
**Ireland**					
Health Information and Quality Authority (HIQA)	https://www.hiqa.ie	01/02/2024	Publications - safety	104	4
National Patient Safety Office, Ireland	https://www.gov.ie/en/campaigns/af4b6-national-patient-safety-office/	01/02/2024	safety	44	0
National Quality and Patient Safety Directorate	https://www2.healthservice.hse.ie/organisation/nqpsd/	01/02/2024	n/a	No search function	3
**Netherlands**					
NIVEL (The Netherlands Institute for Health Services Research)	https://www.nivel.nl/en	01/02/2024	safety management	76	2
Dutch Patient Safety Programme	https://www.vmszorg.nl/	05/12/2023		In Dutch	2
Netherlands Health and Youth Care Inspectorate, Ministry of Health Welfare and Sports	https://english.igj.nl	05/12/2023	safety	56	0
**New Zealand**					

For peer review only

Te Tāhū Hauora Health Quality and Safety Commission	https://www.hqsc.govt.nz/	01/02/2024	Resource library - safety management system	115	9

For peer review only

For peer review only - http://bmjopen.bmj.com/site/about/guidelines.xhtml

BMJ Open

The implementation of Safety Management Systems (SMS) in healthcare: a systematic review and international comparison

Journal:	*BMJ Open*
Manuscript ID	bmjopen-2025-107772.R2
Article Type:	Original research
Date Submitted by the Author:	26-Feb-2026
Complete List of Authors:	de Bell, Siân ; University of Exeter, Exeter HS&DR Evidence Synthesis Centre, Department of Health & Community Sciences<br>Zhelev, Zhivko; University of Exeter, Exeter HS&DR Evidence Synthesis Centre, Department of Health & Community Sciences<br>Bethel, Alison; University of Exeter, Exeter HS&DR Evidence Synthesis Centre, Department of Health & Community Sciences<br>Clarke, Maria; University of Exeter, Exeter HS&DR Evidence Synthesis Centre, Department of Health & Community Sciences<br>Anderson, Rob; University of Exeter, Exeter HS&DR Evidence Synthesis Centre, Department of Health & Community Sciences<br>Thompson Coon, Jo; University of Exeter, Exeter HS&DR Evidence Synthesis Centre, Department of Health & Community Sciences
**Primary Subject Heading**:	Health policy
Secondary Subject Heading:	Health services research
Keywords:	Systematic Review, Safety, Organisation of health services < HEALTH SERVICES ADMINISTRATION & MANAGEMENT

SCHOLARONE™
Manuscripts

I, the Submitting Author has the right to grant and does grant on behalf of all authors of the Work (as defined in the below author licence), an exclusive licence and/or a non-exclusive licence for contributions from authors who are: i) UK Crown employees; ii) where BMJ has agreed a CC-BY licence shall apply, and/or iii) in accordance with the terms applicable for US Federal Government officers or employees acting as part of their official duties; on a worldwide, perpetual, irrevocable, royalty-free basis to BMJ Publishing Group Ltd ("BMJ") its licensees and where the relevant Journal is co-owned by BMJ to the co-owners of the Journal, to publish the Work in this journal and any other BMJ products and to exploit all rights, as set out in our licence.

The Submitting Author accepts and understands that any supply made under these terms is made by BMJ to the Submitting Author unless you are acting as an employee on behalf of your employer or a postgraduate student of an affiliated institution which is paying any applicable article publishing charge ("APC") for Open Access articles. Where the Submitting Author wishes to make the Work available on an Open Access basis (and intends to pay the relevant APC), the terms of reuse of such Open Access shall be governed by a Creative Commons licence – details of these licences and which Creative Commons licence will apply to this Work are set out in our licence referred to above.

Other than as permitted in any relevant BMJ Author's Self Archiving Policies, I confirm this Work has not been accepted for publication elsewhere, is not being considered for publication elsewhere and does not duplicate material already published. I confirm all authors consent to publication of this Work and authorise the granting of this licence.

For peer review only

The implementation of Safety Management Systems (SMS) in healthcare: a systematic review and international comparison

Siân de Bell[1* a], Zhivko Zhelev[1*], Alison Bethel[1], Maria Clarke[1], Rob Anderson[1], Jo Thompson Coon[1]

[1]Exeter HS&DR Evidence Synthesis Centre, Department of Health & Community Sciences, University of Exeter Medical School, University of Exeter, Exeter, UK

*Joint first author

[a] Corresponding author: Sian de Bell s.c.de-bell@exeter.ac.uk

For peer review only

For peer review only - http://bmjopen.bmj.com/site/about/guidelines.xhtml

Abstract

Objectives Many industries where safety is a priority (e.g. aviation) use safety management systems (SMSs) but evidence on their use in healthcare is needed to determine whether they could support patient safety improvement. We investigated the application of national-level SMSs to patient safety in terms of effectiveness, implementation and experience.

Design Systematic review using a case study approach.

Data sources We identified patient safety organisations in each country and searched their websites. We also searched MEDLINE (in December 2023) and Embase (via Ovid), CINAHL (EBSCO) and Web of Science (in February 2024).

Eligibility criteria Any evidence from five high-income countries that have publicly funded healthcare systems with universal coverage: Australia, Canada, Ireland, New Zealand and the Netherlands. We included publications on the effectiveness, implementation or experience of the patient safety approach in each country.

Data extraction and synthesis Included evidence was summarised and mapped onto an initial analytical framework based on analysis of SMSs in high-risk industries, enabling cross-country comparisons. Drafts were shared with experts from patient safety organisations in each country for comment. Formal quality appraisal was not possible as most evidence was non-evaluative.

Results Fifty-three publications were included, from Australia (5), Canada (7), Ireland (8), New Zealand (9), and the Netherlands (24). Only the Netherlands implemented a patient safety programme explicitly based on an SMS approach. Some aspects of patient safety in hospitals improved as a result but there was significant variation in both the implementation of the programme and attributed outcomes.

In the other four countries, the main components of an SMS were identified to some extent, along with evidence that their patient safety approaches had been influenced by concepts from high-risk industries and 'safety science' more widely.

Conclusions Emerging ideas from high-risk industries (beyond SMSs) and broader safety science have influenced all countries, reflecting increasing awareness of the need for initiatives to be context-specific to be successful. However, their implementation and impact need further evaluation.

Registration: CRD42023487512

Key words
safety management systems; patient safety; healthcare; systematic review

Strength and limitations

- This systematic review developed a framework defining the key components of a safety management system, from their use to improve safety in high-risk industries (e.g. aviation), and applied this to analyse national patient safety approaches in healthcare.
- We included a broad evidence base, searching the websites of patient safety organisations alongside searching for peer-reviewed articles, and consulting safety experts in included health systems to verify our understanding of their approach to safety management in healthcare.

For peer review only - http://bmjopen.bmj.com/site/about/guidelines.xhtml

- We focused on high-income countries with healthcare systems comparable to the UK. However, we were only able to include those where evidence (e.g. policy documents) was available in English.
- Our analysis was based mainly on non-evaluative documents, so we were not able to conduct quality appraisal or make firm conclusions about the impacts of adopting a safety management system approach in healthcare.

1. Background

All healthcare systems work in various ways to improve patient safety, from creating policies at national and organisational levels to implementing interventions as a result. The challenges of ensuring safe healthcare are recognised by the World Health Organisation (WHO), which has developed the Global Patient Safety Action Plan 2021-30 to provide countries with a framework to improve their national patient safety approaches.[1] However, patient safety improvement is not an easy process; for example, a national review of patient safety in England in 2024 found that, compared to the previous review in 2022, performance had worsened in a number of areas (e.g. maternity care).[2] Globally, patients continue to be harmed by failures or errors, with examples of safety incidents including mistakes associated with the use of medication, such as in prescribing or dispensing, and wrong site surgery.[3] In high income countries, one in ten patients are thought to be subject to an adverse event whilst receiving hospital care each year,[1] whilst in England, it is estimated that 237 million medical errors occur annually, contributing to more than 1,700 deaths.[4]

Safety is also a significant priority in other industries - such as aviation, nuclear energy and oil and gas - where errors could have serious consequences for people or the environment.[3] Many of these 'safety-critical' industries, and companies within them, have improved safety by taking a systems approach – considering the many interacting organisation and process-level factors (e.g. culture, technology, team relationships) that may result in errors or adverse events.[1] This has led to the development of international or national policies and standards for safety, with organisations operationalising these in an organised approach to managing safety known as a safety management system (SMS).[5] There is no single definition for an SMS, but they are generally considered to have four key components:

- Leadership commitment and safety policy – an expressed leadership commitment to safety with documentation of responsibilities and processes for safety within an organisation,[3, 6]
- Safety risk management – the identification of hazards and risk, and assessment of how to mitigate these,[3]
- Safety assurance – monitoring and measuring safety within the organisation and ensuring continuous improvement,[3]
- Safety promotion and culture – training, education and communication of safety to staff at all levels.[7]

The underlying theory behind SMSs is that by focusing on performance - monitoring and achieving desired outcomes - rather than solely on compliance, they facilitate improvement.[3] In 2012, a systematic review of SMSs in three safety-critical industries – aviation, marine and rail – concluded that the approach led to improvements in safety (e.g. reduced accident rates).[8] This suggests that they have the potential to improve safety in other sectors where safety is paramount, such as healthcare. The WHO Global Patient Safety Action Plan 2021-30 recognises "the strong parallels [of healthcare] with the experience of other high-risk industries, thus creating opportunities for transfer learning" (p2).[1]

For peer review only - http://bmjopen.bmj.com/site/about/guidelines.xhtml

The basic components of an SMS (as described above, with further detail given in Table 1) are quite generic and transferrable.[9] It is not possible to provide a more detailed description of a single SMS, as in order to be effective, the specifics need to be adapted to the context in which they are being used, as can be seen from SMSs in other high-risk industries.[10] For example, the aviation, nuclear, and oil and gas industries all have international guidelines regarding safety management.[6, 7, 11] In aviation, these are used by countries to develop state safety programmes with detailed requirements for safety management (i.e. SMSs) by individual organisations. Nuclear safety is considered a national policy issue, with states responsible for the safety of their own nuclear facilities. The oil and gas industry differs from both in that it identifies and manages safety risks within the context of achieving performance goals and stakeholder benefits.[11] In all these industries, individual organisations implement policy and guidance according to their own ways of working.

Whilst healthcare systems globally are working to improve patient safety, countries have different starting points in terms of the organisation and management of their healthcare, and different pre-existing system components (e.g. approaches to monitoring patient safety incidents). Most healthcare systems have some components of an SMS, such as policies for improving patient safety,[12] with some countries - notably the Netherlands[13] - explicitly developing and promoting an SMS approach. Their experiences offer opportunities for learning, and determining whether the principles of SMS are effective in improving safety in healthcare, and if so, how they can be adapted from industries in which they are used, and implemented within a healthcare system. In this review, we focus on the high-level influence or coordination of the implementation of an SMS approach, rather than the operation of organisation-level SMSs.

1.2 Objectives

This systematic review investigated the application of SMSs to patient safety in healthcare, to consider whether and how they could be implemented more widely, asking:

- How are the components of an SMS reflected in the healthcare policy documents of the included countries?
- What research or other relevant evidence is available regarding the effectiveness, implementation or experience of SMSs within healthcare?
- What does evidence from beyond the UK tell us about the effectiveness, implementation or experience of SMSs within healthcare?

This review was commissioned by the National Institute for Health and Care Research (NIHR) Health Service Delivery Research (HSDR) programme on behalf of the NHS England Patient Safety Team and Department of Health & Social Care, to inform the further development of the NHS England's patient safety policy and practice. The UK was not included in the review as: (i) the NHS has not implemented an SMS; and (ii) the intention was to learn from the experiences of other countries, with different starting points in terms of the organisation and management of healthcare, and different pre-existing (safety) system components, regarding their patient safety approaches.

2. Methods

This systematic review was registered on PROSPERO (CRD42023487512) and can be found in Supplementary Material 1. A summary of the methods is provided below; further detail can be found in the full report.[14]

For peer review only - http://bmjopen.bmj.com/site/about/guidelines.xhtml

2.1 Inclusion and exclusion criteria

Our main criterion for inclusion was the national implementation of an SMS approach in the healthcare system; our scoping searches provided indicative evidence of the adoption of an SMS approach, or some of its key components, to improve patient safety in few countries. We also decided to restrict the review to countries that were comparable to the UK and each other, in that they are high-income countries[15] with national healthcare systems offering universal coverage (via general taxation or social insurance).[16, 17] Consequently, evidence from the following countries was eligible for inclusion in the review: Ireland, Australia, New Zealand, Canada and the Netherlands. Additionally, the majority of evidence from these countries was available in English and we had limited ability to translate non-English documents.

As this is an emerging, policy-relevant topic, we included a broad range of types of evidence that should contain evidence relevant to our review questions[18]:

- Policy documents, including those related to the implementation of an SMS or patient safety approach,,[19]
- Published or unpublished research / evaluations (quantitative, qualitative or mixed methods),
- Other evidence (e.g. patient and staff experience from organisations' websites).

Evidence relating to any service user population and any healthcare setting were included in the review; study participants could include healthcare professionals, service users, or carers. In terms of focus, we included evidence on any policies, initiatives, or programmes related to the key components of an SMS (as defined above and in Table 1) or patient safety approach. Studies were excluded where their focus was not linked to a component of an SMS, or was not patient safety (e.g. occupational health and safety). See Supplementary Material 2 for more information.

2.2 Search strategy and study selection

MEDLINE (from 1946) was searched in December 2023 and Embase (from 1974) (via Ovid), CINAHL Ultimate (from 1937) (EBSCO) and Web of Science (WoS): Science Citation Index (from 1990), Social Science Citation Index (from 1900), Arts and Humanities Index (from 1975), Conference Proceedings, Science, Social Science and Humanities (from 1990), Emerging Sources Citation Index (from 2015) in February 2024; (see search strategies in Supplementary Material 3. In March 2024, forwards citation searching was undertaken in Web of Science using eight papers (from the Netherlands, as the only country explicitly using an SMS approach). The reference lists of all publications that met our inclusion criteria were checked for relevant publications.

Key policy documents and evidence that might not be captured through database searches (e.g. programme reports) were identified by searching the websites of, and contacting, national patient safety organisations from each of the included countries (Supplementary Material 3). To understand the context within each country and identify further sources of evidence, we also held online meetings with patient safety/health policy experts from:

- Australia - Australian Commission on Safety and Quality in Health Care; Department of Health and Aged Care (federal government)
- Canada - Healthcare Excellence Canada
- Ireland - Health Information and Quality Authority (HIQA); Royal College of Surgeons Ireland
- Netherlands - Netherlands Institute for Health Services Research (NIVEL)
- New Zealand - Health Quality and Safety Commission

For peer review only - http://bmjopen.bmj.com/site/about/guidelines.xhtml

All results from the database searching were independently screened by two reviewers (SDB, ZZ). The full text of papers which were judged to meet the inclusion criteria were obtained, along with policy and other documents identified by web-searching. Two reviewers (SDB, ZZ) assessed each full text independently for inclusion. At both stages, disagreements were settled through discussion.

In December 2025, we checked that patient safety policy documents from the included countries remained the same, and conducted further forward citation chasing on included studies.

2.3 Data extraction and synthesis

Whilst creating the review protocol and searching for studies, we developed an analytical framework of the components of an SMS by reviewing policy documents and review articles from high-risk industries (focusing on aviation,[7] oil and gas[11] and nuclear energy[6], see Table 1). Data extraction and thematic analysis of evidence from the healthcare systems of each included country was conducted by two reviewers (SDB, ZZ), who held regular discussions with the wider team, and guided by this framework.

Our protocol specified that we would only include countries which explicitly stated that they were using an SMS approach in their healthcare system, with our scoping searches suggesting that Australia, Canada, Ireland, the Netherlands and New Zealand all met this criterion. However, after screening studies for inclusion, we found that the Netherlands was the only country where this commitment to an SMS approach was explicit. Consultation with our main stakeholders, the NHS Patient Safety Team, indicated that understanding 'the principles underpinning SMS may help healthcare organisations to respond more effectively to patient safety risks' (p. 8).[3] We therefore decided to make a change to our protocol, continuing to include the other four countries in the review, and extracting data on their approaches to patient safety and how these mapped to the components of an SMS.

An additional change was made to the protocol given the quantity of studies retrieved by the database searches. As the number of studies found was too large to allow a meaningful synthesis, we classified this evidence on impact and implementation according to whether they were 'rich' or 'thin', with 'rich' studies considered to be those with conceptual information which would allow us to answer the review's objectives and where the study data was close to the review objective.[20] 'Thin' studies did not contain conceptually rich data, and/or their objectives were not aligned with those of the review.[20] Our analysis concentrates on the rich evidence due to our focus on the implementation of patient safety approaches nationally and the need, therefore, for detailed, explanatory accounts of implementation at this level. Thin studies were included to illustrate the way in which a programme was designed or implemented, and not because of their specific results, as they did not provide the necessary conceptual richness, tending to focus on settings and outcomes that were not generalisable (e.g. specific organisations, small-scale interventions, or outcomes less relevant to our research questions such as the accuracy of a tool).

A case study approach was taken to the analysis of policy documents and other evidence, to fully describe and provide contextual information on the patient safety approach in each country, enabling understanding of how these approaches related to the components of an SMS and cross-country comparison.[19, 21] Each country's approach was described and analysed separately, and draft descriptions for each country shared with experts from patient safety organisations in the respective countries for correction or comment. In the final step of the analysis, we compared the experience and implementation of the SMS or patient safety approach across countries. This involved development of the initial framework - the four main components of an SMS remained the same, but subcategories were revised based on

For peer review only - http://bmjopen.bmj.com/site/about/guidelines.xhtml

the information in the included evidence. Included publications were not quality appraised as the majority were not empirical studies so quality appraisal would not have made a significant contribution to our understanding of the application of SMSs in healthcare. We did consider uncertainties in the evidence, stemming from methodological limitations, when reporting on the Netherlands (as the only country that had explicitly used an SMS approach).

For peer review only

For peer review only - http://bmjopen.bmj.com/site/about/guidelines.xhtml

Table 1 Framework of the components of an SMS

**Key component**	**Subcategory**	**Definition**
Leadership commitment and safety policy	Management commitment and accountability	Individuals in the organisation who are accountable for safety, and their exact role and responsibilities, should be clearly identified.[3] These individuals should ensure that the people they are managing have the capabilities to complete their required activities safely and provide support for them to do this (e.g. resources).[6]
	High-level (policy) documents	The safety policy should outline the aims and objectives of an organisation in terms of safety.[3]
	1. Implementation plan	There should also be a supporting document which details how these will be fulfilled i.e. a framework for the management of safety, including safety standards detailing what needs to be done and by whom, and an improvement strategy.[6]
	2. External and internal boundaries of SMS	External boundaries might include requirements for contractors to have an SMS in place or be considered within the SMS of the organisation. Internally, the relation of the SMS to other management systems (e.g. quality) needs to be defined.
	Coordination of the emergency response plan	Planning for immediate activities in response to an unplanned event e.g. an accident.[7]
Safety risk management	Hazard identification processes	There should be formal processes, of which there are likely to be more than one (e.g. audits, incident reporting), in place to identify hazards.[7]

For peer review only - http://bmjopen.bmj.com/site/about/guidelines.xhtml

	Risk assessment and mitigation processes	This will identify the acceptability of the risk and appropriate measures to control it.[6]
Safety assurance	Safety performance monitoring and measurement	This involves the development of measures, both reactive and proactive, to routinely monitor safety performance within the organisation. It may also involve periodic audits and reviews of performance (including compliance), whether internally or externally (e.g. by regulators).
	Internal safety investigations	Systems are needed to allow the reporting of safety incidents, and processes to investigate their causes.
	Continuous improvement	Organisations should have arrangements to ensure that actions are taken if issues are identified as a result of monitoring and investigations, including ensuring that these are completed within appropriate timescales.
Safety promotion and culture	Training and education	Training may be specific to an individual's role, or to gain a wider understanding of safety issues, but is essential to ensure competence.[6] It should be identified, documented, and reviewed and revised regularly.
	Safety communication	This may be formal (e.g. safety meetings), or informal (e.g. mention of issues in team briefings), but needs to be open and allow individuals to understand and accept why standards of safety are required, and provide feedback.[6]
	Presence of safety culture	Also referred to as a just culture, allowing open communication and reporting of incidents or hazards.

For peer review only

2.4 Public and patient involvement and engagement

Due to the focus of review, we sought public collaborators who had some knowledge of the topic. We held meetings with three public collaborators, two of whom had knowledge of safety management in other industries (chemical and police/counter-terrorism) and one who had experienced a safety incident in the NHS and has been campaigning to raise awareness of patient safety. In these meetings, we discussed their experiences of safety in other industries (where relevant) and of patient safety in the NHS, as well as their views on the improvement of patient safety. Our public collaborators provided important contextual information on safety management and SMS in other industries (e.g. the importance of clear accountability for safety) and their potential use in the NHS. They also informed specific aspects of the review (e.g. discussions of their own safety experiences in the NHS highlighted areas to consider in our data extraction and analysis, such as approaches to involving patients in safety policy and incident investigations and applying learning from safety incidents).

3. Results

Database searches resulted in 11,532 hits; 6,913 records were screened at title and abstract level. Of those, 158 were selected for full text screening and 89 were found to meet the inclusion criteria. Searches of websites resulted in 32 publications being included in the review, along with 22 documents from other sources (14 from the reference lists of included documents and eight from expert suggestions). This resulted in a total of 143 studies being included in the review. Further details can be found in Figure 1 and Supplementary Material 4.

Figure 1 PRISMA diagram of the screening process [figure uploaded separately]

Due to the quantity of evidence found, we grouped publications into two broad categories (Table 2):

- Descriptions of each country's approach to patient safety, including influences from high-risk industries (mainly policy documents but also outputs e.g. opinion papers).
- Evidence on the impact and implementation of a specific patient safety approach, or aspects of it (mainly research papers and project reports). As described above, these were categorised further as:
    - 'rich' – reporting on the impact or implementation of the approach, or an essential part of it, and
    - 'thin' – papers with a very narrow focus and limited relevance to our review question (e.g. the accuracy or acceptability of a risk assessment tool implemented as part of a patient safety programme).

Table 2 Number of evidence sources per category per country.

Country	'Rich' documents (n=53)			'Thin' documents (n=90)
	Policy	Research	Other*	
Netherlands	2	20	2	22
Australia	3	2	0	37[a]

For peer review only - http://bmjopen.bmj.com/site/about/guidelines.xhtml

Canada	5	2	0	27[a]
Ireland	7	1	0	2
New Zealand	9	0	0	6[a]

*Such as opinion papers.

[a]Some included evidence had information from multiple countries (Australia and Canada (n=2) and Australia and New Zealand (n=2)) so has been included in multiple categories.

Our analysis was based primarily on the descriptive and rich evidence. We focused on 53 publications, which can be found in Supplementary Material 5, from Australia (5), Canada (7), Ireland (8), New Zealand (9), and the Netherlands (24) (Table 1). Some 'thin' evidence is used to illustrate specific points (see full list in Supplementary Material 4). Below, we describe the patient safety approach in each country then compare these approaches.

3.1 Netherlands

The Netherlands was the only country to explicitly use an SMS approach, in the form of its national patient safety programme (PSP), which was launched in 2008 and ran until the end of 2012.[22] The programme was implemented in all public hospitals and based on the premise that change in the structure or processes of an organisation is necessary to see a change in outcomes.[23] It had two pillars: (i) hospitals were required to implement a certified SMS, and (ii) a set of guidelines focusing on improving patient safety for ten priority themes.[13] The aim was a 50% reduction in adverse events and potentially preventable deaths over a period of 5 years.[13] The two pillars of the PSP continued to be implemented after 2012, with development and revisions based on evaluations of its success. A new safety programme, based on a Safety-II perspective (see Glossary), began in 2020.[24]

The basic SMS requirements were agreed by key stakeholders and published in 2007 (NTA 8009).[25] The complexity of implementing an SMS was acknowledged, with healthcare providers required to carry this out in stages. In Phase 1 the basic requirements of an SMS were developed (leadership, management, personnel, patient participation, prospective risk assessment, retrospective risk assessment and improvements), with further requirements added in Phase 2 (communication, third-party management, and control measures).[25, 26] The latest version of the document specifying these requirements, NEN 8009, was published in 2019; it introduces a new perspective on patient safety based on Safety-II and resilience engineering (see Glossary). These concepts emphasise the importance of learning from successful, 'incident-free' practice as well as from the investigation and prevention of incidents.[26]

There were ten high-priority themes for patient safety, such as patient mix-up (see Glossary). Expert groups were convened for each theme, and guides were developed describing structure, processes, and outcomes. Interventions to improve patient safety were explained during national theme conferences. The programme also had a website where resources and patient-facing materials were available. Whilst the initial focus was on adult care, six of the patient safety themes were later expanded to include paediatric hospital care.

For peer review only - http://bmjopen.bmj.com/site/about/guidelines.xhtml

3.2 Australia

In Australia, patient safety is primarily a national (i.e. federal government) responsibility.[27] However, state and territory governments are responsible for their own public hospital systems, and the licencing and regulation of the private hospital sector, as recognised in the National Health Reform Act 2011 (NHRA - which specifies responsibilities for funding, operating, managing and regulating the health system).[27] The systems and processes used to ensure safety therefore differ between territories and states.[27]

The Australian Commission for Safety and Quality in Health Care developed the National Safety and Quality Health Service (NSQHS) Standards with the aim of ensuring the relevant systems are in place within a healthcare organisation to ensure quality of care.[28] The first edition was published in 2012, the latest in 2021.[28] The NSQHS Standards detail requirements for healthcare organisations regarding clinical governance and partnering with consumers, as well as specific areas of clinical management. They require providers to have an organisation-wide clinical incident management and investigation system, with an open disclosure policy consistent with the Australian Open Disclosure Framework.[27] Certain indicators which should be measured, such as sentinel events, have been developed by the Commission, and the Australian Health Performance Framework details a common set of metrics for public reporting of safety and quality data.

Australia has not used an SMS approach in its healthcare system, although "*the healthcare sector began to draw on lessons from other high risk industries*" such as aviation and mining, as well as using human factors analysis, to develop its patient safety approach.[27] These concepts from high-risk industries have influenced specific elements of Australia's approach (e.g. surgical checklists).

3.3 Canada

The responsibility for delivering health services lies with the provincial and territorial governments, leading to variation in how patient safety is approached in Canada. The Canadian Quality and Patient Safety (CPQS) Framework was launched in 2020 and provides guidance to drive patient safety and quality improvement in all healthcare sectors and settings. Whilst it is intended to align Canadian legislation, regulations, standards, organizational policies, and public engagement nationally,[29] there is currently variation between governments. Not all jurisdictions have quality and safety plans or policies regarding key elements of patient safety.[30]

Healthcare Excellence Canada, an independent non-profit organisation funded primarily by Health Canada - a department of the federal government – has produced a discussion guide that details the recent change in Canada's approach to patient safety. There has been a move from 'traditional' patient safety improvement (e.g. scorecards and audits), to a focus on creating a proactive and inclusive environment, with a culture and processes that support continuous safety improvement.[31] Canada has drawn on learning from high-risk industries, with the Measurement and Monitoring of Safety Framework (MMSF) underpinning its new approach to patient safety but has not taken an SMS approach.[32]

3.4 Ireland

The Health Information and Quality Authority developed the National Standards for Safer Better Healthcare in 2012; these detail expectations for healthcare, and guide improvement of quality, safety, and reliability.[33] They were replaced by a principles-based approach in 2021.[34] However, the Health Service Executive has developed a national patient safety strategy which builds on the Standards, to be implemented and guide improvement at a local level.[35]

For peer review only - http://bmjopen.bmj.com/site/about/guidelines.xhtml

Irish healthcare providers are required to have an incident reporting system.[36] Incidents are entered into the national system and reviewed externally or internally depending on the severity of the incident.[36, 37] Providers are also expected to undertake clinical and non-clinical audits (which may be part of national or local programmes), implement improvements based on these,[35, 38] and assess and manage risks, with a formal process in place to do this.[39] Sláintecare, an improvement plan and strategy intended to reform and develop health services in Ireland over the next decade and into the future, is currently being implemented in Ireland; the provision of healthcare, including the improvement of safety, is consequently evolving.[35]

Whilst Ireland has not taken an SMS approach, the influence of high-risk industries can be seen in various elements of their approach. The national patient safety strategy details an action regarding information and training for staff drawing on "*patient safety and reliability science, systems thinking, audit, quality improvement methodologies, change management, human factors and multidisciplinary team working for safety*",(p.13)[35] with an example of this being the delivery of simulation-based education and human factors training to improve patient safety (see Glossary).[40, 41]

3.5 New Zealand

Patient safety in New Zealand is a central focus of the healthcare system, with the Te Tāhū Hauora Health Quality & Safety Commission (Te Tāhū Hauora), established in 2010, playing a pivotal role in improving healthcare quality and safety across the country.[42] The Pae Ora (Healthy Futures) Act 2022 replaced the country's 20 District Health Boards, aiming to create a more centralised, effective and efficient healthcare system.[42] The Act[42] and the updated Nga Paerewa Standard Health and disability services standard[43] are associated with important changes in the New Zealand's patient safety policy, such as the 'Healing, Learning, and Improving from Harm: National Adverse Events Policy'[44] which took effect from 1st July 2023 and the new 'Clinical Governance Framework: Collaborating for quality' (which is still in a process of consultation).[45]

The new framework has been intentionally designed for the cultural context of New Zealand, with service users and their families seen as 'partners in care'.[45] Elements of safety are emphasised, such as the importance of having processes and systems in place to ensure quality and safety (e.g. clinical risk management systems to deal with non-compliance with clinical standards and policies), and of focusing on 'system safety' – managing risk within a systems perspective – and learning how the healthcare system shapes the experiences of consumers and staff.[45] The MMSF is suggested as a tool that could help decision makers to assess the overall safety of their organisation. To assess the quality of the health and disability system, Te Tāhū Hauora has adopted a range of baseline measures and indicators:

- Atlas of Healthcare Variation
- Quality and Safety Markers (QSMs)
- Quality Accounts
- Health Quality and Safety Indicators (HQSIs)
- Prevention quality indicators.

The current (2023) version of the clinical governance framework (in consultation) includes a number of ideas (e.g. systems safety, resilient organisations) which are linked to developments in high-risk industries.[45, 46] For example, the 'learning review' method was originally developed for the investigation of firefighter incidents in the context of the US Forest Service and draws on a number of concepts, such as human factors and ergonomics,

For peer review only - http://bmjopen.bmj.com/site/about/guidelines.xhtml

systems safety and resilient organisations. It was specifically developed for 'adaptive complex systems' – complex systems with high level of uncertainty that interact, learn and adapt over time – such as healthcare.[46]

3.6 Comparison

Although the Netherlands was the only country to explicitly use an SMS approach, the main components of an SMS were identified, to varying extents, in the patient safety policies and initiatives of the other four countries, as described in Tables 3-5. Whilst the interdependence of these elements was acknowledged, they were often detailed in different policies and within the scope of different organisations. Key features that were seen in all five countries, organised according to the components of an SMS, included:

Leadership commitment and safety policy

- Leadership commitment to patient safety.
- National patient safety policy (separately or in some cases an aspect of wider quality improvement policy).

Safety risk management

- Prospective risk management, with risks identified using multiple sources of evidence (e.g. local, national, and international research). Countries had specific standards for high-priority patient safety topics (e.g. patient identity mix-up) as well as general guidelines.

Safety assurance

- Retrospective incident reporting and analysis linked to system for feedback and learning to ensure continuous improvement. Methods for categorising events (e.g. actual harm vs near misses) and their severity (high- and low-) were similar across countries and determined whether and how they were investigated (e.g. central vs local, such as in the Netherlands and New Zealand). Investigation differed (e.g. learning review and restorative practice in New Zealand, root cause analysis in the Netherlands and Australia, see Glossary).
- Monitoring of patient safety performance. Whilst systems for monitoring at the local level were less clear, all countries had a national system.

Safety promotion and culture

- Focus on developing a safety culture (including open communication and blame-free reporting).
- Patients' and families' involvement, both in the development of patient safety policies and safety at the local level.

For peer review only

For peer review only - http://bmjopen.bmj.com/site/about/guidelines.xhtml

Table 3 Comparison of the scope of the SMS or safety approach in each country

	Netherlands	Australia	Canada	Ireland	New Zealand
**Geographical**	National coverage.	National standards; state and territory governments are responsible for their own healthcare systems.	National framework; provincial and territorial governments are responsible for their own healthcare systems.	Standards and patient safety strategy are set at the national level, but implementation is local to allow for adaptation to context.	Transition from regional to national coverage.
**Organisational**	Hospital care.	All healthcare settings.	All healthcare settings.	Health and social care.	All healthcare settings; health and disability care.
**Overlap with quality management/systems**	SMS separate from the quality management and occupational health and safety systems.	Quality and safety are considered together.	Quality and safety are considered together.	Safety is considered part of other systems in the organisation (e.g. quality improvement).	Quality and safety are considered together.
**Extent of accountability for 'other' organisations**	Third party management (outsiders performing activities in hospital) included.	-	-	There should be formalised agreements in place for the provision of services provided externally, including quality and safety.	-

Table 4 Comparison of key features of the SMS or safety approach in each country relating to leadership commitment and safety policy and safety risk management

	Netherlands	Australia	Canada	Ireland	New Zealand
**Leadership commitment and safety policy**	BoD formulates multi-year safety policy and safety plan using SMART-formulated objectives; provides resources (funds, time, expertise, training etc); reviews and evaluates impact and promotes safety culture;	The Standards state that the governing body should provide leadership, set the strategic direction (including a framework with processes to drive improvement), and monitor and review safety and quality.	*"Board members are accountable for safety and for ensuring patient safety concerns and incidents are acted upon"*(p.42);[29] this includes quality and safety goals.	The national standards set out the need for "*A commitment to quality and safety articulated and demonstrated by those governing and leading the service*".(p.72)[33] There should be an identified	Leadership is one of the system drivers in the draft Clinical Governance framework. It enables the health workforce to be active members of the learning organisation by creating a culture of psychological safety and

For peer review only - http://bmjopen.bmj.com/site/about/guidelines.xhtml

	management translates the above into their own area of responsibility and reports to BoD. All employees have responsibility and are encouraged to participate in improving safety. Patient safety is included in the hospital's performance system	The governing body of the organisation should also ensure "*that roles and responsibilities are clearly defined for the governing body, management, clinicians and the workforce*"(p.6)[28] in relation to quality and safety, and that clinicians understand their role in patient safety.	Health leaders should encourage and support health teams to improve patient safety, whilst health workers should take an active role in creating a safe environment.	individual with accountability and responsibility for safety, as well as a commitment to safety from leaders at all levels.<br><br>The national patient safety strategy will "*put in place integrated governance structures with clear accountability for planning, managing and addressing the above patient safety priorities.*"(p.17)[35] i.e. boards, management, staff and relevant quality and safety committees within an organisation should all have defined responsibilities for safety.	trust. Organisations need to share leadership by valuing knowledge and expertise drawn from lived experience of consumer and whānau equally alongside clinical and other knowledge. The National Adverse Events Policy states that health and disability service providers are accountable for ensuring the systems they implement recognise various degrees and types of harm; meet the national expectations for reporting, healing, learning and improving; involve consumers and whanau; and create local process to operationalise the policy.
**Safety risk management[a]**	Prospective risk assessment carried out by multi-disciplinary teams using accepted methods (e.g. failure modes, effects, and criticality analysis, FMECA), provided with sufficient time and resources; regularly reviewed.<br><br>Operational control measures: patient safety is integrated in daily business operations; management of change is an aspect of this.	Providers should identify and documents risks (including external and internal disasters and emergencies); take actions to reduce them; regularly review actions, and report on identified risks to staff and patients.	-	The national patient safety strategy details the need to "*proactively identify areas of risk to patient safety*"(p.15),[35] as do the Standards. Learning should also be taken from where things have gone wrong.<br><br>Governance for risk should be integrated into an organisation's management processes.	In the draft Clinical Governance framework, the focus is on system safety and 'learning together' culture. It suggests using the MMSF and actively seeking organisational data that challenges the sense that 'all is well'. A broad range of data sources are listed including quality monitoring, 'soft intelligence', operational data, health workforce and consumer and whanau experience.

For peer review only - http://bmjopen.bmj.com/site/about/guidelines.xhtml

	Focus on ten priority themes.	The NSQHS Standards (6 of which relate to high-risk areas of patient care) and clinical care standards.		Clinical standards.	Clinical standards.
[a] Including risk related to planned changes					

Table 5 Comparison of key features of the SMS or safety approach in each country relating to safety assurance (including subcategory on incident reporting and investigation) and safety promotion and culture

	**Netherlands**	**Australia**	**Canada**	**Ireland**	**New Zealand**
**Safety assurance**[b]					
Safety performance monitoring and measurement	Management is responsible for monitoring patient safety performance in the hospital and reporting of results (e.g. to BoD) at least annually.	Organisations should have quality improvement systems that identify, monitor and report on safety performance and outcomes	*"Ensure that avoidable death and hospital standardized mortality ratio (HSMR) data is tracked and reported so action can be taken to improve outcomes"*(p.34).[29]	Monitoring against National Standards for Safer Better Healthcare by HIQA, using national indicators where possible.	Monitoring against Te Tāhū Hauora's health quality and safety indicators. The Quality and Safety Measures are a mix of process and outcome measures, and focus on key safety priorities: falls, healthcare associated infections, surgical harms and medication safety.
Continuous improvement	Using a multi-disciplinary approach, improvement measures are implemented and their impact assessed.	Organisations should identify areas for improvement and Implement and monitor improvement strategies.	*"Support an audit and feedback system to ensure that recommendations to improve patient safety are implemented"*(p.33).[29]	Requirement for annual audits, national and local. Learning from both incident reporting and monitoring should be used to improve quality and safety.	Continuous learning from adverse events is based on open reporting, investigation and meaningful analysis. Changes are implemented based on the learning process.
Incident reporting and investigation					
Reporting system	Requires safe, efficient and effective reporting processes and a system using accepted methods and pre-specified incident categories (reporting limited to hospital and to BoD). The information is used to improve safety by	NSQHS Standards require an organisation-wide clinical incident management and investigation system.	Health leaders should enable health teams to "*Report, learn from, and act on patient safety concerns and incidents*" (p.24)[29] by ensuring policies and reporting mechanisms are in place.	The Standards detail the need for "*Arrangements to identify, manage, respond to and report patient-safety incidents in a timely manner*".(p.66)[33]<br><br>Incidents need to be entered on a national system, whether they are	The National Adverse Events policy 2023 requires serious adverse events to be reported to and investigated by the Te Tāhū Hauora. Less serious events are reported and managed locally.

For peer review only - http://bmjopen.bmj.com/site/about/guidelines.xhtml

	determining tasks, powers and responsibilities.		Organisational incident reporting and reporting to national systems for certain incidents.	investigated internally or externally depends on severity of incident.	
Method of investigation	Root cause analysis was recommended as an Investigation method initially.[25] NEN8009 (2018) does not recommend a method.[26]	Investigation method is root cause analysis.	-	Investigation method based on systems analysis.[36]	Investigation method is learning review.
Post-incident support	Staff involved in an incident are offered aftercare.	-	Health leaders and boards should ensure that health teams have access to psychological support.	Support (including psychological) for staff involved in a serious incident.	A theme in The National Adverse events policy 2023 'healing': providing emotional and practical support to affected individuals, families and healthcare workers.
**Safety promotion and culture[c]**					
Presence of safety culture	BoD responsible for promoting safety culture, including safe reporting.<br><br>Open communication in all directions.	Organisations should provide '*timely reports on safety and quality systems and performance*'(p.7)[28] to employees at all levels of the organisation, the public, and other healthcare organisations.	Recent move to create a proactive environment, with a culture and processes that support continuous safety improvement (e.g. safety huddles and other methods of open communication).	Systems for staff communication and engagement within organisations as well as with external agencies (e.g. HIQA) and support for programmes to promote a patient safety culture.	Cultural safety; integration of Maori worldview and safety science.
Training and education	BoD and management are responsible for providing training and monitoring whether staff need further training.	The organisation should provide access to training and monitor participation.	Health teams have a responsibility to undertake training and leaders and boards to provide training.	The Strategy identifies the importance *"designing and delivering safety information and training"* (p.13)[35] for staff.	Staff should be provided with learning opportunities.
Patient participation	Patient participation in drawing up and implementing safety policy	Patient participation in "*healthcare governance planning, design, measurement and evaluation*"(p.19)[28]	Patient participation in patient safety (e.g. through incident reporting and as public representatives in policy discussions).	Organisations should "*develop mechanisms to empower patients to contribute*"(p.11),[35] including to governance	*"Organisational clinical priorities, processes and evaluations are co-designed and developed*

For peer review only - http://bmjopen.bmj.com/site/about/guidelines.xhtml

	and reporting incidents, complaints and claims.			structures and reporting incidents.	*collectively*"(p.11)[47] with patients.
[b] Including accreditation, monitoring and reporting of outcomes as well as systematic implementation of improvement measures and evaluation of their impact, [c] Including patient and family involvement. Abbreviations: BoD – Board of Directors					

For peer review only

For peer review only - http://bmjopen.bmj.com/site/about/guidelines.xhtml

There were, however, differences in the implementation of these common features between countries (e.g. incident investigation as described above and see Table 5). There were also other areas of variation, particularly relating to the scope of the patient safety approach (Table 3). In most cases, patient safety was considered alongside healthcare quality improvement, although in the Netherlands it was separate. Implementation of policies varied from national to regional, and their application to all or specific healthcare settings (e.g. hospital care only in the Netherlands, or all care settings in the other four countries). Additionally, a range of concepts from high-risk industries and safety science (see Glossary) more broadly (e.g. Safety II, human factors, resilience engineering; see Glossary) had influenced the patient safety approaches of different countries.

All countries considered inequalities to some extent by targeting groups particularly at risk of unsafe care (e.g. older people). The most specific guidance was found in Australia, Canada and New Zealand. In these countries groups were identified in terms of social (e.g. those living in deprivation), ethnic (e.g. Māori and Pacific peoples in New Zealand), and protected characteristics (e.g. sexuality), and actions or objectives were specified to achieve outcomes for these groups. Ireland highlighted the need to consider a person's circumstances and background in their healthcare; the PSP in the Netherlands did not provide guidance for different patient groups beyond the need to pay special attention to older people (e.g. within the ten patient safety themes).

It is difficult to directly attribute changes in patient outcomes to specific policy and management changes, especially when different components are introduced incrementally, and often over many years. In relation to our third objective, on the effectiveness, implementation or experience of SMSs or patient safety approaches, we found that only three countries conducted national evaluations of their approach. In the Netherlands, a longitudinal study comparing data from 2008[48] and 2011/12[49] reported a 45% decrease in potentially preventable adverse events over the course of the national safety programme (which required all public hospitals to implement an SMS and ten safety themes). However, a more moderate 30% decrease in potentially preventable adverse events, which was not statistically significant, was found after adjusting for data clustering and changes in the patient mix between measurements.[50] There was also variation in the successful implementation of the ten themes between hospitals and departments, with a number of staff-related, organisational and topic-specific factors identified as potential modifiers.[23] Australia and Ireland both found improvements for certain aspects of patient safety,[27, 51, 52] with some of these in areas in which the Netherlands also saw improvement (e.g. medication safety).[13] Similarly, there were factors which all of these countries identified as facilitating better patient safety such as strong governance or leadership.[13, 27, 51, 52]

4. Discussion

Of the five included countries, only the Netherlands has formally adopted an SMS approach.[13] All SMS components were found in the policies of the other four countries, but not in the form of a single unified system. Across all five countries, there was a change in the patient safety discourse away from a narrow focus on reporting and learning from incidents. Whilst reporting was still considered important, the new approaches to patient safety adopted broader definitions of safety (e.g. including psychological and cultural safety) and harm (e.g. including harm resulting from social inequalities and structural oppression), and emphasised taking a systems perspective to safety, learning from successful practice as well as failures. They highlighted the need for wider involvement in the processes of assessing and creating safety, including patients and families as well as staff.

For peer review only - http://bmjopen.bmj.com/site/about/guidelines.xhtml

Healthcare is often thought to have more complex and unpredictable demands compared to other industries which have adopted an SMS approach, leading to debate over the suitability of SMSs in contexts and organisations in which service delivery is not stable or predictable.[5] The Dutch patient safety programme had some positive impact on patient safety, suggesting an SMS approach is suitable for use in healthcare.[13] However, the evaluation focused on the safety programme as a whole, rather than the SMS specifically, making it difficult to determine whether there are specific components of an SMS that are particularly effective in improving safety. Whilst improvement was seen as part of the programme, this does not mean it is the only way to improve patient safety.[5] The elements of an SMS were present, to some extent, in the healthcare systems of all included countries, but other concepts used by high-risk industries, and from safety science more widely, had influenced the patient safety approach in all countries, with evidence of effectiveness in some cases (e.g. Ireland[51]).

The use of these concepts potentially reflects a new, emerging perspective in which healthcare and high-risk industries are seen as societal and corporate domains that share the same theoretical field – safety science – and can learn from each other, as recognised in documents such as the WHO Global Patient Safety Action Plan 2021-30.[1] This is very different from the idea that, in terms of safety, healthcare is lagging behind and should learn and even copy the high-risk industries' approach to process safety, which has dominated the patient safety discourse.[5, 53] There were considerable differences in the implementation of SMS elements and safety concepts between countries, with patient safety systems reflecting different cultural and organisational realities and local preferences. What works, or makes sense, in one context may not work, or make sense, in another. Comparing and understanding such differences could advance the field of safety science as applied to healthcare and help countries review and improve their own policies and practice. For example, a national review of patient safety in England in 2024 found that, whilst individual healthcare organisations are prioritising common patient safety problems, there is a need for a more focused and coherent approach at the national level.[2] The experiences of a smaller country, such as the Netherlands, which was able to implement a single, highly structured patient safety programme, may be more applicable to the UK context than those of larger, federal countries such as Australia and Canada.

Within this general shift in thinking, different countries had preference for specific concepts and theories from safety science, the most prominent of which were the Measurement and Monitoring of Safety Framework (MMSF) and resilience engineering theory (see Glossary). Regardless of the theoretical underpinnings of a particular patient safety approach, it still needs to be both operationalised and adapted to a particular context. Whilst we focused in this review on how a national organisation can influence or coordinate the implementation of an SMS or safety approach, some policies and most interventions are implemented at the level of service commissioning and/or within health service delivery organisations. For example, the MMSF is a framework and a set of "guiding principles for safety measurement and monitoring"(p.70).[5] It does not provide details on implementation, which remains a challenge, as can be seen in the experience of Canadian healthcare providers of applying the MMSF in their healthcare system.[32] In terms of adaptation, current patient safety discourse recognises that both 'Safety I' (learning from incidents) and 'Safety II' (learning from success) are important and should be part of a modern patient safety approach. Safety II has important implications for the organisation of patient safety, particularly the use of a broader range of safety metrics including outcome measures and resilience indicators as well as adverse events.[54]

However, chosen measures and indicators need to be suitable for the context in which they are being used. As monitoring and evaluation are integral to a systems approach to safety,

For peer review only - http://bmjopen.bmj.com/site/about/guidelines.xhtml

this is a key area for further research. Realist research, to understand the causal mechanisms through which patient safety initiatives work, and contextual factors that influence their successful implementation, could aid the choice of suitable methods of evaluation. Understanding the mechanisms behind different components of patient safety initiatives would also support their adaptation to different contexts. For example, the dominant focus of patient safety approaches in all countries was on hospital care (although all countries apart from the Netherlands included all healthcare settings in their patient safety approaches), but there may be different challenges in other settings such as primary and community care, and for specific patient groups (e.g. patients with learning disabilities), requiring the tailoring of standards.

4.1 Limitations

Although we followed best practice for conducting a systematic review, some limitations should be acknowledged:

- The review is focused on patient safety in selected high-income, English-speaking countries with healthcare systems broadly comparable to that in the UK. Although we conducted scoping searches to decide on our countries of focus, this means that there may be different contexts (e.g. lower income countries) in which SMS are being implemented within the healthcare system.
- Our interpretation is based on analysis of non-evaluative and administrative documents, many of which express aspirations regarding patient safety, rather than being empirically grounded evidence detailing outcomes. We tried to mitigate this limitation by talking to experts from each country and sharing our draft descriptions with them for comment.

5. Conclusions

Only the Dutch patient safety programme was explicitly based on an SMS approach and showed some longitudinal evidence of improving patient safety. Process safety as conceptualised in high-risk industries appeared to have been adapted or had some influence on approaches in the other countries, but this was less systematic and explicit. Approaches to patient safety in all countries also draw on a range of concepts from broader safety science. This suggests a shift from the view that healthcare needs to adopt high-risk industries' approach to safety, and increasing awareness that for initiatives to be successful, they need to be adapted to their context, especially through more collaborative learning between industries and sectors.

Glossary

Human factors is the "*study of the interrelationships between humans, the tools they use, and the environment in which they live and work*"(p.210).[53]

Learning review is a specific approach for investigating incidents where the focus is on collaboration and learning, and the system rather than on the individuals involved.[55]

Patient mix-up Errors regarding a patient's identity or their procedure (e.g. surgery involving the wrong side, wrong site, wrong procedure, or wrong patient).[13]

Patient safety "a framework for organised activities that creates cultures, process, procedures, behaviours, technologies and environments in healthcare that consistently and

For peer review only - http://bmjopen.bmj.com/site/about/guidelines.xhtml

sustainability lower risk, reduce the occurrence of avoidable harm, make errors less likely and reduce impact of harm when it does occur”.[1]

Resilience engineering theory “*looks for ways to enhance the ability of systems to succeed under varying conditions*” i.e. proactively building resilient systems.[5, 56]

Resilient systems/organisations can adjust their functioning before, during, or after events, sustaining required operations under both expected and unexpected conditions.[56]

Restorative practice in the context of the New Zealand patient safety policy *“…is a voluntary, relational process where skilled facilitators support all those affected by an adverse event in a safe and supportive environment. Participants can speak openly about what happened, to understand the human impacts and to clarify responsibility for the actions required for healing and learning.*”[57]

Root cause analysis is a process used to identify the root – or most fundamental -cause of a problem or incident (rather than the first viable cause).[6, 7]

Safety I is an approach to safety in which the focus is on preventing errors and incidents occurring by identifying the causes and responding either when an event occurs or when a risk is deemed unacceptable.[54]

Safety-II is an approach to safety which shifts the focus from what goes wrong to ensuring that things go right (learning from success).[5, 54]

Safety science is considered to be any research, education etc. concerning the science and technology of human safety.[58]

SMART stands for Specific, Measurable, Acceptable, Realistic and Time-bound, in relation to setting objectives.

System safety acknowledges that multiple factors, and the interactions between them, lead to safety, and that the system can be modified to improve safety and resilience.[5]

Systems approach considers multiple levels, i.e. “the interaction between individuals, organisations and sociotechnical system systems” (p.25).[1]

Wrong site surgery A surgical procedure carried out on the wrong side of the body, or on the wrong site on the body.[13]

Acknowledgements

The Exeter HSDR Evidence Synthesis Centre team would like to acknowledge and thank Tracey Herlihey, Matt Fogarty and Jason Cox for their support in developing this review, the experts who took the time to provide us with insight on patient safety in their countries, and our public collaborators for their valuable contributions to our understanding of the topic. We

For peer review only - http://bmjopen.bmj.com/site/about/guidelines.xhtml

would also like to thank Sue Whiffin and Jenny Lowe for administrative support throughout this review.

Funding

This report presents independent research funded by the National Institute for Health Research (NIHR). This review (award NIHR136105) was commissioned by the Health Services and Delivery Research (HS&DR) programmes as part of a series of evidence syntheses under award NIHR130538. For more information, visit https://fundingawards.nihr.ac.uk/award/NIHR135660 and https://fundingawards.nihr.ac.uk/award/NIHR130538.

JTC and AB are also supported by the National Institute for Health Applied Research Collaboration South West Peninsula.

Author contributions

Role	Name
Conceptualisation	Sian de Bell (Equal), Zhivko Zhelev (Equal), Alison Bethel (Equal), Jo Thompson Coon (Equal), Rob Anderson (Equal)
Data Curation	Not applicable
Formal Analysis	Sian de Bell (Equal), Zhivko Zhelev (Equal)
Funding Acquisition	Jo Thompson Coon (Equal), Rob Anderson (Equal)
Investigation	Sian de Bell (Equal), Zhivko Zhelev (Equal), Alison Bethel (Supporting), Maria Clarke (Supporting)
Methodology	Not applicable
Project Administration	Jo Thompson Coon (Equal), Rob Anderson (Equal)
Resources	Not applicable
Software	Not applicable
Supervision	Not applicable
Validation	Not applicable
Visualisation	Zhivko Zhelev (Lead)
Writing – Original Draft	Sian de Bell (lead), Zhivko Zhelev (Supporting)
Writing – Editing and Reviewing	Sian de Bell (Equal), Zhivko Zhelev (Equal), Alison Bethel (Equal), Maria Clarke (Equal), Jo Thompson Coon (Equal), Rob Anderson (Equal) Guarantor: Sian de Bell

Competing interests

Siân de Bell - Disclosure of interest: none declared

Zhivko Zhelev - Disclosure of interest: none declared

Alison Bethel - Disclosure of interest: none declared

Maria Clarke - Disclosure of interest: none declared

Jo Thompson Coon

- Member of the NIHR HTA General Board (2019-2024)
- Member of NIHR PRU Commissioning Panel 2022
- Member of the NIHR RPSC Committee (2024 -)

For peer review only - http://bmjopen.bmj.com/site/about/guidelines.xhtml

Rob Anderson - Disclosure of interest: none declared

Data-sharing statement

This is an evidence synthesis study based on published primary research, it did not generate new data. All data extracted from the included publications, along with links to each publication, can be found in the report or Appendices. Further information can be obtained from the corresponding author.

Ethics statement

This was an evidence review, based on published primary studies, so did not require ethical approval.

Information governance statement

All data used in this paper is taken from published sources: no personal data was included.

References

1. World Health Organisation. *Global patient safety action plan 2021-2030 - Towards eliminating avoidable harm in health care*. 2021. URL: https://iris.who.int/server/api/core/bitstreams/a28c34c0-089c-4f5d-a0b1-5d9c35a3cd67/content (accessed 15 December 2025).
2. Illingworth J, Fernandez Crespo R, Hasegawa K, Leis M, Howitt P, Darzi A. *The National State of Patient Safety 2024: Prioritising improvement efforts in a system under stress*. London: Imperial College London; 2024.
3. Health Services Safety Investigations Body. *Safety management systems - an introduction for healthcare*. 2023. URL: https://www.hssib.org.uk/patient-safety-investigations/safety-management-systems/investigation-report/ (accessed 24 April 2024).
4. Institute for Global Health Innovation. *National state of patient safety 2022: What we know about avoidable harm in England*: Imperial College London; 2022.
5. Vincent C, Burnett S, Carthey J. *The measurement and monitoring of safety*. London: The Health Foundation; 2013.
6. International Nuclear Safety Advisory Group. *Management of operational safety in nuclear power plants (INSAG-13)*. Vienna, Austria: International Nuclear Safety Advisory Group; 1999.
7. International Civil Aviation Organisation (ICAOSMM). *Safety management manual (Doc 9859, 4th Edition)*. Montreal, Canada: International Civil Aviation Organisation; 2018.
8. Thomas M. *A systematic review of the effectiveness of safety management systems. ATSB Transport Safety Report, Cross-modal Research Investigation, XR-2011-0*. Canberra: Australian Transport Safety Bureau; 2012.
9. Li Y, Guldenmund FW. Safety management systems: A broad overview of the literature. *Safety Science* 2018;**103**:94-123. https://doi.org/10.1016/j.ssci.2017.11.016
10. Kapur N, Parand A, Soukup T, Reader T, Sevdalis N. Aviation and healthcare: a comparative review with implications for patient safety. *JRSM Open* 2016;**7**. https://doi.org/10.1177/2054270415616548
11. OGP, IPIECA. *Operating Management System Framework for controlling risk and delivering high performance in the oil and gas industry, OGP Report No 510*. London, UK: OGP & IPIEC; 2014.
12. Macchi L, Pietikäinen E, Reiman T, Heikkilä J, Ruuhilehto K. *Patient safety management: Available models and systems*. Espoo, Finland: VTT Technical Research Centre of Finland; 2011.
13. de Blok C, Koster E, Schilp J, Wagner C. *Implementatie VMS Veiligheidsprogramma. Evaluatieonderzoek in Nederlandse ziekenhuizen*. Utrecht /Amsterdam, Netherlands: NIVEL & EMGO+; 2013.
14. Zhelev Z, de Bell S, Bethel A, Clarke M, Anderson R, Thompson Coon J. The implementation of Safety Management Systems (SMS) in healthcare: a systematic review and international comparison. *Health and Social Care Delivery Research* 2025;**13**. https://doi.org/10.3310/QPLF8546

For peer review only - http://bmjopen.bmj.com/site/about/guidelines.xhtml

15. World Bank. *World Bank country and lending groups*. 2023. URL: https://datahelpdesk.worldbank.org/knowledgebase/articles/906519-world-bank-country-and-lending-groups (accessed 31 January 2024).
16. European Observatory on Health Systems and Policies. *Netherlands*. 2016. URL: https://eurohealthobservatory.who.int/countries/netherlands/ (accessed 26 February 2026).
17. The Commonwealth Fund. *Country profiles - International health care system profiles*. 2020. URL: https://www.commonwealthfund.org/international-health-policy-center/countries (accessed 26 February 2026).
18. Braithwaite J, Zurynski Y, Ludlow K, Holt J, Augustsson H, Campbell M. Towards sustainable healthcare system performance in the 21st century in high-income countries: A protocol for a systematic review of the grey literature. *BMJ Open* 2019;**9**:e025892. https://doi.org/10.1136/bmjopen-2018-025892
19. Petticrew M, Anderson L, Elder R, Grimshaw J, Hopkins D, Hahn R*, et al.* Complex interventions and their implications for systematic reviews: a pragmatic approach. *Journal of Clinical Epidemiology* 2013;**66**:1209-14. https://doi.org/https://doi.org/10.1016/j.jclinepi.2013.06.004
20. Ames HMR, Glenton C, Lewin S, Tamrat T, Akama E, Leon N. Clients' perceptions and experiences of targeted digital communication accessible via mobile devices for reproductive, maternal, newborn, child, and adolescent health: a qualitative evidence synthesis. *Cochrane Database Syst Rev* 2019;**14**:CD013447. https://doi.org/10.1002/14651858.CD013447
21. Dalglish SL, Khalid H, McMahon SA. Document analysis in health policy research: the READ approach. *Health Policy and Planning* 2020;**35**:1424-31. https://doi.org/10.1093/heapol/czaa064
22. Bal R, Wagner C. Chapter 27: Patient safety in Dutch hospitals: How can we explain success? In: Braithwaite J, Mannion R, Matsuyama Y, Shekelle P, Whittaker S, Al-Adawi S, editors. *Health systems improvement across the globe: Success stories from 60 countries.*Boca Raton, FL: CRC Press; 2018:205-12.
23. de Blok C, Koster E, Schilp J, Wagner C. *Implementation of the Dutch national Patient Safety Programme (VMS Veiligheidsprogrammesma)*. Utrecht/Amsterdam, Netherlands: NIVEL & EMGO+; 2013.
24. *Tijd voor Verbinding*. no date. URL: https://www.programmatvv.nl/ (accessed 25 April 2024).
25. Royal Netherlands Standards Institute. *Dutch Technical Agreement (NTA) 8009 'Safety management system for hospitals and institutions that provide hospital care'*. Delft, Netherlands: Royal Netherlands Standards Institute; 2007.
26. Royal Netherlands Standards Institute. *Dutch Technical Agreement (NEN) 8009 'Safety management system for hospitals and institutions that provide hospital care'*. Delft, Netherlands: Royal Netherlands Standards Institute; 2018.
27. Australian Commission on Safety and Quality in Health Care. *The state of patient safety and quality in Australian hospitals 2019*. Sydney, Australia: Australian Commission on Safety and Quality in Health Care; 2019.
28. Australian Commission on Safety and Quality in Health Care. *National safety and quality health service standards. 2nd edition*. Sydney, Australia: Australian Commission on Safety and Quality in Health Care; 2021.
29. Health Standards Organisation, Canadian Patient Safety Institute. *The Candanian quality and patient safety framework for health services*. 2020. URL: https://www.healthcareexcellence.ca/en/resources/canadian-quality-and-patient-safety-framework-for-health-services/ (accessed 24 April 2024).
30. Canadian Patient Safety Institute. *Strengthening commitment for improvement together. A policy framework for patient safety*. Edmonton, AB, Canada: Canadian Patient Safety Institute; 2019.
31. Healthcare Excellence Canada, Patients for Patient Safety Canada. *Rethinking patient safety. A discussion guide for patients, healthcare providers and leaders*. 2023. URL: https://www.healthcareexcellence.ca/media/gx4l3idd/rethinking-patient-safety.pdf (accessed 24 April 2024).

For peer review only - http://bmjopen.bmj.com/site/about/guidelines.xhtml

32. Goldman J, Rotteau L, Flintoft V, Jeffs L, Baker G. Measurement and monitoring of safety framework: a qualitative study of implementation through a Canadian learning collaborative. *BMJ Qual Saf* 2023;**32**:470. https://doi.org/10.1136/bmjqs-2022-015017
33. Health Information and Quality Authority. *National standards for safer better healthcare*. Dublin, Ireland: Health Information and Quality Authority; 2012.
34. Health Information and Quality Authority. *Standards development framework: a principles-based approach*. Dublin, Ireland: Health Information and Quality Authority; 2021.
35. Health Service Executive. *Patient safety strategy 2019-2024*. Dublin, Ireland: Health Service Executive; 2019.
36. Health Service Executive. *Incident management framework*. Dublin, Ireland: Health Service Executive; 2020.
37. Health Information and Quality Authority. *National standards for the conduct of reviews of patient safety incidents*. Dublin, Ireland: Health Information and Quality Authority; 2017.
38. Health Service Executive. *Healthcare audit – quality assurance and verification. Standards.* Dublin, Ireland: Health Service Executive; 2019.
39. Health Service Executive. *HSE Enterprise risk management policy and procedures 2023*. Dublin, Ireland: Health Service Executive; 2023.
40. Byrne D, O'Dowd E, Lydon S, McDermott BR, O'Connor P. *The national simulation strategic guide for the implementation of simulation on clinical sites*. Galway, Ireland: National University of Ireland Galway; 2021. https://doi.org/10.13025/cn0z-bp50
41. Irish Assoication for Simulation. *Irish Association for Simulation*. 2024. URL: https://www.iassim.com/about-us (accessed 08 April 2024).
42. Ministry of Health. *Pae Ora (Healthy Futures) Act 2022*. Wellington, New Zealand: New Zealand Government; 2022.
43. Standards New Zealand. *NZS 8134:2021 Ngā Paerewa Health and disability services standard*. Wellington: Standards New Zealand; 2021.
44. Te Tāhū Hauora Health Quality & Safety Commission. *Te Kaupapa Here hei tuku pūrongo mō ngā 'Aituā ā-Motu': National adverse events reporting policy*. Wellington, Aotearoa New Zealand: Te Tāhū Hauora Health Quality & Safety Commission; 2023.
45. Te Tāhū Hauora Health Quality & Safety Commission. *Clinical governance framework: collaborating for quality (draft, November 2023)*. Wellington, Aotearoa New Zealand: Te Tāhū Hauora Health Quality & Safety Commission; 2023.
46. Te Tāhū Hauora Health Quality & Safety Commission. *National adverse events reporting policy*. 2023).
47. Te Tāhū Hauora Health Quality & Safety Commission. *Clinical governance: guidance for health and disability providers*. Wellington, Aotearoa New Zealand: Te Tāhū Hauora Health Quality & Safety Commission; 2017.
48. Langelaan M, Baines RJ, Broekens MA, Siemerink KM, van de Steeg L, Asscheman H*, et al. Monitor zorggerelateerde schade 2008: Dossieronderzoek in Nederlandse ziekenhuizen*. Utrecht, Amsterdam: NIVEL en EMGO+ Instituut; 2010.
49. Langelaan M, de Bruijne MC, Baines RJ, Broekens MA, Hammink K, Schilp J*, et al. Monitor Zorggerelateerde Schade 2011/2012: Dossieronderzoek in Nederlandse ziekenhuizen* Utrecht, Amsterdam: NIVEL & EMGO+ Instituut; 2013.
50. Baines R, Langelaan M, de Bruijne M, Spreeuwenberg P, Wagner C. How effective are patient safety initiatives? A retrospective patient record review study of changes to patient safety over time. *BMJ Qual Saf* 2015;**24**:561-71. https://doi.org/10.1136/bmjqs-2014-003702
51. Health Information and Quality Authority. *Overview report of five years of HIQA monitoring in Irish public acute hospitals against national standards: 2015–2019*. Dublin, Ireland: Health Information and Quality Authority; 2020.

For peer review only - http://bmjopen.bmj.com/site/about/guidelines.xhtml

For peer review only

52. Australian Commission on Safety and Quality in Health Care. *Creating safer, better health care - the impact of the national safety and quality health service standards*. Sydney, Australia: Australian Commission on Safety and Quality in Health Care; 2018.
53. Institute of Medicine (US) Committee on Quality of Health Care in America. *To err is human: building a safer health system*. Washington, DC: National Academies Press (US); 2000.
54. Hollnagel E, Wears RL, Braithwaite J. *From safety-I to safety-II: A White Paper*: The Resilient Health Care Net: Published simultaneously by the University of Southern Denmark, Denmark, University of Florida, USA, and Macquarie University, Australia; 2015.
55. Te Tāhū Hauora Health Quality & Safety Commission. *Guide to doing a learning review*. Wellington, Aotearoa New Zealand: Te Tāhū Hauora Health Quality & Safety Commission; 2023.
56. Hollnagel E. *Resilience Assessment Grid (RAG)*. 2024. URL: https://erikhollnagel.com/ideas/resilience%20assessment%20grid.html (accessed 15/04/2024).
57. Te Tāhū Hauora Health Quality & Safety Commission. *Mahi haumanu, hohou te rongo. Restorative practice and hohou te rongo*. 2023. URL: https://www.hqsc.govt.nz/our-work/system-safety/healing-learning-and-improving-from-harm-policy/restorative-practice-and-hohou-te-rongo/ (accessed 22 April 2024).
58. Aven T. What is safety science? *Saf Sci* 2014;**67**:15-20. https://doi.org/doi.org/10.1016/j.ssci.2013.07.026

For peer review only

For peer review only - http://bmjopen.bmj.com/site/about/guidelines.xhtml

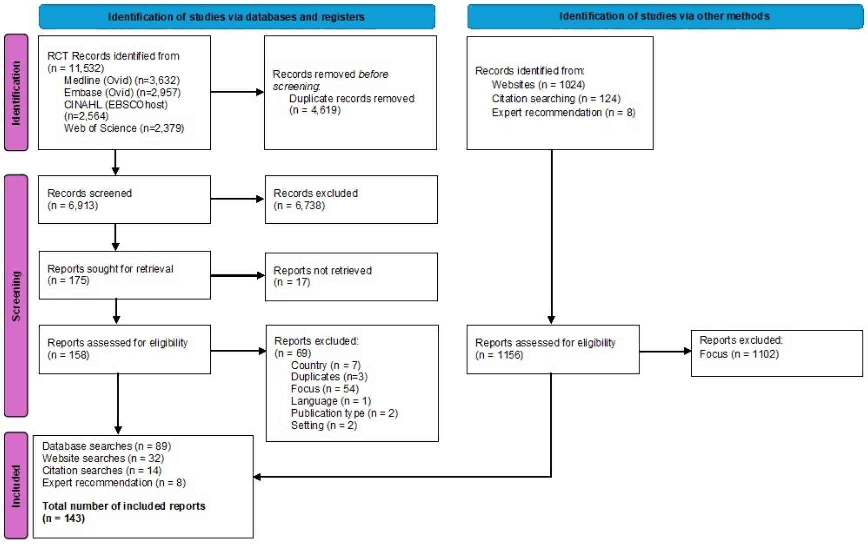

Figure 1 PRISMA flow diagram of the screening process

304x184mm (96 x 96 DPI)

Supplementary material 1: Protocol

University of Exeter

ESMI Evidence Synthesis & Modelling for Health Improvement

The implementation of Safety Management Systems (SMS) in healthcare

Systematic review protocol

Version 5

24 November 2023

For peer review only - http://bmjopen.bmj.com/site/about/guidelines.xhtml

**Produced by**	Exeter HS&DR Evidence Synthesis Centre<br>Department of Health & Community Sciences<br>South Cloisters<br>St Luke's Campus<br>Heavitree Road<br>Exeter<br>EX1 2LU<br>UK
**Authors**	**Zhivko Zhelev** Systematic Reviewer [1]<br>z.zhelev@exeter.ac.uk<br>**Sian de Bell** Systematic Reviewer [1]<br>s.c.de-bell@exeter.ac.uk<br>**Alison Bethel** Information Specialist[1]<br>a.bethel@exeter.ac.uk<br>**Maria Clarke** Information Specialist[1]<br>m.e.clarke2@exeter.ac.uk<br>**Rob Anderson** Senior/Academic Lead[1]<br>r.anderson@exeter.ac.uk<br>**Jo Thompson Coon** Senior/Academic Lead[1]<br>j.thompson-coon@exeter.ac.uk<br><br>[1] University of Exeter
**Correspondence to**	z.zhelev@exeter.ac.uk
**Date completed**	24 November 2023
**Source of funding**	This work was commissioned by the NIHR HSDR programme as a project (project code NIHR136105) within grant number NIHR130538.
**Role of funder/institution in protocol creation**	The aims and scope of this protocol and the planned work were informed by information provided by and discussions with staff in the NHS Patient Safety Team and Department of Health & Social Care

For peer review only - http://bmjopen.bmj.com/site/about/guidelines.xhtml

**Declared competing interests of the authors**	None
**Rider on responsibility for document**	The views and opinions expressed by authors in this publication are those of the authors and do not necessarily reflect those of the NHS, the NIHR, NETSCC, the HSDR programme or the Department of Health & Social Care. Any errors are the responsibility of the authors.
**PROSPERO registration number**	CRD42023487512

For peer review only

For peer review only - http://bmjopen.bmj.com/site/about/guidelines.xhtml

Table of Contents

1 Background 5
1.1 Development of the review 6
1.2 Research questions 7
2 Methods 8
2.1 Approach to evidence synthesis 8
2.2 Identification of studies 9
2.2.1 Searches 9
2.2.2 Inclusion and exclusion criteria 9
2.2.3 Process for applying inclusion criteria 11
2.3 Data extraction 11
2.4 Study quality assessment strategy 12
2.5 Data analysis and presentation 12
3 Stakeholder and patient/public involvement 14
4 Dissemination plans 15
5 References 16

For peer review only

1 Background

There are a number of industries, from aviation to nuclear power, in which safety is a priority. Within these industries, failures or errors could have serious consequences for people, causing loss of life or injury, and for the environment, in terms of environmental damage or harm to equipment or property.(1) Healthcare is now considered to be one of these ‘safety-critical industries’, with safety in this setting defined as:

> “The avoidance, prevention and amelioration of adverse outcomes or injuries stemming from the process of healthcare.”(2)

Examples of safety incidents in healthcare include medication errors, wrong site surgery, and lack of a timely response to deterioration in a patient’s condition.

High-risk industries have improved safety by moving from measuring and responding to specific incidents, to assessing the presence of, and managing, the conditions that lead to hazards.(3) This requires a systems thinking approach, considering interactions and intersections between social and technological elements, which are where safety events tend to occur.(3, 4) Change in some areas may be slow, such as to structural factors e.g. physical infrastructure or the organisation of departments, whereas in other areas of the system, particularly those dependent on more transient ‘mediating’ factors such as morale, teamwork, and individual performance, change may be affected more rapidly.(3)

Within many safety-critical industries, a systems approach to safety has been operationalised in the form of safety management systems (SMS): “an organised approach to managing safety, including the necessary organisational structures, accountabilities, policies and procedures”.(5) These are generally considered to have four key components:

- Policy and objectives,
- Risk management,
- Safety assurance,
- Safety promotion (safety culture).(5)

These are sometimes expanded into six areas, offering further detail on considerations and requirements within the four key components identified above: (i) a safety policy; (ii) organisational arrangements to support safety e.g. supervision and training of staff in implementing the safety policy and processes; (iii) a safety plan, detailing standards and processes for safety, including risk assessment; (iv) measurement of safety performance; (v)

For peer review only - http://bmjopen.bmj.com/site/about/guidelines.xhtml

reviews of safety performance e.g. incident investigation, safety audits; and (vi) feedback loops to improve safety performance.(3)

A systematic review of SMS in three safety-critical industries - aviation, marine and rail – published in 2012 indicated that they led to improvements in safety e.g. reduced accident rates.(6) There is some debate over whether indicators such as accident rates are an appropriate way of measuring the effectiveness of SMS,(7) but this does suggest that they have the potential to improve patient safety in organisations providing healthcare. However, whilst the basic principles of SMS are widely accepted as transferrable,(4, 7) evidence from other industries suggests that the details (e.g. of accountability frameworks) need to be context-specific to be effective.(8) In healthcare, this might involve identifying and improving the management of known patient safety risks.(9) Components of SMS, such as national strategies for improving patient safety,(4) exist in most healthcare systems, and some countries, such as Australia (10) and the Netherlands,(11) are explicitly developing and promoting safety management systems. Their experiences offer opportunities for learning that could support the implementation of the principles of SMS in other healthcare systems, which will have different starting points in terms of the organisation and management of healthcare, and different pre-existing system components.

1.1 Development of the review

This review of research and other evidence has been commissioned by the NIHR HSDR programme to provide evidence and information to NHS England and the NHS Patient Safety Team. It is primarily intended to inform the further development of the NHS England's patient safety policy and practice as laid out in the NHS Patient Safety Strategy 2019(12) and related documents.

The policy questions of interest detailed in the project brief were:

Q1: What are the key attributes of a successful SMS for the NHS in England?

Q2: What are the links between a SMS and a Quality Management System?

Q3: What are the next steps for safety management in the NHS in England?

Evidence is emerging that could begin to answer these questions following the implementation of NHS Patient Safety Strategy 2019.(12) For example, the work of the National Patient Safety team is saving an estimated 160 lives per year (13), and evaluation of the Patient Safety Incident Response Framework (PSIRF) early adopter programme indicates it has led to improved safety cultures and identification of more effective risk reduction strategies in participating organisations.(13) A recent report published by the

For peer review only - http://bmjopen.bmj.com/site/about/guidelines.xhtml

Healthcare Safety Investigation Branch (HSIB)(14) has used evidence on the implementation of SMS in a range of high-risk industries, from aviation to the oil and gas industry, to describe key attributes of SMS at a broad level and make recommendations for the further development of a SMS in the NHS. However, more detailed evidence is needed to assess how a SMS approach might be applicable to the NHS in England.

The review described in this protocol aims to provide information on SMS in healthcare specifically, by investigating how they have been implemented in other countries. This will offer context to inform primary research into the questions raised above, and consideration of how NHS England's current patient safety policy, processes and practice could be extended into a full SMS.

1.2 Research questions

In high-income countries applying the SMS approach (as specified below), what principles have been used to inform healthcare policy, management and practice related to patient and staff safety and what is the evidence supporting their adoption and implementation?

Within this broader question, our specific questions are:

How are the components of SMS reflected in the healthcare policy documents of the above countries, or their regional healthcare systems?

What research (e.g. formal evaluations, or based on routine data) or other relevant evidence (e.g. staff and patient narratives) is available regarding the effectiveness, implementation or experience of SMS within healthcare?

What does existing research and other relevant evidence from beyond the UK tell us about the effectiveness, implementation or experience of SMS within healthcare?

For peer review only - http://bmjopen.bmj.com/site/about/guidelines.xhtml

2 Methods

2.1 Approach to evidence synthesis

As noted above, there is a paucity of empirical evidence on the use of SMS in healthcare. Also, the specific application of such systems in healthcare is likely to depend on the local context, reflecting pre-existing arrangements as well as current priorities and initiatives. To better understand the context of each application and given the expectation of limited empirical evidence, we will conduct a systematic review with a broader scope, including, in addition to research evidence, an analysis of policy documents, training materials and other relevant information (e.g. patients and healthcare professionals' narratives). This will allow better understanding of the logic and process of each implementation; more adequate interpretation of the available empirical evidence; better understanding of the similarities and differences across systems; and the identification of specific barriers, facilitators and challenges that need to be addressed in future research. The review will follow the process detailed below.

Firstly, we will take the four pillars of a SMS as defined by HSIB (14), and compare them to other key SMS literature e.g. documents from safety-critical industries such as aviation (5). In consultation with stakeholders, we will produce an expanded and more detailed framework of the components of a SMS. This will be used to inform data extraction and analysis, to allow us to identify the aspects of SMS that have and have not been adopted by different healthcare systems.

Secondly, we will identify national or regional healthcare systems in which SMS principles have been used to inform policy and practice related to patient and staff safety. For pragmatic reasons, we will focus first on high-income English-speaking countries and, if necessary and feasible, include other countries after discussion with stakeholders. If key policy documents do not state explicitly that a SMS approach was used, then we will contact relevant organisations to enquire about this. Only healthcare systems where such a link and commitment are explicitly stated will be included in the review.

Thirdly, we will identify relevant policy documents, research and other relevant information (e.g. patient stories) from the organisations' websites and by focused searches of electronic databases and other sources. Each organisation will be contacted to ascertain that all relevant information has been captured and request additional data that may have been missed or is not available online.

Fourthly, we will develop a data extraction form organised around the core principles of SMS (as defined in the background) and the different types of evidence we are including: policy

For peer review only - http://bmjopen.bmj.com/site/about/guidelines.xhtml

documents, research and other evidence. If possible, data will be extracted verbatim or, if too long, summarised with reference to its location in the source document. Rather than just depicting a snapshot of the *status quo*, as much as possible, the information gathered will attempt to distinguish the sequence and timings of the adoption and application of different core principles of SMS, or of the main strategies and processes to implement and embed them in a particular country or region/province. This therefore aims to shed light on which principles and systems/processes may have pre-existed the formal policy commitment to adopting a SMS approach, which were seen as most critical for expanding the scope and depth of the SMS, and interactions between the different components of SMS.

Finally, information on the implementation of SMS in the eligible healthcare systems will be synthesised narratively and summarised in tables and graphs. Common themes across different healthcare systems will be identified, evidence gaps will be highlighted, and recommendations on how to address those will be made.

2.2 Identification of studies

2.2.1 Searches

The main method for identifying relevant policy documents, research and other evidence will be through searching the websites of, and direct contact with, relevant organisations (e.g. Australian Commission on Safety and Quality in Healthcare). Website searching will be carried out either by browsing publication lists or using site search functionality. Search parameters (e.g. keywords, publication date) will be pre-specified and the search process will be documented (e.g. number of hits, number of potentially relevant titles) to allow transparency and reproducibility.

The above will be complemented by focused database searches using search strategies developed by an information specialist (AB) in consultation with the review team and the commissioning group. The database search strategies will use both controlled vocabulary when available and relevant (e.g. MeSH in MEDLINE) and free-text searching. Search terms will be partly derived from the literature and from stakeholders and experts' input. Searches will be limited to English-language texts published after a pre-specified date (to be agreed with stakeholders and experts). The reference lists of all relevant documents will be hand-searched for additional titles.

2.2.2 Inclusion and exclusion criteria

The following overarching inclusion and exclusion criteria will be applied:

For peer review only - http://bmjopen.bmj.com/site/about/guidelines.xhtml

- **Safety management system:** Safety management systems are defined by the following key elements:
    - policy and objectives
    - risk management
    - safety assurance
    - safety promotion (safety culture).

  Texts will be excluded if they refer to different concepts or models without direct link to the above definition of a safety management system.

- **Application of SMS** in the healthcare system of a high-income country (as defined by the World Bank). For pragmatic reasons, we will focus on English-speaking countries; if necessary and feasible, and after discussion with our policy costumer, we may extend the scope to include other, selected high-income countries.

- **Type of evidence:** we will include three distinct types of evidence:
    - Relevant policy documents, including documents related to the implementation of SMS, such as staff training and patient-facing materials
    - Published or unpublished research / evaluations (quantitative, qualitative or mixed methods),
    - Other evidence, e.g. patient and staff experience or opinions published on the organisations' websites.

- **Provenance of evidence:** As mentioned earlier, for pragmatic reasons we will focus first on high-income English-speaking countries as evidence from such healthcare systems is more likely to be relevant to the UK and all relevant policy documents and research will be available in English. Therefore, evidence related to the implementation of SMS in the healthcare of the following countries will be considered first: Ireland, Australia, New Zealand, Canada and the Netherlands. The list will be revised / amended once the initial searches have been completed and the volume, type and quality of evidence is assessed; or, it has been decided in discussion with experts and stakeholders that including a specific country or expanding the list is desirable and feasible.

For peer review only - http://bmjopen.bmj.com/site/about/guidelines.xhtml

- **Language:** we will include texts available in English; if the scope of the review is extended (see above) we may decide to include texts in other languages, if necessary and feasible.

2.2.3 Process for applying the inclusion criteria

A small sample (n ~ 10) of potentially relevant documents from the three types of evidence will be used to calibrate the selection process. Each document will be reviewed by multiple reviewers. Decisions will be compared and discussed in a group meeting to ensure consistent application of criteria. If necessary, definitions will be updated and explanatory notes added to aid the process.

The full text of all potentially relevant documents identified through the website searches and direct contact with the healthcare organisations will be retrieved and checked for relevance against the inclusion criteria independently by two reviewers.

The results from the database searches will be screened following the standard process. Two reviewers will independently apply the inclusion and exclusion criteria to the title and abstract of each identified citation. We will obtain the full text of papers where either reviewer judges it to be a potential inclusion. Two reviewers will assess the full text of each record independently for inclusion, with disagreements settled through discussion or by a third reviewer. The full texts of all potentially relevant publications identified from the website searches will be obtained and screened as per the above process.

The study selection process will be detailed using a PRISMA-style flowchart, with a reason reported for exclusion of each record assessed at full text.

2.3 Data extraction

A data extraction form will be developed and piloted independently by two reviewers on a small sample of included documents. Data will be extracted by one reviewer and checked by a second reviewer. Disagreements will be resolved through discussion.

The following data will be extracted:

- Country
- Healthcare organisation (purpose and remit, website)
- Type of document (policy, research, anecdotal evidence)
- Aims

For peer review only - http://bmjopen.bmj.com/site/about/guidelines.xhtml

- Relation to SMS (e.g. explicitly stated or implied, description)
- Information regarding specific SMS components:
    - Policy and objectives
    - Risk management
    - Safety assurance
    - Safety promotion (safety culture).
- Information regarding implementation and/or impact
- **Research:** research design, aim, methods, results, conclusions
- **Other evidence:** type of document, informant (patient, healthcare professional, policy maker), perspective (relationship to the programme), main points.

The NIHR-INCLUDE guidelines (15) were used to reflect on Equality, Diversity and Inclusion (EDI) whilst designing the protocol. EDI is important to consider in relation to the topic of safety management systems, as there may be differences in safety incidents between social and demographic groups and resulting inequities in health outcomes. However, it is likely this information will be difficult to ascertain from the types of evidence e.g. policy documents, we will be including in the review. We will consider PROGRESS-Plus characteristics during the process of data extraction and analysis (16, 17) and, where possible, use this information to inform the findings and recommendations of the review.

2.4 Study quality assessment strategy

The methodological quality of all research studies will be assessed using validated quality assessment tools according to the specific research design. Quality will be assessed by two reviewers independently and disagreements resolved through discussion or arbitration. No quality appraisal will be carried out on policy documents or non-research evidence.

2.5 Data analysis and presentation

The type of information identified for each healthcare system will be summarised and the following information will be reported:

- The way in which the SMS approach has informed patient and staff safety policy and practice.
- Information related to each of the SMS components (see above).

For peer review only - http://bmjopen.bmj.com/site/about/guidelines.xhtml

- Information related to the process and timing of implementing SMS (e.g. steps, processes etc.).
- Evidence related to the effectiveness / impact and implementation of SMS.
- Stakeholders' perceptions and experience.

Comparisons across healthcare systems will be carried out to identify similarities and differences, including barriers, facilitators and challenges; the volume, type and quality of the available evidence and any evidence gaps that need to be addressed in future research. The results will be summarised in tables and diagrams and presented narratively, considering their relevance to the application of SMS in the NHS of England.

For peer review only

For peer review only - http://bmjopen.bmj.com/site/about/guidelines.xhtml

3 Stakeholder and patient/public involvement and engagement

The following stakeholders will be involved:

- NHS England's Patient Safety Team and the Department of Health and Social Care (as commissioners of the work)
- Topic experts (including from other countries and other industries, if possible)
- Any other relevant Patient Safety organisations (NHS or external) e.g. local patient safety teams

Due to the focus of the review, we will consult Public and Patient Involvement and Engagement representatives who have some knowledge of the topic e.g. members of local patient safety teams, or PPIE representatives who have worked with the National Patient Safety Team.

We will consult stakeholders throughout the review process, to discuss topics such as the focus of the review, analysis, and findings. Meetings will be arranged to suit the project progress and stakeholder availability. We will also ask for their input on dissemination plans and materials as described below.

For peer review only

4 Dissemination plans

We will produce a report on the implementation of safety management systems in healthcare in high-income countries, available as an open access in the NIHR Journals Library.

Further materials for dissemination will be finalised after discussion with the project's advisory group. These are likely to include:

- an evidence briefing, giving a plain language summary of the report and its findings (primarily aimed at policy makers and health service managers);
- an article in an academic journal identified as being relevant to stakeholders for this review; and
- presentations at key national and regional meetings.

Outputs will be disseminated via the Exeter HSDR Evidence Synthesis Centre webpage and social media. Additional material may be produced to promote them, such as a blog post based on the evidence briefing and report.

For peer review only

For peer review only - http://bmjopen.bmj.com/site/about/guidelines.xhtml

5 References

1. Saunders F. Safety-critical industries: definitions, tensions and tradeoffs 2015 [Available from: http://fionasaunders.co.uk/safety-critical-industries-definitions-tensions-and-tradeoffs/#:~:text=Combining%20these%20two%20definitions%2C%20a%20safety-critical%20industry%20can,environmental%20damage%2C%20or%20harm%20to%20plant%20or%20property.

2. Vincent C. Patient safety. 2nd edition ed. Chichester: John Wiley and Sons; 2010.

3. Vincent C, Burnett S, Carthey J. The measurement and monitoring of safety. London: The Health Foundation; 2013.

4. Macchi L, Pietikäinen E, Reiman T, Heikkilä J, Ruuhilehto K. Patient safety management: Available models and systems. VTT; 2011.

5. International Civil Aviation Organisation Safety Management Manual (ICAOSMM). 2nd ed. Doc 9859, Safety Management Manual (SMM) 2009 [Available from: www2.icao.int/en/ism/Guidance%20Materials/DOC_9859_FULL_EN.pdf.

6. Thomas M. A systematic review of the effectiveness of safety management systems. ATSB Transport Safety Report, Cross-modal Research Investigation, XR-2011-0. Canberra: Australian Transport Safety Bureau; 2012.

7. Li Y, Guldenmund FW. Safety management systems: A broad overview of the literature. Safety Science. 2018;103:94-123.

8. Kapur N, Parand A, Soukup T, Reader T, Sevdalis N. Aviation and healthcare: a comparative review with implications for patient safety. JRSM Open. 2016;7.

9. HSIB. A thematic analysis of HSIB's first 22 national investigations. Healthcare Safety Investigation Branch I2020/016; 2021.

10. Australian Commission on Safety and Quality in Health Care. The state of patient safety and quality in Australian hospitals 2019. Sydney: Australian Commission on Safety and Quality in Health Care; 2019.

11. Bolk M. Final report: Embedding patient safety in education & training. VMS Safety Program; 2013.

12. NHS England. The NHS Patient Safety Strategy no date [Available from: https://www.england.nhs.uk/patient-safety/the-nhs-patient-safety-strategy/#key-docs.

13. NHS England. NHS patient safety strategy – progress so far no date [Available from: https://www.england.nhs.uk/patient-safety/the-nhs-patient-safety-strategy/nhs-patient-safety-strategy-progress-so-far/.

14. HSIB. Safety management systems - an introduction for healthcare. 2023.

15. National Institute for Health Research. Improving inclusion of under-served groups in clinical research: Guidance from the NIHR-INCLUDE project. UK: National Institute for Health Research; 2020.

For peer review only - http://bmjopen.bmj.com/site/about/guidelines.xhtml

16. O'Neill J, Tabish H, Welch V, Petticrew M, Pottie K, Clarke M, et al. Applying an equity lens to interventions: using PROGRESS ensures consideration of socially stratifying factors to illuminate inequities in health. J Clin Epidemiol. 2014;67:56-64.

17. Cochrane Methods Equity. PROGRESS-Plus no date [Available from: https://methods.cochrane.org/equity/projects/evidence-equity/progress-plus.

For peer review only

For peer review only - http://bmjopen.bmj.com/site/about/guidelines.xhtml

Supplementary material 2: Inclusion criteria

	Include	Exclude
**Population**	Populations with any health need.	-
**Initiative**	Any use of an SMS approach in a healthcare setting, with an SMS being defined by four key components:<br>• policy and objectives<br>• risk management<br>• safety assurance<br>• safety promotion (safety culture).<br><br>Where it is not clear whether a country is using an SMS approach, we will include studies if they relate to one or more components of an SMS.	Studies of safety where this is not linked to an SMS component.<br>Studies of occupational health and safety.
**Setting**	Any healthcare setting:<br>- Secondary care,<br>- Primary care.	Non-healthcare settings:<br>- Care homes,<br>- Home care.
**Country**	Australia, Canada, Ireland, the Netherlands, New Zealand.<br><br>Studies which included other countries were included if the results for these countries were reported separately.	Any other country.
**Language**	English or Dutch.	Any other language.

For peer review only - http://bmjopen.bmj.com/site/about/guidelines.xhtml

	Include	Exclude
**Type of document**	• Relevant policy documents, including documents related to the implementation of SMS, such as staff training and patient-facing materials<br>• Published or unpublished research / evaluations (quantitative, qualitative or mixed methods),<br>• Other evidence (e.g. patient and staff experience or opinions published on the organisations' websites).	Theoretical

For peer review only

For peer review only - http://bmjopen.bmj.com/site/about/guidelines.xhtml

Supplementary material 3: Excluded and thin studies

Excluded papers from database searching (n=68)

Excluded on country (n=7)

Ma E, Wei W, Ho C. An environmental scan of patient safety reporting and learning systems in community healthcare for multi- disciplinary teams. *Can J Hosp Pharm*. 2022;**75**(2):135.

Coffey M, Marino M, Lyren A, Purcell D, Hoffman JM, Brilli R, et al. Association between hospital-acquired harm outcomes and membership in a national patient safety collaborative. *JAMA Pediatrics*. 2022;**176**(9):924-32.

Kristensen S, Hammer A, Bartels P, Sunol R, Groene O, Thompson CA, et al. Quality management and perceptions of teamwork and safety climate in European hospitals. *Int J Qual Health Care*. 2015;**27**(6):499-506.

Holmstrom AR, Laaksonen R, Airaksinen M. How to make medication error reporting systems work - Factors associated with their successful development and implementation. *Health Policy*. 2015;**119**(8):1046-54.

Windsor JA, Maxim S. Patient safety in medicine: Are surgeons ready for checklists? *ANZ Journal of Surgery*. 2010;**80**(1-2):3-5.

Sunol R, Vallejo P, Groene O, Escaramis G, Thompson A, Kutryba B, et al. Implementation of patient safety strategies in European hospitals. *Qual Saf Health Care. 2009*;**18**(Suppl 1):i57-61.

Shaw C, Kutryba B, Crisp H, Vallejo P, Sunol R. Do European hospitals have quality and safety governance systems and structures in place? *Qual Saf Health Care*. 2009;**18**(Suppl 1):i51-6.

Excluded on focus (n=54)

Ong N, Lucien A, Long JC, Weise J, Walton M, Burgess A. What do healthcare staff think about the quality and safety of care provided to children and young people with an intellectual disability? A qualitative study using the framework method of analysis. *BMJ Open*. 2023;**13**(7):e071494.

Kaud Y, McKeon D, Lydon S, O'Connor P. Measuring and monitoring patient safety in hospitals in the Republic of Ireland. *Irish J Med Sci*. 2023;1**92:**2581–259.

For peer review only - http://bmjopen.bmj.com/site/about/guidelines.xhtml

For peer review only

Cords CI, van Baar ME, Nieuwenhuis MK, Pijpe A, van der Vlies CH. Reliability and validity of a frailty assessment tool in specialized burn care, a retrospective multicentre cohort study. *Burns*. 2023;**49**(7):1621-31.

Terry D, Kim J-a, Gilbert J, Jang S, Nguyen H. "Thank You for Listening": An Exploratory Study Regarding the Lived Experience and Perception of Medical Errors Among Those Who Receive Care. *International Journal of Health Services*. 2022;**52**(2):292-302.

Arnold A, Ward I, Gandhidasan S. Incident review in radiation oncology. *J Med Imaging Radiat Oncol*. 2022;**66**(2):291-8.

Malik RF, Buljac-Samardzic M, Amajjar I, Hilders C, Scheele F. Open organisational culture: what does it entail? Healthcare stakeholders reaching consensus by means of a Delphi technique. *BMJ Open*. 2021;**11**(9):e045515.

Connell CJ, Cooper S, Endacott R. Measuring the safety climate in an Australian emergency department. *Int Emerg Nurs*. 2021;**58**:101048.

Adie K, Fois RA, McLachlan AJ, Walpola RL, Chen TF. The nature, severity and causes of medication incidents from an Australian community pharmacy incident reporting system: The QUMwatch study. *Br J Clin Pharmacol*. 2021;**87**(12):4809-22.

Wright KM, Bonser M. The Essential Steps of Medication Administration Practices Project medication administration improvement practices among acute inpatients in a tertiary hospital: a best practice implementation project. *International Journal of Evidence-Based Healthcare*. 2020;**18**(4):408-19.

Floyd E, Hodgkins R, Naylon L, Noon M, Sirotti L, Monaro S. The costs and benefits of pressure injury point prevalence auditing. *Wound Practice & Research*. 2020;**28**(2):66-74.

Emond Y, Wolff AP, Peters YAS, Bloo GJA, Westert GP, Damen J, et al. Reducing work pressure and IT problems and facilitating IT integration and audit & feedback help adherence to perioperative safety guidelines: a survey among 95 perioperative professionals. *Implement*. 2020;**1**:49.

Clay-Williams R, Taylor N, Winata T, Ting HP, Arnolda G, Braithwaite J. Organization quality systems and department-level strategies: refinement of the Deepening our Understanding in Quality in Australia (DUQuA) organization and department-level scales. *Int J Qual Health Care*. 2020;**32**(Supplement_1):22-34.

Braithwaite J, Taylor N, Clay-Williams R, Ting HP, Arnolda G. Conclusion: the road ahead: where should we go now to improve healthcare quality in acute settings? *Int J Qual Health Care*. 2020;**32**(Supplement_1):99-103.

Ward M, Ni She E, De Brun A, Korpos C, Hamza M, Burke E, et al. The co-design, implementation and evaluation of a serious board game 'PlayDecide patient safety' to educate junior doctors about patient safety and the importance of reporting safety concerns. *BMC Med Educ*. 2019;**19**(1):232.

For peer review only

For peer review only - http://bmjopen.bmj.com/site/about/guidelines.xhtml

McHugh S, Droog E, Foley C, Boyce M, Healy O, Browne JP. Understanding the impetus for major systems change: A multiple case study of decisions and non-decisions to reconfigure emergency and urgent care services. *Health Policy*. 2019;**123**(8):728-36.

Liao E, Eisenberg N, Kaushal A, Montbriand J, Tan KT, Roche-Nagle G. Utility of the Vascular Quality Initiative in improving quality of care in Canadian patients undergoing vascular surgery. *Can J Surg*. 2019;**62**(1):66-9.

Eljiz K, Greenfield D, Derrett A, Radmore S. Health system redesign: Changing thoughts, values, and behaviours for the co-production of a safety culture. *Int J Health Plann Manage*. 2019;**34**(4):1477-84.

Veitch D. One province, one healthcare system: A decade of healthcare transformation in Alberta. *Healthc Manage Forum*. 2018;**31**(5):167-71.

Sinclair JE, Austin MA, Bourque C, Kortko J, Maloney J, Dionne R, et al. Barriers to self-reporting patient safety incidents by paramedics: A mixed methods study. *Prehospital Emergency Care*. 2018;**22**(6):762-72.

Khalil H, Lee S. Medication safety challenges in primary care: Nurses' perspective. *Journal of Clinical Nursing*. 2018;**27**(9-10):2072-82.

Debono D, Taylor N, Lipworth W, Greenfield D, Travaglia J, Black D, et al. Applying the Theoretical Domains Framework to identify barriers and targeted interventions to enhance nurses' use of electronic medication management systems in two Australian hospitals. *Implement Sci*. 2017;**12**(1):42.

Davies JM, Delaney G. Can the Aviation Industry be Useful in Teaching Oncology about Safety? *Clin Oncol*. 2017;**29**(10):669-75.

Blijleven V, Koelemeijer K, Wetzels M, Jaspers M. Workarounds emerging from electronic health record system usage: Consequences for patient safety, effectiveness of care, and efficiency of care. *JMIR Hum Factors*. 2017;**4**(4):e27.

Wagner C, Merten H, Zwaan L, Lubberding S, Timmermans D, Smits M. Unit-based incident reporting and root cause analysis: variation at three hospital unit types. *BMJ Open*. 2016;**6**(6):e011277.

Ng J, Andrew P, Crawley M, Pevreal W, Peach J. Assessing a hospital medication system for patient safety: findings and lessons learnt from trialling an Australian modified tool at Waitemata District Health Board. *N Z Med J*. 2016;**129**(1430):63-77.

Gill FJ, Leslie GD, Marshall AP. Family initiated escalation of care for the deteriorating patient in hospital: Family centred care or just "box ticking". *Aust Crit Care*. 2016;**29**(4):195-200.

Yates M, Reddy M, Machumpurath B, Phelps G, Hampson SA. Modification of the National Inpatient Medication Chart improves venous thromboembolism prophylaxis rates in high-risk medical patients. *Intern Med J*. 2014;**44**(2):190-4.

For peer review only - http://bmjopen.bmj.com/site/about/guidelines.xhtml

Wong KS, Ryan DP, Liu BA. A system-wide analysis using a senior-friendly hospital framework identifies current practices and opportunities for improvement in the care of hospitalized older adults. *J Am Geriatr Soc*. 2014;**62**(11):2163-70.

Teare GF. Measurement of quality and safety in healthcare: the past decade and the next. *Healthc Q*. 2014;**17 Spec No**:45-50.

Russell L, Dawda P. Lessons for the Australian healthcare system from the Berwick report. *Aust Health Rev*. 2014;**38**(1):106-8.

Berg M, Black G. A Canadian perspective on clinical governance. *Clinical Governance: An International Journal*. 2014;**19**(4):314-21.

van Beuzekom M, Boer F, Akerboom S, Dahan A. Perception of patient safety differs by clinical area and discipline. *Br J Anaesth*. 2013;**110**(1):107-14.

Levi M. Towards a hospital-wide integrated system for quality and safety of health care. Neth J Med. 2013;**71**(1):2-3.

Marsden J, van Dijk M, Doris P, Krause C, Cochrane D. Improving care for British Columbians: the critical role of physician engagement. *Healthc Q*. 2012;**15 Spec No**:51-5.

Krause C, Cochrane D. BC Patient Safety & Quality Council: using network and social movement theory to improve healthcare. *Healthc Manage Forum*. 2012;**25**(4):181-4.

Ilan R, Squires M, Panopoulos C, Day A. Increasing patient safety event reporting in 2 intensive care units: a prospective interventional study. *J Crit Care*. 2011;**26**(4):431.e11-8.

Green E, White R, Janes K, Fields A, Easty A. Courage, collaboration, complexity and chemotherapy safety: the view from the sharp end. *Canadian Oncology Nursing Journal*. 2011;**21**(2):81-90.

Duckers ML, Wagner C, Vos L, Groenewegen PP. Understanding organisational development, sustainability, and diffusion of innovations within hospitals participating in a multilevel quality collaborative. *Implement Sci*. 2011;**6**:18.

van Gaal BG, Schoonhoven L, Hulscher ME, Mintjes JA, Borm GF, Koopmans RT, et al. The design of the SAFE or SORRY? study: a cluster randomised trial on the development and testing of an evidence based inpatient safety program for the prevention of adverse events. *BMC Health Serv Res*. 2009;**9**:58.

Relihan E, Silke B, O'Grady F. Internally-developed electronic reporting system for medication errors. *Ir Med J*. 2009;**102**(7):223-4.

For peer review only - http://bmjopen.bmj.com/site/about/guidelines.xhtml

Michel J, Nghiem HD, Jackson TJ. Using ICD-10-AM codes to characterise hospital-acquired complications. *Health Inf Manag*. 2009;**38**(3):18-25.

Neudorf K, Dyck N, Scott D, Davidson Dick D. Nursing education: a catalyst for the patient safety movement. *Healthc Q*. 2008;**11**(3 Spec No.):35-9.

Makeham MA, Saltman DC, Kidd MR. Lessons from the TAPS study--recall and reminder systems. *Aust Fam Physician*. 2008;**37**(11):923-4.

Makeham MA, Cooper C, Kidd MR. Lessons from the TAPS study - message handling and appointment systems. *Aust Fam Physician*. 2008;**37**(6):438-9.

Duckett S, Daniels S, Kamp M, Stockwell A, Walker G, Ward M. Pay for performance in Australia: Queensland's new Clinical Practice Improvement Payment. *Journal of Health Services Research and Policy*. 2008;**13**(3):174-7.

Booth BJ, Snowdon T, Harris MF, Tomlins R. Safety and quality in primary care: The view from general practice. *Australian Journal of Primary Health*. 2008;**14**(2):19-27.

David U. Medication safety... Your call to make a difference. *Alta RN*. 2007;**63**(8):10-1.

Runciman WB, Williamson JA, Deakin A, Benveniste KA, Bannon K, Hibbert PD. An integrated framework for safety, quality and risk management: an information and incident management system based on a universal patient safety classification. *Qual Saf Health Care*. 2006;**15**(Suppl 1):i82-90.

Greenberg A, Kramer S, Welch V, O'Sullivan E, Hall S. Cancer Care Ontario's computerized physician order entry system: a province-wide patient safety innovation. *Healthc Q*. 2006;**9 Spec No**:108-13.

Yassi A, Hancock T. Patient safety--worker safety: building a culture of safety to improve healthcare worker and patient well-being. *Healthc Q*. 2005;**8 Spec No**:32-8.

Greenall J, U D, Lam R. An effective tool to enhance a culture of patient safety and assess the risks of medication use systems. *Healthc Q*. 2005;**8 Spec No**:53-8.

Court D. Quest for patient safety in a challenging environment. *Aust N Z J Obstet Gynaecol*. 2003;**43**(2):97-100.

Nicklin W, McVeety JE. Canadian nurses' perceptions of patient safety in hospitals. *Can J Nurs Leadersh*. 2002;**15**(3):11-21.

For peer review only - http://bmjopen.bmj.com/site/about/guidelines.xhtml

For peer review only

Baldwin I, Beckman U, Shaw L, Morrison A. Australian Incident Monitoring Study in intensive care: local unit review meetings and report management. *Anaesth Intensive Care*. 1998;**26**(3):294-7.

Excluded on language (n=1)

Broeke RT, Lammers R, Ackerman E. The safety management system for medication contributes to a 'safe culture' in the hospital: The pharmacist participates in the medical round at the nursing department. *Pharmaceutisch Weekblad*. 2008;**143**(17):30-2.

Excluded on publication type (n=2)

Hanskamp-Sebregts M, Zegers M, Boeijen W, Westert GP, van Gurp PJ, Wollersheim H. Effects of auditing patient safety in hospital care: design of a mixed-method evaluation. BMC Health Serv Res. 2013;13:226.

Barker A, Mengersen K, Morton A. What is the value of hospital mortality indicators, and are there ways to do better? Aust Health Rev. 2012;36(4):374-7.

Excluded on setting (n=2)

Masotti P, Green M, McColl MA. Adverse events in community care: implications for practice, policy and research. *Healthc Q*. 2009;**12**(1):69-76.

Lang A, Edwards N, Fleiszer A. Safety in home care: a broadened perspective of patient safety. *Int J Qual Health Care*. 2008;**20**(2):130-5.

Update search

Excluded on focus (n=2)

Lam JYJ, Barras M, Scott IA, Abdel-Hafez A, Snoswell C, Gordon E, Morris C, Long D, Wang A, Falconer N. Impact evaluation of the modified adverse inpatient medication event (AIME-Frail) model in hospitalised adults. *Res Soc Ad Pharm.* 2025;**21**:687-696.

McLennan C, Sherrington C, Naganathan V, Tilden W, Richards B, McVeigh T, Hallahan A, Nayak V, Jennings M, Hassett L, Haynes A. Supported implementation of tailored multicomponent fall prevention interventions in hospital: a feasibility study. *BMJ Open Qual.* 2025:**14:**e003313.

For peer review only

List of 'thin' studies

Australia

Abou Elnour A, Hernan AL, Ford D, Clark S, Fuller J, Johnson JK, et al. Surveyors' perceptions of the impact of accreditation on patient safety in general practice. *Med J Aust*. 2014;**201**(3):S56-S9. https://dx.doi.org/10.5694/mja14.00198

Adamson L, Beldham-Collins R, Sykes J, Thwaites D. Evaluating incident learning systems and safety culture in two radiation oncology departments. *J Med Radiat Sci*. 2022;**69**(2):208-17. https://dx.doi.org/10.1002/jmrs.563

Adie K, Fois RA, McLachlan AJ, Chen TF. Medication incident recovery and prevention utilising an Australian community pharmacy incident reporting system: the QUMwatch study. *Eur J Clin Pharmacol*. 2021;**77**(9):1381-95. https://dx.doi.org/10.1007/s00228-020-03075-9

Avery MJ, Cripps AW, Rogers GD. Health boards' governance of quality and risk: quality improvement agenda for the board. *Int J Health Gov*. 2021;**26**(3):292-306. https://dx.doi.org/10.1108/IJHG-01-2021-0006

Braithwaite J, Westbrook MT, Mallock NA, Travaglia JF, Iedema RA. Experiences of health professionals who conducted root cause analyses after undergoing a safety improvement programme. *Qual Saf Health Care*. 2006;**15**(6):393-9. https://dx.doi.org/10.1136/qshc.2005.017525

Braithwaite J, Westbrook MT, Travaglia JF, Iedema R, Mallock NA, Long D, et al. Are health systems changing in support of patient safety? A multi-methods evaluation of education, attitudes and practice. *Int J Health Care Qual Assur*. 2007;**20**(7):585-601. https://dx.doi.org/10.1108/09526860710822725

Braithwaite J, Westbrook M, Travaglia J. Attitudes toward the large-scale implementation of an incident reporting system. *Int J Qual Health Care*. 2008;**20**(3):184-91. https://dx.doi.org/10.1093/intqhc/mzn004

Brand C, Ibrahim J, Bain C, Jones C, King B. Engineering a safe landing: engaging medical practitioners in a systems approach to patient safety. *Intern Med J*. 2007;**37**(5):295-302. https://dx.doi.org/10.1111/j.1445-5994.2007.01310.x

Canaway R, Bismark M, Dunt D, Kelaher M. Medical directors' perspectives on strengthening hospital quality and safety. *J Health Organ Manag*. 2017;**31**(7-8):696-712. https://dx.doi.org/10.1108/JHOM-05-2017-0109

Canaway R, Bismark M, Dunt D, Kelaher M. Public reporting of hospital performance data: views of senior medical directors in Victoria, Australia. *Aust Health Rev*. 2018;**42**(5):591-9. https://dx.doi.org/10.1071/AH17120

For peer review only - http://bmjopen.bmj.com/site/about/guidelines.xhtml

Clay-Williams R, Taylor N, Ting HP, Arnolda G, Winata T, Braithwaite J. Do quality management systems influence clinical safety culture and leadership? A study in 32 Australian hospitals. *Int J Qual Health Care*. 2020;**32**(Supplement_1):60-6. https://dx.doi.org/10.1093/intqhc/mzz107

Clay-Williams R, Taylor N, Ting HP, Winata T, Arnolda G, Austin E, et al. The relationships between quality management systems, safety culture and leadership and patient outcomes in Australian Emergency Departments. *Int J Qual Health Care*. 2020;**32**(Supplement_1):43-51. https://dx.doi.org/10.1093/intqhc/mzz105

Graudins LV, Dooley MJ. Medication safety: Experiential learning for pharmacy students and staff in a hospital setting. *Pharmacy*. 2016;**4**(4):17. https://dx.doi.org/10.3390/pharmacy4040038

Greenfield D, Hinchcliff R, Banks M, Mumford V, Hogden A, Debono D, et al. Analysing 'big picture' policy reform mechanisms: the Australian health service safety and quality accreditation scheme. *Health Expect*. 2015;**18**(6):3110-22. https://dx.doi.org/10.1111/hex.12300

Hanley E, Quoyle C. Approaches to surveillance of staphylococcus aureus bacteraemia and clostridium difficile infection in Australian states and territories. *Healthc Infect*. 2014;**19**(4):141-6. https://dx.doi.org/10.1071/HI14019

Hughes CF, Mackay P. Sea change: Public reporting and the safety and quality of the Australian health care system. *Med J Aust*. 2006;**184**(10S):S44-S7. https://dx.doi.org/10.5694/j.1326-5377.2006.tb00361.x

Johnson A, Clay-Williams R, Lane P. Framework for better care: reconciling approaches to patient safety and quality. *Aust Health Rev*. 2019;**43**(6):653-5. https://dx.doi.org/10.1071/AH18050

Jones D. Improving clinical leadership in patient safety: from strategic principle to active participation -- the Western Australian approach. *Int J Clin Leadersh*. 2008;**16**(3):157-62.

Leggat SG, Balding C. Effective quality systems: implementation in Australian public hospitals. *Int J Health Care Qual Assur*. 2018;**31**(8):1044-57. https://dx.doi.org/10.1108/IJHCQA-02-2017-0037

Lucas J, Leggat SG, Taylor NF. Association between use of clinical governance systems at the frontline and patient safety: a pre-post study. *Int J Health Gov*. 2022;**27**(3):282-95. https://dx.doi.org/10.1108/IJHG-02-2022-0023

Ong N, Lucien A, Long J, Weise J, Burgess A, Walton M. What do parents think about the quality and safety of care provided by hospitals to children and young people with an intellectual disability? A qualitative study using thematic analysis. *Health Expect*. 2023;**27**(1):e13925. https://dx.doi.org/10.1111/hex.13925

For peer review only - http://bmjopen.bmj.com/site/about/guidelines.xhtml

For peer review only

Pain C, Green M, Duff C, Hyland D, Pantle A, Fitzpatrick K, et al. Between the flags: implementing a safety-net system at scale to recognise and manage deteriorating patients in the New South Wales Public Health System. *Int J Qual Health Care*. 2017;**29**(1):130-6. https://dx.doi.org/10.1093/intqhc/mzw132

Rubin GL, Leeder SR. Health care safety: what needs to be done? *Med J Aust*. 2005;**183**(10):529-31. https://dx.doi.org/10.5694/j.1326-5377.2005.tb07155.x

Runciman WB. Lessons from the Australian Patient Safety Foundation: setting up a national patient safety surveillance system--is this the right model? *Qual Saf Health Care*. 2002;**11**(3):246-51. https://dx.doi.org/10.1136/qhc.11.3.246

Sinni SV, Wallace EM, Cross WM. Perinatal staff perceptions of safety and quality in their service. *BMC Health Serv Res*. 2014;**14**:591. https://dx.doi.org/10.1186/s12913-014-0591-4

Spigelman AD, Swan J. Review of the Australian incident monitoring system. *ANZ J Surg*. 2005;**75**(8):657-61. https://dx.doi.org/10.1111/j.1445-2197.2005.03482.x

Spigelman AD, Rendalls S. Clinical governance in Australia. *Clin Gov*. 2015;**20**(2):56-73. https://doi.org/10.1108/CGIJ-03-2015-0008

Stark HE, Graudins LV, McGuire TM, Lee CYY, Duguid MJ. Implementing a sustainable medication reconciliation process in Australian hospitals: The World Health Organization High 5s project. *Res Social Adm Pharm*. 2020;**16**(3):290-8. https://doi.org/10.1016/j.sapharm.2019.05.011

Stride P, Seleem M, Nath N, Horne A, Kapitsalas C. Integration of patient safety systems in a suburban hospital. *Aust Health Rev*. 2012;**36**(4):359-62. https://doi.org/10.1071/AH11099

Taitz J, Genn K, Brooks V, Ross D, Ryan K, Shumack B, et al. System-wide learning from root cause analysis: a report from the New South Wales Root Cause Analysis Review Committee. *Qual Saf Health Care*. 2010;**19**(6):e63. https://doi.org/10.1136/qshc.2008.032144

Taylor KA, Durrheim DN, Merritt T, Massey P, Ferguson J, Ryan N, et al. Multidisciplinary analysis of invasive meningococcal disease as a framework for continuous quality and safety improvement in regional Australia. *BMJ Open Qual*. 2018;**7**(1):e000077. https://doi.org/10.1136/bmjoq-2017-000077

Travaglia JF, Westbrook MT, Braithwaite J. Implementation of a patient safety incident management system as viewed by doctors, nurses and allied health professionals. *Health*. 2009;**13**(3):277-96. https://doi.org/10.1177/1363459308101804

For peer review only - http://bmjopen.bmj.com/site/about/guidelines.xhtml

Westbrook MT, Braithwaite J, Travaglia JF, Long D, Jorm C, Iedema RA. Promoting safety: longer-term responses of three health professional groups to a safety improvement programme. Int *J Health Care Qual Assur*. 2007;**20**(7):555-71. https://doi.org/10.1108/09526860710822707

Australia & Canada

Arah OA, Klazinga NS. How safe is the safety paradigm? *Qual Saf Health Care*. 2004;**13**(3):226-32. https://doi.org/10.1136/qshc.2003.007070

Cheng L, Sun N, Li Y, Zhang Z, Wang L, Zhou J, et al. International comparative analyses of incidents reporting systems for healthcare risk management. *J Evid Based Med*. 2011;**4**(1):32-47. https://doi.org/10.1111/j.1756-5391.2011.01119.x

Australia & New Zealand

Adamson L, Beldham-Collins R, Sykes J, Thwaites D. Safety culture and incident learning systems in radiation oncology: Staff perceptions across Australia and New Zealand. *J Med Imaging Radiat Oncol*. 2022;**66**(2):299-309. https://doi.org/10.1111/1754-9485.13335

Harrison R, Lee H, Sharma A. A survey of the impact of patient adverse events and near misses on anaesthetists in Australia and New Zealand. *Anaesth Intensive Care*. 2018;**46**(5):510-5. https://doi.org/10.1177/0310057X1804600513

Canada

Baker RG. Governance, policy and system-level efforts to support safer healthcare. *Healthc Q*. 2014;**17 Spec no**:21-6. https://doi.org/10.12927/hcq.2014.23955

Beard P, Smyrski L. Reporting for learning and improvement: the Manitoba and Saskatchewan experience. *Healthc Q*. 2006;**9**:61-4. https://doi.org/10.12927/hcq..18461

Beesoon S, Robert J, White J. Surgery Strategic Clinical Network: Improving quality, safety and access to surgical care in Alberta. *CMAJ*. 2019;**191**:S27-S9. https://doi.org/10.1503/cmaj.190590

Chartier LB, Ovens H, Hayes E, Davis B, Calder L, Schull M, et al. Improving quality of care through a mandatory provincial audit program: Ontario's emergency department return visit quality program. *Ann Emerg Med*. 2021;**77**(2):193-202. https://doi.org/10.1016/j.annemergmed.2020.09.449

Cochrane D, Taylor A, Miller G, Hait V, Matsui I, Bharadwaj M, et al. Establishing a provincial patient safety and learning system: pilot project results and lessons learned. *Healthc Q*. 2009;**12**:147-53. https://doi.org/10.12927/hcq.2009.20717

For peer review only - http://bmjopen.bmj.com/site/about/guidelines.xhtml

Cowell J, Harvie M. HQCA: building a credible, transparent, and independent healthcare quality and safety organization in Alberta. *Healthc Manage Forum*. 2012;**25**(4):185-7. https://doi.org/10.1016/j.hcmf.2012.09.011

Espin S, D'Arpino M, Indar A, Gross M. Incident management in health care: A pan-Canadian perspective. *J Nurs Care Qual*. 2022;**37**(1):E15-E21. https://doi.org/10.1097/NCQ.0000000000000572

Hewitt T, Chreim S, Forster A. Sociocultural factors influencing incident reporting among physicians and nurses: understanding frames underlying self- and peer-reporting practices. *J Patient Saf*. 2017;**13**(3):129-37. https://doi.org/10.1097/PTS.0000000000000130

Jeffs L, Baker GR, Taggar R, Hubley P, Richards J, Merkley J, et al. Attributes and actions required to advance quality and safety in hospitals: Insights from nurse executives. *Nurs Leadersh*. 2018;**31**(2):20-31. https://doi.org/10.12927/cjnl.2018.25606

Jeffs L, Bruno F, Zeng RL, Schonewille N, Kinder K, De Souza G, et al. Integrating implementation science in a quality and patient safety improvement learning collaborative: Essential ingredients and impact. *Jt Comm J Qual Patient Saf*. 2023;**49**(5):255-64. https://doi.org/10.1016/j.jcjq.2023.02.001

Kassam A, Sharma N, Harvie M, O'Beirne M, Topps M. Patient safety principles in family medicine residency accreditation standards and curriculum objectives: Implications for primary care. *Can Fam Physician*. 2016;**62**(12):e731-e9.

Law MP, Zimmerman R, Baker GR, Smith T. Assessment of safety culture maturity in a hospital setting. *Healthc Q*. 2010;**13**:110-5. https://doi.org/10.12927/hcq.2010.21975

Milligan C, Allin S, Farr M, Farmanova E, Peckham A, Byrd J, et al. Mandatory reporting legislation in Canada: improving systems for patient safety? *Health Econ Policy Law*. 2021;**16**(3):355-70. https://doi.org/10.1017/S1744133121000050

Milne JK, Lalonde AB. Patient safety in women's health-care: professional colleges can make a difference. The Society of Obstetricians and Gynaecologists of Canada MORE(OB) program. *Best Pract Res Clin Obstet Gynaecol*. 2007;**21**(4):565-79. https://doi.org/10.1016/j.bpobgyn.2007.01.013

Mitchell JI, Izad Shenas SA, Kuziemsky C. Governance standards: A roadmap for increasing safety at care transitions. *Healthc Manage Forum*. 2015;**28**(1):28-33. https://doi.org/10.1177/0840470414551894

Nicklin W, Mass H, Affonso DD, O'Connor P, Ferguson-Paré M, Jeffs L, et al. Patient safety culture and leadership within Canada's Academic Health Science Centres: towards the development of a collaborative position paper. Nurs Leadersh. 2004;**17**(1):22-34. https://doi.org/10.12927/cjnl.2004.16243

For peer review only - http://bmjopen.bmj.com/site/about/guidelines.xhtml

O'Beirne M, Reid R, Zwicker K, Sterling P, Sokol E, Flemons W, et al. The costs of developing, implementing, and operating a safety learning system in community practice. *J Patient Saf*. 2013;**9**(4):211-8. https://doi.org/10.1097/PTS.0000000000000052

O'Beirne M, Sterling PD. Medical safety and community practice: necessary elements and barriers to implement a safety learning system. *Healthc Q*. 2009;**12**:141-6. https://doi.org/10.12927/hcq.2009.20982

O'Beirne M, Sterling PD, Zwicker K, Hebert P, Norton PG. Safety incidents in family medicine. *BMJ Qual Saf*. 2011;**20**(12):1005-10. 10.1136/bmjqs-2011-000105

Skutezky T, Small SS, Peddie D, Balka E, Hohl CM. Beliefs and perceptions of patient safety event reporting in a Canadian Emergency Department: a qualitative study. *CJEM*. 2022;**24**(8):867-75. https://doi.org/10.1007/s43678-022-00400-2

Weaver RR. Seeking high reliability in primary care: Leadership, tools, and organization. *Health Care Manage Rev*. 2015;**40**(3):183-92. https://doi.org/10.1097/HMR.0000000000000022

Williams A. Nursing informaticians address patient safety to improve usability of health information technologies. *Stud Health Technol Inform*. 2019;**257**:501-7.

Zecevic AA, Ho-Ting Li A, Ngo C, Halligan M, Kothari A, Li AH-T. Improving safety culture in hospitals: Facilitators and barriers to implementation of Systemic Falls Investigative Method (SFIM). *Int J Qual Health Care*. 2017;**29**(3):371-7. https://doi.org/10.1093/intqhc/mzx034

Update search:

Kuluski K, Asselbergs M, Baker R, Burns KKK, Bruno F, Saragosa M, MacLaurin A, Flintoft V, Jeffs L. 'Safety is about partnership': Safety through the lens of patients and caregivers. *Health Expect*. 2024;**27**:e13939.

Recsky C, Rush KL, MacPhee M, Stowe M, Blackburn L, Muniak A, Currie LM. Clinical informatics team members' perspectives on health information technology safety after experiential learning and safety process development: Qualitative descriptive study. *JMIR Formative Research*. 2024;**5:**e53302.

Ireland

Flynn MA, Burgess T, Crowley P. Supporting and activating clinical governance development in Ireland: sharing our learning. *J Health Organ Manag*. 2015;**29**(4):455-81. https://doi.org/10.1108/JHOM-03-2014-0046

For peer review only - http://bmjopen.bmj.com/site/about/guidelines.xhtml

O'Connor P, O'Malley R, Kaud Y, St Pierre E, Dunne R, Byrne D, et al. A scoping review of patient safety research carried out in the Republic of Ireland. *Irish J Med Sci*. 2023;**192**(1):1-9. https://doi.org/10.1007/s11845-022-02930-1

Netherlands

Bilijam CW. Patient safety in general practice. *Tijdschrift voor Kindergeneeskunde*. 2014;**82**(5):155-9.

Cords CI, van der Vlies CH, Stoop M, Nieuwenhuis MK, Boudestein K, Mattace-Raso FUS, et al. Frailty screening practice in specialized burn care-a retrospective multicentre cohort study. *Eur Burn J*. 2023;**4**(1):87-100. https://doi.org/10.3390/ebj4010009

de Korne DF, Van Wijngaarden JDH, Van Dyck C, Hiddema UF, Klazinga NS. Evaluation of aviation-based safety team training in a hospital in The Netherlands. *J Health Organ Manag*. 2014;**28**(6):731-53. https://doi.org/10.1108/jhom-01-2013-0008

Harmsen M, Gaal S, van Dulmen S, de Feijter E, Giesen P, Jacobs A, et al. Patient safety in Dutch primary care: a study protocol. *Implement Sci*. 2010;**5**:50. https://doi.org/10.1186/1748-5908-5-50 Heim N, van Fenema EM, Weverling-Rijnsburger AW, Tuijl JP, Jue P, Oleksik AM, et al. Optimal screening for increased risk for adverse outcomes in hospitalised older adults. *Age Ageing*. 2015;**44**(2):239-44. https://doi.org/10.1093/ageing/afu187

Hermans MPJ, Eindhoven DC, van Winden LAM, de Grooth GJ, Blauw GJ, Muller M, et al. Frailty score for elderly patients is associated with short-term clinical outcomes in patients with ST-segment elevated myocardial infarction treated with primary percutaneous coronary intervention. *Neth Heart J*. 2019;**27**(3):127-33. https://doi.org/10.1007/s12471-019-1240-7

Jepma P, Verweij L, Tijssen A, Heymans MW, Flierman I, Latour CHM, et al. The performance of the Dutch Safety Management System frailty tool to predict the risk of readmission or mortality in older hospitalised cardiac patients. *BMC Geriatr*. 2021;**21**(1):299. https://doi.org/10.1186/s12877-021-02243-5

Jessurun JG, Hunfeld NGM, Van Rosmalen J, Van Dijk M, Van Den Bemt P. Effect of automated unit dose dispensing with barcode scanning on medication administration errors: an uncontrolled before-and-after study. *Int J Qual Health Care*. 2021;**33**(4):13. https://doi.org/10.1093/intqhc/mzab142

Oud FMM, Schut MC, Spies PE, van der Zaag-Loonen HJ, de Rooij SE, Abu-Hanna A, et al. Interaction between geriatric syndromes in predicting three months mortality risk. *Arch Gerontol Geriatr*. 2022;**103:**104774. https://doi.org/10.1016/j.archger.2022.104774

Oud FMM, Wolzak NK, Spies PE, Zaag-Loonen HJV, van Munster BC. The predictive value of the 'VMS frail older patients' for adverse outcomes in geriatric inpatients. *Arch Gerontol Geriatr*. 2021;**97**:104514. https://doi.org/10.1016/j.archger.2021.104514

For peer review only - http://bmjopen.bmj.com/site/about/guidelines.xhtml

Schuijt HJ, Oud FMM, Bruns EJR, van Duijvendijk P, Van der Zaag-Loonen HJ, Spies PE, et al. Does the Dutch Safety Management Program predict adverse outcomes for older patients in the emergency department? *Neth J Med*. 2020;**78**(5):244-50.

Snijders BMG, Emmelot-Vonk MH, Souwer ETD, Kaasjager HAH, van den Bos F. Prognostic value of screening instrument based on the Dutch national VMS guidelines for older patients in the emergency department. *Eur Geriatr Med*. 2021;**12**(1):143-50. https://doi.org/10.1007/s41999-020-00385-0

Snijders C. Patient safety in the neonatal intensive care unit: A look back on the NEOSAFE study. *Tijdschrift voor Kindergeneeskunde*. 2014;**82**(5):160-5.

van Dam CS, Trappenburg MC, Ter Wee MM, Hoogendijk EO, de Vet HC, Smulders YM, et al. The accuracy of four frequently used frailty instruments for the prediction of adverse health outcomes among older adults at two Dutch emergency departments: Findings of the AmsterGEM study. *Ann Emerg Med*. 2021;**78**(4):538-48. https://doi.org/10.1016/j.annemergmed.2021.04.027

van der Starre, C., van Dijk, M. & Tibboel, D. Real-time registration of adverse events in Dutch hospitalized children in general pediatric units: first experiences. *Eur J Pediatr.* 2012;**171**:553–558. https://doi.org/10.1007/s00431-011-1608-1

van der Zanden V, Paarlberg KM, van der Zaag-Loonen HJ, Meijer WJ, Mourits MJE, van Munster BC. Risk assessment for postoperative outcomes in a mixed hospitalized gynecological population by the Dutch safety management system (Veiligheidsmanagementsysteem, VMS) screening tool 'frail elderly'. *Arch Gynecol Obstet*. 2021;**304**(2):465-73. https://doi.org/10.1007/s00404-021-06073-z

van Munster BC, Drost D, Kalf A, Vogtlander NP. Discriminative value of frailty screening instruments in end-stage renal disease. *Clin Kidney J*. 2016;**9**(4):606-10. https://doi.org/10.1093/ckj/sfw061

van Schoten SM, Baines RJ, Spreeuwenberg P, de Bruijne MC, Groenewegen PP, Groeneweg J, et al. The ecometric properties of a measurement instrument for prospective risk analysis in hospital departments. *BMC Health Serv Res*. 2014;**14**:103. https://doi.org/10.1186/1472-6963-14-103

Verstappen W, Gaal S, Bowie P, Parker D, Lainer M, Valderas JM, et al. A research agenda on patient safety in primary care. Recommendations by the LINNEAUS collaboration on patient safety in primary care. *Eur J Gen Pract*. 2015;**21**(sup1):72-7. https://doi.org/10.3109/13814788.2015.1043726

Warnier RMJ, van Rossum E, van Kuijk SMJ, Magdelijns F, Schols J, Kempen G. Frailty screening in hospitalised older adults: How does the brief Dutch National Safety Management Program perform compared to a more extensive approach? *J Clin Nurs*. 2020;**29**(7-8):1064-73. https://doi.org/10.1111/jocn.15148

For peer review only - http://bmjopen.bmj.com/site/about/guidelines.xhtml

Weske U, Boselie P, van Rensen E, Schneider M. Physician compliance with quality and patient safety regulations: The role of perceived enforcement approaches and commitment. *Health Serv Manage Res*. 2019;**32**(2):103-12. https://doi.org/10.1177/0951484818813324

Weske U, Boselie P, van Rensen ELJ, Schneider MME. Using regulatory enforcement theory to explain compliance with quality and patient safety regulations: the case of internal audits. *BMC Health Serv Res*. 2018;**18**(1):62. https://doi.org/10.1186/s12913-018-2865-8

Update search:

Oud FMM, Meulman MD, Merten H, Wagner C, van Munster BC. Value of the Safety Management System (VMS) frailty instrument as a frailty screener in care for older hospital patients: a systematic review. *Eur Geriat Med*. 2024;**15**:609-620.

New Zealand

Raymont A, Graham P, Hider PN, Finlayson MP, Fraser J, Cumming JM. Variation in the adoption of patient safety practices among New Zealand district health boards. *Aust Health Rev*. 2012;**36**(2):163-8. https://doi.org/10.1071/AH10972

Robin G, Horsburgh S. Healthcare professional perspectives on quality and safety in New Zealand public hospitals: findings from a national survey. *Aust Health Rev*. 2014;**38**(1):109-14. https://doi.org/10.1071/AH13116

Shuker C, Bohm G, Bramley D, Frost S, Galler D, Hamblin R, et al. The Health Quality and Safety Commission: making good health care better. *N Z Med J*. 2015;**128**(1408):97-109.

Mobius Research & Strategy. The Health Quality & Safety Commission Surgical Safety Culture Survey Research Report. 2017. URL: https://www.hqsc.govt.nz/assets/Our-work/Improved-service-delivery/Safe-surgery/Publications-resources/2017_Surgical_Safety_Culture_Survey_Report_-_FINAL.pdf (accessed 24 April 2024).

For peer review only

Supplementary material 4: Rich and descriptive studies

Documents relating to patient safety and/or health services in each country included in the review

Document	Year of publication	Description of document
**Netherlands**		
Policy		
NTA 8009 'Safety management system for hospitals and institutions that provide hospital care' (initial technical agreement).[29*]	2007	Describes the basic requirements for an SMS for hospitals and institutions that provide hospital care. It focuses on managing risks and reducing harm to patients. It is based on the Rein Willems report '*Here you work safely or you don't work here'* (2004) and the improvement cycle 'plan-do-check-act' on which other SMS are based. The structure of the document includes: 1. Subject and scope, 2. Terms and definitions, 3. Responsibilities of the Board of Directors, and 4. Basic requirements for the SMS. Additional information is provided in the appendices on the organisations involved in the drafting of the document; the reporting matrix (roles and responsibilities for reporting different types of incidents such as near misses and serious incidents); conversion tables that explain how the NTA relates to other standards (e.g. ISO 9001: 2000 Quality Management System standard); examples of prospective and retrospective risk assessment; and safety culture (which is not explicitly included in the basic requirements).
NEN 8009 'Safety management system for hospitals and institutions that provide hospital care' (replaces NTA 8009 2014).[30*]	2018	This is the third update of the original NTA 8009 (following updates in 2011 and 2014). It supplements, without replacing, the original SMS requirements with Safety-II concepts that promote a proactive safety culture and encourage learning from daily activities with desired outcomes as well as incidents. It acknowledges that improvement could vary from continuous improvements in small steps to significant changes at organisational level and highlights the importance of encouraging employees and patients to play an active role, identify opportunities and successful practices and contribute to the overall process of organisational learning.

For peer review only - http://bmjopen.bmj.com/site/about/guidelines.xhtml

Other				
Bal R, Wagner C. The Netherlands: Patient safety in Dutch hospitals: How can we explain success, in Health systems improvement across the Globe: Success stories from 60 countries, eds. Braithwaite J et al. Boca Raton: CRC Press.[31]	2018	Provides an overview of the Dutch patient safety programme and tries to explain its success, accounting for the broader socio-cultural and historical context, and identifying the ‘conditions under which such a programme was able to succeed’ (p. 208). It argues that five such factors contributed to the success of the Dutch patient safety programme: experience in project work, market competition, public pressure, activities of regulatory agencies, and public reporting of adverse events and mortality.		
Bolk M. Final report: Embedding patient safety in education and training. Utrecht: VMS Safety Programme.[32]	2013	Reports on the ‘*Embedding patient safety in education and training*’ project which aimed ‘to support, facilitate and accelerate education for doctors, nurses and the supporting professions (medical and otherwise), with the help of a national incentive program and the corresponding support structure’ (p. 4). As a result, a national ‘patient safety competencies framework’ was developed; all findings from the project were compiled in a digital portal and campaign website and a ‘patient safety toolkit’ was developed; examples for good patient safety education were collected; and the attention generated regarding patient safety resulted in the project linking to other educational initiatives already working on patient safety. The project made a number of recommendations for embedding patient safety in various educational and learning processes and programmes.		
Research		**Aims**	**Methods**	**Findings**
Baines R et al. How effective are patient safety initiatives? A retrospective patient record review study of changes to patient safety over time. *BMJ Qual Saf.* 2015;**24**(9):561-71.[33]	2015	To assess whether hospital care in 2011/12 was safer than in 2004 and 2008.	Retrospective patient record review. 15, 997 patient admissions were included, 7926 from 2004, 4023 from 2008 and 4048 from 2011/2012.	In comparison with 2008, uncorrected crude overall adverse event rates showed no change in 2011/2012 but preventable adverse event rates showed a reduction of 45%. After multilevel corrections, the

For peer review only - http://bmjopen.bmj.com/site/about/guidelines.xhtml

				decrease was still visible but not statistically significant.
de Blok C et al. Implementation of the Dutch National Patient Safety Programme (VMS Veiligheidsprogrammesma). Utrecht/Amsterdam: NIVEL/EMGO+.[34]	2013	English summary of de Blok[21] (see below).		
de Blok C, Koster E, Schilp J, Wagner C. Implementatie VMS Veiligheidsprogrammesma. Evaluatieonderzoek in Nederlandse ziekenhuizen. Utrecht / Amsterdam: NIVEL/EMGO+.[21]*	2013	To assess the extent to which the ten themes of the Dutch patient safety programme have been implemented and gain insight into the factors that influence the degree of implementation and compliance with the advice.	Data collection varied across themes and included observations and semi-structured interviews, review of patient records and registry data. A large proportion of Dutch hospitals were involved in the evaluation, which varied across themes.	Implementation objectives set in 2008 were almost achieved for some of the themes (e.g. 'Preventing renal insufficiency during intravascular use of iodinated contrast agent' and subtheme 'Treatment of severe sepsis'). There was a positive trend for other themes, but considerable variation was observed across and within hospitals. The study identified a range of organisational, staff- and theme-related factors associated with the success of implementation.
de Bruijne MC, Zegers M, Hoonhout LHF, Wagner C. Onbedoelde schade in Nederlandse ziekenhuizen: Dossieronderzoek van ziekenhuisopnames in 2004. Amsterdam: EMGO+ Instituut, Utrech: NIVEL[35]*	2007	To establish a baseline measurement in the field of patient safety.	Retrospective patient record review; 200 patient records from patients who were discharged and 200 from patients who died in hospitals in 2004 were randomly selected from each of the participating 21	Of all admissions: (i) 5.7% suffered unintentional healthcare-related harm, 5% of which experienced permanent harm and 8% died partly as a result of this; (ii) 2.3% suffered preventable adverse events. Of the patients who died in hospital,

For peer review only - http://bmjopen.bmj.com/site/about/guidelines.xhtml

			Dutch hospitals. Patients admitted to psychiatry and obstetrics and those less then one year of age were excluded as the assessment system was not suitable for such patients.	10.7% suffered unintentional healthcare-related events; of those, 4.1% were related to preventable adverse events during admission. It was estimated that in 2004, there were 1735 (95%CI 1482 to 2032) potentially preventable deaths in Dutch hospitals.
de Groot JF et al. Implementing paediatric early warning scores systems in the Netherlands: future implications. *BMC Pediatr*. 2018;**18**(1):128.[36]	2018	(i) To evaluate the implementation of paediatric early warning score systems in five hospitals.<br>(ii) To gain insight into the attitudes of professionals towards the system.	(i) Retrospective patient record review (n = 554) at the start, mid and endpoint of implementation.<br>(ii) Semi-structured interviews with professionals (n = 8).	(i) None of the hospitals started with a system but after 1 year, they were recorded in 69.2% of patient records and resulted in appropriate action in 49.1%.<br>(ii) Sustainable implementation might be hindered by staff concerns about validity, effectiveness and communication with other hospitals during transfers to higher levels of care.
Klopotowska J, Schutijser B, de Bruijne M, Wagner C. Tweede evaluatie van VMS Programma in Nederlandse ziekenhuizen op basis van vier veiligheidsthema's: aangevuld met EPD Scan naar mogelijkheden voor toekomstige metingen. Utrecht/Amsterdam: NIVEL/EMGO+.[37*]	2016	To investigate the progress of the implementation of four safety themes from the Dutch national patient safety programme compared to the first evaluation study in 2011/2012 and assess the possibility of automatic monitoring of process indicators from	Data collection:<br>(i) Theme 'medication verification': review of patient records (n=1060) in 15 hospitals.<br>(ii) Theme 'high risk medication': observations of nurses (n=349) in 15 hospitals.	There was improvement in compliance for (i) and (ii) but still the target of 100% was not met; authors note that 100% compliance may not be desirable as in some cases deviation from guidelines could be beneficial for the patient. For (iii) compliance increased by 34% (to 73% against 90% target). For (iv) hospital mortality among severe sepsis patients

For peer review only - http://bmjopen.bmj.com/site/about/guidelines.xhtml

		electronic patient records for two of the themes.	Themes (iii) 'prevention of wound infections after surgery' and (iv) 'prevention of line sepsis and treatment of severe sepsis': data from national registries.	has fallen from 34% to 29% (15% relative mortality reduction) but for line sepsis positive results were observed only for the ICU departments on which the hospitals focused initially (incidence was 2.5 times higher in non-ICU).
Langelaan M et al. Monitor zorggerelateerde schade 2008: Dossieronderzoek in Nederlandse ziekenhuizen. Utrecht: NIVEL, Amsterdam: EMGO+ Instituut.[38]*	2010	To estimate and characterise potentially avoidable adverse events and deaths, and those related to the use of medical technology; to compare them to 2004.	Retrospective patient record review: stratified samples of 100 admissions of discharged patients and 100 of patients who died in hospital were drawn from each of the 20 participating hospitals. Psychiatry, obstetrics and patients < 1 year of age were excluded.	2.9% potentially avoidable adverse events (of all admissions). The change from 2004 was not statistically significant; 0.8% was related to the use of medical technology. Potentially avoidable mortality was 5.5% (95%CI 4.5% to 6.6%); the change from 2004 was not statistically significant. In absolute numbers, in 2008 there were 1960 (95%CI 1600 to 2460) patients in whom potentially avoidable harm contributed to the death.
Langelaan M et al. Monitor Zorggerelateerde Schade 2011/2012. Dossieronderzoek in Nederlandse ziekenhuizen. Amsterdam: VU medisch centrum & EMGO+ Instituut, Utrecht: NIVEL.[39]*	2013	To estimate and characterise potentially avoidable adverse events and mortality in Dutch hospitals, including those related to medical technology, and compare them to previous measurements.	Retrospective patient record review study based on data from 20 randomly selected Dutch hospitals (>4000 records in total); random samples were drawn from each hospital of 100 discharged patients and 100 patients who died in the hospital. Psychiatry,	Of all patients admitted in 2011/2012, 1.6% (95% CI: 1.1% to 2.2%) experienced potentially avoidable harm: 30% and 45% less than in 2004 and 2008, respectively. Potentially avoidable mortality decreased by 37% and 53% compared to 2004 and 2008, respectively, which expressed in absolute

For peer review only - http://bmjopen.bmj.com/site/about/guidelines.xhtml

		To estimate the costs of healthcare-related harm and the quality of file management.	obstetrics and patients < 1 year of age were excluded.	numbers and extrapolated to all Dutch hospitals was approx. 1,735 patients (95% CI: 1,482 to 2,032) in 2004, 1,960 patients (95% CI: 1,600 to 2,360) in 2008, and 970 (95% CI: 738 to 1,274) patients between 1st April 2011 and 31st March 2012. Other findings included harms related to the application of medical technology, costs related to adverse events and the quality of patient records.
Langelaan M et al. Monitor Zorggerelateerde Schade 2015/2016: Dossieronderzoek bij overleden patiënten in Nederlandse ziekenhuizen. Utrecht: NIVEL, Amsterdam: EMGO+ Instituut.[40]*	2017	To estimate and characterise potentially avoidable adverse events and mortality in Dutch hospitals, including those related to medical technology, and compare them to previous measurements.	Retrospective patient record review: a random sample of 150 patients who died in hospital was drawn from each of the 19 hospitals, randomly selected to participate in the study. Psychiatry, obstetrics and patients < 1 year of age were excluded.	Of all deceased patients, 4.3% (95%CI 3.6% to 5.1%) suffered potentially preventable adverse events and 3.1% (95%CI 2.5% to 3.8%) suffered potentially preventable mortality. The difference with 2011/12 was not statistically significant.<br>The respective results related to the use of medical technology were 1.7% and 1.3%, out of all cases in which technology was used.
Meulman M, Merten H, Wagner C. Het screenen van kwetsbare ouderen: onderzoek vanuit een Safety-I en Safety-II perspectief: evaluatie van het VMS thema	2021	To investigate the degree of compliance with the national Dutch patient safety programme screening	Functional Resonance Analysis Method was used, based on analysis of hospital protocols, interviews with nurses (n=31), and a feedback meeting. Additionally, a	Hospital protocols were more detailed than the national guidelines but largely similar. There were actions in the protocols that were not performed, and actions that were performed but not included

For peer review only - http://bmjopen.bmj.com/site/about/guidelines.xhtml

Kwetsbare ouderen. Utrecht: NIVEL[41]*		tool for vulnerable older people.	survey of employees' resilience (n=146), an evaluation interview in each department, and compliance data (the percentage of admitted patients aged ≥70 who were screened). Data were collected between 2020-2021, from 10 departments in 6 hospitals.	in the protocols. An average of 74% of admitted patients were screened. Individual resilience (66%) was rated more positively than team resilience (49%).
Meurs M, de Groot JF. Veiligheidsmanagement systeem (VMS) thema's kindzorg Pijn en (Lijn)sepsis: verbeterpunten vanuit het werkveld voor verdere kwaliteitsverbetering. Utrecht: NIVEL. [42]*	2020	To identify bottlenecks and areas of improvement for implementation of the Dutch national patient safety programme themes '(line) sepsis' and 'pain' within paediatric care hospitals.	Working groups with paediatricians and paediatric nurses, and a survey (n=209). Data was collected in 2019.	Theme '(line) sepsis': the main recommendation regarded earlier recognition and treatment of sepsis (e.g. through training).<br>Theme 'pain': the main recommendation for improvement was to revise guidelines to adopt a holistic approach to pain recognition and treatment.
Sambeeck SJV et al. Pediatric Early Warning System Scores: Lessons to be learned. *J Pediatr Intensive* Care. 2018;**7**(1):27-32.[43]	2018	To investigate how many hospitals complied with the national recommendation to implement a paediatric early warning system score and if so, the score they chose to implement.	Cross-sectional electronic survey of Dutch hospitals in 2014 (n=91).	Three-quarters of Dutch hospitals with a paediatric department implemented a score. These hospitals used 45 different versions of a paediatric early warning system score, the majority of which were not validated.

For peer review only - http://bmjopen.bmj.com/site/about/guidelines.xhtml

Schlinkert C, Jelsma J, Wagner C. Patiëntveiligheidscultuur in Nederlandse Ziekenhuizen. Van openheid naar reflectie en veerkracht. Utrecht: NIVEL.[44*]	2021	To measure the patient safety culture in Dutch hospitals in 2020 and compare it with previous measurements (before and after the implementation of the Dutch patient safety programme). To measure the employees' resilience.	Cross-sectional survey on patient safety culture (Dutch version of Hospital Survey on Patient Safety Culture) and employees' resilience. Data were collected in 2020; 3014 employees from 5 hospitals completed the survey. Comparison with data collected using the same survey in 2005-2007 and 2012.	62.5% of respondents rated patient safety in their own department 'very good' or 'excellent'. Teamwork was the highest rated dimension, 'Adequate staffing', 'Support from management' and 'Collaboration between departments' were rated lowest. Nursing staff rated patient safety more negatively than other professional groups. More respondents considered patient safety to be 'very good' or 'excellent' in 2020 compared to previous measurements.
van Dijk LM, van Eikenhorst L, Muns L, Wagner C. Medicatieverificatie bij ontslag: nieuwe benadering vanuit een Safety-I en Safety-II perspectief: derde evaluatie van het VMS-thema. Utrecht: NIVEL.[45*]	2021	To investigate the implementation of the 'medication verification at discharge' theme of the Dutch national patient safety programme.	Functional Resonance Analysis Method was used, based on hospital protocols, interviews with nurses (n=77), and a feedback meeting. A survey of employees' resilience (n=142) and an evaluation interview in each department were carried out, and compliance data were collected (percentage of discharged patients aged ≥18 for whom medication verification took place upon discharge). Data was collected between 2020-	Hospital protocols were more detailed than the national guidelines. There were variations in how the processes were carried out in practice, normally in response to circumstances in the work environment (e.g. IT problems, staff shortages). Compliance rates varied between 67%-100% across departments over the data collection period. Both individual (64%) and team (69%) resilience was rated positively.

For peer review only - http://bmjopen.bmj.com/site/about/guidelines.xhtml

			2021, from 9 departments in 8 hospitals (4 cardiology departments and 5 orthopaedics departments).	
van der Starre C et al. Paediatric critical incident analysis: lessons learnt on analysis, recommendations and implementation. *Eur J Pediatr*. 2014;**173**(11):1449-57.[46]	2014	To identify potential causal and contributing factors of serious paediatric patient safety incidents, and to report on and assess the extent of implementation of recommendations for improvement.	Retrospective review of critical incident analyses (n=17) from 2005–2010.	A median number of 5 causal and contributing factors was identified per incident and were mostly likely to be team or task-related. There was a median of 5 recommendations per analysis, only one-third of recommendations were implemented.
van Noord N, Zwijnenberg I, Wagner C. Patiëntveiligheidscultuur in Nederlandse Ziekenhuizen. Een stap in de goede richting. Utrecht: NIVEL.[47]*	2013	To examine whether the Dutch national patient safety programme improved patient safety culture.	Comparison of two cross-sectional safety culture surveys (Dutch version of Hospital Survey on Patient Safety Culture). Between 2005-2007, 3779 healthcare providers in 45 hospitals responded, in 2012, 6605 healthcare providers in 24 hospitals responded.	Patient safety culture improved between 2005-2007 and 2012, with 48.8% of respondents rating it 'very good' or 'excellent' in 2012. However, there was variation between hospitals and departments. 'Teamwork' was the highest rated dimension of safety; 'Collaboration between departments', 'Reporting frequency' and 'Support from management' were lowest rated. Nursing staff rated patient safety more negatively than other professionals.
van Schoten S et al. The association between quality system development stage and	2018	To examine the association between the development stage	Analysis of a national survey on the development stage of hospital quality	No association was found between the development stage of a hospital quality system and

For peer review only - http://bmjopen.bmj.com/site/about/guidelines.xhtml

the implementation of process-level patient safety themes in Dutch hospitals: an observational study. *BMC Health Serv Res.* 2018;**18**:189.[48]		of hospital quality systems and the process-level implementation of patient safety themes within the Dutch patient safety programme.	systems (conducted in 2011) and an evaluation study of the national Dutch patient safety program (2011-2012). Data from 30 hospitals (12,485 observations) were included.	the process-level implementation of patient safety themes.
van Schoten S et al. Monitor Zorggerelateerde Schade 2019: Dossieronderzoek bij overleden patiënten in Nederlandse ziekenhuizen. Utrecht: NIVEL, Amsterdam: APH.[49*]	2022	To estimate and characterise potentially avoidable adverse events and mortality in Dutch hospitals, including those related to medical technology and diagnostic processes, and those in vulnerable older patients, and compare them to previous measurements.<br>To assess the quality of file management.	Retrospective patient record review: a random sample of 150 patients who died in hospital was drawn from each of the 20 hospitals, randomly selected to participate in the study. Psychiatry, obstetrics and patients < 1 year of age were excluded.	14.6% (95%CI 13.3% to 16.0%) of patients who died in hospital suffered care-related harm. This is a significant increase compared to 2015/2016 (was 9.9% (95% CI 8.9% to 11.0%)). Potentially avoidable adverse events and mortality were 4.2% (95%CI 3.5% to 5.0%) and 3.1% (95% CI 2.5% to 3.9%), respectively, indicating that the change from the previous measurement was not statistically significant.
van Stralen S, Schutijser B, Vonk A, van Eikenhorst L, Wagner C. Tweede controle bij het klaarmaken en toedienen van high-risk medicatie: nieuwe benadering vanuit een Safety-I en Safety-II perspectief. Derde evaluatie van het VMS thema. Utrecht: NIVEL. [50*]	2021	To investigate the implementation of the 'high-risk medication' theme (specifically the second check of medication) of the Dutch national patient safety programme.	Functional Resonance Analysis Method was used, based on hospital protocols, interviews with nurses (n=77), and a feedback meeting. Additionally, a survey of employee resilience (n=142) and an evaluation interview in each	Compared to the national guidelines from the patient safety programme, hospital protocols were largely the same. There were variations in how nurses carried out the processes, normally in response to circumstances in the work environment (e.g. time pressures). Individual (average

For peer review only - http://bmjopen.bmj.com/site/about/guidelines.xhtml

			department were carried out, and compliance data collected. Data was collected between 2020-2021, from 10 departments in 9 hospitals (3 surgery departments, 3 internal medicine departments and 4 intensive care units).	score 3.21/9) and team resilience (average score 4.08/9) was rated highly.
Verbeek-van Noord I et al. A nation-wide transition in patient safety culture: a multilevel analysis on two cross-sectional surveys. *Int J Qual Health Care*. 2019;**31**(8):627–632.[51]	2019	To examine whether the Dutch national patient safety programme improved patient safety culture.	Comparison of two cross-sectional safety culture surveys (Dutch version of Hospital Survey on Patient Safety Culture). Between 2005-2007, 3779 healthcare providers in 45 hospitals responded, in 2012, 6605 healthcare providers in 24 hospitals responded.	Patient safety culture improved over the studied period. However, there was variation between hospitals and departments.
**Australia**				
Policy				
Australian Commission on Safety and Quality in Health Care. National model clinical governance framework.[63]	2017	This framework defines clinical governance as 'a set of relationships and responsibilities established by a health service organisation between its state or territory department of health, governing body, executive, workforce, patients, consumers and other stakeholders'.(p.iii) The framework contains all the elements that acute health service organisations need to implement integrated corporate and clinical governance systems, with the aim of ensuring good clinical outcomes through the delivery of safe and quality care. It is based on the National Safety and Quality Health Service Standards (NSQHS) Clinical Governance and Partnering with Consumers (see below) and contains five components: governance, leadership and culture, patient safety and quality		

For peer review only - http://bmjopen.bmj.com/site/about/guidelines.xhtml

		improvement systems, clinical performance and effectiveness, safe environment for the delivery of care, and partnering with consumers.		
Australian Commission on Safety and Quality in Health Care. National safety and quality health service standards. 2nd edition.[64]	2021	Describe standards intended for organisation-wide implementation in all hospital and day procedure services to enable the delivery of nationally consistent, safe and quality care. There are eight Standards - Clinical Governance, Partnering with Consumers, Preventing and Controlling Healthcare-Associated Infection, Medication Safety, Comprehensive Care, Communicating for Safety, Blood Management and Recognising and Responding to Acute Deterioration. The Clinical Governance and Partnering with Consumers Standards set out overarching requirements for the implementation of the other six Standards. The Standards do not specify how they should be implemented to allow for local adaptation.		
Australian Commission on Safety and Quality in Health Care. National safety and quality primary and community healthcare standards.[65]	2021	Describes a framework which is intended for use in all primary and community healthcare services. The processes and structures in the Standards are intended to enable the delivery of, and improve, the quality and safety of services. There are three Standards - Clinical Governance, Partnering with Consumers, and Clinical Safety – with the former two Standards setting out overarching requirements for the implementation of the Clinical Safety Standards.		
Research		**Aims**	**Methods**	**Findings**
Australian Commission on Safety and Quality in Health Care. Creating safer, better healthcare - the impact of national safety and quality health service standards.[66]	2018	To provide an overview of changes associated with implementation of the first edition of the NSQHS Standards, identifying areas where improvement has been made and where further work is needed.	Draws on a numbers of research projects, including surveys of hospital staff, hospital board members, and specialist medical colleges, National Inpatient Medication Chart Audits, and a Consumer Participation Study involving interviews and focus groups.	Implementation of the NSQHS Standards has resulted in the prevention of harm, improvements in patient care including empowerment of consumers and patients, development of better governance systems, and reduction of waste.

For peer review only - http://bmjopen.bmj.com/site/about/guidelines.xhtml

Australian Commission on Safety and Quality in Health Care. The state of patient safety and quality in Australian hospitals 2019.[67]	2019	To report on safety and quality in public hospitals in Australia, including common patient safety risks, action taken to deliver appropriate care, and the Commission's approach to supporting value-based healthcare.	Draws on data on public hospitals from a wide range of sources, such as the Admitted Patient Care National Minimum Data Set, 2013–14 to 2017–18 and the Australian Atlas of Healthcare Variation.	There have been significant improvements in the safety and quality of care over the last 10 years. These include improved patient outcomes such as reductions in hospital acquired infections and preventable in-hospital cardiac arrests, improved patient experience and involvement in care, and better governance of clinical care.
**Canada**				
Policy				
Canadian Patient Safety Institute. Canadian disclosure guidelines. Being open with patients and family.[68]	2011	'The guidelines are intended to encourage and support healthcare providers, interdisciplinary teams, organizations and regulatory authorities in developing and implementing disclosure policies, practices and training methods.' (p. 13) The following guiding principles underpin the development of the guidelines: patient-centred healthcare, patient autonomy, safe healthcare, leadership support, disclosure is the right thing to do, honesty and transparency. It recommends the following elements to be included in a disclosure policy: policy statement/objectives, definitions of key terms, provision for patient support, provision for healthcare provider support and education, the disclosure process, provision for particular circumstances (Appendix C).		
Canadian Patient Safety Institute. Strengthening commitment for improvement together. A policy framework for patient safety.[69]	2019	A policy framework for patient safety aiming to stimulate conversation and action on the following policy levers: regulations, standards, organisational policies and public engagement.		
Health Standards Organisation & Canadian Patient Safety Institute. The Canadian quality and patient	2020	Defines five areas - people-centred care, safe care, accessible care, appropriate care, and integrated care – for health services to focus on to provide safe and quality care. These goal areas are intended 'to drive improvement and to align Canadian legislation, regulations, standards, organizational policies, and public engagement on patient safety		

For peer review only - http://bmjopen.bmj.com/site/about/guidelines.xhtml

safety framework for health services.[70]		and quality improvement.'(p.3) The framework includes actions guides customised to five stakeholder groups - the public, health teams, health leaders, board members and policymakers - which identify objectives and outcomes for each goal, indicators to track success, and resources.		
Healthcare Excellence Canada. Status of patient safety incident legislation and best practices across Canada. Summary report.[71]	2021	Summarises and compares the status of four types of patient safety incident legislation - mandatory reporting, mandatory disclosure, apology protection, and quality assurance - in federal, provincial and territorial jurisdictions across Canada. The report also describes important characteristics of each type of legislation and comments on current or potential changes in policy direction which may be significant for the future development of legislation.		
Healthcare Excellence Canada, Patients for Patient Safety Canada. Rethinking patient safety. A discussion guide for patients, healthcare providers and leaders.[72]	2023	Summarises discussions with health service users, healthcare professionals, and safety scientists. Describes a new approach to patient safety in Canada, involving (i) the development of a positive culture of safety, with everyone contributing to creating safe conditions, (ii) acknowledging that the absence of harm does not mean that care is safe, and (iii) that all forms of harm matter, including those that are not physical.		
Research		**Aims**	**Methods**	**Findings**
Goldman J, Rotteau L. Evaluation research of Measurement and Monitoring of Safety Framework collaborative.[73]	2020	To (i) understand the effectiveness of a learning collaborative in teaching participants about the Measurement and Monitoring of Safety Framework (MMSF), and (ii) examine the implementation of the MMSF.	Qualitative study with three types of data collection:<br>(i) Interviews (n=36, with participants from teams including emergency, surgery, medicine, cardiology, psychiatry, supportive living and long-term care settings).<br>(ii) Observations of sites (n=5) where MMSF was being implemented and learning sessions and calls	Participants found the sessions to learn about MMSF useful, but some felt overwhelmed by the amount of information. Coaching was felt to play a key role in the implementation of MMSF in participants' organisations. A range of context-specific strategies were used by participants to implement MMSF (e.g. teaching to groups of stakeholders, integrating its language into usual communication, using it to

For peer review only - http://bmjopen.bmj.com/site/about/guidelines.xhtml

			where participants learnt about MMSF (29 hours were spent on site visits and 33 hours at a learning session).<br><br>(iii) Documentary data collection (e.g. learning session resources, tools being used in MMSF implementation).	inform activities such as safety huddles and incident reporting). Most participants reported positive impacts from MMSF implementation (e.g. changes in thinking about safety and resulting behaviour change); they supported its wider spread but found opportunities to do this beyond their sites were variable.
Goldman J, Rotteau L, Flintoft V, *et al* Measurement and Monitoring of Safety Framework: a qualitative study of implementation through a Canadian learning collaborative *BMJ Quality & Safety* 2023; **32:**470-478.[74]	2023	Journal article summarising the report[73] described above.		
**Ireland**				
Policy				
Health Information and Quality Authority. National standards for safer better healthcare.[75]	2012	Describes eight standards: person-centred care and support, effective care and support, safe care and support, better health and wellbeing, leadership, governance and management, workforce, use of resources, and use of information. These were intended to set expectations for healthcare in Ireland and guide improvement of quality, safety, and reliability. They were replaced recently by a principles-based approach.[76]		
Health Information and Quality Authority. National standards for the conduct of reviews of patient safety incidents.[77]	2017	Details standards based around five themes: governance and accountability, person-centred approach to the review of patient safety incidents, workforce, reviews of patient safety incidents, and sharing the learning for improvement. These are intended to ensure a consistent national approach to reviews of patient safety incidents.		

For peer review only - http://bmjopen.bmj.com/site/about/guidelines.xhtml

Health Information and Quality Authority. Standards development framework: a principles-based approach.[76]	2021	Describes a set of principles which are designed to underpin all national standards for health and social care services, to be used as framework during their development. These principles are a human rights-based approach, safety and wellbeing, responsiveness, and accountability; they are all intended to ensure patients receive person-centred care and support.
Health Service Executive. Patient safety strategy 2019-2024.[78]	2019	Details eight patient safety commitments (including specific actions within each commitment, and the rationale and principles behind them): empowering and engaging patients to improve patient safety, empowering and engaging staff to improve patient safety, anticipating and responding to risks to patient safety, reducing common causes of harm, using information to improve patient safety, and leadership and governance to improve patient safety. Its primary purpose is to guide safety improvement at the service level.
Health Service Executive. Healthcare audit – quality assurance and verification. Standards.[79]	2019	Sets out a framework and basic principles – integrity, objectivity, confidentiality, and competency - for the conduct of healthcare audits in Ireland. All HSE healthcare organisations and private or voluntary bodies with a service level agreement with the HSE are required to be audited (in addition to their internal audit functions) by HSE Healthcare auditors, who are members of the Chartered Institute of Internal Auditors. Audits are independent, objective assessments of the delivery of healthcare services. They provide assurance that standards are being met and recommendations to drive improvement.
Health Service Executive. Incident management framework.[80]	2020	A framework that describes a practical approach all healthcare providers can use to identify, report and review incidents. The framework focuses on the system rather than individual. Six steps are described in the process: preventing incidents by supporting a culture where safety is a priority, identifying incidents and immediate actions, initial reporting and notification, categorisation and initial assessment (from category 1, a major incident, to category 3, a minor incident, which determines who needs to be notified and the level of review required), review and analysis, and improvement planning and monitoring.

For peer review only - http://bmjopen.bmj.com/site/about/guidelines.xhtml

Health Service Executive. HSE Enterprise risk management policy and procedures 2023.[81]	2023	A practical resource for healthcare professionals, whether working in a clinical or management capacity, intended to be used in planning and strategy development, decision-making, and day-to-day management. It sets out policy, processes and procedures for the proactive management of risk in healthcare organisations, based on an enterprise approach (i.e. considers all risks, whether management or service delivery-related, together). This involves identifying and predicting uncertainties and threats, describing and analysing the level of the threat, responding where possible (e.g. through control measures), and reviewing and acting (e.g. communication and escalation regarding a threat).		
Research		**Aims**	**Methods**	**Findings**
Health Information and Quality Authority. Overview report of five years of HIQA monitoring in Irish public acute hospitals against national standards: 2015–2019.[82]	2020	To report key findings, examples of good practice and opportunities for improvement identified during monitoring of four national standards - infection prevention and control, medication safety, maternity services, and nutrition and hydration.	On-site inspections in hospitals between 2015-2019: infection prevention and control (n=161), medication safety (n=64), maternity services (n=22), and nutrition and hydration (n=5).	The greatest improvements were seen in relation to infection prevention and control and medication safety. However, there was variation between hospitals, affecting the quality of care provided. Factors such as insufficient resources hindered the implementation of the national standards.
**New Zealand**				
Policy				
Ministry of Health. Pae Ora (Healthy Futures) Act 2022.[85]	2022	The purpose of the Act is to provide for the public funding and provision of services in order to: (a) protect, promote, and improve the health of all New Zealanders; and (b) achieve equity in health outcomes among New Zealand's population groups, including striving to eliminate health disparities, in particular for Māori; and (c) build towards pae ora (healthy futures) for all New Zealanders.		

For peer review only - http://bmjopen.bmj.com/site/about/guidelines.xhtml

Simmonds S, Carter M, Haggie H, Mills V, Lyndon M, Tipene-Leach D. A Cultural Safety Training Plan for Vocational Medicine in Aotearoa. Te ORA and the Council of Medical Colleges.[86]	2023	Presents 'a plan for cultural safety training that medical colleges can employ in the development of their own cultural safety training programmes and in continuing professional development (CPD) with college fellows.'(p. 4) The plan is specific to the New Zealand context and 'focuses on cultural safety through the lens of Māori patients and their whānau.'(p. 4)
Standards New Zealand. NZS 8134:2021 Ngā Paerewa Health and disability services standard.[87]	2022	The standard promotes the safe provision of fair and equitable services for people and their whānau in New Zealand Aotearoa. It sets the foundations for promoting best practices and fostering continuous improvement in the quality (including safety) of health and disability services.
Te Tāhū Hauora Health Quality & Safety Commission. From knowledge to action: A framework for building quality and safety capability in the New Zealand health system.[88]	2016	'This document describes a high-level framework to guide the development of quality and safety capability across all levels in the health and disability sector'.(p. 4) It aims to address '…existing workforce needs, sustainably building the quality improvement capability of the future workforce, developing specialist roles in quality improvement science, supporting consumer/patient participation, ensuring decision-making is based on data and evidence, and supporting boards to provide leadership that encourages a quality improvement and patient safety focus throughout the sector.'(p. 4)
Te Tāhū Hauora Health Quality & Safety Commission. Clinical governance: guidance for health and disability providers.[89]	2017	Sets out a high-level framework for clinical governance in health and disability services. The values that underpin the framework are consumer wellbeing and safety. The guide is intended for use by all health and disability service providers, regardless of their size or complexity, and their staff, as well as other stakeholders.
Te Tāhū Hauora Health Quality & Safety Commission. Clinical governance framework: collaborating for quality (draft, November 2023).[90]	2023	A draft clinical governance framework to replace the clinical governance framework of 2017. Its purpose is '…to provide a comprehensive approach to enable the health sector to develop their own clinical governance infrastructure, adapted to their clinical context.' (p. 6) 'The framework is intended to support a culture of participatory leadership, where collaboration drives quality' and is '… deliberately inclusive of the health workforce, as opposed to being solely focussed on managers and clinicians, as it is applicable across the health sector and needs to be inclusive of all health environments.' The document also states that: 'The framework has been intentionally designed for the Aotearoa New Zealand context, to enact Te Tiriti o Waitangi [the founding document of Aotearoa New

For peer review only - http://bmjopen.bmj.com/site/about/guidelines.xhtml

		Zealand, signed by the Crown and many hapū and iwi leaders] and strive to achieve equity.'(p. 6)
Te Tāhū Hauora Health Quality & Safety Commission. Guide to doing a learning review.[91]	2023	To enable health providers to review harm by applying the learning review method, in which the reviewer aims 'to understand the realities of everyday work to uncover how harm occurs, minimise risk in the system and support health care workers to do the right thing'(p. 3), including identifying areas for improvement. This method is influenced by concepts such as human factors and resilient healthcare, and takes a systems approach to safety.
Te Tāhū Hauora Health Quality & Safety Commission. National adverse events reporting policy.[92]	2023	The purpose of the policy is to provide 'a consistent way to understand and improve through reporting, reviewing and learning from all types of harm.' (p. 5). It is intended to guide reporting to Te Tāhū Hauora [Health Quality and Safety Commission], and the use of information from learning reviews, and quality improvement approaches to strengthen system safety. The policy differs from the previous policy by shifting the focus to 'system safety' and restorative response in the context of partnership with consumers and whānau.
The National Collaborative for Restorative Initiatives in Health. He Maungarongo ki Ngā Iwi: Envisioning a restorative health system in Aotearoa New Zealand.[93]	2023	Provides an overview of the systems that mitigate and respond to healthcare harm in Aotearoa NZ, adopting a human-centred and relational approach. It presents a framework of principles and practices and defines restorative systems as being 'distinguished by an emphasis on relational principles, practices and goals that promote and restore human dignity and wellbeing.'(p. 10)

[a]The original NTA 8009 document was updated twice, in 2011 and 2014, before the most recent NEN 8009 (2018) version.

[*]In Dutch, accessed in translation.

For peer review only - http://bmjopen.bmj.com/site/about/guidelines.xhtml

Supplementary material 5: Search information

Medline via Ovid, 8/12/23:

1 (safety adj3 management adj3 system*1).ti,ab. (588)
2 (safety adj3 management adj3 program*).ti,ab. (93)
3 1 or 2 (670)
4 (patient*1 adj2 (safety or harm* or risk*)).ti,ab. (204300)
5 patient harm/ or patient safety/ (25896)
6 4 or 5 (219200)
7 ((safety or hazard* or risk or risks) adj2 (accountability or assess* or assurance* or commitment* or communicat* or control* or coordinat* or cultur* or education or framework* or guidance or implement* or improvement* or leadership* or management or measurement* or monitor* or plan or plans or planning or policy or policies or promot* or quality or regulat* or reporting or responsibilit* or strateg* or training)).ti,ab. (285563)
8 *Safety Management/ (13937)
9 7 or 8 (295644)
10 system*1.ab. /freq=2 (1032216)
11 system*1.ti. (682356)
12 10 or 11 (1448707)
13 6 and 9 and 12 (3003)
14 3 or 13 (3632)

Embase via Ovid, 9/2/2024

1 (safety adj3 management adj3 system*1).ti,ab. 924
2 (safety adj3 management adj3 program*).ti,ab. 128
3 1 or 2 1038
4 (patient*1 adj2 (safety or harm* or risk*)).ti,ab. 343333
5 patient safety/ or patient harm/ or patient risk/ 173057
6 4 or 5 469009
7 ((safety or hazard* or risk or risks) adj2 (cultur* or education or implement* or improvement* or management or measurement* or

For peer review only - http://bmjopen.bmj.com/site/about/guidelines.xhtml

monitor* or quality or regulat* or reporting or responsibilit* or strateg* or training)).ti,ab. 123921
8 System*1.ab. /freq=2 1290950
9 System*1.ti. 770274
10 8 or 9 1735239
11 6 and 7 and 10 3017
12 3 or 11 3986
13 limit 12 to conference abstract status 996
14 12 not 13 2957

CINAHL via EBSCOhost, 9/2/2024

#	Query	Results
S8	S1 OR S7	2,564
S7	S3 AND S4 AND S6	2,373
S6	S2 OR S5	107,816
S5	(MM "Patient Safety")	38,651
S4	TI (system or systems) OR AB (system or systems)	516,337
S3	TI (((safety or hazard* or risk or risks) N1 (cultur* or education or implement* or improvement* or management or measurement* or monitor* or quality or regulat* or reporting or responsibilit* or strateg* or training)).) OR AB (((safety or hazard* or risk or risks) N1 (cultur* or education or implement* or improvement* or management or measurement* or monitor* or quality or regulat* or reporting or responsibilit* or strateg* or training)).)	37,449
S2	TI ((patient* N1 (safety or harm* or risk*))) OR AB ((patient* N1 (safety or harm* or risk*)))	81,650

For peer review only - http://bmjopen.bmj.com/site/about/guidelines.xhtml

S1	TI safety N3 management N3 system* OR AB safety N3 management N3 system*	238

Web of Science: 10/2/2024

- WOS.SCI: 1900 to 2024
- WOS.AHCI: 1975 to 2024
- WOS.ESCI: 2015 to 2024
- WOS.ISTP: 1990 to 2024
- WOS.SSCI: 1900 to 2024
- WOS.ISSHP: 1990 to 2024

Searches:
1: TS=(safety near/1 management) AND TS=((management near/1 (system or systems))) Results:3363
2: TI=((patient* near/2 (safety or harm* or risk*))) Results: 61188
3: TS=(safety near/1 (cultur* or education or implement* or improvement* or management or measurement* or monitor* or quality or regulat* or reporting or responsibilit* or strateg* or training)) Results: 78695
4: TS=(hazard* near/1 (cultur* or education or implement* or improvement* or management or measurement* or monitor* or quality or regulat* or reporting or responsibilit* or strateg* or training)) Results: 8001
5: TS=(risk near/1 (cultur* or education or implement* or improvement* or management or measurement* or monitor* or quality or regulat* or reporting or responsibilit* or strateg* or training)) Results: 122887
6: TS=(risks near/1 (cultur* or education or implement* or improvement* or management or measurement* or monitor* or quality or regulat* or reporting or responsibilit* or strateg* or training)) Results: 122887
7: TS=(system or systems) Results: 11251037
8: #6 OR #5 OR #4 OR #3 Results: 205099
9: #7 AND #8 AND #2 Results: 1288
10: TS=(patient* or health* or hospital*) Results: 11662421
11: #10 AND #1 Results: 1116
12: #9 OR #11 Results: 2379

For peer review only - http://bmjopen.bmj.com/site/about/guidelines.xhtml

Details of website searches of patient safety organisations in included countries

Organisation	URL	Search date		No. of hits	No. of includes
**Australia**					
Australian Commission on Safety and Quality in Health Care	https://www.safetyandquality.gov.au/	01/12/2023	safety management system	141	5
Department of Health and Aged Care	https://www.health.gov.au/	06/12/2023	safety management system	171	0
Australian Institute of Health and Welfare	https://www.achs.org.au/	06/12/2023	safety	63	0
Australian Council on Healthcare Standards	https://www.achs.org.au/	06/12/2023	safety	43	0
**Canada**					
Healthcare Excellence Canada	https://www.healthcareexcellence.ca/en	05/01/2024	safety	406 (200 screened)	4

For peer review only - http://bmjopen.bmj.com/site/about/guidelines.xhtml

Institute for Safe Medication Practices (ISMP) Canada	https://ismpcanada.ca	05/01/2024	safety management system	11	0
**Ireland**					
Health Information and Quality Authority (HIQA)	https://www.hiqa.ie	01/02/2024	Publications - safety	104	4
National Patient Safety Office, Ireland	https://www.gov.ie/en/campaigns/af4b6-national-patient-safety-office/	01/02/2024	safety	44	0
National Quality and Patient Safety Directorate	https://www2.healthservice.hse.ie/organisation/nqpsd/	01/02/2024	n/a	No search function	3
**Netherlands**					
NIVEL (The Netherlands Institute for Health Services Research)	https://www.nivel.nl/en	01/02/2024	safety management	76	2
Dutch Patient Safety Programme	https://www.vmszorg.nl/	05/12/2023		In Dutch	2
Netherlands Health and Youth Care Inspectorate, Ministry of Health Welfare and Sports	https://english.igj.nl	05/12/2023	safety	56	0
**New Zealand**					

For peer review only - http://bmjopen.bmj.com/site/about/guidelines.xhtml

Te Tāhū Hauora Health Quality and Safety Commission	https://www.hqsc.govt.nz/	01/02/2024	Resource library - safety management system	115	9

For peer review only
